# Genome-Wide Identification, Evolutionary Characterization, and Salt-Stress Expression Profiling of Antimicrobial Peptide Gene Families in the Xerophytic Recretohalophyte *Reaumuria soongarica*

**DOI:** 10.3390/genes17091126

**Published:** 2026-09-16

**Authors:** Youwu Wang, Shuihong Chen, Amjad Hussain, Xiufeng Han

**Affiliations:** 1 College of Agriculture, Tarim University, Alaer 843300, China; wangyw1975@126.com; 2 College of Life Sciences and Technology, Tarim University, Alaer 843300, China; rainbow@taru.edu.cn; 3 Department of Botany, Government College University, Hyderabad 71000, Pakistan; amjadhussainla@outlook.com

**Keywords:** *Reaumuria soongarica*, antimicrobial peptides, plant defensin, Snakin/GASA, non-specific lipid transfer protein, sodium sulfate, gene-family curation, phylogenetic sensitivity, RNA sequencing, Tamaricaceae

## Abstract

**Background/Objectives:** Antimicrobial peptides (AMPs) contribute to plant innate immunity and abiotic-stress adaptation, but their repertoires remain poorly characterized in extremophytic plants. We aimed to define AMP gene families and their expression under Na_2_SO_4_ treatment in the xerophytic recretohalophyte *Reaumuria soongarica*. **Methods:** Six canonical AMP families were screened using hidden Markov model and homology searches, domain verification, gene-model and transcript-support assessment, nsLTP boundary curation, phylogenetic and synteny analyses, and reanalysis of public RNA-sequencing data from 0, 200, and 400 mM Na_2_SO_4_ treatments. **Results:** We identified 73 candidates: 15 defensins, 18 Snakin/GASA genes and 40 nsLTPs, including seven previously unannotated, transcript-supported models. *RsLTP20* was reported separately as a reference-dependent C4-like candidate. Four sequence and alignment strategies supported two defensin, one Snakin/GASA and three nsLTP local groups. No tested promoter pattern was significantly enriched after multiple-testing correction. At 400 mM Na_2_SO_4_ relative to the control, 18 of the 66 catalogue members represented in the quantification reference were differentially expressed (12 upregulated and six downregulated; BH-adjusted *p* < 0.05 and |log_2_ fold change| > 1). Comparison with 38 *Tamarix austromongolica* candidates identified 27 reciprocal-best-hit pairs; genomic collinearity supported 25 direct candidate pairs. A separate Arabidopsis comparison recovered 26 candidate-anchored pairs. **Conclusions:** This curated catalogue defines conservative phylogenetic groups and provides candidates for experimental analysis of stress adaptation and peptide function.

## 1. Introduction

Antimicrobial peptides (AMPs) are a structurally diverse group of small, usually cationic and cysteine-rich peptides that form a conserved arm of plant innate immunity [1,2]. Stabilized by multiple disulfide bridges, they adopt compact folds that are resistant to proteolysis and physicochemical extremes. Beyond activity against bacteria, fungi, and oomycetes, some plant AMPs, particularly cyclotides, also have insecticidal functions. A genome-wide study in maize identified cyclotide-family candidates and AMP expression patterns associated with fungal or insect resistance [3]. Several AMP families also function in abiotic-stress responses, developmental signalling, and membrane lipid trafficking [4,5]. Plant AMPs are conventionally grouped by structural scaffold and Pfam domain into families including defensins (PF00304, cysteine-stabilized αβ/CSαβ fold), thionins (PF00321), Snakin/GASA peptides (PF02704), non-specific lipid transfer proteins (nsLTPs; PF00234, prolamin superfamily), hevein-like chitin-binding peptides (PF00187), and cyclotides (PF03784) [6].

Among these families, nsLTPs and defensins are of particular interest in stress biology. nsLTPs, defined by an eight-cysteine motif (8CM) that supports a hydrophobic cavity, participate in cutin and wax assembly, cell-wall remodelling, and systemic signalling, processes central to maintenance of the apoplastic barrier under drought and salinity [4,7,8]. Plant defensins combine direct antimicrobial activity with reported roles in ion homeostasis and abiotic-stress signalling [9]. Snakin/GASA peptides, which respond to gibberellin and abscisic acid (ABA), connect redox and stress signalling with growth [10].

Defensins and Snakin/GASA peptides have relatively distinctive cysteine-rich architectures, whereas nsLTP discovery is complicated by the broad-prolamin superfamily. The PF00234/PF14368 sequence space includes proteins with overlapping cysteine frameworks but different biological identities, such as 2S seed-storage proteins and alpha-amylase/trypsin inhibitors. Family membership therefore cannot be inferred from a broad domain or a single similarity hit alone. Published surveys combine profile or homology retrieval with checks of the eight-cysteine motif, secretion signals and alternative-family assignments, and apply comparable rules across species [11,12]. Conversely, small secreted peptide genes are often under-predicted in plant genomes [6]. Family surveys therefore need to distinguish related protein families and search for loci missing from the reference annotation.

*R. soongarica* (Pall.) Maxim. (Tamaricaceae) is a small xerophytic shrub that dominates desert and semi-desert communities across arid Central Asia. It is a recretohalophyte that excretes excess salt through specialized glands and tolerates extreme drought, temperature, and salinity, making it an ecologically important sand-fixing species and a model for dissecting multi-stress adaptation [13,14]. Its AMP small-peptide repertoire remains uncharacterized despite the availability of a chromosome-level genome assembly (1.28 Gb, with 11 chromosomes; 21,791 protein-coding genes) and a public Na_2_SO_4_-treatment transcriptome [15]. The same public expression dataset has been used to characterize the AP2/ERF transcription-factor family [16], whereas AMP families and their family-specific boundaries have not been evaluated.

Here, we present a genome-wide identification and characterization of canonical plant AMP families in *R. soongarica*. We (i) screened six Pfam-defined AMP families across the proteome and examined genomic and transcript evidence for candidate loci missing from the annotation; (ii) assessed nsLTP membership using domain, sequence, secretion and membrane-topology evidence; (iii) characterized physicochemical properties, predicted subcellular localization, phylogenetic relationships, gene architecture, conserved motifs, chromosomal distribution, duplication modes, within-species collinearity, selective pressures and upstream sequence patterns; (iv) examined *Arabidopsis thaliana* collinearity, and compared family composition and synteny with the close Tamaricaceae relative *T. austromongolica*; and (v) profiled differential expression across a Na_2_SO_4_ dose gradient. We also compared expression changes in selected homologous candidates using a separate public NaCl-treatment dataset from *Tamarix chinensis*. These analyses provide candidates for experimental investigation of plant salt-stress responses and peptide function.

## 2. Materials and Methods

### 2.1. Genome, Annotation, and Public Sequencing Data

The *R. soongarica* chromosome-level assembly, annotation, protein set, and coding sequences were obtained from Figshare (10.6084/m9.figshare.25533064.v2) and GenBank assembly JBEBFM000000000, associated with the genome report [15]. File integrity was checked against repository checksums. The assembly spans 1,281,161,807 bp, including 1,254,936,269 bp on 11 chromosomes and 26,225,538 bp in unplaced sequences and contains 21,791 predicted genes. Public RNA-seq runs SRR27540875-SRR27540881, SRR27540883, and SRR27540884 (BioProject PRJNA1063761) and the Iso-Seq run SRR27540882 were downloaded from the NCBI Sequence Read Archive. FASTQ gzip integrity, mate counts, sequence counts, and checksums were verified before analysis.

### 2.2. Identification of AMP-Family Members

Defensin (PF00304), thionin (PF00321), Snakin/GASA (PF02704), nsLTP/prolamin (PF00234/PF14368), hevein-like (PF00187), and cyclotide (PF03784) families were screened with HMMER 3.4 [17], BLAST+ 2.17.0 [18], curated plant reference sequences, and InterProScan 5.77-108.0 [19]. A genome-wide tblastn search used 128 references (63 defensins, five thionins, and 60 Snakin/GASA; E < 1 × 10^−5^); clusters of high-scoring segment pairs (HSPs) were compared with the reference annotation to distinguish gene-span overlap from evidence for a complete same-strand coding model. Candidates with a complete reference-annotated gene model or a complete reconstructed model supported by genomic and transcript evidence, together with family-specific evidence, formed the primary catalogue. Genomic fragments encoding an AMP-like open reading frame (ORF) but lacking a supported complete gene model were listed separately and excluded from analyses of the primary catalogue. Throughout the manuscript, “Snakin/GASA” denotes the family, “Snakin/GASA genes” refers to genomic loci, and “Snakin/GASA proteins” or “peptides” refers to their encoded products.

Untrimmed protein sequences were analyzed, retaining the complete available sequence for each model. Predicted signal peptides were not removed before localization prediction. Signal peptides and cleavage positions were predicted with DeepSig 1.2.5 using the eukaryotic model (-k euk) [20]. Subcellular localization was predicted with WoLF PSORT 0.2 [21] in plant mode, with k = 14. After exact-sequence deduplication, 98 distinct proteins were analyzed. File S5 records the software revision, commands, input checksums and raw outputs. Sequences were linked to catalogue identifiers by exact amino-acid sequence identity; the reference-annotated candidates, broader nsLTP screening set and reconstructed models are identified separately in Table S39.

WoLF PSORT score profiles were retained without converting the scores to probabilities. Output categories sharing the highest score were recorded as tied predictions, and combined localization labels were preserved as returned by the program. DeepSig assesses secretory signal peptides, whereas WoLF PSORT predicts cellular compartments. For the nsLTP screening set and assessed reconstructed-model sequences, TMbed [22] was used to assess membrane topology and distinguish predicted N-terminal signal regions from additional transmembrane segments; PredGPI [23] was used to assess potential GPI-anchor signals where evaluated. TMbed and PredGPI results were linked to candidates by exact sequence identity; candidates without an assessment are distinguished from those with negative predictions. Disagreement among predictors was retained as uncertainty. Predicted localization alone was not used to establish family identity.

Physicochemical properties were calculated for the 73 complete, untrimmed protein sequences using Biopython 1.87 ProteinAnalysis: sequence length, average molecular weight, theoretical isoelectric point (pI), net charge at pH 7.0, grand average of hydropathy (GRAVY), instability index and cysteine content. The aliphatic index was calculated as 100 × [n(A) + 2.9n(V) + 3.9(n(I) + n(L))]/L, where n denotes residue counts and L is sequence length. These are theoretical precursor-sequence properties; signal-peptide cleavage, disulfide formation and other post-translational modifications were not modelled. Member-level values and family summaries are provided in Tables S51 and S52.

### 2.3. nsLTP Family Assignment

nsLTP candidates were assessed using the same ordered criteria in both species, informed by multistep family surveys [11,12]. Broad PF00234/PF14368 retrieval or IPR016140/IPR036312 annotation alone does not establish membership. Within the classical secreted eight-cysteine scope, a compatible core and secretion architecture must accompany a specific family-profile match or qualifying reviewed-reference core homology. Explicit alternative-family evidence is distinguished from unresolved broad-prolamin annotation. Predicted GPI attachment defines a potential subtype, not a requirement for all nsLTPs; retained transmembrane segments and conflicts between the core and predicted cleavage are flagged separately. Tables S30–S33 and File S6 record family evidence, model provenance and primary or extended membership for each candidate. For structural comparison, the positions of the eight core cysteines were checked against each complete sequence, and spacing was recorded as the number of intervening residues between successive core cysteines. Core spacing and predicted GPI-anchor architecture are reported separately from phylogenetic group membership (Table S56).

IPR044741/CDD cd04660 is treated consistently as evidence for an nsLTP-like subgroup in both annotation-derived and reconstructed candidates. Its matches span the complete selected core in the two profile-dependent primary candidates, *RsLTP29* and Tamarix evm.model.Chr05.234; both are identified as depending on this single profile. Signature/core coordinate checks distinguish whole-domain matches from local PRINTS or PROSITE fingerprints. Failure of a local pattern to cover the entire core is not treated as absence of the domain.

Candidates whose inclusion depended on a single positive reference were listed separately in an extended catalogue. Omitting Q6ZFW0 from the similarity results removed qualifying positive-core support for *RsLTP20* and Tamarix evm.model.Chr10.1284; both are therefore kept outside the primary count and reported as C4-like, reference-dependent candidates. Primary membership was unchanged when this reference was omitted. The same rules and flags apply to both species. Section 2.9 specifies shared retrieval and similarity thresholds; File S6 records versions, candidate-level decisions and the effects of alternative inclusion criteria.

### 2.4. Assessment of Six Genomic AMP-like Fragments

Six AMP-like peptide fragments identified by genome-wide tblastn were checked for exact genome encoding, strand-aware upstream in-frame methionines, downstream stop codons, overlap or gaps in the reference GFF annotation, and short-read coverage in all nine RNA-seq libraries. Iso-Seq evidence was assessed from 17,136,488 reads (62.35 Gb) using a two-stage procedure: local-window screening followed by global genome remapping of candidate reads with minimap2 [24]. Candidate-region transcript models were assembled with StringTie2 [25]. A locus was included in the primary catalogue only if the coding sequence was incorporated into a coherent, complete gene model supported by genomic context and transcript evidence.

### 2.5. Phylogenetic Analysis

Maximum-likelihood trees were inferred for the catalogue members, reference proteins and related-family controls, including the reviewed OsC4 reference Q6ZFW0. The family inputs contained 110 defensin, 43 Snakin/GASA and 159 nsLTP sequences, respectively, and included all 113 extended-catalogue members of the two species. All 73 primary *R. soongarica* members and the extended candidate *RsLTP20* were represented. Four strategies per family compared full precursors and an operational core bounded by the first and last cysteine, each analyzed with an untrimmed or trimAl v1.5.rev1 [26] gappyout alignment. This core is an analytical sequence segment, not an experimentally mapped mature peptide. MAFFT v7.526 [27] used local-pair alignment and 1000 refinement iterations. IQ-TREE 3.1.2 [28] used ModelFinder [29] with the default candidate model set (MFP), 1000 SH-aLRT replicates, 1000 ultrafast bootstrap replicates with BNNI optimization [30,31], four threads per inference and seed 20260909.

A stable local group required an identical all-taxon unrooted bipartition in all four strategies, each with SH-aLRT ≥ 80% and UFBoot ≥ 95%, and at least two primary *R. soongarica* members on the local side, with at least one primary member outside that side. *R. soongarica*-restricted grouping alone was insufficient. Six non-overlapping qualifying groups were selected for display; all qualifying splits, including nested groups, are listed in Table S7.

The full-precursor, gappyout-trimmed trees were used for the main display. Figure 2 includes all primary *R. soongarica* members, *RsLTP20*, their nearest reference sequences and all taxa in highlighted groups; branches failing the joint support threshold are collapsed. Figure S1 provides the complete trees, support values and branch lengths. Circular radius is a cladogram layout, not evolutionary distance or evidence for a biological root. Family admission is evaluated separately from assignment to a supported sequence group [32].

Rooting feasibility was assessed independently in 16 additional unconstrained ML analyses (File S2). Candidate outgroups comprised 10 gymnosperm defensins, three Selaginella GASA proteins, and eight 2S proteins. The defensin candidates were identified by PF00304, a length of 40–180 amino acids and eight cysteines; two were present in the family-reference set, and eight additional candidates were included. Selaginella PF02704 candidates were filtered to 70–350 amino acids and deduplicated by their cysteine-delimited cores. Defensin and GASA were each tested under the four full/core and untrimmed/gappyout combinations. For nsLTP, the ingroup was fixed at 36 *R. soongarica* members, 24 Tamarix members and 48 positive references (108 sequences). All eight 2S candidates were tested under four strategies; the three Medicago and five Arabidopsis subsets were each tested with full and core gappyout alignments, yielding eight nsLTP analyses. The Arabidopsis 2S entries were reviewed; the Medicago and Selaginella entries were unreviewed. These tests used MAFFT L-INS-i, trimAl gappyout and IQ-TREE 3.1.2, with ModelFinder, 1000 SH-aLRT and 1000 UFBoot replicates, BNNI and seed 20260908. A conditional root was assigned only when an exact unrooted bipartition separated the designated candidate set from the ingroup. Basal partitions were compared for the same ingroup taxa. Candidate provenance, model settings, commands, unrooted outputs and conditional trees are supplied in File S2.

### 2.6. Gene Structure, Motifs, Chromosomal Distribution, and Duplication

Members were mapped to chromosomes using their coding coordinates, and exon–intron structures were derived from the reference annotation or reconstructed transcript models (Table S46). Chromosome lengths share one megabase scale; gene density uses 1 Mb midpoint windows from the 21,339 reference genes anchored to the 11 chromosomes. No centromere locations were inferred. Structures retain source-annotated untranslated regions (UTRs) where recorded. For seven reconstructed models, assembled exon portions outside the CDS and terminal stop codon are labelled as inferred noncoding portions, without claiming an independently annotated UTR or TSS. Models are displayed in the 5′-to-3′ direction and normalized independently to their full source spans; genomic span lengths are also reported.

Conserved sequence motifs were identified independently with MEME 5.5.9 [33] on 15 defensin, 18 Snakin/GASA, and 40 nsLTP complete precursor sequences using -protein -nmotifs 5 -minw 6 -maxw 50 -mod zoops -maxsize 200,000. Motif sites were displayed at *p* ≤ 10^−4^. All five requested motifs per family are displayed, with motif-level E-values retained and weak motifs identified as exploratory. Motif ranks are family-specific and do not imply homology across families.

Duplication modes were assigned from the genome-wide MCScanX gene classification [34], using codes 0–4 for singleton, dispersed, proximal, tandem and whole-genome/segmental duplication, respectively. Protein identifiers, exact sequences and genomic coordinates were checked before extracting assignments for the 66 reference-annotated primary members (Tables S53 and S54). The seven reconstructed models were absent from this classification reference and were recorded as not assessed. The 23 candidate–candidate pairs used for Ka/Ks analysis (16 tandem and seven segmental pairs involving 33 members) form a separate pair-level dataset. The seven segmental pairs were matched to their original within-species collinearity blocks and anchor records (Table S55). The recorded collinearity parameters were MATCH_SCORE = 50, MATCH_SIZE = 5, GAP_PENALTY = −1, OVERLAP_WINDOW = 5, E_VALUE = 1 × 10^−5^ and MAX GAPS = 25. The genome-wide classification reference comprised 21,791 annotated proteins.

### 2.7. Estimation of Ka/Ks

Protein-guided codon alignments were evaluated before using YN estimates from KaKs_Calculator [35]. Pairs were excluded from inferential summaries when a member was outside the 33-member duplication subset, the CDS did not translate consistently, the usable codon alignment was shorter than 150 nt, estimates were non-finite, fewer than 10 substitutions were available, or dS suggested saturation (>2). Because AMP coding regions are short, retained estimates were described with short-peptide caution. Ka/Ks < 1 was considered consistent with purifying selection only when the associated Fisher test had *p* < 0.05; no individual estimate was treated as proof of selection.

### 2.8. Promoter Motif Occurrence and Enrichment

For each of the 73 primary *R. soongarica* members, we extracted a strand-aware 2 kb upstream interval as a putative promoter proxy. Boundaries were taken from the reference gene annotation for 66 members and from the supported reconstructed transcript-model spans for seven reconstructed members. The latter spans include the modelled transcript extremities and are not the CDS start coordinates. Transcription start sites were not independently mapped; the seven new models also lack independently annotated 5′UTRs. Full-length denotes an extracted interval of exactly 2000 bp. The panel contains 14 double-stranded IUPAC patterns, including the single merged TGACG/CGTCA core; exact sequences, aliases, sources and interpretation limits are listed in Table S27. This finite sequence-pattern panel is not a complete database or PWM scan. Overlapping matches were allowed, and identical forward/reverse-complement coordinates were counted once within each pattern. Target and background sequences were extracted from the genome and scanned with the same patterns.

The background comprised the 21,791 reference-annotated genes after removal of all 66 official-annotation target genes; the seven new target models were never background members. Of the 21,725 non-target genes, 21,695 provided complete upstream intervals. The primary comparison therefore used 73 target and 21,695 background intervals. Two sensitivity comparisons applied identical rules to both cohorts: S1 additionally required only A/C/G/T bases and no overlap with another gene body on either strand, using the union of the 21,791 official gene spans and the seven new model spans; S2 additionally required a linked 5′UTR feature in the reference annotation. Excluded targets never entered the background.

One-sided Fisher exact tests evaluated greater motif prevalence, with Benjamini–Hochberg correction over 14 patterns within each scenario and, additionally, over all 42 comparisons; enrichment required a BH-adjusted *p* < 0.05. Tables S9 and S10 provide occurrences and primary tests, Table S28 documents target coordinates and quality flags, and Table S29 reports all three scenarios. These checks do not fully match sequence composition or evolutionary dependence. Sequence matches do not establish transcription-factor binding or treatment-specific regulation.

### 2.9. Comparative Genomics and Interspecies Collinearity

*T. austromongolica* was selected as a close Tamaricaceae comparator with a chromosome-level genome and public assembly and annotation provenance (GCA_039764185.1; 1.33 Gb, 12 chromosomes, 22,374 predicted proteins, BUSCO completeness 98.2%) [36]. The primary catalogues contain 73 *R. soongarica* and 38 *T. austromongolica* candidates. The sources of the gene models differ: *R. soongarica* includes seven transcript-supported reconstructed models, whereas all five Tamarix defensins are additional homology-derived models without transcript validation. Annotation absence is not biological absence. Defensin and Snakin/GASA membership requires complete gene models and family-specific evidence; nsLTP candidates in both species follow the shared family rules. The extended set adds one reference-dependent C4-like candidate per species. These resources are not presented as equally exhaustive species-wide censuses.

nsLTP screening used PF00234/PF14368 profiles and reviewed nsLTP homology, then evaluated secretion, a domain-localized eight-cysteine framework, family-specific InterPro evidence, explicit alternative-family references and membrane topology. The classical framework required adjacent C3/C4 and C5-X-C6. Core-homology support required BLASTP E ≤ 1 × 10^−5^, identity ≥ 30%, coordinate coverage ≥ 80% on both sequences and alignment of all eight cysteines. Family identity and GPI subtype were evaluated separately. Ambiguous broad-prolamin references were not treated as confirmed 2S proteins. Two shared core-tree strategies, untrimmed and gappyout, served as sensitivity checks rather than independent validation. The four-strategy analyses in Section 2.5 separately assess phylogenetic groups within each family. The operational rules, 99-candidate evidence matrix, inclusion decisions, threshold checks and reference-omission analysis are supplied in File S3. These thresholds define this comparison and are not claimed to identify every atypical nsLTP.

For cross-species sequence comparison, BLASTP 2.9.0+ [18] was performed for all 113 extended-catalogue queries against 44,177 proteins: the 21,791 *R. soongarica* and 22,374 Tamarix reference proteins, five Tamarix defensin homology models and seven reconstructed *R. soongarica* models. Searches used E ≤ 1 × 10^−5^, SEG enabled, composition-based statistics 2 and max_target_seqs 50000. A reported pair required unique opposite-species best matches in both directions, at least 50% coordinate coverage on each protein and retained membership at both ends; tied best matches were not resolved arbitrarily. Annotation-based counts per 10,000 reference proteins exclude additional models from the numerator. For the *R. soongarica*–*T. austromongolica* collinearity analysis, the reference proteins and genomic coordinates were supplemented with the five homology-derived *Tamarix* defensin models, giving 44,170 input records. The seven newly reconstructed *R. soongarica* models were not included in this input. Whole-genome MCScanX blocks were then filtered for members of the current catalogues. We report both candidate-involving pairs and pairs with primary candidates at both ends. This genomic comparison concerns inventories, sequence similarity and collinearity. Separate public *T. chinensis* analyses provide annotation-based expression context (Section 2.13) and a supported candidate-pair comparison (Section 2.14). These limited comparisons do not constitute a systematic survey across ecological groups. Sequence-comparison and collinearity results are provided in File S8, with supporting family-assignment evidence in File S3.

A separate collinearity comparison used the *A. thaliana* TAIR10.1 reference assembly (GCF_000001735.4; TAIR and Araport annotation). The reference contained 27,444 representative proteins from the five nuclear chromosomes, with one mRNA selected per locus by its greatest genomic span. Bidirectional DIAMOND 2.1.18 [37] searches between these proteins and the 21,791 *R. soongarica* reference proteins used E ≤ 1 × 10^−5^, up to five hits per query and 16 threads, followed by MCScanX [34] with default parameters. The resulting genome-wide blocks were re-examined against the current *R. soongarica* catalogue. Arabidopsis partners were identified by their RefSeq protein accessions and locus tags; they were not treated as a separately curated AMP-family inventory. Pair records, reference coverage, and the original execution records are provided in Tables S57 and S58 and File S11. The seven reconstructed *R. soongarica* models were absent from this anchor reference.

### 2.10. RNA-Seq Quantification and Differential Expression

The source RNA-seq experiment sampled tender leaves following 0 (CK), 200 (S200), or 400 mM (S400) Na_2_SO_4_ treatment, with three biological replicates per condition [15]. The original data paper reports cultivation at 26 °C during the day and 16 °C at night, a 16 h light/8 h dark cycle, and half-strength Hoagland solution every three days [15]. A later study states that it followed the earlier transcriptome sampling protocol and describes eight-week-old seedlings, one week of treatment and eight seedlings per replicate [16]. Treatment metadata and source attributions are provided in Table S25. fastp 1.3.3 [38] provided read-level QC. Salmon 1.10.3 [39] quantified all nine libraries against a decoy-aware index of 21,791 transcripts and 327 genomic decoys (22,118 targets) using library-type ISR and --keepDuplicates. Transcript estimates were aggregated with tximport [40] (GFF mRNA ID/Parent mapping; countsFromAbundance = “no”) and imported using DESeqDataSetFromTximport. Genes with total integer counts <10 across nine libraries were prefiltered (Supplementary Table S26). DESeq2 [41] used a treatment-group design and Wald tests, with default independent filtering; no sample was deleted. DE genes required Benjamini–Hochberg-adjusted *p*-value (FDR) < 0.05 and absolute unshrunken maximum-likelihood log_2_ fold change > 1. apeglm-shrunken estimates [42] were used for an additional sensitivity display; the main volcano plot and differential-expression calls use unshrunken fold changes.

Results for the 66 reference-annotated primary members, including *RsLTP2*, *RsLTP18* and *RsLTP30*, were extracted from the whole-transcriptome analysis using the transcript-to-gene map. Thus, multiple-testing correction was performed genome-wide before selecting AMP-family results. The extended annotation-derived set adds *RsLTP20*. The seven reconstructed models were absent from the Salmon reference and were not included in the differential-expression analysis. Figures 6 and 7, the main expression displays, show members represented in the quantification reference. Tables S12–S15, S23 and S26 retain all 73 catalogue members with their quantification availability, count-filter results and contrast-specific testing status; Tables S36–S38 provide results for the 66 reference-annotated members.

Variance-stabilized values (blind = FALSE) were standardized within each row across nine libraries using the population standard deviation; rows retain family/catalogue order without clustering. Transcript mappability was assessed for all 66 reference-covered members against the full transcriptome using near-identity searches and Salmon ambiguity diagnostics; this does not establish primer specificity. Transcript query coverage uses the inclusive aligned query-coordinate span divided by full query length, rather than the gapped alignment length.

### 2.11. Classification of Transcriptome-Unassigned and Genome-Unmapped Reads

Read pairs not assigned by Salmon were recovered from the FASTQ files passing the integrity checks in Section 2.1 and were quality-filtered with fastp. They were classified in a fixed hierarchy by exact 27-mer matching to SILVA 138.2 SSU/LSU rRNA [43], followed by 31-mer screens allowing one mismatch against the *R. soongarica* chloroplast genome (NC_041273) and a *Myricaria laxiflora* mitochondrial sequence proxy (MW971331). Residual pairs were intersected with the independent STAR [44] genome-unmapped set and screened against labelled AMP references. Read categories were mutually exclusive within this hierarchy. The 107 ORF records obtained from genome-mapped, transcriptome-unassigned reads were classified by their relationship to reference gene models: known loci, overlapping models or intergenic regions. Complete model and transcript-support criteria are detailed below and in File S4; Tables S30–S34 and File S6 provide the unified names, independent-locus checks and analysis-set membership.

Ten candidate ORF models from genome-mapped, transcriptome-unassigned reads were examined in detail. Temporary identifiers CAND01–CAND10 distinguish these models; their final gene names and correspondence to source identifiers are provided in Table S33. The models were assessed for locus independence before counting genes. Model completeness, family identity and locus independence were evaluated separately using genomic back-translation, per-library transcript assemblies, coding-region and exact-junction support, and overlap with the reference annotation and the six tblastn-derived genomic fragments. Qualifying short-read junction support required primary uniquely mapped records (NH = 1), the correct transcript strand, MAPQ ≥ 20 and at least 10 aligned bases on each side of the exact junction. Coding-base checks additionally required base Q ≥ 20 and collapsed overlapping mates at each position. Local long-read support required a primary alignment with MAPQ ≥ 20, the exact local splice chain and at least 90% aligned-base coverage of each coding segment; this does not establish a full-length nonchimeric transcript. Family assessment combined curated Pfam gathering thresholds, InterProScan 5.77-108.0 signatures, cysteine framework, secretion and GPI predictions, and reviewed plant-reference comparisons. Inclusion in the primary catalogue required concordant family evidence and reproducible complete-model support without an unresolved coding-boundary or independent-locus conflict. Uncertain family assignments, alternative initiation boundaries and connecting models were retained separately. File S4 provides candidate-level criteria, sequences, processed evidence and the scope of additional assembly and recursive-search records. These reconstructed models were absent from the annotation-based Salmon reference and were not assessed for differential expression.

### 2.12. Reproducibility

Software versions, command-line scripts, sample metadata, alignments, phylogenetic trees, candidate-assessment tables and processed expression results are provided in Files S1–S11 and the Supplementary Tables workbook. Input identifiers, commands and checksum manifests document the analyses. Code and processed data are available at https://github.com/tarimteacher1/rsoamp-genes-revision (accessed on 11 August 2026), with version-specific links in the Data Availability Statement. Raw sequencing reads are available from the source repositories; BAM files, database indices and third-party software installations are not redistributed.

OpenAI Codex assisted with drafting and revising manuscript text, including the Methods and Discussion, developing and debugging analysis and plotting code, and checking data consistency and the supporting literature. AI-assisted content addressing scientific interpretation was assessed by the authors against the study data and original sources. The authors reviewed and edited the outputs, approved the final content, and took full responsibility for the work.

### 2.13. Public T. chinensis RNA-Seq Reanalysis for External Expression Context

We analyzed six public *T. chinensis* libraries from PRJNA855335 [45]: SS1–SS3 sampled after seven days at 300 mM NaCl and contemporaneous untreated SCS1–SCS3 controls (shoots; three biological replicates per group). The source study used clonally propagated material and a two-hour 200 mM NaCl pretreatment. Sample timing and library metadata were obtained from the source study and checked against ENA records (Table S40). Read counts matched the source study’s reported clean-read counts; the downloaded files therefore represent submitted processed reads. The source expression CSV did not specify units suitable for count-based inference and was not used as DESeq2 input.

Genome and annotation files were obtained from GigaDB (10.5524/102417). GFF Parent relationships connected transcripts to genes. One self-parented transcript absent from the official CDS and protein files was excluded, while all 26,426 gene loci were retained. The gffRead 0.12.9 [46] reconstructed 46,080 transcripts from the annotated exons; exon-only feature input prevented implicit boundary expansion at three inconsistent CDS/exon models. Excluded records and reference-construction details are provided in File S7. FASTQ size, MD5 and gzip integrity were verified. fastp produced reports without supplying modified reads to quantification. Archived quality scores showed extensive simplification (sampled first 10,000 R1 records per library all had Phred 30); the resulting Q30 values describe the submitted files and were not interpreted as original instrument quality.

Salmon 1.10.3 [39] used a full-genome decoy index with duplicate transcript sequences retained, automatic library-type inference, selective alignment and sequence/GC-bias correction. tximport 1.30.0 [40] aggregated transcript estimates through the GFF mapping with countsFromAbundance = “no”. DESeq2 1.50.2 [41] fitted ~ condition after requiring a summed integer estimated count of at least 10 across the six libraries. The Salt-versus-CK Wald contrast used genome-wide Benjamini–Hochberg adjustment, retaining default independent filtering and Cook’s handling. DE entries required BH-adjusted *p* (FDR) < 0.05 and observed absolute MLE log_2_FC > 1; apeglm 1.32.0 [42] estimates were used for display. This is an operational filter, not a test of an effect-size-threshold null hypothesis.

Before differential-expression analysis, we selected 41 source-study gene IDs whose published annotations in that study’s Tables S14–S17 [45] contained lipid-transfer-related wording (33) or GASA/gibberellin-regulated wording (8). Source annotations and all selected entries are listed (Table S41). Broad lipid-transfer annotations, including EARLI1/AZI1-related entries, were not relabelled as nsLTPs validated by the *R. soongarica* decision tree. Statistical tests were performed for the whole transcriptome before extracting results for the selected genes (Figure S2; Tables S42 and S43). Missing source-study values were distinguished from genes not tested in this analysis. A separate comparison of homologous candidate pairs is described in Section 2.14. Neither analysis provides a complete family census, a directly comparable family response rate or a species-effect test. Selection from the source DEG tables remains a limitation.

### 2.14. Expression Comparison of Homologous Candidates

The homologue-expression comparison used a subset of 63 reference-annotated *R. soongarica* candidates (nine defensins, 18 Snakin/GASA and 36 nsLTPs). *RsLTP2*, *RsLTP18*, *RsLTP30* and the seven reconstructed models were not included in this query set (Table S47). The *T. chinensis* reference was derived from CDS features in the official GigaDB annotation [45] using gffread 0.12.7. Of 46,081 extracted transcript records, the known self-parented orphan TC08G1595T2 lacked a valid gene mapping and was excluded. We retained 46,080 mapped records and the longest extracted protein per gene, using first occurrence for equal-length ties, covering all 26,426 annotated genes. All 24 *T. chinensis* target proteins identified by these queries matched the released sequence for the corresponding transcript. Reference checks and the complete analysis scope are supplied in Table S47 and File S9. Profile-screening hits were retained as retrieval evidence rather than a curated *T. chinensis* family census.

BLASTP 2.17.0+ [18] searches used E ≤ 1 × 10^−5^, SEG filtering, composition-based statistics mode 2, eight threads, and the standard 12-column tabular output. Searches specified max_target_seqs 20 for the forward search and five for the reverse search; the retained outputs contain 325 and 151 HSP rows, respectively. A qualifying reciprocal-best-hit pair required a unique highest bit-score target in each direction among the retained hits, together with at least 50% aligned-coordinate coverage of each protein in the *R. soongarica*-to-*T. chinensis* high-scoring segment pair (HSP). Inclusive coordinate spans were divided by the corresponding protein lengths. Reverse queries were searched against the 21,791-protein *R. soongarica* reference. Because these output-retention limits were applied, uniqueness remains conditional on the retained hits rather than on unrestricted search output.

For collinearity, DIAMOND 2.2.1 [37] protein-search outputs for the combined 48,217-protein reference were supplied to MCScanX [34]; the search used E ≤ 1 × 10^−5^, sensitive mode, 32 threads and up to ten reported targets, followed by MCScanX with default parameters. MCScanX version 1.0.0 (Bioconda build h9948957_0) was identified from package metadata and the executable checksum (File S9). The main expression set retained qualifying reciprocal pairs supported by an interspecies MCScanX anchor. A stricter sensitivity check additionally required both proteins to reach 50% coordinate coverage in the reverse HSP. Pair assignments, coverage values and the main and sensitivity subsets are listed in Tables S48–S50 and File S9.

Gene identifiers linked the whole-transcriptome estimates from the separate studies; samples were not pooled into a joint model. *R. soongarica* S200-versus-CK and S400-versus-CK effects were compared separately with the *T. chinensis* Salt-versus-CK effect using the models in Section 2.10 and Section 2.13. Each within-study DE call required genome-wide Benjamini–Hochberg-adjusted *p* (FDR) < 0.05 and absolute unshrunken maximum-likelihood log_2_ fold change > 1. Missing and untested values remained distinct from tested entries that did not meet this criterion. Tables retain all supported pairs and identify the conditional subset meeting the DE criterion in both studies. A pair represented in both *R. soongarica* contrasts contributes two comparison rows but one unique pair; repeated use of the same *T. chinensis* estimate is not independent replication. No species effect or strict one-to-one orthology is inferred.

## 3. Results

### 3.1. Genome-Wide Identification of AMP Families

**Figure 1:**
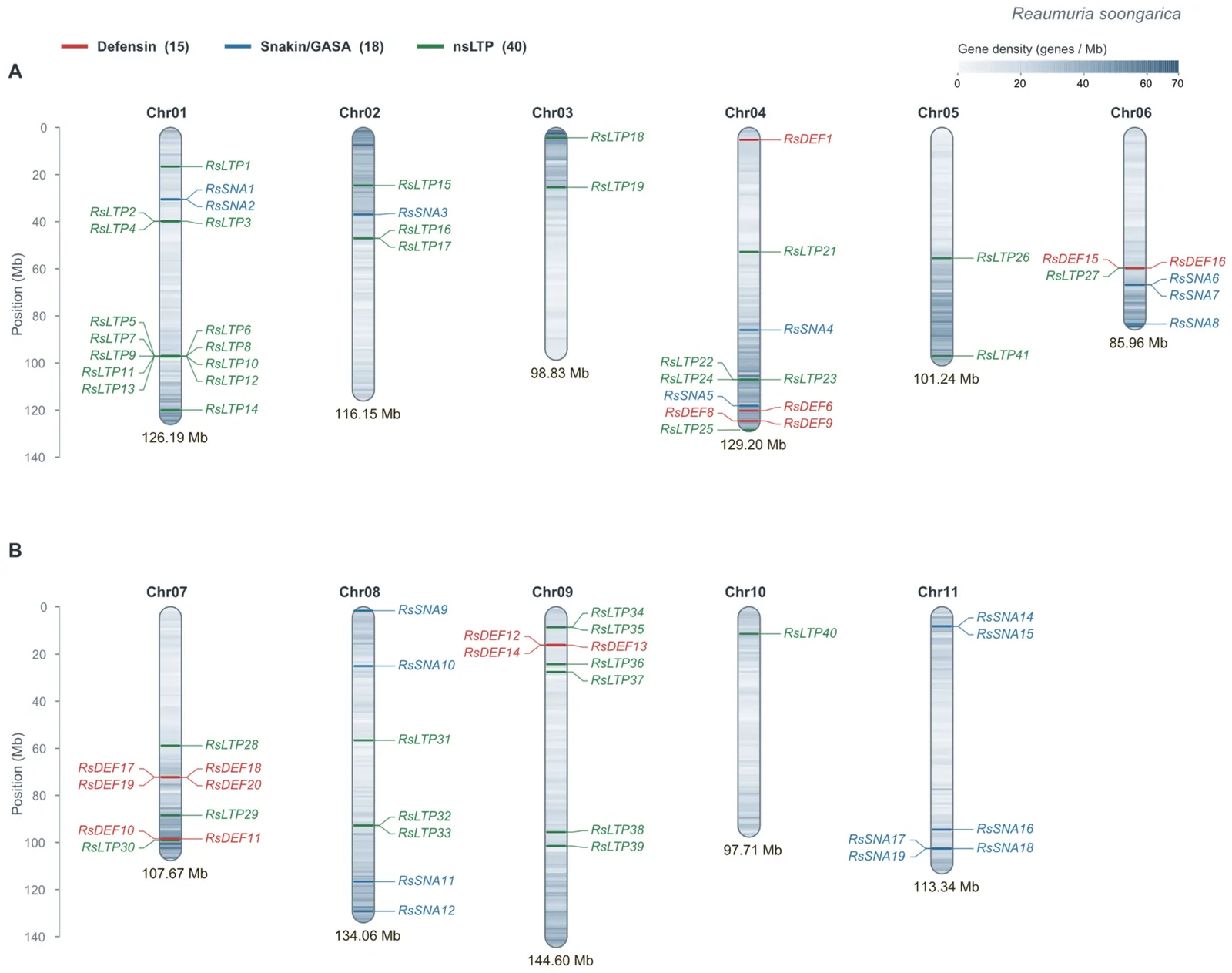
Chromosomal distribution of the 73 primary-catalogue AMP-family members in *Reaumuria soongarica*. The catalogue contains 15 defensins, 18 Snakin/GASA proteins and 40 nsLTPs. Chromosome rods use the chromosome lengths and a common megabase scale. Coloured labels identify the three families; leader lines connect each label to its genomic position. The density strip shows the number of genes in the source genome annotation per megabase, using 1 Mb windows and assigning each gene by its midpoint. Density is based on the 21,339 annotated genes anchored to the 11 chromosomes; the seven reconstructed models are shown as AMP-family members but are not included in this annotation-based density background. Centromere positions are not inferred. *RsLTP20* belongs to the extended catalogue and is not included in this primary-catalogue figure. Panels (**A**) and (**B**) show chromosomes Chr01–Chr06 and Chr07–Chr11, respectively.

**Figure 2:**
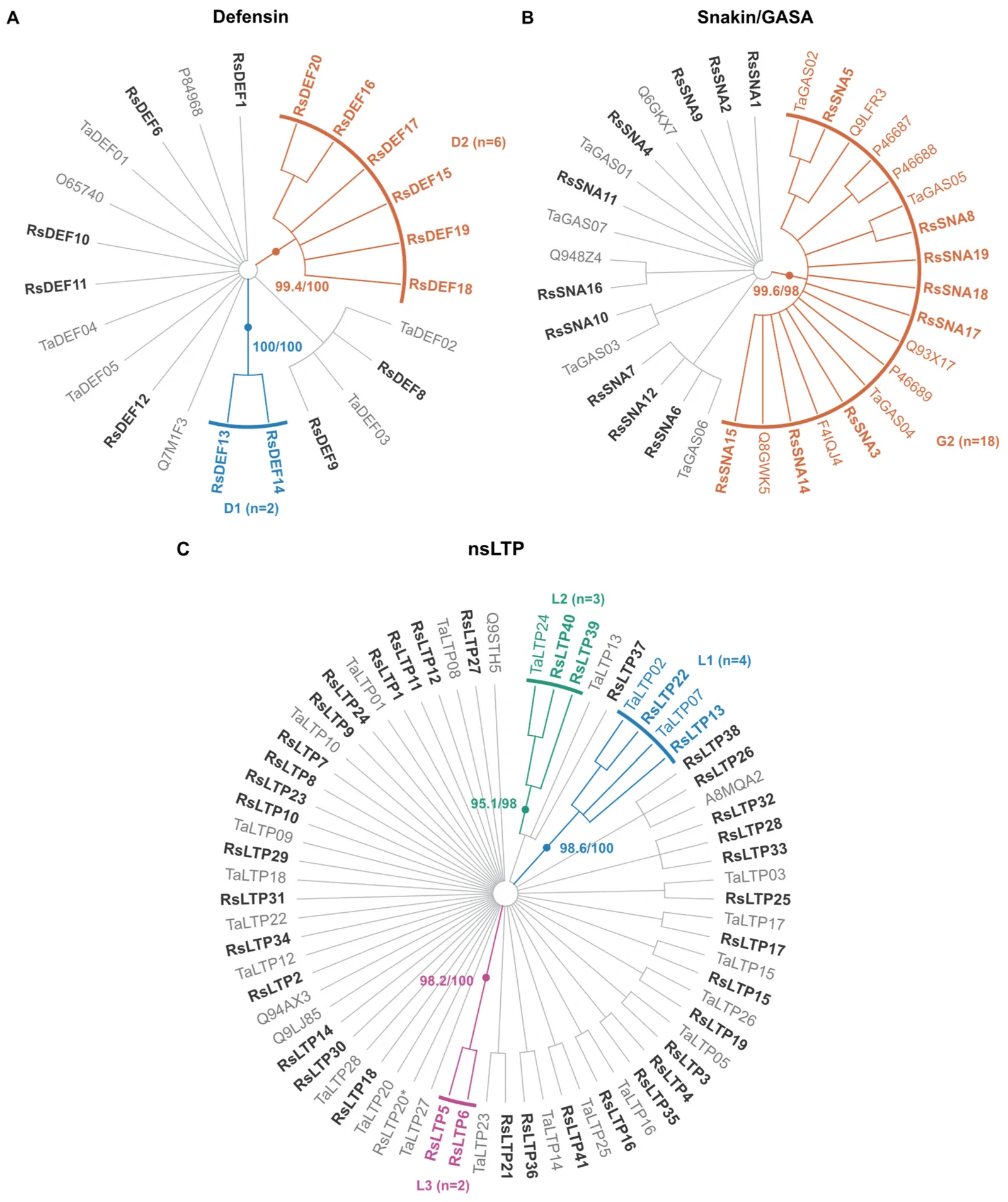
Phylogenetic relationships of AMP-family proteins. (**A**) Defensin, (**B**) Snakin/GASA and (**C**) nsLTP. The panels use the full-precursor gappyout maximum-likelihood trees and include all 73 primary *R. soongarica* members across the three families. Bold labels denote primary *R. soongarica* members; RsLTP20* is the extended-catalogue candidate. Coloured groups satisfy the same all-taxon unrooted split and SH-aLRT ≥ 80%/UFBoot ≥ 95% in all four alignment strategies; n is the number of all sequences in the highlighted group. Numerical labels report SH-aLRT/UFBoot in the display-source tree. Low-support internal branches are collapsed in main-display copies. These are supported local relationships, not formal subfamilies. D1, D2, G2 and L1–L3 are descriptive group labels. TaDEF, TaGAS and TaLTP labels denote *T. austromongolica* candidates; accession labels identify reference proteins. Radial lengths are not to scale, and the centre does not imply a biological root. Complete uncollapsed 110-, 43- and 159-tip trees are provided in Figure S1 and all branch values in Table S8.

**Figure 3:**
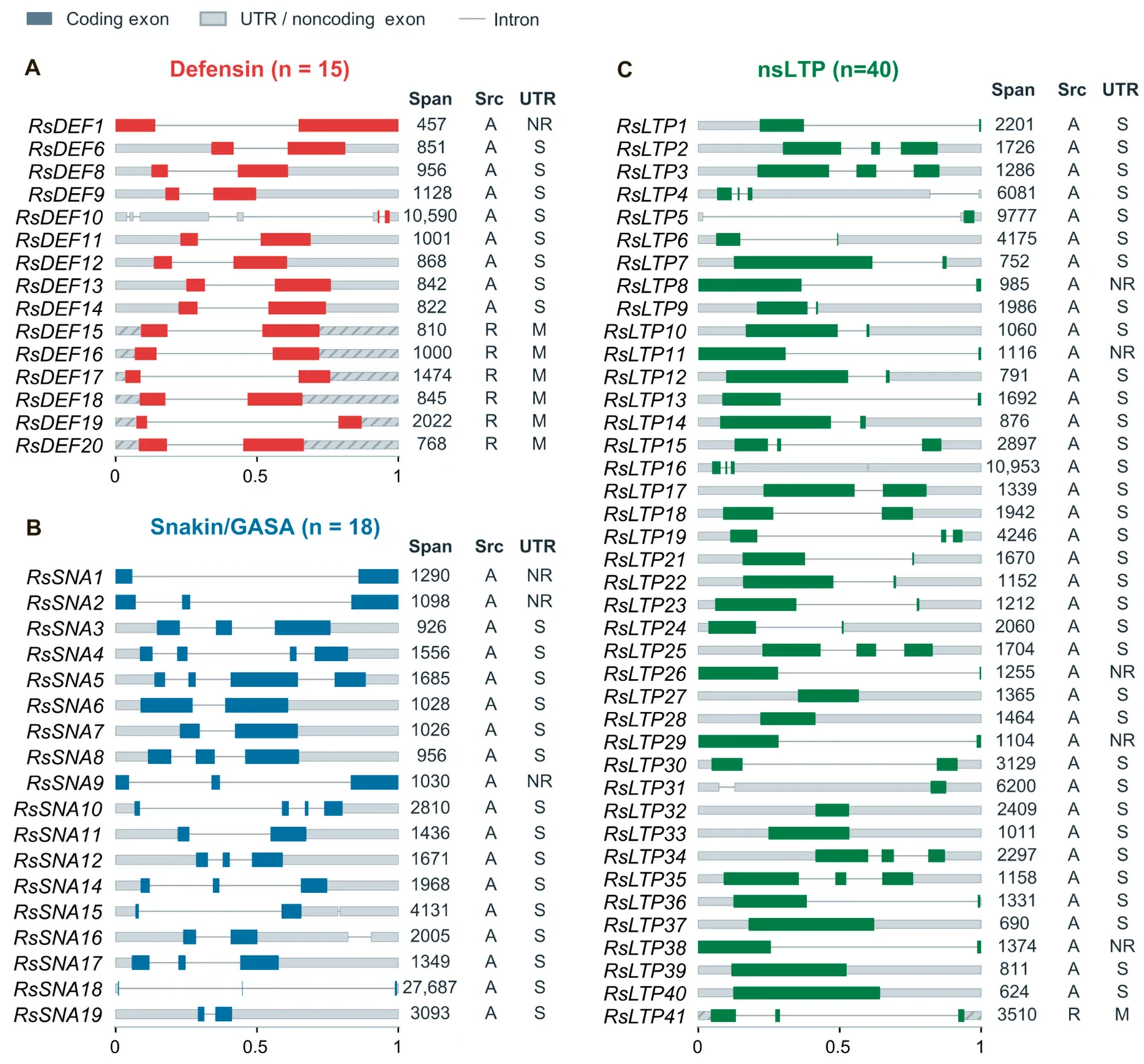
Gene structures of the 73 primary-catalogue AMP-family members. Models are shown in the 5′-to-3′ direction. Each model is independently normalized to 0–1 across its full genomic span, including introns; the adjacent span column reports that span in base pairs. Displayed widths therefore cannot be compared directly as absolute lengths across genes. Family-coloured blocks represent coding exon portions including terminal stop codons, thin lines indicate introns, and grey blocks indicate source-annotated untranslated regions (UTRs) or assembled noncoding exon portions. Grey and coloured portions may belong to the same exon. Source A denotes the reference annotation, and R denotes a reconstructed transcript model. UTR status: S denotes an explicit UTR feature in the source annotation, Diagonal hatching indicates model-derived noncoding exon portions in reconstructed transcript models (UTR status M); these regions are inferred from the assembled transcript models and do not represent independently annotated UTRs, and NR denotes no UTR feature recorded in the source. For reconstructed models, the noncoding portions are inferred from the assembled transcript model and do not establish an independently annotated UTR or transcription start site. NR does not establish biological absence of a UTR. *RsLTP20* is excluded from the primary-catalogue panel. (**A**), (**B**) and (**C**) show defensins, Snakin/GASA proteins and nsLTPs, respectively.

**Figure 4:**
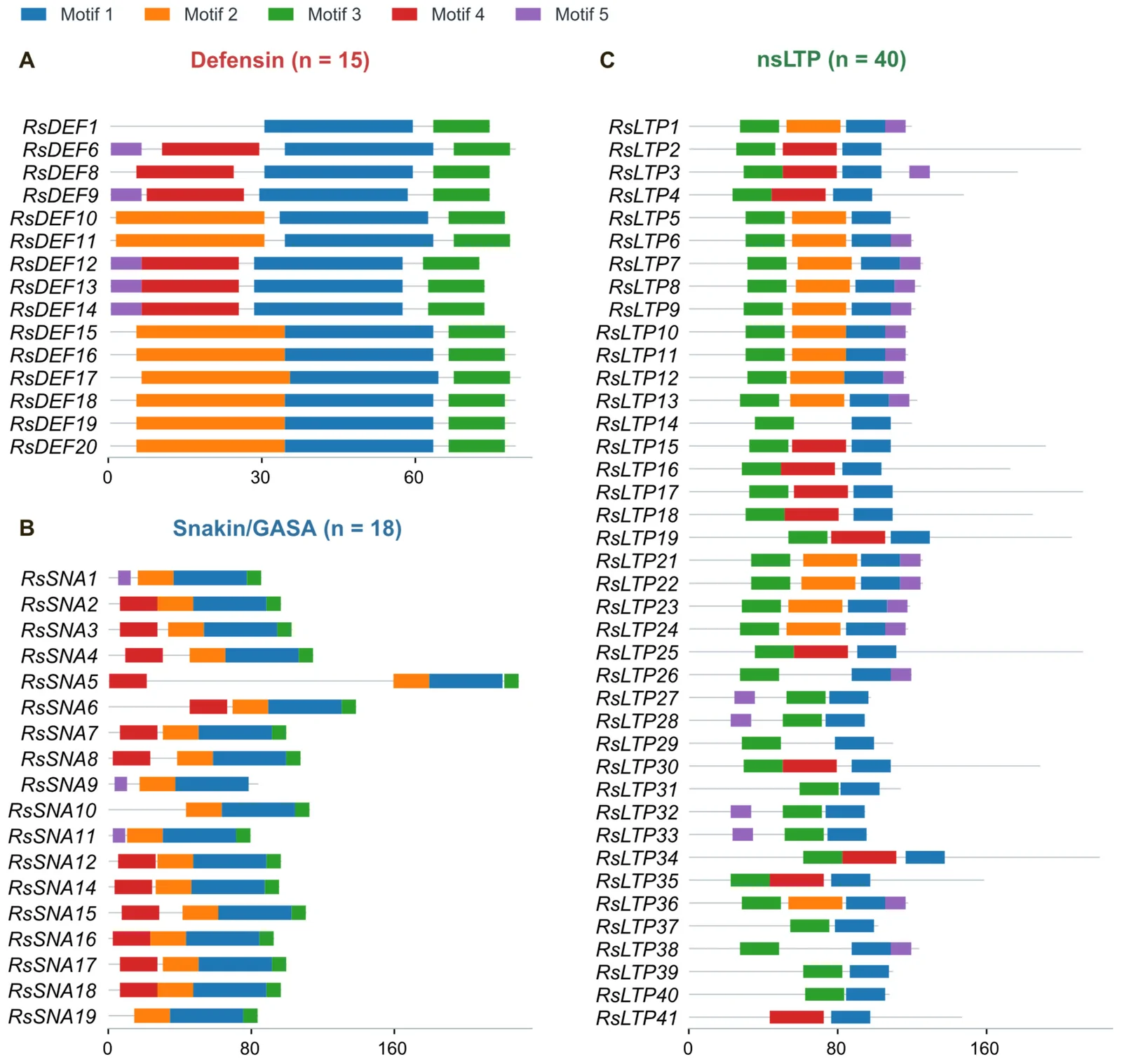
De novo protein-motif searches in the 73 primary-catalogue members. MEME searches were performed independently for (**A**) 15 defensins, (**B**) 18 Snakin/GASA proteins and (**C**) 40 nsLTPs using their complete precursor sequences. The five returned motifs per family are displayed at their amino acid residue positions along the full protein, at a *p*-value threshold of 10^−4^. Horizontal lines indicate protein lengths; coloured boxes indicate motif positions. Motif numbers and colours are ranks within each family and do not imply homology between families. The complete requested top-five output is shown, including the fifth defensin and fifth Snakin/GASA motifs with E-values of 3.0 and 27, respectively; these two motifs are exploratory and should not be described as statistically enriched. Motif definitions, E-values, contributing sites and scan sites are provided in Tables S44 and S45.

**Figure 5:**
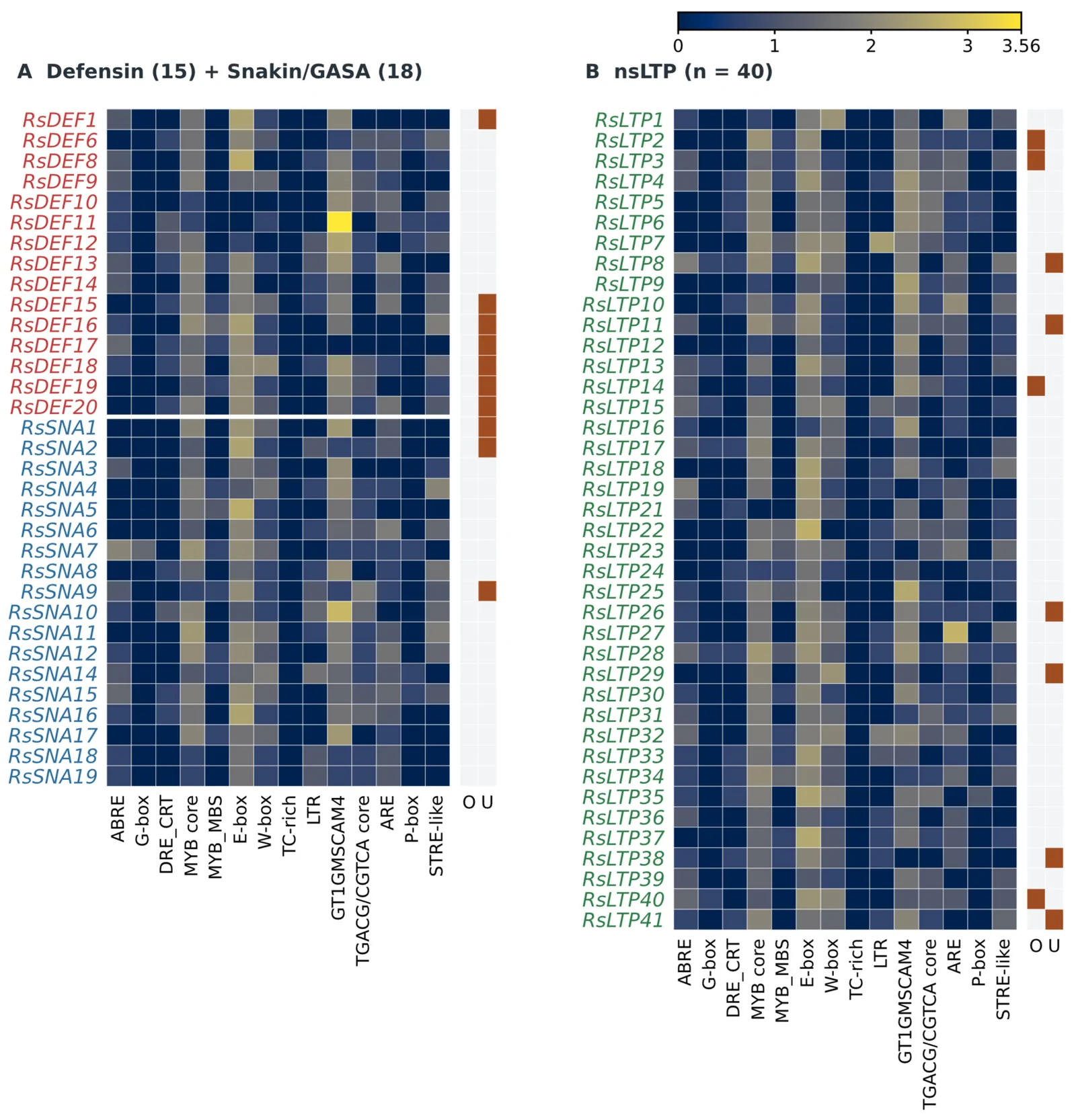
Sequence-pattern occurrences in 2 kb upstream proxies of the 73 primary *R. soongarica* AMP-family members (15 defensin, 18 Snakin/GASA and 40 nsLTP members). Proxies use the annotated 5′ gene boundaries of 66 members and the supported reconstructed-model boundaries of seven members; TSSs were not independently mapped. The panel contains 14 double-stranded IUPAC patterns and merges the reverse-complement-equivalent TGACG/CGTCA aliases; their sources and interpretation limits are listed in Table S27. Colour indicates ln(1 + nonredundant site count), with fixed family/member ordering and no row scaling or clustering. Gene-label font colors denote family membership: red, defensin; blue, Snakin/GASA; and green, nsLTP. Filled red-brown squares in column O indicate overlap with another official or reconstructed gene model, whereas those in column U indicate the lack of an annotated 5′UTR. Missing annotation does not establish biological absence of a UTR. No pattern was significantly enriched in the primary or either predefined sensitivity comparison (Table S29). Occurrence does not establish transcription-factor binding or regulatory activity. (**A**) Defensin and Snakin/GASA members; (**B**) nsLTP members. Both panels share the same colour scale. Columns O and U denote neighbouring-gene overlap and lack of an annotated 5′UTR, respectively.

The primary catalogue contains 73 *R. soongarica* candidates: 15 defensins, 18 Snakin/GASA genes and 40 nsLTPs (Tables S1 and S30). Of these, 66 are represented in the reference annotation, and seven are transcript-supported reconstructed models: *RsDEF15*–*RsDEF20* and the predicted GPI-anchored nsLTP (LTPg) candidate *RsLTP41*. Six additional genomic AMP-like fragments lacked supported complete gene models and were excluded (Section 3.3). *RsLTP20* is listed separately as a reference-dependent C4-like candidate, giving 74 entries when included (Table S31). Figure 1, Figure 2, Figure 3, Figure 4 and Figure 5 show the primary catalogue, with additional phylogenetic context sequences identified separately. RNA-seq quantification was available for the 66 reference-annotated members, of which 52 passed the count filter (Section 3.8). The genes included in the expression, collinearity and duplication analyses are listed in Table S34. Thionin, hevein-like and cyclotide screening did not yield candidates meeting the inclusion criteria. These families are therefore reported as screened but not detected, rather than as conclusively absent from the species.

Among the 73 primary members, WoLF PSORT assigned a unique highest score to extracellular localization for 54 proteins, chloroplast for 12, cytosol for two, vacuole for two and plasma membrane for one. *RsSNA10* and *RsLTP21* have tied highest-scoring categories and remain unresolved (Table S39). All seven proteins encoded by the reconstructed transcript models have predicted signal peptides; WoLF PSORT returns a unique highest extracellular score for five and a chloroplast score for *RsDEF18* and *RsDEF19*. *RsLTP2*, *RsLTP18* and *RsLTP30* have signal-peptide predictions but highest WoLF PSORT scores for chloroplast, plasma membrane and vacuole, respectively. These disagreements are retained without overriding either prediction or treating localization as proof of family identity.

The complete protein sequences ranged from 72 to 229 amino acids, with predicted molecular weights of 7.62–24.25 kDa and theoretical pI values of 4.05–10.50 (Tables S51 and S52). Defensin, Snakin/GASA and nsLTP precursors had median lengths of 78, 97.5 and 120.5 amino acids, respectively. All 18 Snakin/GASA sequences had a positive predicted net charge at pH 7.0, whereas both positive and negative values occurred among defensins and nsLTPs. Median GRAVY values were 0.368, −0.239 and 0.258 for the three families, respectively, describing variation in precursor composition.

### 3.2. nsLTP Family Assignments

Shared nsLTP curation yields 39 annotation-derived primary candidates in *R. soongarica* and 26 in *T. austromongolica*; the extended tiers contain 40 and 27. Including the reconstructed *R. soongarica* model *RsLTP41* gives 40 primary nsLTP candidates. *RsLTP17* and *RsLTP19* retain predicted LTPg annotations. *RsLTP20* lacks a specific nsLTP InterPro call but aligns to the Q6ZFW0 C4 core at 50.8 bits, 44.262% identity and complete coordinate coverage, with all eight cysteines corresponding. The strongest explicit alternative-family core scores 29.6 bits. The stronger full-sequence match to Q9LTK4 cannot establish a 2S identity because this reference has an unresolved broad-prolamin assignment. Both short-core trees have mixed reference neighbourhoods. *RsLTP20* is therefore reported as a reference-dependent C4-like candidate, not a resolved nsLTP candidate or a confirmed 2S protein. The profile-dependent members *RsLTP29* and Tamarix evm.model.Chr05.234 retain primary membership because their specific subgroup matches coincide with the complete core. Their membership depends on this single profile and is identified accordingly in Table S35. Profile-coordinate comparisons also identified approximate profile endpoints in *RsLTP28*, *RsLTP32*, *RsLTP33* and Tamarix novel_model_206_643946eb: their sequence cores retain all eight cysteines, but the profile spans end 2–3 residues before C8. The three *R. soongarica* members have additional qualifying reviewed-core matches; the Tamarix member does not and retains a profile-edge uncertainty flag. The 40 primary nsLTP candidates retain a compatible eight-cysteine core, while 13 have positive GPI-anchor predictions, including *RsLTP41* (Table S56). The table reports core-cysteine spacing and secretion architecture without imposing a complete type 1/type 2 classification.

### 3.3. Six Genomic AMP-like Fragments Lack Supported Complete Gene Models

The six tblastn-derived genomic sequences were retained as genomic AMP-like fragment candidates outside the primary catalogue. Each peptide fragment was exactly encoded in the genome, but the local uninterrupted reading frame examined lacked an upstream in-frame methionine that completed the proposed model; this check does not exclude an unmodeled spliced transcript. None of the six target coding intervals had aligned short-read coverage in the nine tested public libraries, and the available long-read analysis did not reconstruct a complete start-to-stop ORF containing any of these fragments. *RsDEF7* had one long read overlapping 19 of 258 coding bases (7.36%), providing insufficient support for a complete model rather than complete absence of local overlap (Table S5). The tblastn screen using 128 reference sequences (63 defensin, five thionin and 60 Snakin/GASA references) yielded 1003 HSPs and 37 clustered intervals: 31 overlapped existing gene spans, and the six non-overlapping intervals corresponded to these six fragment candidates (Table S6). Gene-span overlap does not establish a same-strand complete coding model; two intervals, GWRS014 and GWRS017, overlapped only opposite-strand gene spans and remain explicitly qualified. The seven supported reconstructed transcript models in the primary catalogue do not overlap these six fragments. These results do not establish exhaustive family recovery or determine why each fragment was absent from the reference annotation.

### 3.4. Chromosomal Distribution

All 73 primary members map to the 11 chromosomes (Figure 1). Counts on Chr01–Chr11 are 16, 4, 2, 11, 2, 6, 9, 7, 9, 1 and 6, respectively. The mapped members include all seven reconstructed models. Coordinates and source identifiers are provided in Table S1.

Among the 66 members represented in the duplication-classification reference, 19 were assigned to tandem duplication, two to proximal duplication, 14 to the whole-genome/segmental category, 23 to dispersed duplication and eight to singleton status (Tables S53 and S54). The remaining seven primary members were not assessed because their reconstructed models were absent from that reference. Within-species collinearity supported seven candidate pairs across five blocks: five nsLTP pairs and two Snakin/GASA pairs (Table S55). These positional relationships complement the member-level duplication classes and provide genomic context for the selected Ka/Ks comparisons.

### 3.5. Phylogenetic Relationships and Supported Groups

Maximum-likelihood trees were only partly resolved (Figure 2; Table S8). Six non-overlapping local groups met the joint SH-aLRT ≥ 80% and UFBoot ≥ 95% criterion in all four sequence and alignment strategies: two defensin, one Snakin/GASA and three nsLTP groups. Defensin group D1 contained *RsDEF13* and *RsDEF14* (two total sequences; full-precursor gappyout SH-aLRT/UFBoot 100/100), and D2 contained *RsDEF15*–*RsDEF20* (six sequences; 99.4/100). Snakin/GASA group G2 contained *RsSNA3*, *RsSNA5*, *RsSNA8*, *RsSNA14*, *RsSNA15*, *RsSNA17*, *RsSNA18,* and *RsSNA19* (18 total sequences, including references; 99.6/98). nsLTP groups L1, L2 and L3 contained *RsLTP13*/*RsLTP22* (four total sequences; 98.6/100), *RsLTP39*/*RsLTP40* (three; 95.1/98) and *RsLTP5*/*RsLTP6* (two; 98.2/100), respectively.

Other relationships were sensitive to sequence selection or alignment processing. For example, one Snakin/GASA split had UFBoot values of 94% and 92% in the untrimmed-core and gappyout-core trees, respectively, despite identical 43-sequence sampling. The *RsLTP26*/*RsLTP38* split met the support criterion in three strategies but fell below both thresholds in the full-precursor gappyout tree (77.7%/90%). These groups describe supported local relationships rather than a complete subfamily classification.

The additional outgroup tests did not establish a consistent family root across the evaluated strategies (File S2). All eight nsLTP analyses recovered a supported separation of the 2S candidates (SH-aLRT 96.7-100; UFBoot 100), but the fixed 108-sequence ingroup yielded six distinct basal partitions. The 10 gymnosperm defensin candidates were separated as a complete set only in the full-length, untrimmed analysis (96.7/98); the other three strategies did not recover that split. The three Selaginella GASA candidates were not separated as a complete set in any of the four analyses. Nine conditional trees were obtained, eight for nsLTP and one for defensin. These are sensitivity results rather than adopted biological roots. These 16 outgroup tests used the taxon sets specified in Section 2.5, which differ from the 12 family trees in Figure S1; their support values therefore apply only to the corresponding test trees.

### 3.6. Gene Structure and Conserved Motifs

Gene structure and conserved motifs were examined for all 73 primary members (Figure 3 and Figure 4). All 15 defensins have two coding segments. The 18 Snakin/GASA models have two (seven members), three (8) or four (3) coding segments; the 40 nsLTP models have one (9), two (21) or three (10). Coding-segment counts are distinguished from total exon counts. Source UTR features are recorded for 57 members, absent from the source annotation for nine, and replaced by explicitly inferred noncoding exon portions for seven reconstructed transcript models. The MEME output contains five requested motifs per family and 246 scan sites. Defensin motifs 1 and 3 occur in all 15 members, Snakin/GASA motifs 1 and 2 in all 18, and nsLTP motif 1 in all 40. The fifth defensin and fifth Snakin/GASA motifs have E-values of 3.0 and 27 and are exploratory.

### 3.7. Ka/Ks Estimates and Promoter Sequence Patterns

Of the 23 duplicate pairs involving 33 reference-annotated catalogue members, 19 had usable Ka/Ks estimates, of which 16 have Ka/Ks < 1 and nominal Fisher *p* < 0.05, consistent with purifying selection under the stated short-peptide limitations. These estimates describe the selected pairs, rather than all possible duplications among the 73 members.

All 73 primary members yielded complete, unambiguous 2 kb upstream proxies. MYB-core, W-box, ABRE and DRE patterns occurred in 72, 66, 48 and 27 of the 73 upstream intervals, respectively; the TC-rich pattern was not detected. These frequencies describe sequence occurrence, independently of enrichment relative to the genomic background (Tables S9 and S10). No tested pattern was significantly enriched in the primary comparison (73 targets versus 21,695 background intervals; 14 tests; minimum BH-adjusted *p* = 0.913). The same conclusion held after symmetric sequence-quality and neighbouring-gene-overlap filtering (69 versus 19,957; minimum adjusted *p* = 0.594) and after additionally requiring annotated 5′UTRs in both cohorts (53 versus 19,144; minimum adjusted *p* = 0.719; Table S29). Joint correction over all 42 comparisons gave adjusted *p* = 1.0 for every test. Four target intervals overlapped neighbouring-gene models, and 16 targets lacked a linked 5′UTR annotation, including all seven reconstructed transcript models (Table S28). Figure 5 displays sequence occurrences without implying regulatory activation.

### 3.8. RNA-Seq Reanalysis Identifies Expression Changes Under Na_2_SO_4_ Treatment

**Figure 6:**
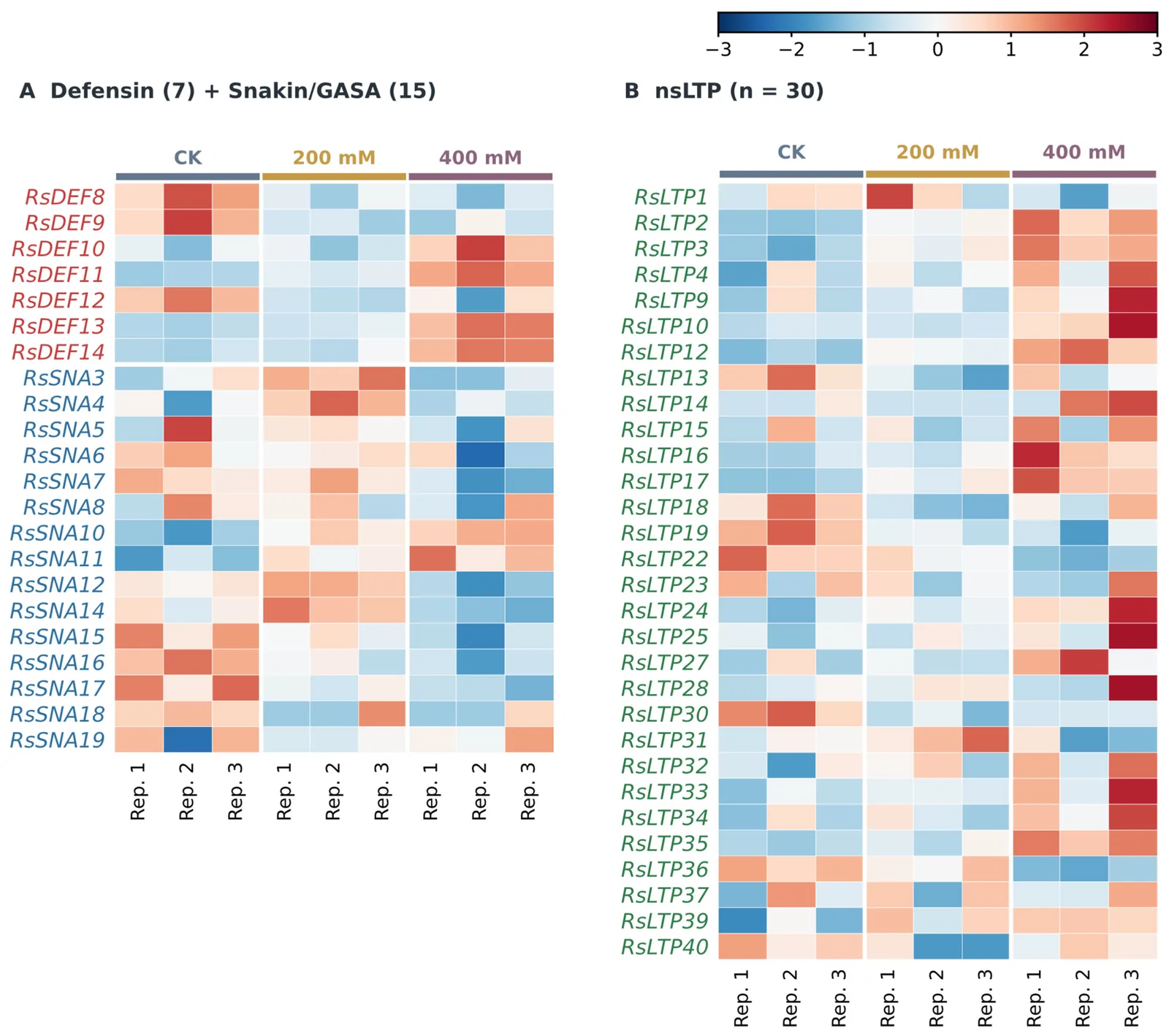
Expression profiles of 52 primary-catalogue AMP members passing the RNA-seq count filter. Columns show three biological replicates each of CK, 200 mM Na_2_SO_4_ and 400 mM Na_2_SO_4_. Colours represent row-wise z-scores of variance-stabilized expression values from the whole-transcriptome DESeq2 analysis; the symmetric colour scale spans −3 to 3 and no values are clipped. Label colours identify defensin, Snakin/GASA and nsLTP families. Rows follow catalogue order within families, without clustering. Of the 73 primary members, 66 were present in the annotation-based transcript reference; 11 had all-zero rounded counts, three had total rounded counts below 10, and 52 were retained. The seven new models absent from the reference are not plotted, and no missing values were imputed. Full expression data and testing status are provided in Tables S12, S13 and S26. (**A**) Seven defensins and 15 Snakin/GASA members; (**B**) 30 nsLTPs. Both panels share the same z-score scale and library order.

Of the 73 primary AMP candidates, 66 were represented in the annotation-based Salmon reference, and 52 passed the total-count filter, comprising seven defensins, 15 Snakin/GASA members and 30 nsLTPs. Eleven reference-covered members had zero rounded counts in all nine libraries, and three had non-zero totals below 10. The seven reconstructed models were absent from the quantification reference. Figure 6 therefore displays 52 members, whereas the supplementary expression tables retain all 73 members and their distinct quantification or testing states.

**Figure 7:**
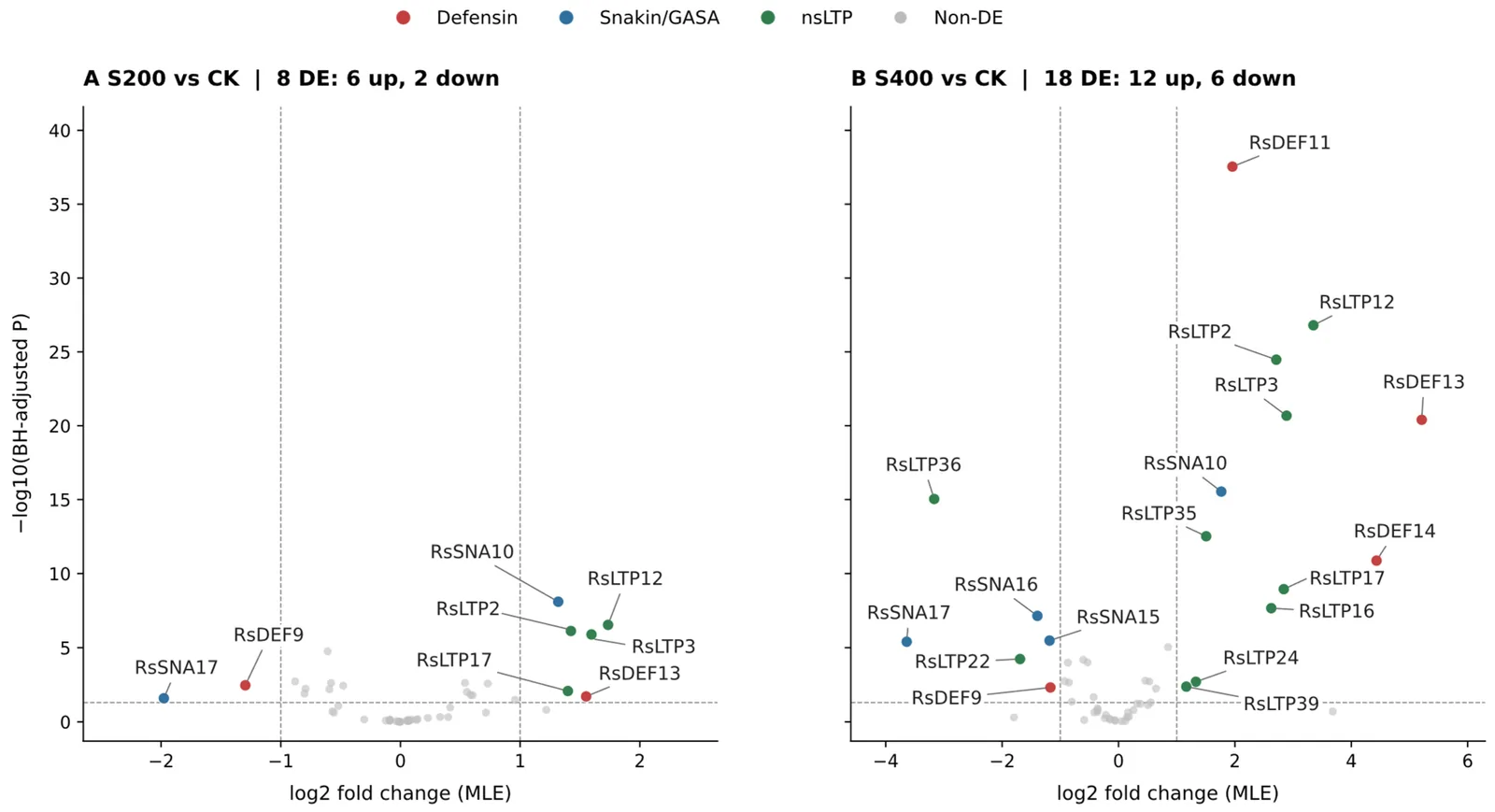
Differential expression of primary-catalogue AMP members represented in the annotation-based RNA-seq reference. Panels show (**A**) S200 versus CK and (**B**) S400 versus CK. The x-axis is the unshrunken maximum-likelihood log_2_ fold change; positive and negative values indicate upregulation and downregulation, respectively. The y-axis is −log10 of the Benjamini–Hochberg-adjusted *p*-value from the whole-transcriptome DESeq2 analysis. Dashed lines mark adjusted *p* = 0.05 and log_2_ fold changes of −1 and 1. Coloured points satisfy BH-adjusted *p* (FDR) < 0.05 and |log_2_ fold change| > 1, with colours identifying the three families; grey points represent genes not classified as differentially expressed (Non-DE) under these joint criteria. All eight differentially expressed members in panel A (six upregulated, two downregulated) and all 18 in panel B (12 upregulated, six downregulated) are labelled with individual leader lines. The panels contain 49 and 52 members with finite plotting statistics, respectively, out of 52 count-filtered members in the 66-member reference-covered cohort. Three additional members in panel A have unavailable adjusted *p*-values after independent filtering. Members excluded by the count filter and the seven models absent from the reference are not represented as grey points. Their distinct states are retained in Tables S14 and S26.

Differential-expression analysis identified eight differentially expressed members in S200 versus CK (six upregulated and two downregulated), and 18 in S400 versus CK (12 upregulated and six downregulated; Figure 7). The S400-versus-S200 contrast identified 13 differentially expressed members (nine upregulated and four downregulated; Table S15). *RsLTP2* was upregulated in all three contrasts: its log_2_ fold changes were 1.424 in S200 versus CK, 2.711 in S400 versus CK and 1.286 in S400 versus S200, with adjusted *p*-values of 7.48 × 10^−7^, 3.38 × 10^−25^ and 8.29 × 10^−6^, respectively. *RsLTP18* and *RsLTP30* passed the count filter but did not exceed the absolute log_2_ fold-change threshold in any contrast. Their available expression evidence therefore does not satisfy the specified two-part differential-expression criterion. At S400 versus CK, the DE set comprises four defensins (three up/one down), four Snakin/GASA genes (one up/three down) and ten nsLTPs (eight up/two down). *RsLTP20* has one total count and *RsLTP29* has all-zero counts; neither adds a DE call in the family-assignment sensitivity analyses. *RsSNA17* illustrates the repressed response: its maximum-likelihood log_2_ fold changes were −1.98 at S200 versus CK (adjusted *p* = 0.0257) and −3.64 at S400 versus CK (adjusted *p* = 3.97 × 10^−6^; Table S14).

### 3.9. RsLTP12 Expression Under Na_2_SO_4_ Treatment

In the analyzed leaf RNA-seq dataset, *RsLTP12* was strongly induced in S400 versus CK (baseMean = 292.45; maximum-likelihood log_2_ fold change = 3.35; shrunken log_2_ fold change = 3.30; adjusted *p* = 1.58 × 10^−27^). Its transcript had low mappability risk: the best non-self transcript match was 76.26% identical over 51.24% of the query transcript, with no near-identical transcript hit or Salmon ambiguity. *RsDEF11*, *RsDEF13*, *RsSNA10*, *RsLTP2*, *RsLTP3*, *RsLTP16*, *RsLTP17*, *RsLTP35*, and *RsLTP36* also contributed to the high-dose–response pattern (Figure 7 and Supplementary Tables). These genes are prioritized for further investigation based on expression changes in this dataset. An eight-member list spanning the three families is provided for future expression validation (Table S22).

### 3.10. Comparative Analysis with Tamarix and Arabidopsis

**Figure 8:**
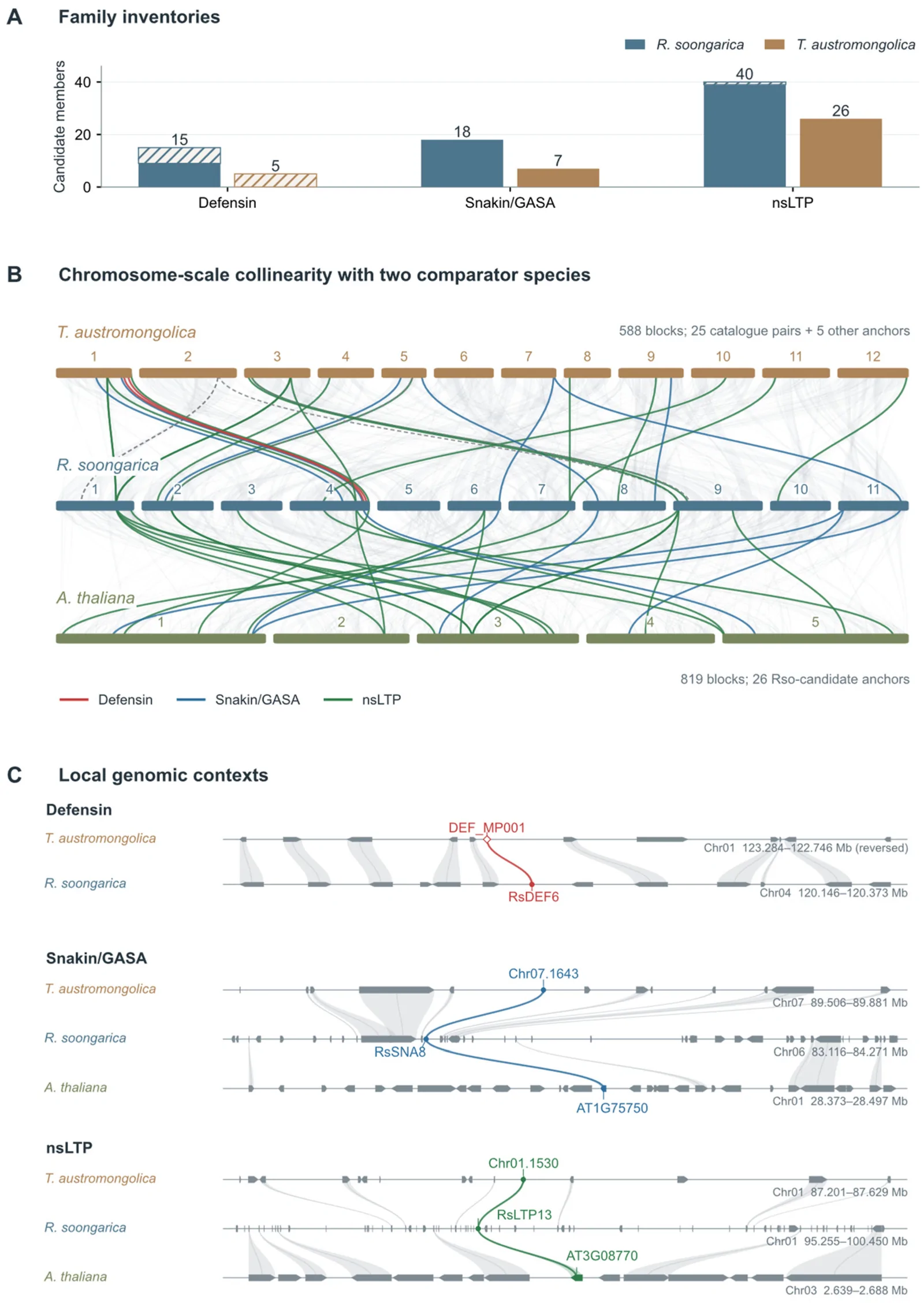
Family inventories and genomic collinearity of *R. soongarica* with *T. austromongolica* and *A. thaliana*. (**A**) Curated family counts in *R. soongarica* and *T. austromongolica*: 15/18/40 versus 5/7/26 defensin, Snakin/GASA and nsLTP candidates. Diagonal hatching in panel (A) denotes reconstructed or homology-derived gene models. Arabidopsis is included as a collinearity comparator, without a matched family inventory. (**B**) The Tamarix comparison contains 588 background blocks, 25 pairs with primary candidates at both ends (coloured) and five other candidate-involving anchors (grey dashed). The Arabidopsis comparison contains 819 background blocks and 26 anchors involving primary *R. soongarica* candidates; colours follow the *R. soongarica* family assignment. Chromosome lengths are scaled independently within each genome.Grey solid lines in panel (B) represent background genome-wide collinearity links, whereas colored links indicate anchors involving primary *R. soongarica* candidates. Grey dashed links denote the five additional Tamarix candidate-involving anchors with only one primary-candidate endpoint. (**C**) Local neighbourhoods of *RsDEF6* with Tamarix, and of *RsSNA8* and *RsLTP13* with both comparators. Arrows indicate gene position and orientation; highlighted links connect the focal genes. Local tracks are scaled independently, with reversed coordinates labelled. Open diamonds identify additional homology-derived models. Seven reconstructed *R. soongarica* models are absent from both anchor references. Complete pair records and local neighbourhoods are supplied in Tables S21 and S57–S59 and Files S8 and S11.

The primary resources contain 15/18/40 defensin, Snakin/GASA and nsLTP candidates in *R. soongarica* and 5/7/26 in *T. austromongolica* (73 versus 38; Figure 8A; Table S19). Six *R. soongarica* defensins and one nsLTP are reconstructed models; all five Tamarix defensins are additional homology models, with an official annotation count of zero. The extended resource adds one C4-like candidate per species, giving 74/39 total entries and 41/27 nsLTPs. Removing these two reference-dependent entries leaves the nsLTP count difference unchanged. The effects of dependence on a single profile and uncertain profile boundaries are reported in Table S35 and File S6. Candidate counts are sensitive to annotation and discovery scope and do not establish lineage-specific expansion.

Sequence comparison yielded 27 unique primary-catalogue reciprocal-best-hit pairs: five defensin, five Snakin/GASA, and 17 nsLTP pairs (Table S20); the extended resource yielded 28. *RsLTP41* forms a qualifying reciprocal pair with Tamarix evm.model.Chr02.1305 (75.904% identity; E = 1.18 × 10^−42^; coordinate coverage 56.85%/69.17%). These pairs indicate candidate homology, not proven one-to-one orthology. The 588 interspecies MCScanX blocks yielded 30 primary-candidate-involving anchors, including 25 with primary candidates at both ends (two defensin, seven Snakin/GASA and 16 nsLTP pairs) and five with only one primary endpoint. The 25 direct pairs involve 22 distinct *R. soongarica* and 23 distinct Tamarix genes. The seven new *R. soongarica* models were absent from that genomic-anchor reference; their absence from these links is not evidence of absent homology. File S8 supplies all 30 Tamarix candidate-involving contexts. Separate *T. chinensis* annotation-context and supported candidate-pair expression results are reported in Section 3.12 and Section 3.13.

The Arabidopsis comparison contained 819 interspecies collinearity blocks. Of the 66 reference-annotated *R. soongarica* primary candidates eligible for this analysis, 22 members (17 nsLTP and five Snakin/GASA) contributed 26 anchors to 22 Arabidopsis loci: 20 nsLTP-associated and six Snakin/GASA-associated pairs (Figure 8B; Table S57). No anchor was recovered for the nine reference-annotated defensins. The separate extended candidate *RsLTP20* contributed one additional pair and was excluded from these primary counts. Thirteen *R. soongarica* members had candidate-associated collinearity links to both comparator species (Table S59). Figure 8C illustrates these relationships for *RsSNA8* and *RsLTP13*, together with the *R. soongarica*–Tamarix defensin example. The complete Arabidopsis anchor neighbourhoods are provided in File S11. These links describe genomic correspondence and do not establish one-to-one orthology, equivalent family assignments, or conserved biological activity.

### 3.11. Unassigned Reads and Additional Transcript-Supported Gene Models

Across the nine libraries, 5,863,532 fragments unassigned to annotated transcripts matched genome decoys; 149 were classified as low quality or low complexity, 104,313 as rRNA-derived and 15,094 as plastid-derived. None matched the mitochondrial sequence proxy. The remaining categories comprised 26,234,614 genome-mapped but transcriptome-unassigned fragments and 9,552,732 genome-unmapped residual fragments. The targeted residual-read screen identified 23,126 read-level matches passing the AMP-reference screening criteria, but no novel AMP genes meeting the complete-model and family-evidence criteria; read-level similarity was not treated as a complete ORF or gene model. Genome-mapped, transcriptome-unassigned reads yielded 107 ORF records: 61 at known loci, 32 overlapping or altered models and 14 intergenic records. Seven complete models met the family and transcript-support criteria and were named *RsLTP41* and *RsDEF15*–*RsDEF20* (Table S33). Four other ORF records at three loci remain incomplete models. Further homology searches yielded six additional clusters listed in the supplementary results, including four with specific nsLTP-domain support but without equivalent per-locus transcript/model validation (Table S33; Files S4 and S6).

The seven supported models arose outside the reference annotation and the six tblastn-derived fragment intervals (File S4). *RsDEF15*–*RsDEF20* met the defensin-family criteria, and *RsLTP41* met the criteria for a predicted GPI-anchored nsLTP-type member. The six defensin models passed the PF00304 gathering threshold and had complete-CDS transcript models in eight or nine libraries, together with exact-junction and coding-region support. *RsLTP41* combined an nsLTP-specific IPR000528 annotation with a compatible cysteine framework, signal-peptide and GPI predictions, complete-CDS models in all nine libraries, and short- and local long-read support. CAND01 remained a transcriptionally supported defensin-like candidate with unresolved family assignment; CAND02 had nsLTP-family support but an uncertain precursor initiation boundary. CAND08 connects coding exons shared with *RsDEF19* and *RsDEF18*. Its exact connecting junction was supported by 18 qualifying fragments across seven libraries, but no qualifying long read supported the complete local coding structure. It was retained as a connecting model without counting an additional independent locus. The ten models thus comprise seven primary-catalogue members, two unresolved candidates and one connecting model. Additional assembly and homology-search candidates are documented in File S4. The seven supported models are included in the structural, motif, phylogenetic and protein-sequence comparisons. They are absent from the Salmon and genomic-collinearity references, so their transcriptional support does not establish differential expression under treatment.

### 3.12. A Public T. chinensis Dataset Adds External Expression Context

All six *T. chinensis* libraries were quantified against the exon-defined transcript reference. Assignment to annotated transcripts ranged from 58.77% to 63.91% of processed read pairs; these values are not whole-genome alignment rates. The analysis included 26,426 reference gene loci, of which 19,214 passed the count prefilter. The fixed 41-entry annotation-context list included all selected genes regardless of significance or direction in this analysis (Figure S2; Tables S40–S43; File S7).

Within the 33 entries carrying source lipid-transfer-related annotations, four met the operational upregulation criterion, and four met the downregulation criterion; the corresponding counts among the eight GASA/gibberellin-regulated entries were one and four. These counts describe only the selected context list. They are not estimates of complete family responses and cannot be compared as response proportions with the *R. soongarica* catalogue. No defensin absence or non-response is inferred from the absence of matching annotation wording in the source DEG tables. This literature-selected display supplies expression background from another halophyte. The homologous-pair comparison is reported separately in Section 3.13.

### 3.13. Expression Responses of Homologous Candidates in R. soongarica and T. chinensis

The 63-candidate query subset recovered matches for 49 members to 24 *T. chinensis* genes. Applying the reciprocal-score and forward-HSP coverage rules retained 20 reciprocal pairs: 15 nsLTP and five Snakin/GASA pairs. Sixteen were also supported by collinearity and formed the main expression set (11 nsLTP and five Snakin/GASA pairs). The corresponding whole-genome output contained 906 MCScanX blocks, including 566 interspecies blocks and 26 anchors involving an *R. soongarica* query candidate. These counts describe this *T. chinensis* analysis and are separate from the *T. austromongolica* results in Section 3.10. Query-set membership, excluded catalogue members, pair assignments and coverage subsets are supplied in Tables S47–S50 and File S9.

For each *R. soongarica* contrast, 12 of the 16 supported pairs had usable effect estimates and adjusted *p*-values in both studies; four were unavailable for this comparison. Effects had the same sign in 6/12 pairs for S200 versus CK and 9/12 for S400 versus CK (Figure S3). Three pairs met the DE criterion in both studies at S200 (*RsLTP3*–TC01G0837, *RsLTP17*–TC12G1360 and *RsSNA10*–TC12G1896), and four did so at S400 (the same three plus *RsLTP16*–TC12G1359); all were upregulated in both studies. These observations comprise seven comparison rows but four unique pairs and four *T. chinensis* genes. They describe a conditional subset, not uniform concordance across all supported pairs.

The additional reverse-HSP coverage requirement retained 18 reciprocal pairs, of which 14 had collinearity support. It excluded *RsLTP17* and *RsLTP34* from the main supported set. In this stricter set, 10 pairs per contrast had usable estimates and adjusted *p*-values in both studies; five comparison rows involving three unique pairs (*RsLTP3*, *RsLTP16* and *RsSNA10*) met the DE criterion in both studies and remained concordantly upregulated. Thus, the highlighted *RsLTP17* pairing is coverage-sensitive. The two downregulated pairs in the less restrictive output were not included in the main set: *RsSNA15* lacked reciprocal and collinearity support, while *RsSNA16* had only 43.66% target coverage in its forward HSP and lacked collinearity support. These results provide limited candidate-level parallels under different experimental conditions, not proof of conserved function or a species effect.

## 4. Discussion

The *R. soongarica* catalogue comprises 73 candidates, including 40 nsLTPs, 18 Snakin/GASA members and 15 defensins. The predominance of nsLTP candidates provides a basis for investigating the diversity of lipid-transfer-related proteins in this desert shrub. Transcript evidence also supported seven previously unannotated models, comprising six defensins and one predicted GPI-anchored nsLTP. These models extend the sequence resources available for studying small cysteine-rich proteins in *R. soongarica*. Their recovery is consistent with the underannotation of small-peptide genes reported in other plants [6,47]. The contribution is therefore both an improved family catalogue and the identification of transcribed candidate loci that would be overlooked by an analysis restricted to the existing protein annotation (Section 3.11).

Family boundaries also affect how this diversity is described. *RsLTP20* shows sequence similarity to the rice protein OsC4 (Q6ZFW0), but its family assignment remains uncertain and depends on that reference. It is retained as a supplementary candidate outside the primary count of 73. Combining family-specific and structural evidence follows the approach of previous nsLTP surveys [11,12], while retaining uncertain candidates makes them available for further characterization. The occurrence of tomato nsLTPs that do not fit established type classifications [32] further illustrates why a useful family catalogue need not imply a complete classification of every member.

The expression analysis identifies members associated with the leaf response to Na_2_SO_4_. Relative to CK, the number of differentially expressed members increased from eight at 200 mM to 18 at 400 mM, and 13 differed between the two treatment concentrations. At 400 mM relative to CK, eight of ten differentially expressed nsLTPs and three of four defensins were induced, whereas three of four differentially expressed Snakin/GASA members were repressed (Figure 7). These observations reveal a broader transcriptional response at the higher tested concentration, with individual members responding in different directions. They provide a basis for examining why related peptide genes differ in their regulation under the same treatment.

The known biological roles of these families suggest specific questions arising from these patterns. nsLTPs participate in extracellular lipid transport and surface-lipid deposition in other plants [4,7,8]. Their induction in *R. soongarica* raises the possibility that particular members contribute to lipid-associated processes during leaf exposure to salinity. Defensin responses broaden the set of candidates for investigating links between cysteine-rich peptides and stress physiology [9]. For Snakin/GASA genes, the mixture of induction and repression can be examined in relation to their reported connections with growth, hormone signalling and redox homeostasis [10]. These are hypotheses about individual candidates, rather than demonstrated functional differences between the three families. Including both induced and repressed members would help distinguish general treatment responses from responses associated with particular peptide genes.

The results also narrow the choice of candidates for such studies. *RsLTP12* showed strong induction at 400 mM Na_2_SO_4_, with a shrunken log_2_ fold change of 3.30, adequate transcript abundance and low ambiguity in transcript assignment (Section 3.9). *RsLTP2* was induced in all three contrasts, providing a candidate for investigating expression changes across the tested concentrations. These genes address complementary questions about a strong high-concentration response and variation along the treatment gradient. The use of expression context to select members from large families has precedents in anther-enriched wheat nsLTPs [48] and cacao GASA responses to pathogen inoculation [49]. Here, the catalogue and expression profiles identify specific starting points for linking peptide-gene variation to leaf stress responses. *RsSNA17*, repressed relative to CK at both treatment concentrations, adds a contrasting candidate for investigating why individual family members respond in opposite directions.

The sequence and genomic comparisons place these candidates in an evolutionary context. Six local phylogenetic groups were supported under all four sequence and alignment strategies, providing sets of related members for comparing sequence structure and expression without assigning unresolved branches to formal subfamilies. Among the 19 usable Ka/Ks estimates, 18 were below one, and 16 of these also had nominal Fisher *p* < 0.05, consistent with purifying selection under the stated short-peptide limitations (Section 3.7). Comparison with *T. austromongolica* identified 27 reciprocal-best-hit pairs and 25 direct candidate pairs supported by collinearity. These relationships provide positional and sequence-based connections through which variation among related genes can be examined. The higher candidate counts in *R. soongarica* cannot by themselves establish lineage-specific expansion because the two catalogues differ in annotation and gene-discovery support. The presence of tandem, dispersed, and collinearity-supported duplicates provides complementary groups for examining whether sequence-related members retain similar expression or differ in their regulation (Tables S53–S55). The retained Arabidopsis comparison extends this context to a more distant plant reference: 22 *R. soongarica* members contributed 26 collinear anchors, and 13 members had links to both comparator species. Such positional correspondence provides candidates for studying the persistence and diversification of genomic neighbourhoods. The lack of recovered Arabidopsis anchors for the nine testable defensins cannot establish gene loss, and the Arabidopsis partners have not undergone the matched family curation used for Tamarix.

The external *T. chinensis* dataset adds expression context to these comparisons. Four supported homologous pairs were upregulated in both studies in at least one *R. soongarica* contrast; three remained under the stricter reciprocal-coverage requirement (Section 3.13). Thus, the available data identify a small set of homologous candidates whose salt-associated induction recurs across the two studies. Such pairs are useful for testing whether related genes share aspects of transcriptional regulation under salinity. The mixed directions among other evaluable pairs also indicate that this pattern is restricted to particular members. Differences in species, sampled organs and salt treatments mean that these parallels should guide candidate selection rather than be interpreted as conservation of an antimicrobial or salt-tolerance mechanism.

The promoter survey provides a sequence-level starting point for investigating this differential regulation. None of the tested patterns was significantly enriched after multiple-testing correction, including after the alternative upstream-region filters were applied (Section 3.7). Thus, the observed expression responses were not accompanied by detectable enrichment of these patterns at the catalogue level. Further questions concern features not resolved by motif counts, including the activity of individual sites, their arrangement and the relevant transcription factors. Because the analyzed regions are annotation-based upstream proxies, the contribution of specific sequences would need to be examined in relation to experimentally defined promoter boundaries and regulatory activity.

The expression results derive from one public leaf RNA-seq experiment with three biological replicates per treatment and lack independent qRT-PCR validation. They establish expression associations under the sampled Na_2_SO_4_ conditions, whose reproducibility in other samples and tissues remains to be tested. Independent expression measurements would address reproducibility, whereas peptide detection and functional studies would be required to determine antimicrobial activity or a causal contribution to salt tolerance. The gene models and expression profiles presented here provide candidates and defined hypotheses for these distinct questions.

The catalogue and each downstream analysis also have different coverage. The seven reconstructed models have transcript support but were not included in the quantification reference or the existing genomic-anchor reference. No comparable differential-expression estimates are therefore available for them. Localization and regulatory annotations remain computational predictions, and short sequences limit some phylogenetic and selection analyses. The *T. chinensis* comparison covers the defined 63-member query subset rather than an exhaustive family inventory, and its NaCl-treated shoots differ from the Na_2_SO_4_-treated *R. soongarica* leaves. These boundaries define the present resource as a basis for studying selected peptide genes in a desert halophyte, with priorities informed by sequence, genomic and expression evidence.

## 5. Conclusions

This study provides a curated genome-wide catalogue of AMP gene families in the xerophytic recretohalophyte *R. soongarica*. Seventy-three candidates were identified across three families: 15 defensins, 18 Snakin/GASA genes and 40 nsLTPs, including seven previously unannotated, transcript-supported gene models. *RsLTP20* is reported separately as a reference-dependent C4-like candidate. Six local phylogenetic groups were supported across four sequence and alignment strategies. Among the 66 members represented in the RNA-seq reference, 18 were differentially expressed at 400 mM Na_2_SO_4_ relative to the control. Most differentially expressed nsLTPs and defensins were induced, whereas three of the four differentially expressed Snakin/GASA genes were repressed. Comparisons with *T. austromongolica* and *A. thaliana* provide genomic context, and the separate *T. chinensis* expression analysis identifies limited response parallels among selected homologous candidates. These results provide a reference for experimental investigation of stress adaptation and peptide function, with *RsLTP12* and other expression-responsive members as candidates for further study.

## Data Availability

No new sequencing data were generated. The *R. soongarica* genome and annotation are available from Figshare (10.6084/m9.figshare.25533064.v2) and GenBank (JBEBFM000000000). Na_2_SO_4_-treatment RNA-seq and Iso-Seq data are available under BioProject PRJNA1063761/SRA study SRP483612 (SRR27540875–SRR27540884, with SRR27540882 as Iso-Seq). The *T. austromongolica* genome and annotation are available as GCA_039764185.1 and from Figshare (10.6084/m9.figshare.25106726). *T. chinensis* RNA-seq data are available under PRJNA855335, with the six selected run accessions listed in Table S40; its genome, annotation, and supporting data are available through GigaDB (10.5524/102417) [45]. Processed tables and analysis files are supplied as Supplementary Tables S1–S59 and Files S1–S11. Code and processed results are available at https://github.com/tarimteacher1/rsoamp-genes-revision/releases/tag/revision-20260909 (11 August 2026; commit 7593467f6fc8a81fe3c7440ecc857f7677ac1991). Additional figure-label documentation is available at https://github.com/tarimteacher1/rsoamp-genes-revision/blob/74083a22f652b4db0948ccedd5eaf2be0a7ee555/READER_LABEL_UPDATE_20260909.md (accessed on 11 August 2026). The 14-pattern promoter analysis, multiple-testing correction and Figure 7 display files are available at https://github.com/tarimteacher1/rsoamp-genes-revision/blob/4734dc35bcb3b90c6c115758f08d126c3fdf468a/PROMOTER14_UPDATE_20260909.md (accessed on 11 August 2026). The repository provides file mappings and checksum manifests for these deposits. The *A. thaliana* reference is NCBI RefSeq assembly GCF_000001735.4 (TAIR10.1; TAIR and Araport annotation). The Arabidopsis collinearity source records and Figure 8 update are supplied in File S11. The 10 September 2026 update supplies Files S10–S11, the current Tables S1–S59, and the current figure PDFs and source mappings at https://github.com/tarimteacher1/rsoamp-genes-revision/releases/tag/revision-20260910 (accessed on 11 August 2026). This update complements the preceding release and its 14-pattern promoter amendment; download links and checksums are listed at https://github.com/tarimteacher1/rsoamp-genes-revision/blob/main/REVISION_UPDATE_20260910.md (accessed on 11 August 2026). The update also documents the source-specific treatment metadata in Table S25.

## Supplementary Materials

The following supporting information can be downloaded at https://www.mdpi.com/article/10.3390/genes17091126/s1: Table S1. Primary catalogue of 73 *R. soongarica* AMP-family candidates. Table S2. Screened candidate inventory and family assignments in *R. soongarica*. Table S3. Integrated evidence for nsLTP-family assignment in *R. soongarica*. Table S4. *R. soongarica* candidates excluded from the primary nsLTP catalogue. Table S5. Genomic and transcript evidence for six genomic AMP-like fragments. Table S6. Genome-wide homology-rescue loci and annotation-overlap assessments. Table S7. Phylogenetic group recurrence and support across four analysis strategies. Table S8. Branch-level SH-aLRT and UFBoot support across four phylogenetic strategies. Table S9. Occurrences of 14 promoter sequence patterns in the primary catalogue. Table S10. Promoter-pattern enrichment against the genomic background. Table S11. Ka/Ks estimates and reliability assessments for selected duplicate pairs. Table S12. Transcript abundance and quantification availability for the primary catalogue. Table S13. DESeq2 normalized counts and quantification availability for the primary catalogue. Table S14. Differential-expression estimates and testing status for the primary catalogue. Table S15. Family-level differential-expression summaries by contrast. Table S16. RNA-seq mapping and quantification quality metrics. Table S17. Classification of transcriptome-unassigned reads. Table S18. AMP-reference sequence matches in residual reads. Table S19. Comparative family counts in the primary and extended catalogues. Table S20. Reciprocal-best-hit sequence pairs between *R. soongarica* and *T. austromongolica*. Table S21. Candidate-involving collinearity anchors between *R. soongarica* and *T. austromongolica*. Table S22. Candidates proposed for future qRT-PCR validation. Table S23. Transcript mappability and read-assignment ambiguity assessments. Table S24. Candidate reference genes proposed for future experimental stability assessment. Table S25. Treatment metadata and source-evidence records. Table S26. Expression-filtering status for the primary catalogue. Table S27. Definitions, sources and interpretation limits of 14 promoter sequence patterns. Table S28. Promoter intervals and sequence-quality assessments for the primary catalogue. Table S29. Promoter-pattern enrichment sensitivity across three interval-selection scenarios. Table S30. Unified primary catalogues of *R. soongarica* and *T. austromongolica*. Table S31. Extended candidate catalogues including reference-dependent assignments. Table S32. Shared nsLTP decision criteria and candidate-level evidence. Table S33. Reconstructed transcript-model evidence and inclusion decisions. Table S34. Gene coverage and interpretation limits of individual analyses. Table S35. Sensitivity of family assignments and comparative results to catalogue boundaries. Table S36. Differential-expression results for 66 members represented in the quantification reference. Table S37. Transcript abundance for 66 members represented in the quantification reference. Table S38. Expression-filtering status for 66 members represented in the quantification reference. Table S39. Subcellular-localization and secretion-related predictions with sequence provenance. Table S40. Sample metadata and provenance for the *T. chinensis* expression dataset. Table S41. Published annotations used to select 41 *T. chinensis* genes. Table S42. Expression results for 41 annotation-selected *T. chinensis* genes. Table S43. Sequencing and quantification quality metrics for *T. chinensis* samples. Table S44. Family-specific protein-motif definitions. Table S45. Protein-motif sites in primary-catalogue sequences. Table S46. Gene-structure features and model-source annotations. Table S47. Query membership and analysis coverage for the *R. soongarica*–*T. chinensis* comparison. Table S48. Homology, reciprocal-hit, alignment-coverage and collinearity evidence for cross-species candidate pairs. Table S49. Paired expression estimates and testing status for cross-species candidate comparisons. Table S50. Cross-species paired-expression summaries and sensitivity analyses. Table S51. Predicted physicochemical properties of 73 complete precursor sequences. Table S52. Family-level summaries of precursor physicochemical properties. Table S53. Member-level duplication-mode assignments and assessment status. Table S54. Family-level duplication-mode counts. Table S55. Within-species collinearity anchors for seven segmental duplicate pairs. Table S56. nsLTP cysteine-core spacing and predicted secretion architecture. Table S57. Collinearity anchors between *R. soongarica* and *A. thaliana*. Table S58. Arabidopsis comparison coverage for the primary *R. soongarica* catalogue. Table S59. Member-level collinearity correspondence across the two comparator species.
