# Supplementary material for: Genome-Wide Identification, Evolutionary Characterization, and Salt-Stress Expression Profiling of Antimicrobial Peptide Gene Families in the Xerophytic Recretohalophyte Reaumuria soongarica
Source: Genes (Basel). 2026 Sep 16;17(9):1126. doi: 10.3390/genes17091126 (PMC13606368; doi:10.3390/genes17091126)
Supplement: Supplementary file 1 [file genes-17-01126-s001.zip › Figures/FigureS3.pdf]

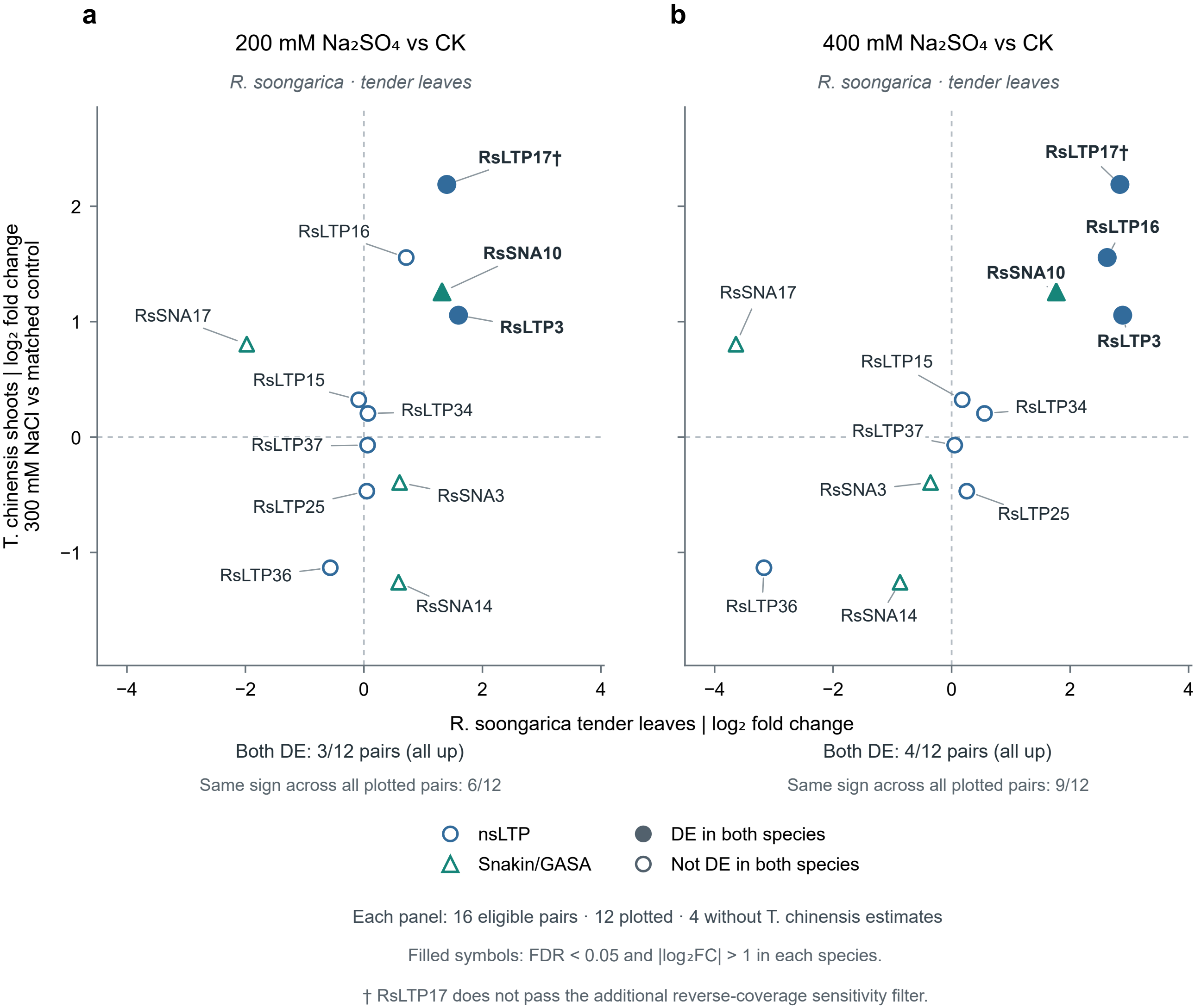
a
200 mM Na₂SO₄ vs CK
R. soongarica · tender leaves
b
400 mM Na₂SO₄ vs CK
R. soongarica · tender leaves
RsLTP17†
RsLTP16
RsSNA10
RsLTP3
RsSNA17
RsLTP15
RsLTP34
RsLTP37
RsLTP25
RsSNA3
RsLTP36
RsSNA14
T. chinensis shoots | log₂ fold change
300 mM NaCl vs matched control
R. soongarica tender leaves | log₂ fold change
Both DE: 3/12 pairs (all up)
Same sign across all plotted pairs: 6/12
Both DE: 4/12 pairs (all up)
Same sign across all plotted pairs: 9/12
nsLTP
Snakin/GASA
DE in both species
Not DE in both species
Each panel: 16 eligible pairs · 12 plotted · 4 without T. chinensis estimates
Filled symbols: FDR < 0.05 and |log₂FC| > 1 in each species.
† RsLTP17 does not pass the additional reverse-coverage sensitivity filter.
