# Supplementary material for: Genome-Wide Identification, Evolutionary Characterization, and Salt-Stress Expression Profiling of Antimicrobial Peptide Gene Families in the Xerophytic Recretohalophyte Reaumuria soongarica
Source: Genes (Basel). 2026 Sep 16;17(9):1126. doi: 10.3390/genes17091126 (PMC13606368; doi:10.3390/genes17091126)
Supplement: Supplementary file 1 [file genes-17-01126-s001.zip › Files/File_S11_Arabidopsis_comparison_20260910.zip!/File_S11/figures/Arabidopsis_all_26_anchor_contexts.pdf]

R. soongarica–A. thaliana: all candidate-anchor contexts

Independent local coordinate scales; colours follow the R. soongarica candidate family.

01 RsLTP25 — AT1G55260 | Block 42

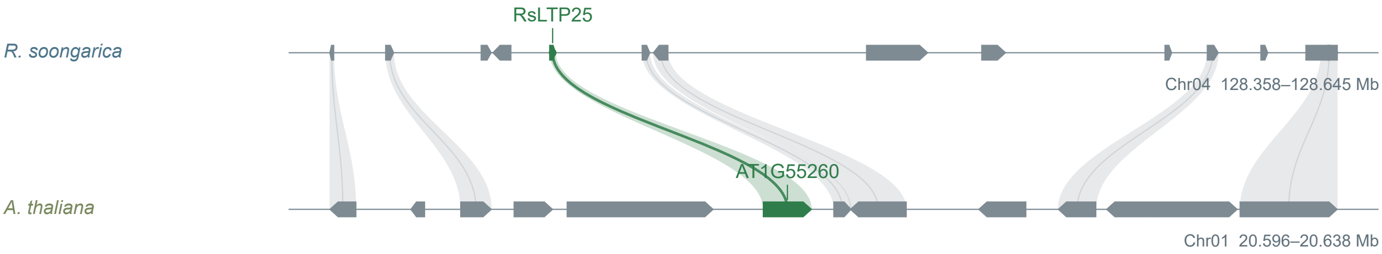

02 RsSNA8 — AT1G75750 | Block 82

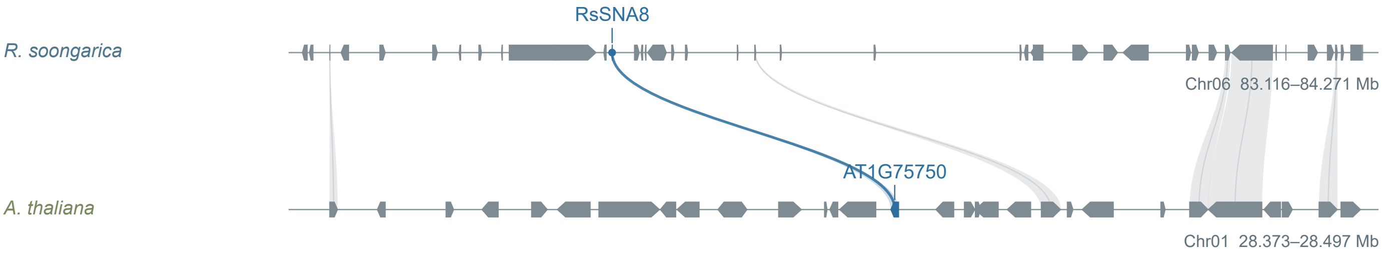

03 RsLTP27 — AT1G73780 | Block 83

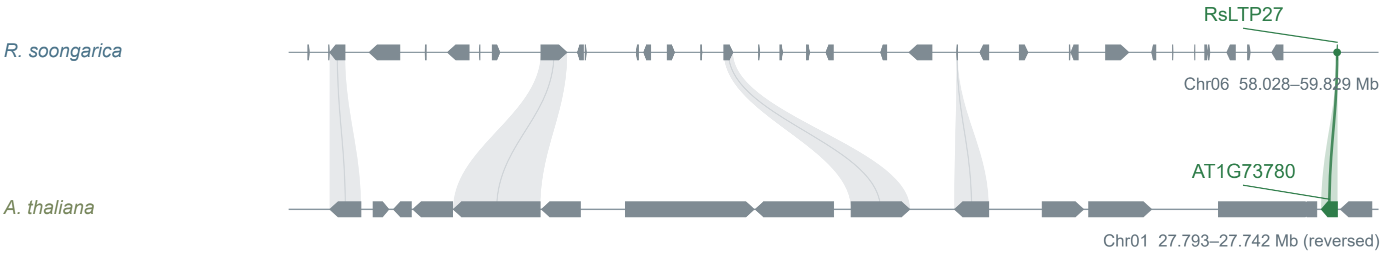

04 RsLTP30 — AT1G27950 | Block 100

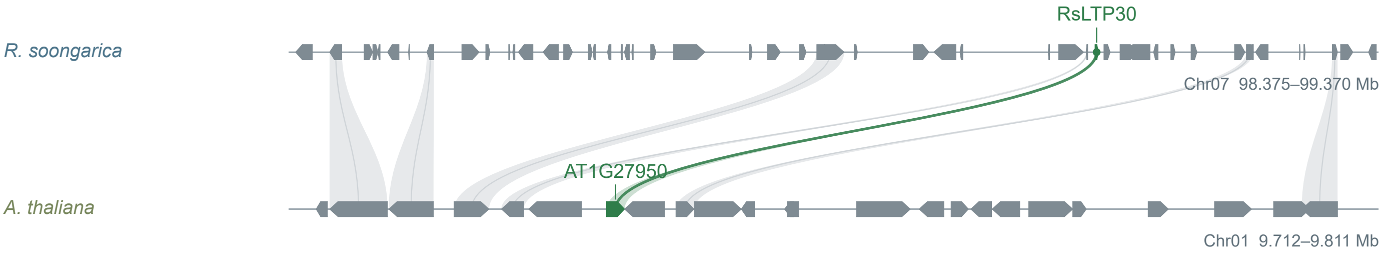

Complete pair, scope and reference records are provided in Tables S51–S53 and File S10.

R. soongarica–A. thaliana: all candidate-anchor contexts

Independent local coordinate scales; colours follow the R. soongarica candidate family.

05 RsLTP34 — AT1G03103 | Block 134

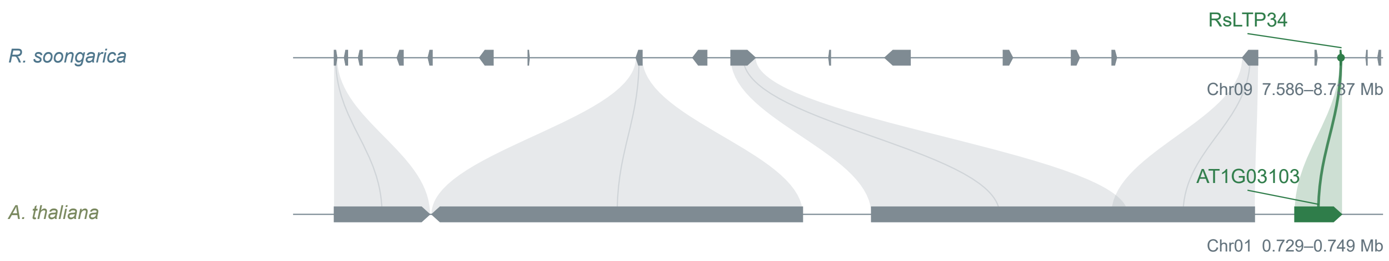

06 RsSNA17 — AT1G75750 | Block 176

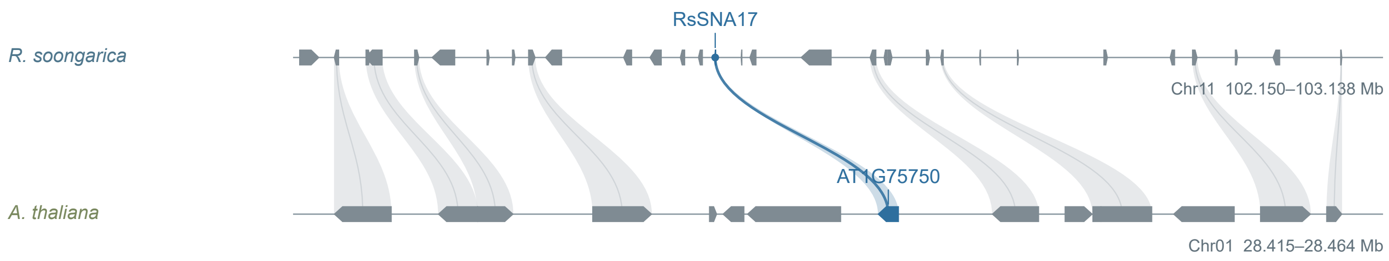

07 RsSNA14 — AT1G22690 | Block 194

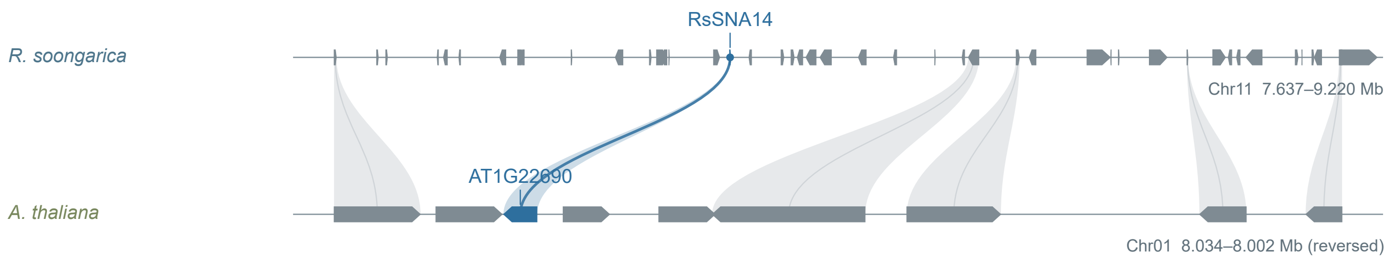

08 RsLTP9 — AT2G38540 | Block 215

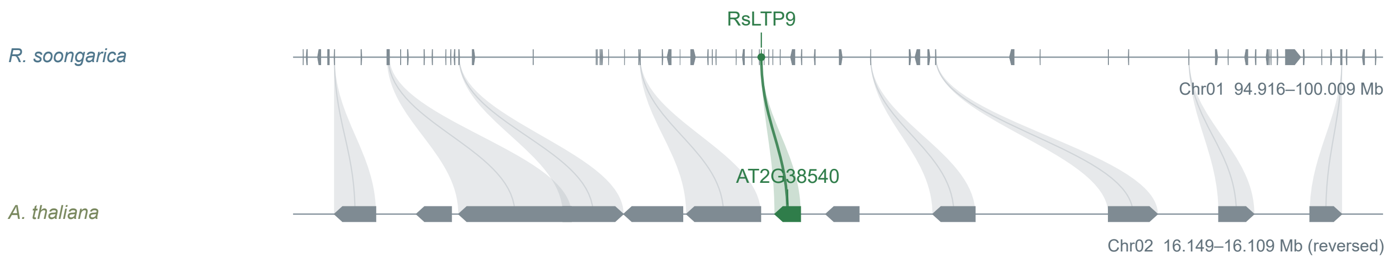

Complete pair, scope and reference records are provided in Tables S51–S53 and File S10.

R. soongarica–A. thaliana: all candidate-anchor contexts

Independent local coordinate scales; colours follow the R. soongarica candidate family.

09 RsLTP23 — AT2G38540 | Block 258

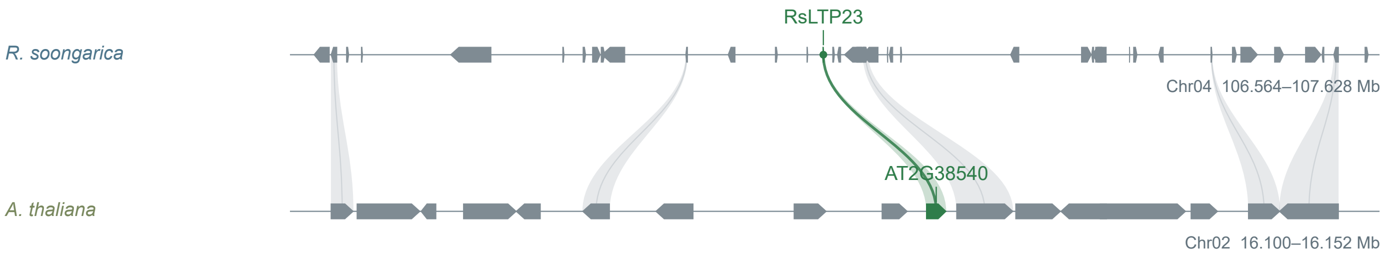

10 RsLTP14 — AT3G53980 | Block 348

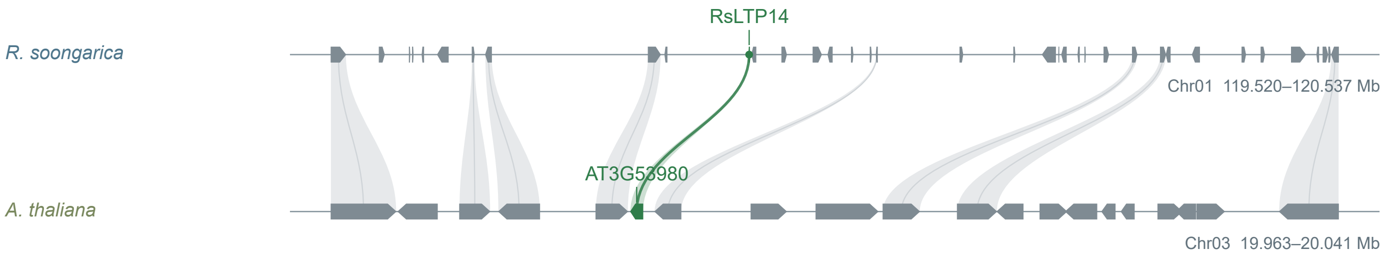

11 RsLTP13 — AT3G08770 | Block 349

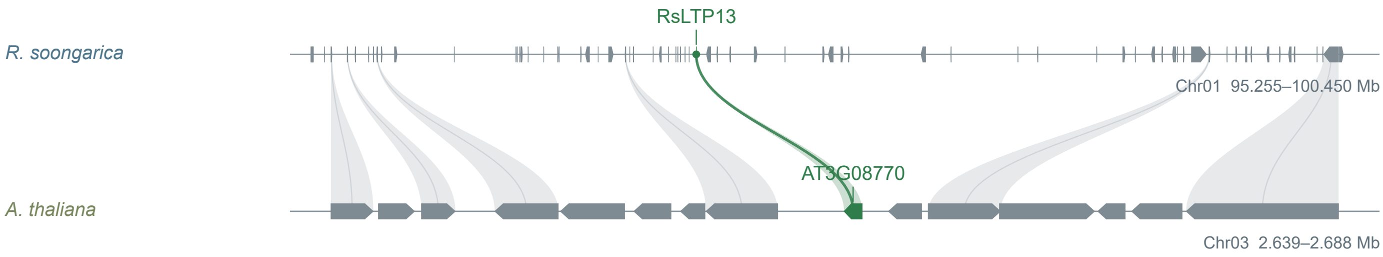

12 RsLTP10 — AT3G51600 | Block 353

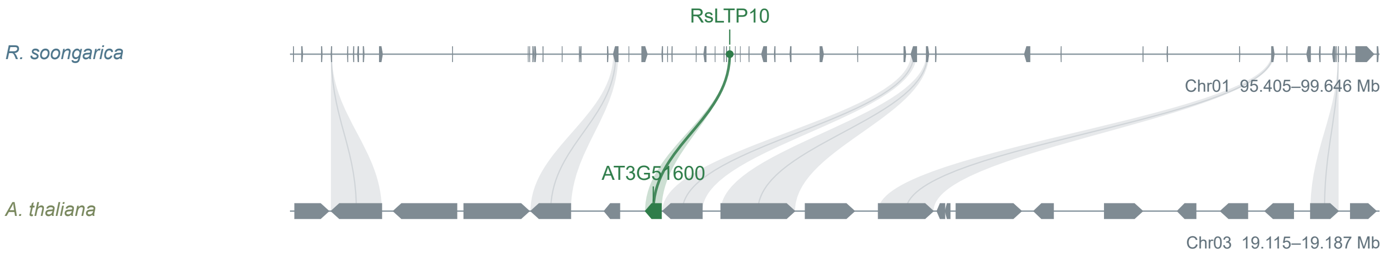

Complete pair, scope and reference records are provided in Tables S51–S53 and File S10.

R. soongarica–A. thaliana: all candidate-anchor contexts

Independent local coordinate scales; colours follow the R. soongarica candidate family.

13 RsLTP17 — AT3G22620 | Block 364

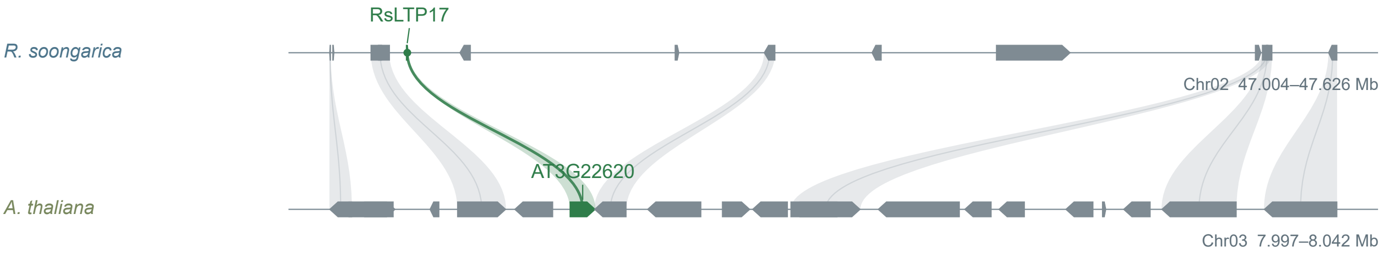

14 RsLTP16 — AT3G22600 | Block 370

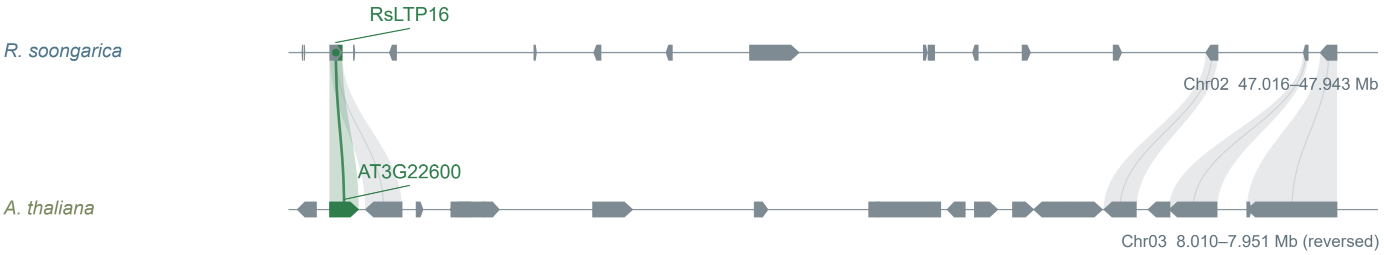

15 RsLTP19 — AT3G43720 | Block 380

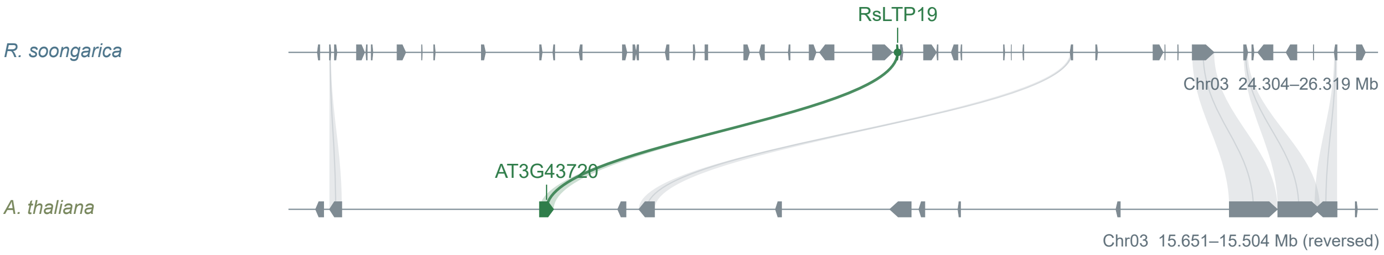

16 RsLTP27 — AT3G18280 | Block 433

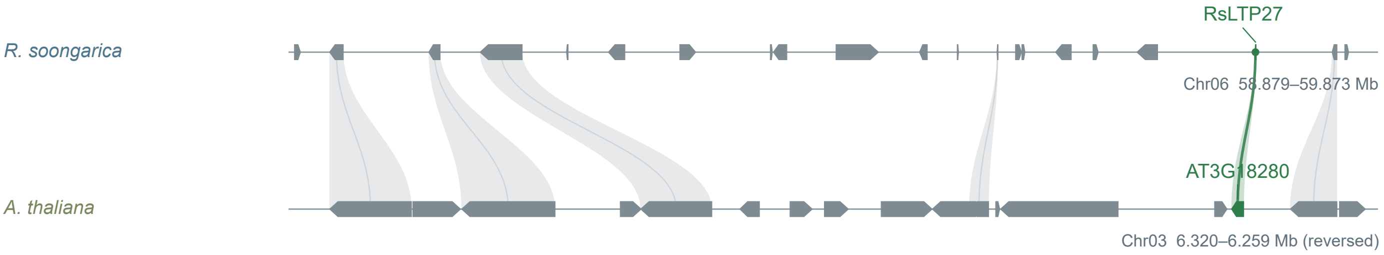

Complete pair, scope and reference records are provided in Tables S51–S53 and File S10.

R. soongarica–A. thaliana: all candidate-anchor contexts

Independent local coordinate scales; colours follow the R. soongarica candidate family.

17 RsSNA10 — AT3G10185 | Block 472

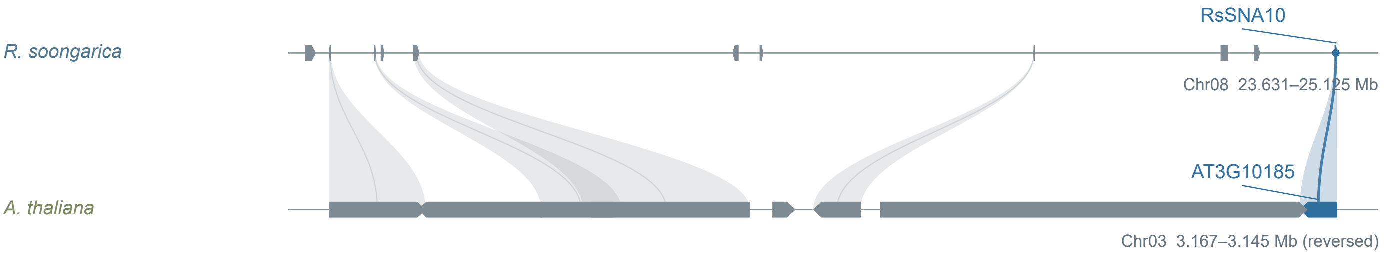

18 RsLTP35 — AT3G22580 | Block 478

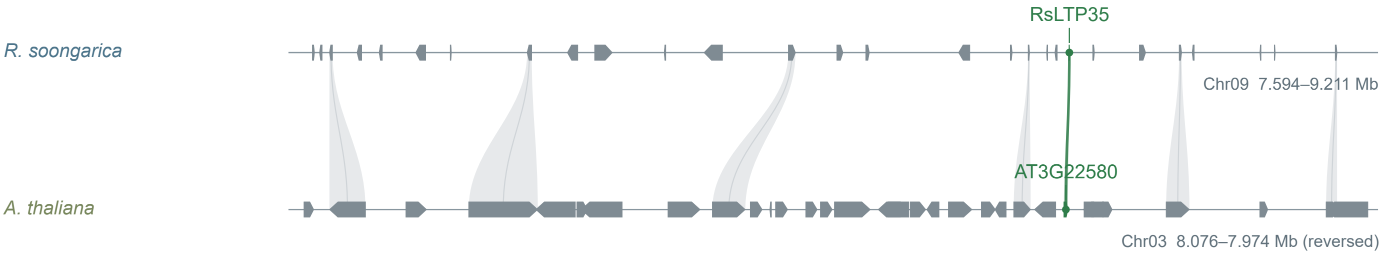

19 RsLTP34 — AT3G22600 | Block 478

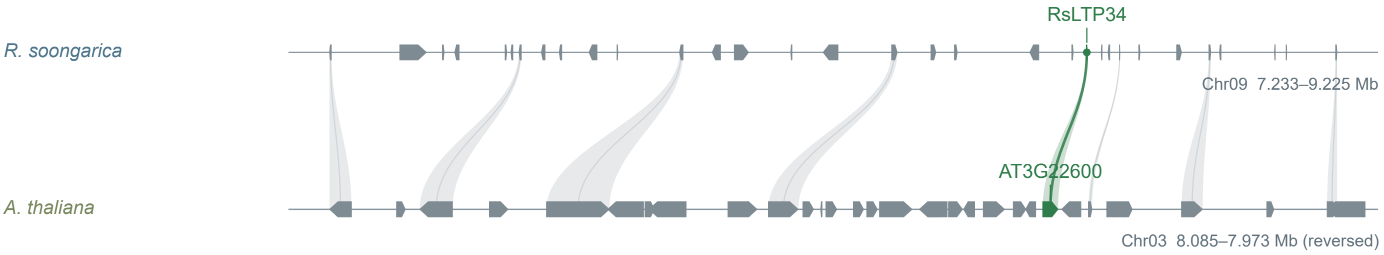

20 RsLTP34 — AT4G14815 | Block 595

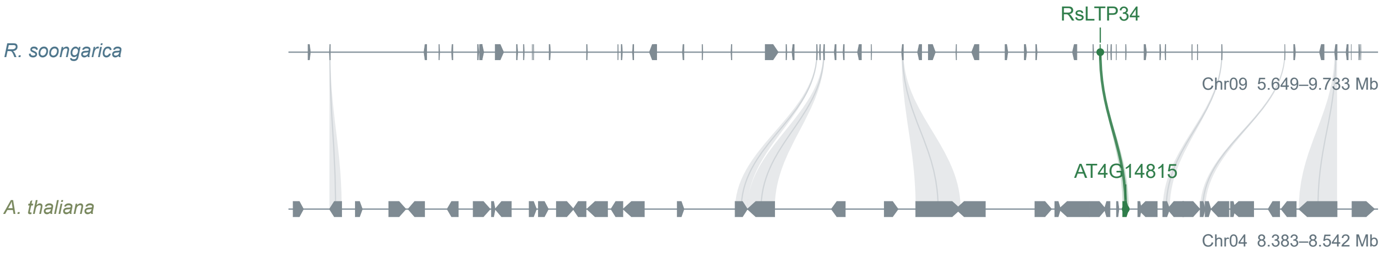

Complete pair, scope and reference records are provided in Tables S51–S53 and File S10.

R. soongarica–A. thaliana: all candidate-anchor contexts

Independent local coordinate scales; colours follow the R. soongarica candidate family.

21 RsSNA14 — AT4G09600 | Block 625

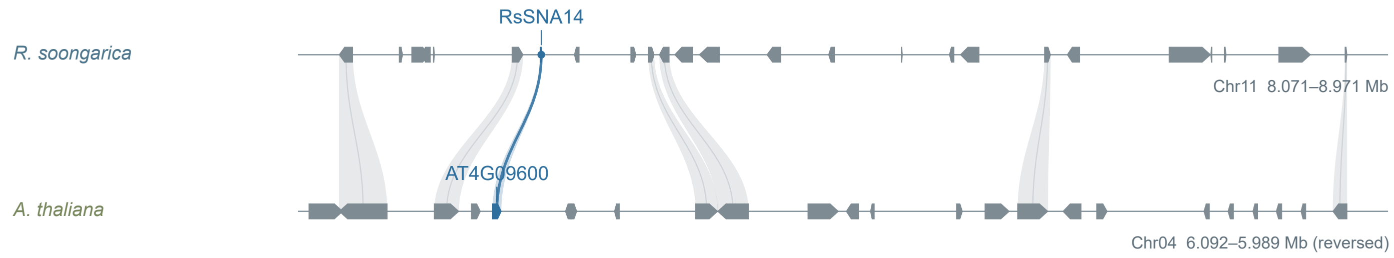

22 RsLTP5 — AT5G01870 | Block 644

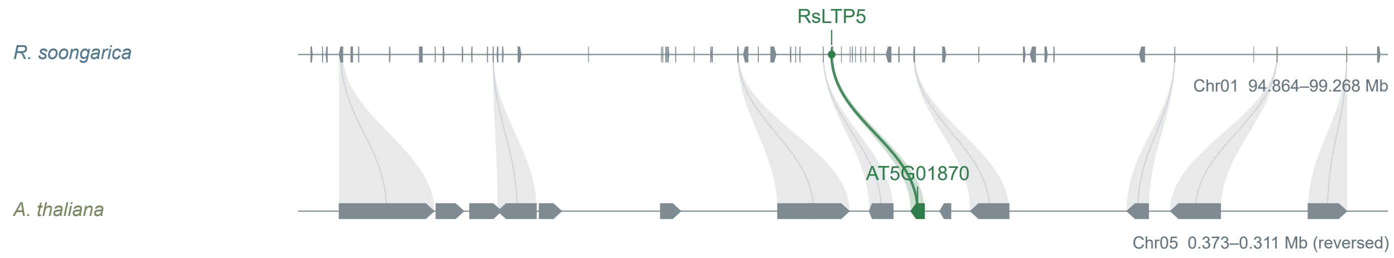

23 RsLTP22 — AT5G01870 | Block 691

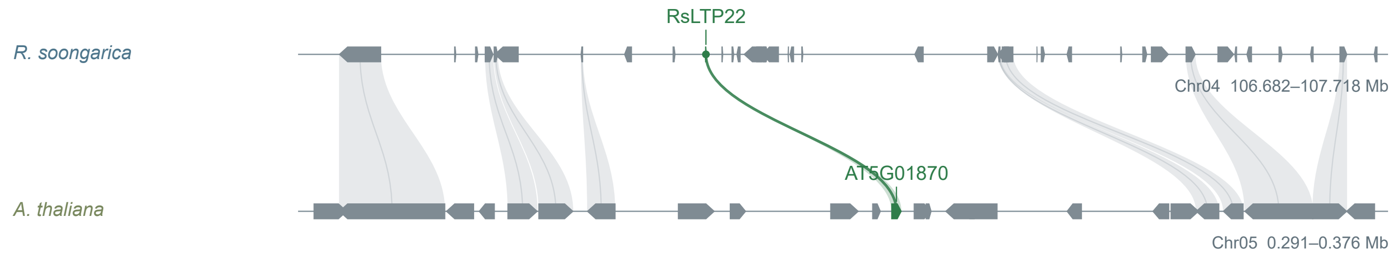

24 RsLTP21 — AT5G62065 | Block 703

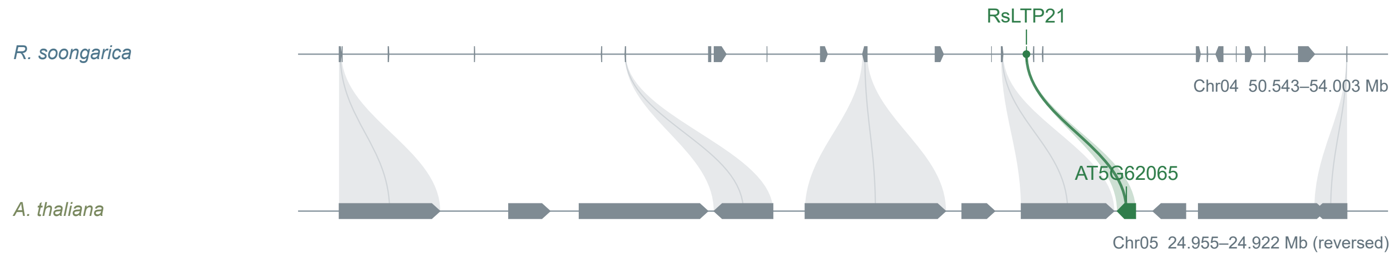

Complete pair, scope and reference records are provided in Tables S51–S53 and File S10.

R. soongarica–A. thaliana: all candidate-anchor contexts

Independent local coordinate scales; colours follow the R. soongarica candidate family.

25 RsSNA5 — AT5G14920 | Block 707

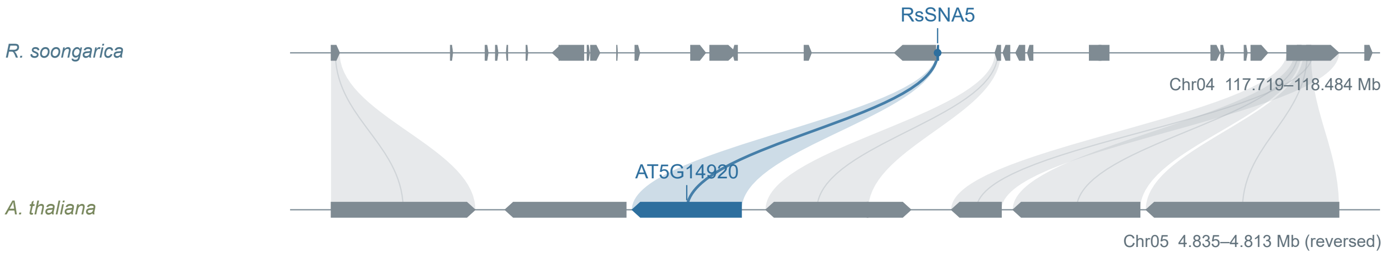

26 RsLTP38 — AT5G44265 | Block 798

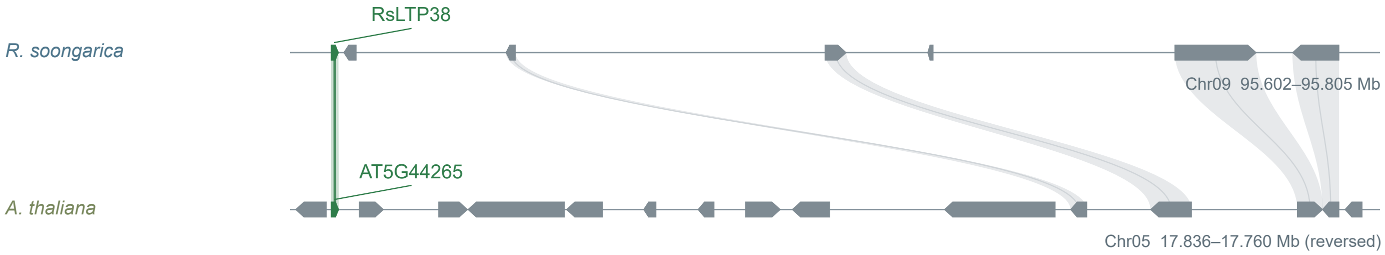

Complete pair, scope and reference records are provided in Tables S51–S53 and File S10.
