# Supplementary material for: Genome-Wide Identification, Evolutionary Characterization, and Salt-Stress Expression Profiling of Antimicrobial Peptide Gene Families in the Xerophytic Recretohalophyte Reaumuria soongarica
Source: Genes (Basel). 2026 Sep 16;17(9):1126. doi: 10.3390/genes17091126 (PMC13606368; doi:10.3390/genes17091126)
Supplement: Supplementary file 1 [file genes-17-01126-s001.zip › Files/File_S11_Arabidopsis_comparison_20260910.zip!/File_S11/figures/Figure8_current_author.pdf]

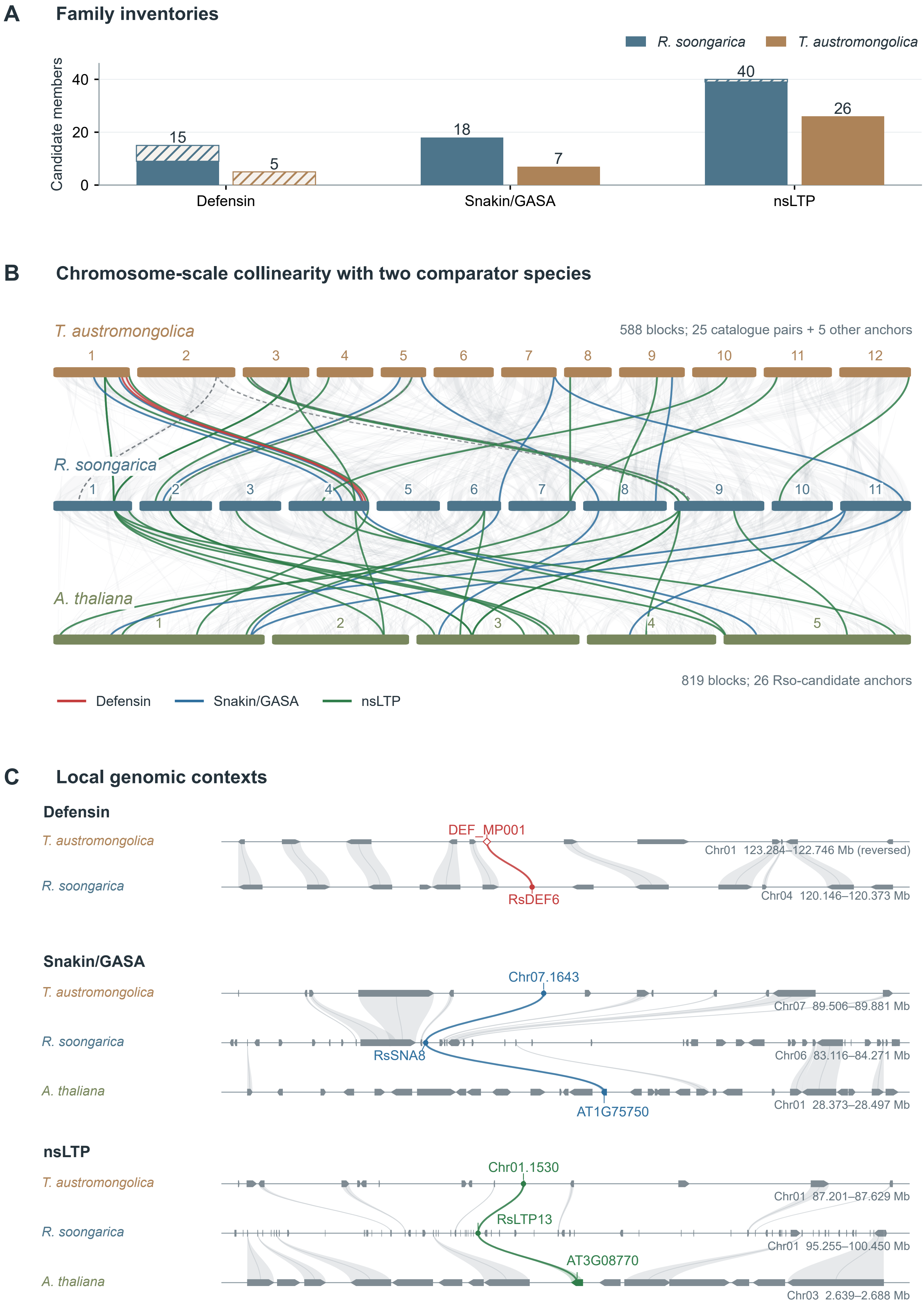
A
Family inventories
R. soongarica
T. austromongolica
Candidate members
0
20
40
15
5
18
7
40
26
Defensin
Snakin/GASA
nsLTP
B
Chromosome-scale collinearity with two comparator species
T. austromongolica
588 blocks; 25 catalogue pairs + 5 other anchors
R. soongarica
A. thaliana
819 blocks; 26 Rso-candidate anchors
Defensin
Snakin/GASA
nsLTP
C
Local genomic contexts
Defensin
T. austromongolica
R. soongarica
DEF_MP001
RsDEF6
Chr01 123.284–122.746 Mb (reversed)
Chr04 120.146–120.373 Mb
Snakin/GASA
T. austromongolica
R. soongarica
A. thaliana
Chr07.1643
RsSNA8
AT1G75750
Chr07 89.506–89.881 Mb
Chr06 83.116–84.271 Mb
Chr01 28.373–28.497 Mb
nsLTP
T. austromongolica
R. soongarica
A. thaliana
Chr01.1530
RsLTP13
AT3G08770
Chr01 87.201–87.629 Mb
Chr01 95.255–100.450 Mb
Chr03 2.639–2.688 Mb
