# Supplementary material for: Genome-Wide Identification, Evolutionary Characterization, and Salt-Stress Expression Profiling of Antimicrobial Peptide Gene Families in the Xerophytic Recretohalophyte Reaumuria soongarica
Source: Genes (Basel). 2026 Sep 16;17(9):1126. doi: 10.3390/genes17091126 (PMC13606368; doi:10.3390/genes17091126)
Supplement: Supplementary file 1 [file genes-17-01126-s001.zip › Files/File_S11_Arabidopsis_comparison_20260910.zip!/File_S11/figures/Figure8_Rso_Tamarix_Arabidopsis_20260910.pdf]

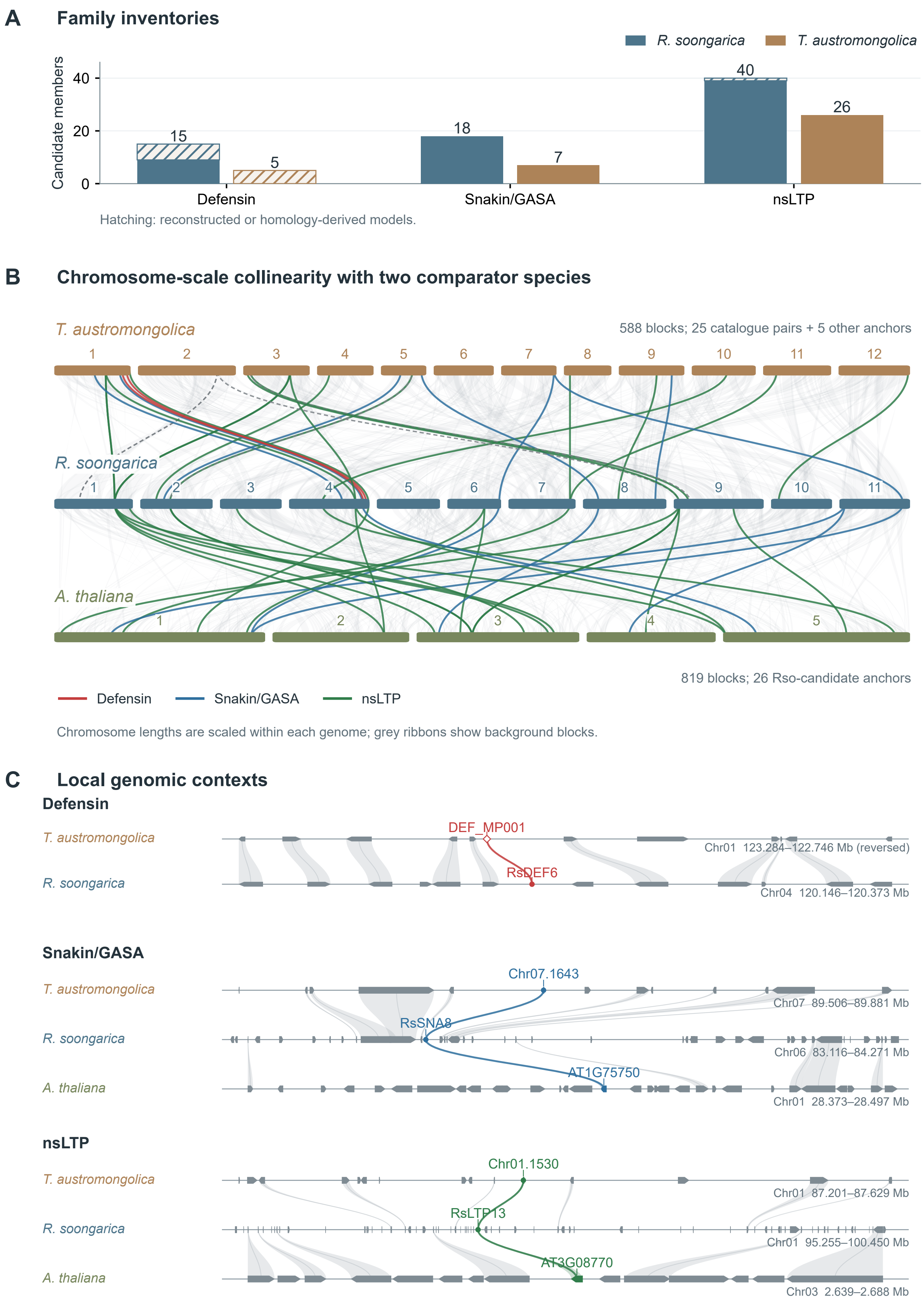
A
Family inventories
R. soongarica
T. austromongolica
Candidate members
0
20
40
15
5
18
7
40
26
Defensin
Snakin/GASA
nsLTP
Hatching: reconstructed or homology-derived models.
B
Chromosome-scale collinearity with two comparator species
T. austromongolica
588 blocks; 25 catalogue pairs + 5 other anchors
1 2 3 4 5 6 7 8 9 10 11 12
R. soongarica
1 2 3 4 5 6 7 8 9 10 11
A. thaliana
1 2 3 4 5
819 blocks; 26 Rso-candidate anchors
Defensin
Snakin/GASA
nsLTP
Chromosome lengths are scaled within each genome; grey ribbons show background blocks.
C
Local genomic contexts
Defensin
T. austromongolica
DEF_MP001
Chr01 123.284–122.746 Mb (reversed)
R. soongarica
RsDEF6
Chr04 120.146–120.373 Mb
Snakin/GASA
T. austromongolica
Chr07.1643
Chr07 89.506–89.881 Mb
R. soongarica
RsSNA8
Chr06 83.116–84.271 Mb
A. thaliana
AT1G75750
Chr01 28.373–28.497 Mb
nsLTP
T. austromongolica
Chr01.1530
Chr01 87.201–87.629 Mb
R. soongarica
RsLTP13
Chr01 95.255–100.450 Mb
A. thaliana
AT3G08770
Chr03 2.639–2.688 Mb
