# Supplementary material for: Genome-Wide Identification, Evolutionary Characterization, and Salt-Stress Expression Profiling of Antimicrobial Peptide Gene Families in the Xerophytic Recretohalophyte Reaumuria soongarica
Source: Genes (Basel). 2026 Sep 16;17(9):1126. doi: 10.3390/genes17091126 (PMC13606368; doi:10.3390/genes17091126)

Inter-species synteny: 27 AMP-anchored syntenic pairs (R. soongarica – A. thaliana)

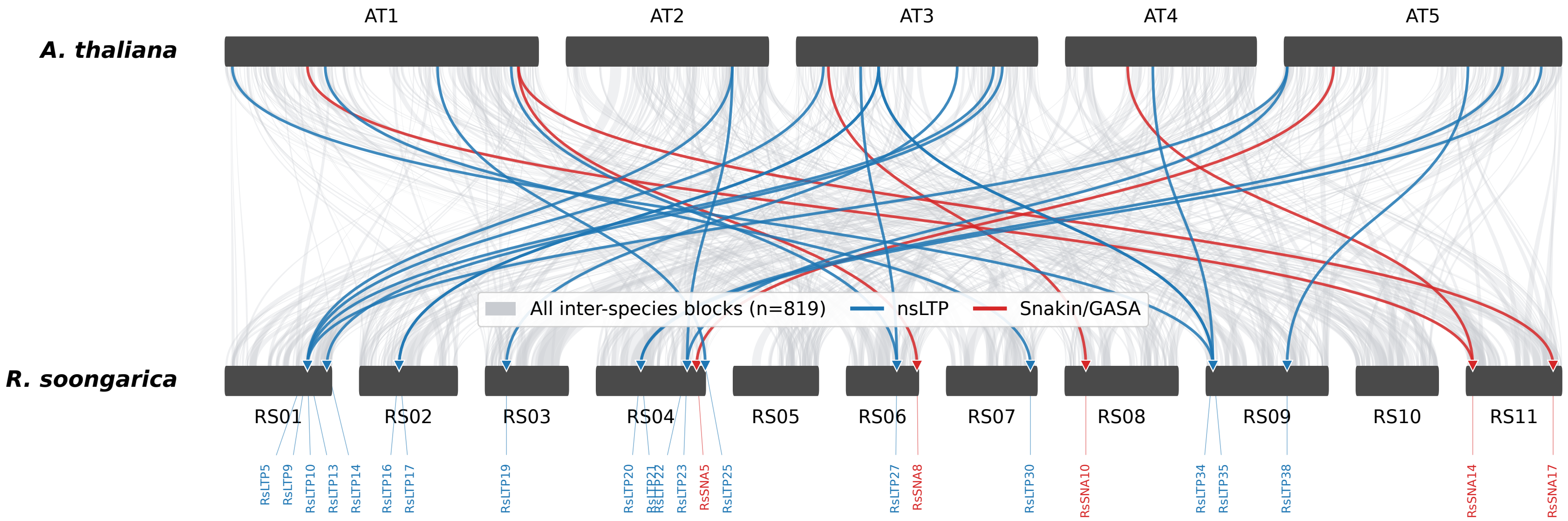

Supplement: Supplementary file 1 [file genes-17-01126-s001.zip › Files/File_S11_Arabidopsis_comparison_20260910.zip!/File_S11/sources/arabidopsis/figures/F8_synteny.pdf]
