# Supplementary material for: Genome-Wide Identification, Evolutionary Characterization, and Salt-Stress Expression Profiling of Antimicrobial Peptide Gene Families in the Xerophytic Recretohalophyte Reaumuria soongarica
Source: Genes (Basel). 2026 Sep 16;17(9):1126. doi: 10.3390/genes17091126 (PMC13606368; doi:10.3390/genes17091126)
Supplement: Supplementary file 1 [file genes-17-01126-s001.zip › Files/File_S2_outgroup_rooting_20260908.zip!/Figures/Conditional_rooted_trees_9pages.pdf]

Conditionally rooted ML tree - no root is endorsed as the resolved family history

1/9 Defensin | DEF_Gymnosperms10
full length / untrimmed | WAG+R4 | DEF conditional root

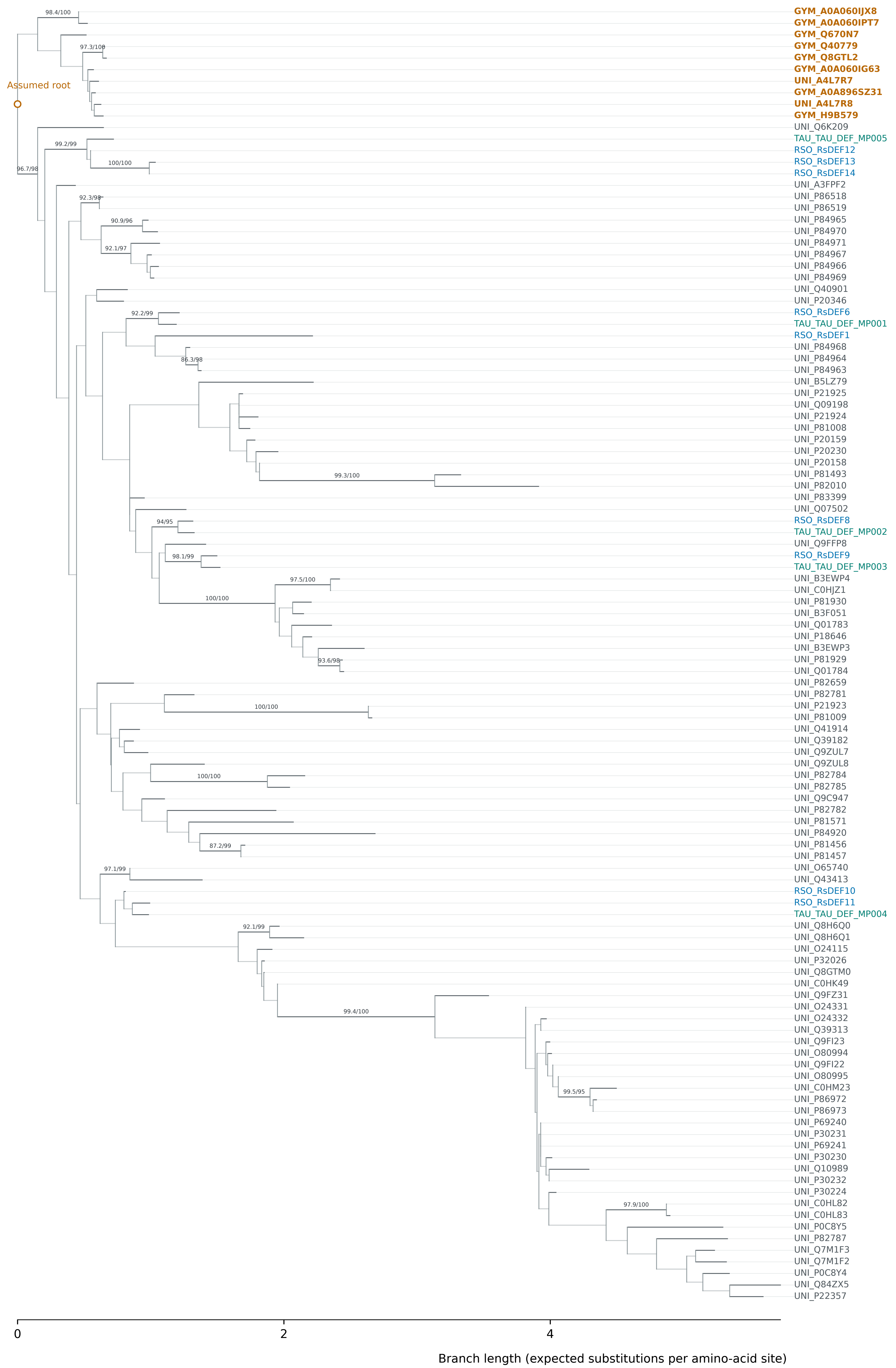

Orange: candidate outgroup; blue: R. soongarica; green: Tamarix; grey: other references.
Labels show SH-aLRT / UFBoot only when both are >=80 / >=95; all estimates remain in raw trees.
All tips and edge lengths retained. Root-edge length was split equally for representation, not time inference.

Conditionally rooted ML tree - no root is endorsed as the resolved family history

2/9 nsLTP | LTP_2S_Arabidopsis5
first-to-last Cys span / gappyout | LG+R4 | R1

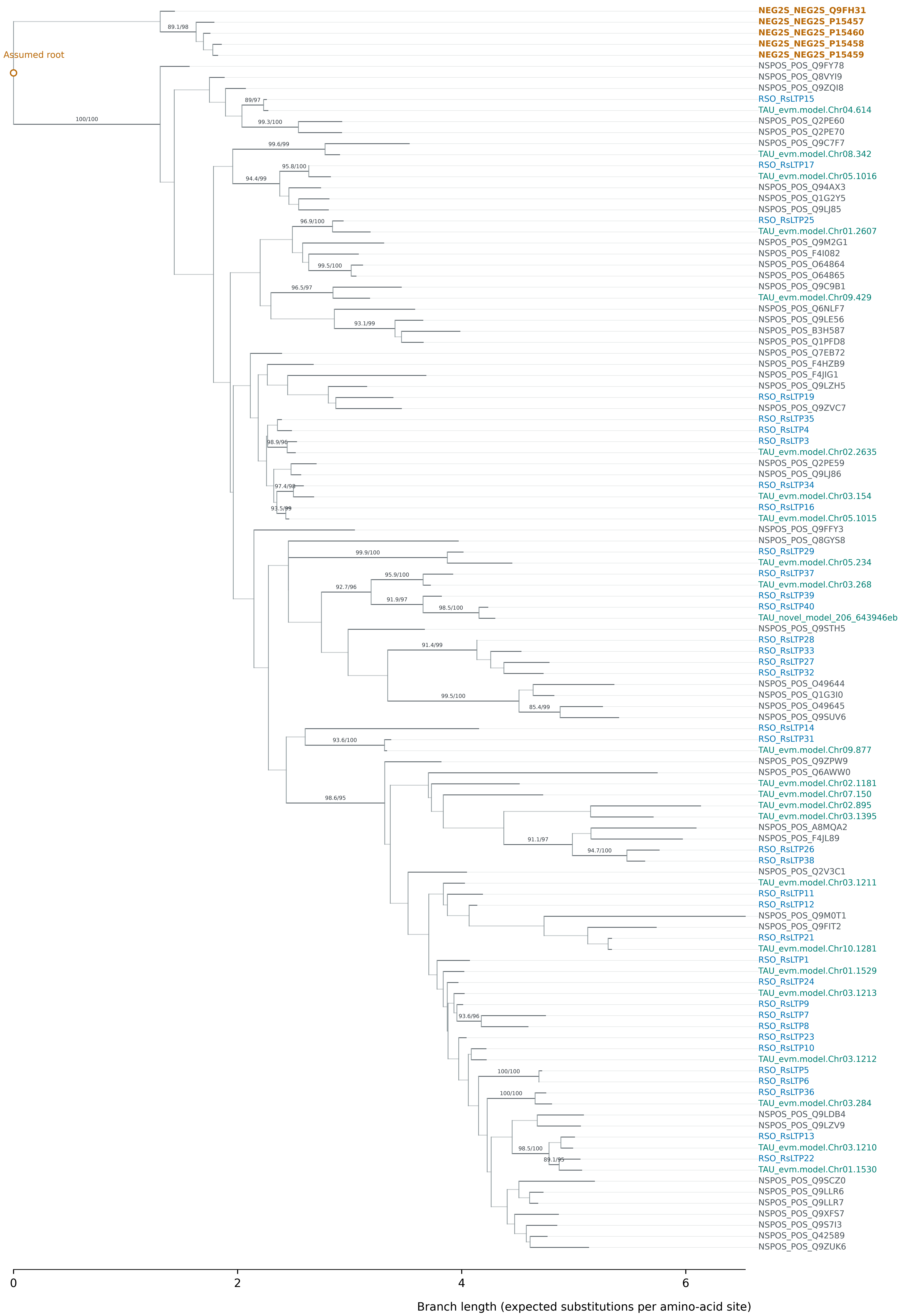

Orange: candidate outgroup; blue: R. soongarica; green: Tamarix; grey: other references.
Labels show SH-aLRT / UFBoot only when both are >=80 / >=95; all estimates remain in raw trees.
All tips and edge lengths retained. Root-edge length was split equally for representation, not time inference.

Conditionally rooted ML tree - no root is endorsed as the resolved family history

3/9 nsLTP | LTP_2S_Arabidopsis5
full length / gappyout | LG+R4 | R2

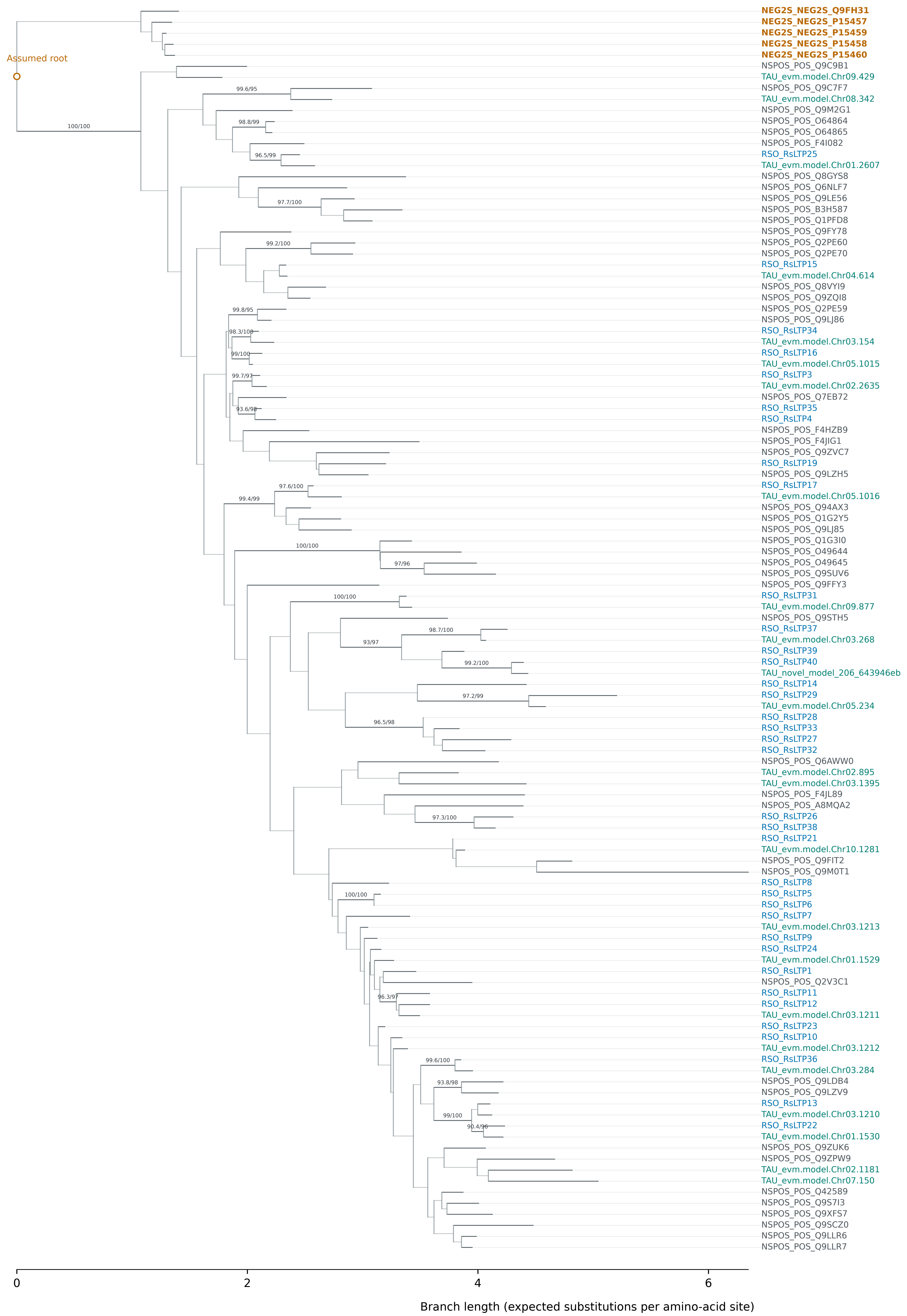

Orange: candidate outgroup; blue: R. soongarica; green: Tamarix; grey: other references.
Labels show SH-aLRT / UFBoot only when both are >=80 / >=95; all estimates remain in raw trees.
All tips and edge lengths retained. Root-edge length was split equally for representation, not time inference.

Conditionally rooted ML tree - no root is endorsed as the resolved family history

4/9 nsLTP | LTP_2S_G7_3
first-to-last Cys span / gappyout | LG+R4 | R3

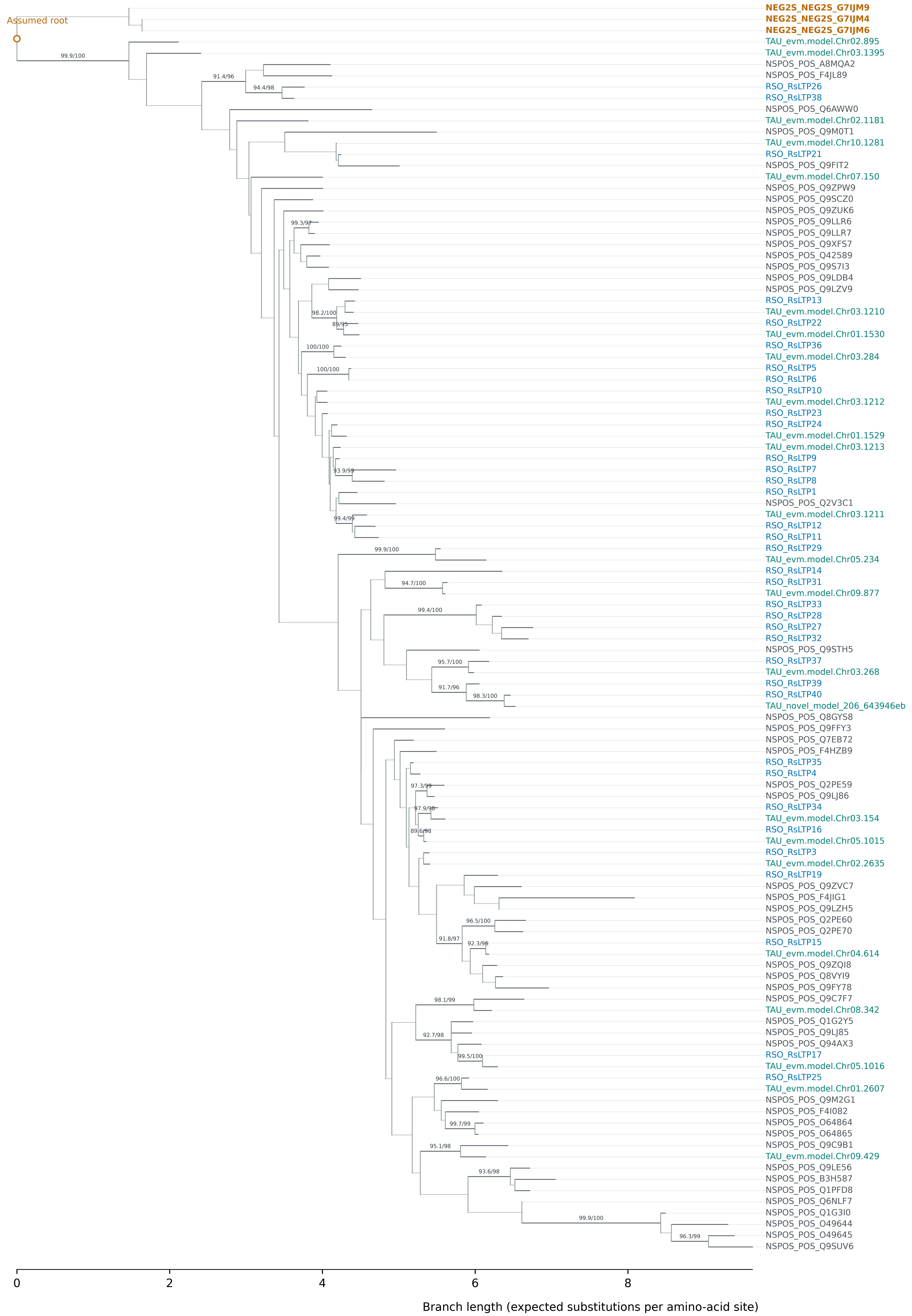

Orange: candidate outgroup; blue: R. soongarica; green: Tamarix; grey: other references.
Labels show SH-aLRT / UFBoot only when both are >=80 / >=95; all estimates remain in raw trees.
All tips and edge lengths retained. Root-edge length was split equally for representation, not time inference.

Conditionally rooted ML tree - no root is endorsed as the resolved family history

5/9 nsLTP | LTP_2S_G7_3
full length / gappyout | LG+R4 | R4

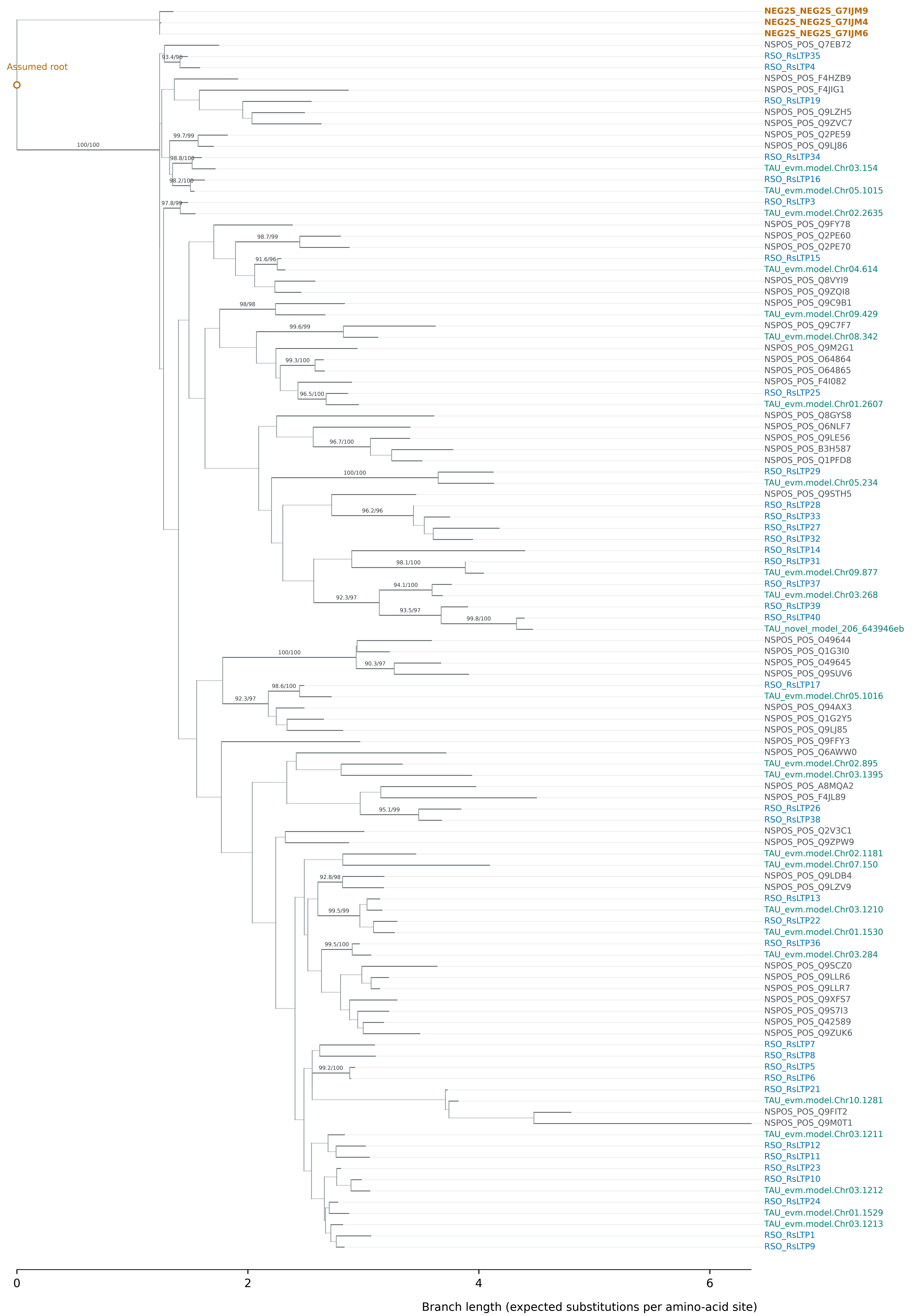

Orange: candidate outgroup; blue: R. soongarica; green: Tamarix; grey: other references.
Labels show SH-aLRT / UFBoot only when both are >=80 / >=95; all estimates remain in raw trees.
All tips and edge lengths retained. Root-edge length was split equally for representation, not time inference.

Conditionally rooted ML tree - no root is endorsed as the resolved family history

6/9 nsLTP | LTP_2S_all8
first-to-last Cys span / gappyout | LG+R4 | R5

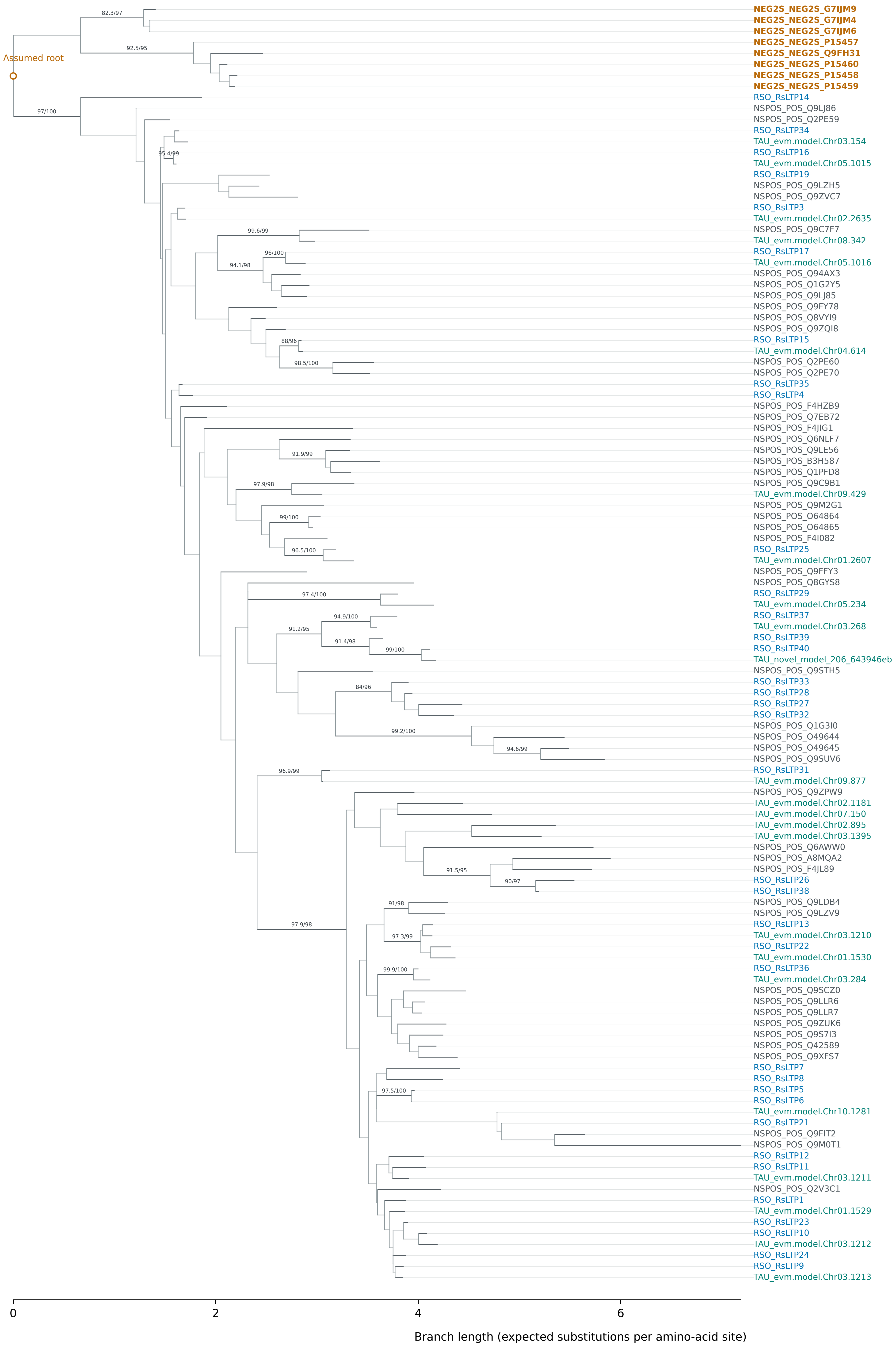

Orange: candidate outgroup; blue: R. soongarica; green: Tamarix; grey: other references.
Labels show SH-aLRT / UFBoot only when both are >=80 / >=95; all estimates remain in raw trees.
All tips and edge lengths retained. Root-edge length was split equally for representation, not time inference.

Conditionally rooted ML tree - no root is endorsed as the resolved family history

7/9 nsLTP | LTP_2S_all8
first-to-last Cys span / untrimmed | LG+R4 | R5

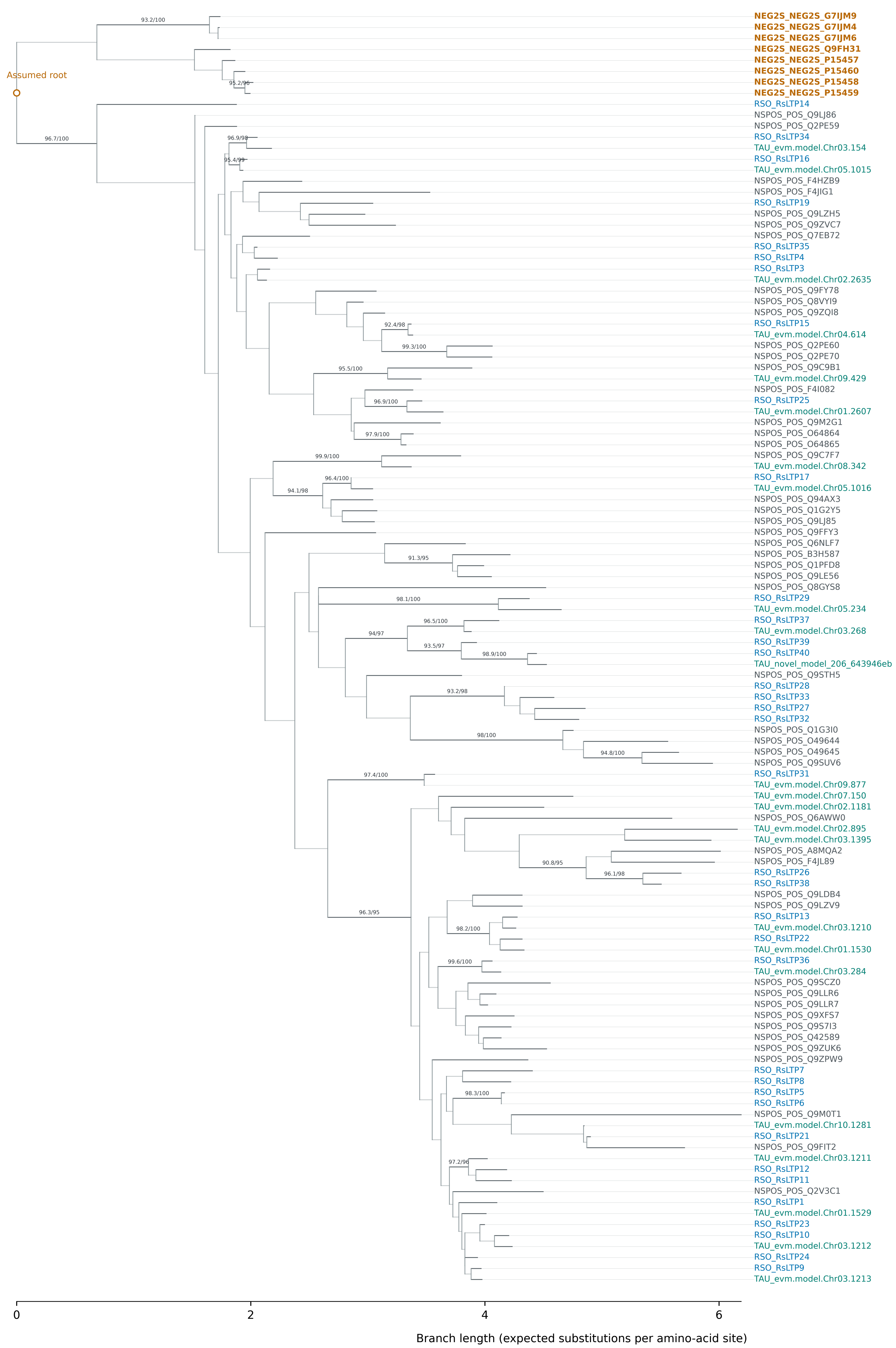

Orange: candidate outgroup; blue: R. soongarica; green: Tamarix; grey: other references.
Labels show SH-aLRT / UFBoot only when both are >=80 / >=95; all estimates remain in raw trees.
All tips and edge lengths retained. Root-edge length was split equally for representation, not time inference.

Conditionally rooted ML tree - no root is endorsed as the resolved family history

8/9 nsLTP | LTP_2S_all8
full length / gappyout | LG+R4 | R6

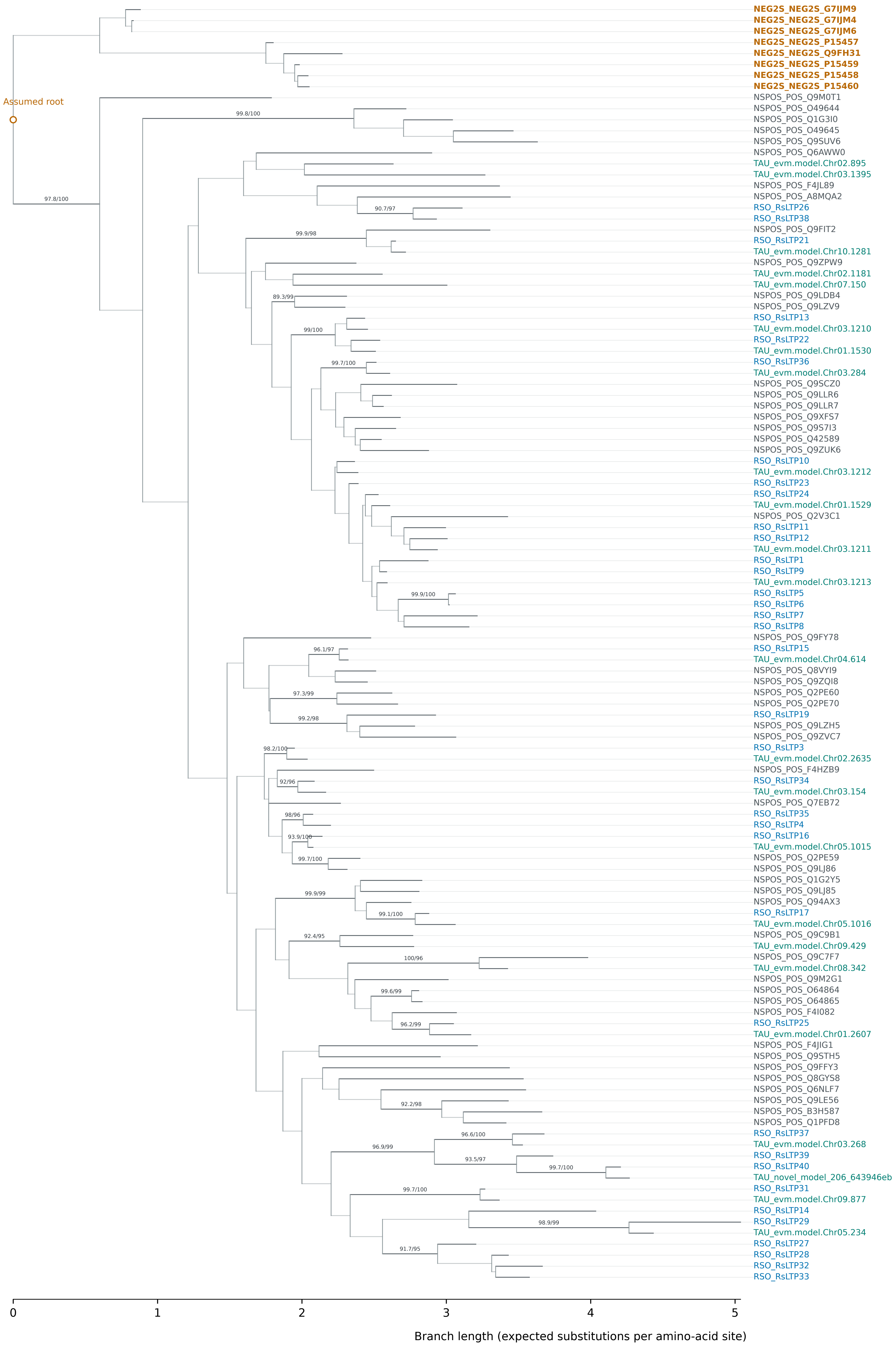

Orange: candidate outgroup; blue: R. soongarica; green: Tamarix; grey: other references.
Labels show SH-aLRT / UFBoot only when both are >=80 / >=95; all estimates remain in raw trees.
All tips and edge lengths retained. Root-edge length was split equally for representation, not time inference.

Conditionally rooted ML tree - no root is endorsed as the resolved family history

9/9 nsLTP | LTP_2S_all8
full length / untrimmed | WAG+R4 | R2

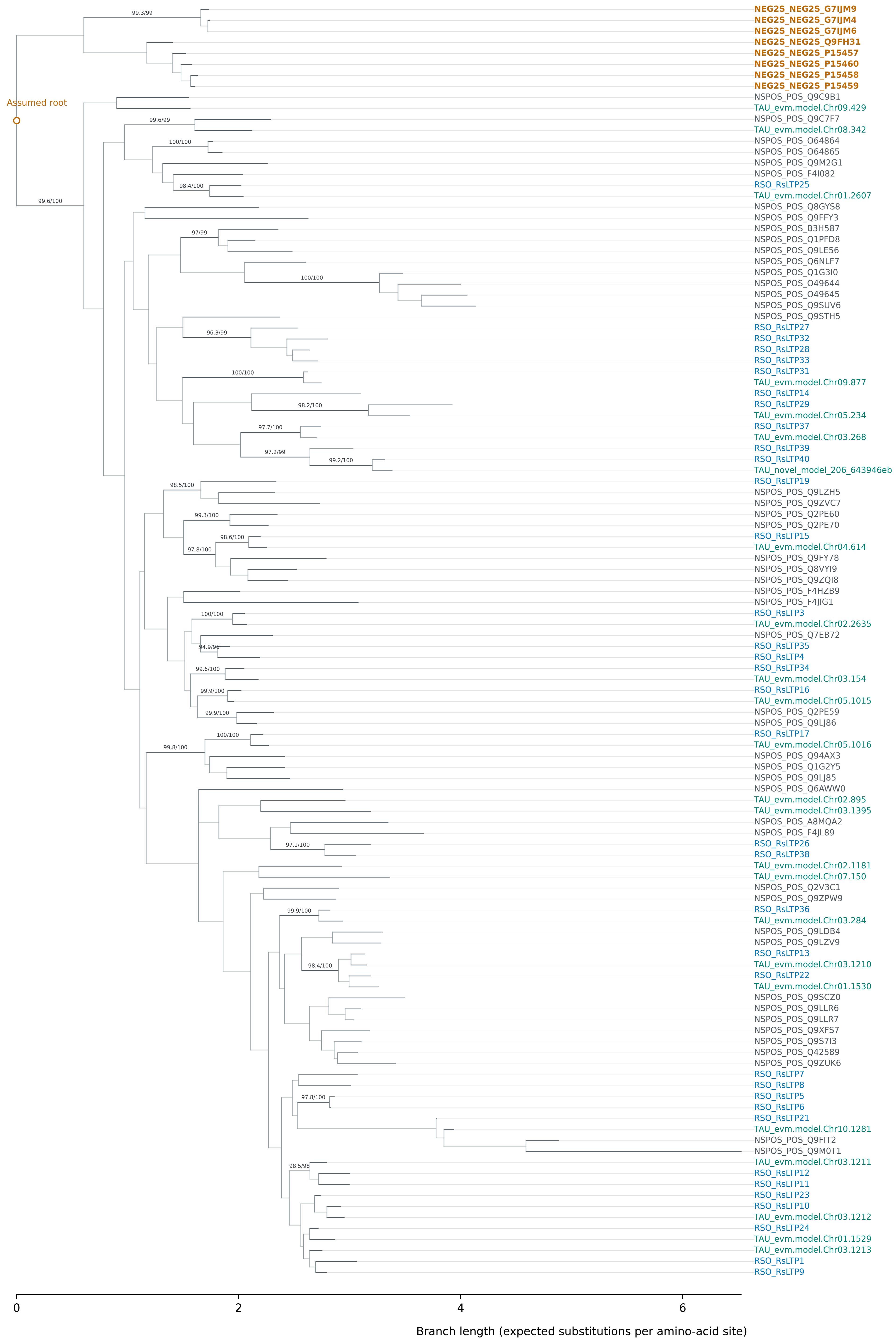

Orange: candidate outgroup; blue: R. soongarica; green: Tamarix; grey: other references.
Labels show SH-aLRT / UFBoot only when both are >=80 / >=95; all estimates remain in raw trees.
All tips and edge lengths retained. Root-edge length was split equally for representation, not time inference.
