# Supplementary material for: Genome-Wide Identification, Evolutionary Characterization, and Salt-Stress Expression Profiling of Antimicrobial Peptide Gene Families in the Xerophytic Recretohalophyte Reaumuria soongarica
Source: Genes (Basel). 2026 Sep 16;17(9):1126. doi: 10.3390/genes17091126 (PMC13606368; doi:10.3390/genes17091126)
Supplement: Supplementary file 1 [file genes-17-01126-s001.zip › Files/File_S2_outgroup_rooting_20260908.zip!/Figures/Rooting_sensitivity_summary.pdf]

Outgroup sensitivity: branch support and root stability are different questions

A Candidate-set separation across alignment strategies

	Full untrimmed	Full gappyout	Cys span untrimmed	Cys span gappyout
Defensin | 10 gymnosperms	**96.7/98**	No split	No split	No split
GASA | 3 Selaginella	No split	No split	No split	No split
nsLTP | all 8 2S	**99.6/100**	**97.8/100**	**96.7/100**	**97/100**
nsLTP | 3 Medicago 2S	Not tested	**100/100**	Not tested	**99.9/100**
nsLTP | 5 Arabidopsis 2S	Not tested	**100/100**	Not tested	**100/100**

Cells show SH-aLRT / UFBoot for the exact candidate-set split. These values are not root confidence.

B nsLTP: eight analyses produce six basal ingroup partitions

	R1	R2	R3	R4	R5	R6
All 8 | full / untrimmed		**R2**				
All 8 | full / gappyout						**R6**
All 8 | Cys span / untrimmed					**R5**	
All 8 | Cys span / gappyout					**R5**	
Medicago 3 | full / gappyout				**R4**		
Medicago 3 | Cys span / gappyout			**R3**			
Arabidopsis 5 | full / gappyout		**R2**				
Arabidopsis 5 | Cys span / gappyout	**R1**					

The same 108 ingroup taxa are compared in every row. R1-R6 are descriptive IDs, not ranked roots.

C Members on the smaller side of each basal partition

**R1**	Ref: Q9FY78	1 | 107 taxa
**R2**	Ref: Q9C9B1, Ta: Chr09.429	2 | 106 taxa
**R3**	Ta: Chr02.895	1 | 107 taxa
**R4**	Ref: Q7EB72, RsLTP35, RsLTP4	3 | 105 taxa
**R5**	RsLTP14	1 | 107 taxa
**R6**	Ref: Q9M0T1	1 | 107 taxa
