# Supplementary material for: Genome-Wide Identification, Evolutionary Characterization, and Salt-Stress Expression Profiling of Antimicrobial Peptide Gene Families in the Xerophytic Recretohalophyte Reaumuria soongarica
Source: Genes (Basel). 2026 Sep 16;17(9):1126. doi: 10.3390/genes17091126 (PMC13606368; doi:10.3390/genes17091126)
Supplement: Supplementary file 1 [file genes-17-01126-s001.zip › Files/File_S2_outgroup_rooting_20260908.zip!/外群定根_先看这一页.html]

外群定根：先看这一页


GENES 修订 · 2026 年 9 月 8 日 · 分层阅读

# 外群定根：先看这一页

近缘种比较提供了值得检验的同源序列。能否把它们当作外群，要看基因家族中的位置和复制关系。

本次已重新运行 16 次分析，并检查原有 12 棵树。下面按家族区分结果。

nsLTP

### 可以画出条件性定根树，但根位置不一致

候选：2S 蛋白。完成 8 次分析；8 次能分离候选外群，得到 6 种内群根部划分。

Snakin/GASA

### 未形成可统一分离的候选外群

候选：卷柏 GASA。完成 4 次分析；0 次能分离候选外群，得到 0 种内群根部划分。

Defensin

### 候选外群未在所有策略中成立

候选：裸子植物 defensin。完成 4 次分析；1 次能分离候选外群，得到 1 种内群根部划分。

**现有稳定分组分析仍有价值。**分组回答“哪些成员稳定聚在一起”；定根回答“从哪里开始分化”。根未解决时，仍可如实报告有支持的分组，保留未解决关系。

第一层：Tamarix 比较到底帮了什么？

归一化数量用于比较成员数量；双向最佳匹配用于筛选可能对应的拷贝；共线性用于核对基因所在区段的关系。它们不能直接给出整个家族的树根。

已有 28 对家族一致的最佳匹配，其中 **12 对在全部四种策略下形成高支持分支**。这是有用的同源关系证据。不过，在全部 12 棵原树中，所有 Tamarix 成员都不能被一条枝与其余成员整体分开。

因此可以保留并使用这项比较；目前不能把全部 Tamarix 序列直接指定为三个家族的统一外群。

第二层：用一个真实例子理解

RsLTP39、RsLTP40 和一条 Tamarix 序列在四种策略下都聚成稳定的三成员组。但组内最稳定的一对是 **RsLTP40 与 Tamarix**。

其余 150 条序列红砂 RsLTP39红砂 RsLTP40Tamarix99.6 / 100无箭头：未指定根；枝长未按比例绘制

连接与支持度来自原全长 + gappyout 树。Tamarix 序列为 novel\_model\_206\_643946eb；与 RsLTP40 对应 RBH\_0013。99.6/100 是分支支持度，不是树根正确率。

截出这三条序列后，软件确实能把 Tamarix 放在一侧、两个红砂成员放在另一侧。但这取决于人为指定的根，不能用来证明红砂成员的复制先后。

第三层：查看 16 次分析的具体结果

“序列/位点”为输入数量与比对长度；“分支支持”为 SH-aLRT/UFBoot；“内群两侧数量”描述条件性根部的划分。相同数量不保证成员相同，精确成员对应保存在 TSV 表中。首末 C 区域沿用项目定义，不等同于经实验确认的成熟肽。

| 候选集合 | 策略 | 序列 / 位点 | 外群分支支持 | 内群两侧数量 |
| --- | --- | --- | --- | --- |
| nsLTP：拟南芥 5 条 2S | 首末 C 区域 / gappyout 修剪 | 113 / 106 | 100/100 | 107 / 1 |
| nsLTP：拟南芥 5 条 2S | 全长 / gappyout 修剪 | 113 / 92 | 100/100 | 106 / 2 |
| nsLTP：Medicago 3 条 2S | 首末 C 区域 / gappyout 修剪 | 111 / 106 | 99.9/100 | 107 / 1 |
| nsLTP：Medicago 3 条 2S | 全长 / gappyout 修剪 | 111 / 95 | 100/100 | 105 / 3 |
| nsLTP：8 条 2S | 首末 C 区域 / gappyout 修剪 | 116 / 74 | 97/100 | 107 / 1 |
| nsLTP：8 条 2S | 首末 C 区域 / 未修剪 | 116 / 328 | 96.7/100 | 107 / 1 |
| nsLTP：8 条 2S | 全长 / gappyout 修剪 | 116 / 93 | 97.8/100 | 107 / 1 |
| nsLTP：8 条 2S | 全长 / 未修剪 | 116 / 493 | 99.6/100 | 106 / 2 |
| GASA：3 条卷柏序列 | 首末 C 区域 / gappyout 修剪 | 46 / 58 | 没有对应分支 | — |
| GASA：3 条卷柏序列 | 首末 C 区域 / 未修剪 | 46 / 167 | 没有对应分支 | — |
| GASA：3 条卷柏序列 | 全长 / gappyout 修剪 | 46 / 115 | 没有对应分支 | — |
| GASA：3 条卷柏序列 | 全长 / 未修剪 | 46 / 382 | 没有对应分支 | — |
| Defensin：10 条裸子植物序列 | 首末 C 区域 / gappyout 修剪 | 112 / 43 | 没有对应分支 | — |
| Defensin：10 条裸子植物序列 | 首末 C 区域 / 未修剪 | 112 / 85 | 没有对应分支 | — |
| Defensin：10 条裸子植物序列 | 全长 / gappyout 修剪 | 112 / 45 | 没有对应分支 | — |
| Defensin：10 条裸子植物序列 | 全长 / 未修剪 | 112 / 203 | 96.7/98 | 101 / 1 |

所有新结果保存在独立的 rooting\_assessment\_20260908 目录；原论文图件和原始树未覆盖。实验设计 · 完整指标 · 根位置成员表 · IQ-TREE 定根说明
