# Supplementary material for: Genome-Wide Identification, Evolutionary Characterization, and Salt-Stress Expression Profiling of Antimicrobial Peptide Gene Families in the Xerophytic Recretohalophyte Reaumuria soongarica
Source: Genes (Basel). 2026 Sep 16;17(9):1126. doi: 10.3390/genes17091126 (PMC13606368; doi:10.3390/genes17091126)
Supplement: Supplementary file 1 [file genes-17-01126-s001.zip › Files/File_S3_comparative_audit_20260909.zip!/figure8/Figure8_comparative_synced_20260909.pdf]

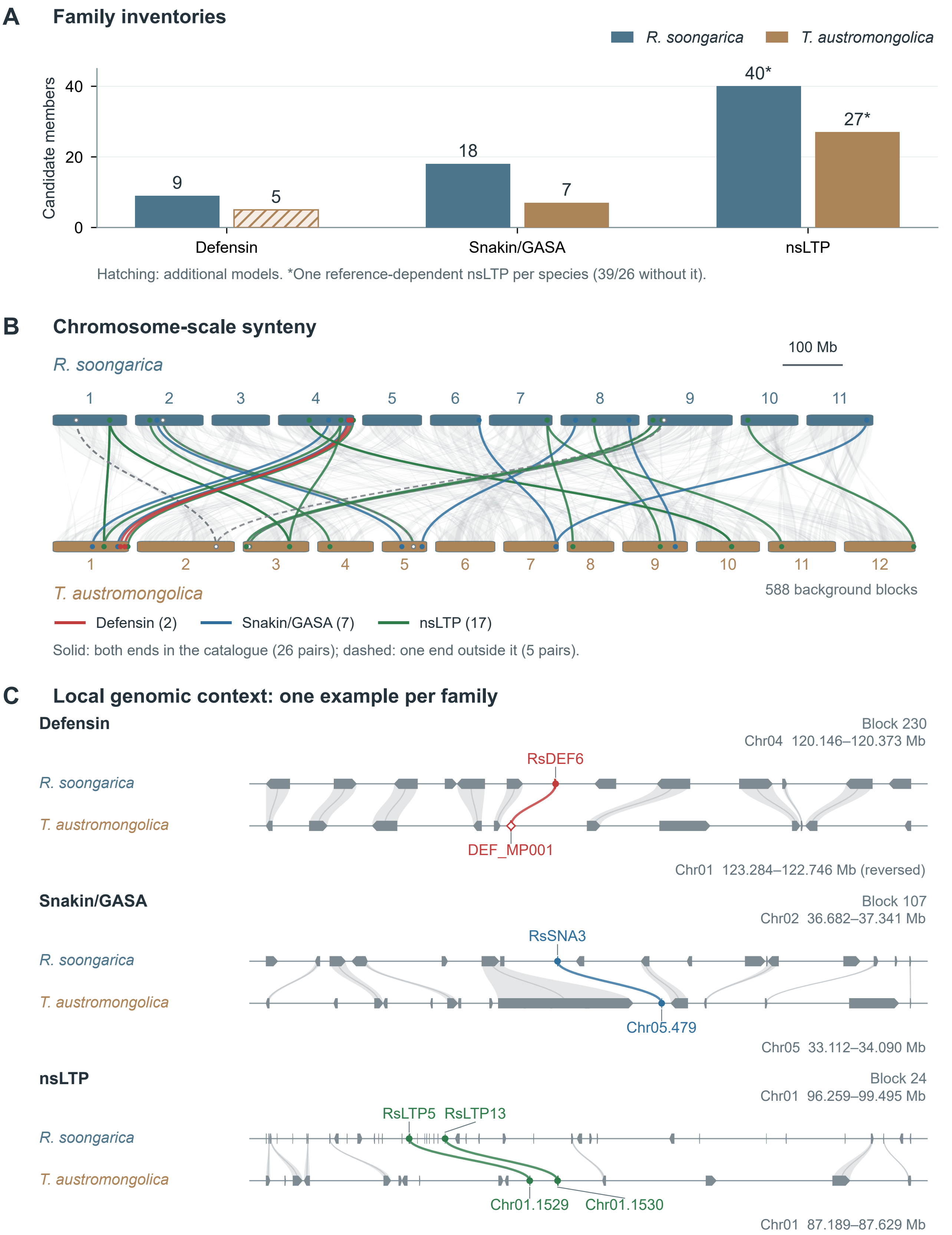

Dots: focal gene positions; open diamonds: additional homology models. Arrows show orientation.
Local rows are scaled independently; reversed coordinate directions are labelled.
