# Supplementary material for: Genome-Wide Identification, Evolutionary Characterization, and Salt-Stress Expression Profiling of Antimicrobial Peptide Gene Families in the Xerophytic Recretohalophyte Reaumuria soongarica
Source: Genes (Basel). 2026 Sep 16;17(9):1126. doi: 10.3390/genes17091126 (PMC13606368; doi:10.3390/genes17091126)
Supplement: Supplementary file 1 [file genes-17-01126-s001.zip › Files/File_S3_comparative_audit_20260909.zip!/figure8/全部31对_局部共线性核对.pdf]

AMP-involving synteny: all candidate-pair contexts

Shared comparison cohort · corrected reader · independent genomic scales per row

01 nsLTP | Block 24

Block 24

Chr01 96.260–99.456 Mb

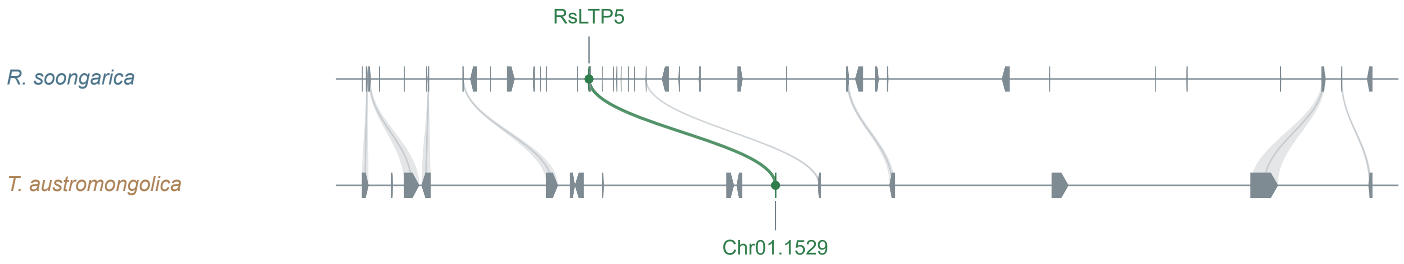

Chr01 87.189–87.629 Mb

Both ends in current catalogue

RSO__gmmChr01G010000.1 / TAU__evm.model.Chr01.1529

02 nsLTP | Block 24

Block 24

Chr01 96.389–99.492 Mb

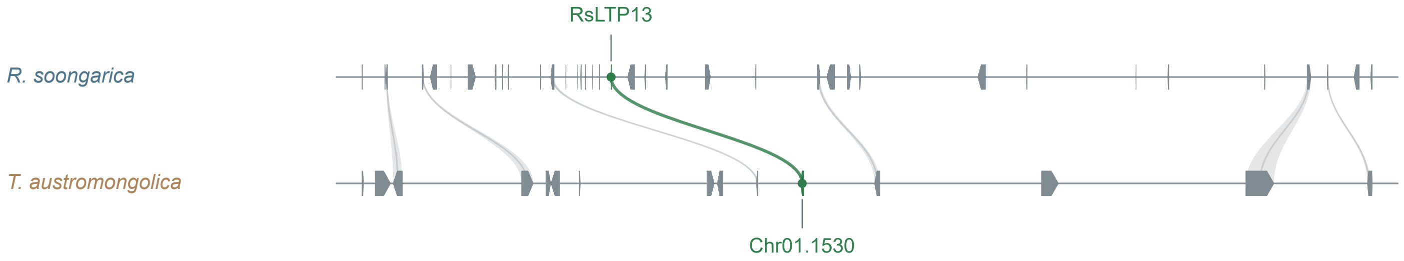

Chr01 87.201–87.629 Mb

Both ends in current catalogue

RSO__gmmChr01G010080.1 / TAU__evm.model.Chr01.1530

03 nsLTP | Block 42

Block 42

Chr01 39.165–40.093 Mb

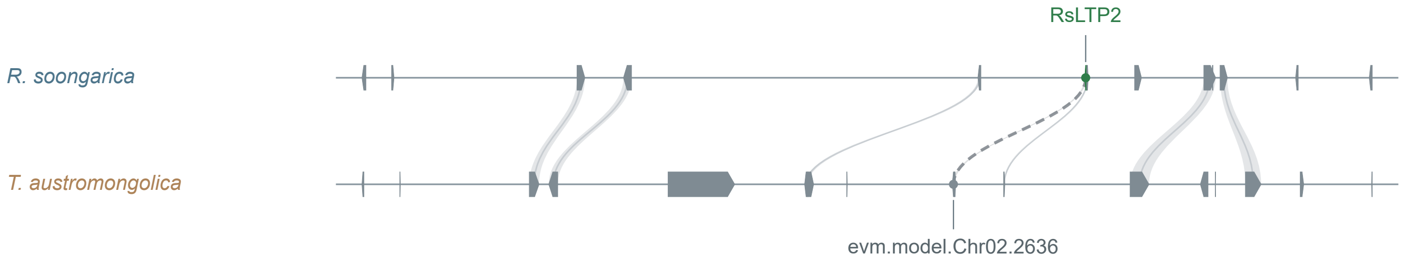

Chr02 135.623–134.746 Mb (reversed)

One endpoint is outside current AMP catalogue

RSO__gmmChr01G005030.1 / TAU__evm.model.Chr02.2636

04 nsLTP | Block 42

Block 42

Chr01 39.191–40.122 Mb

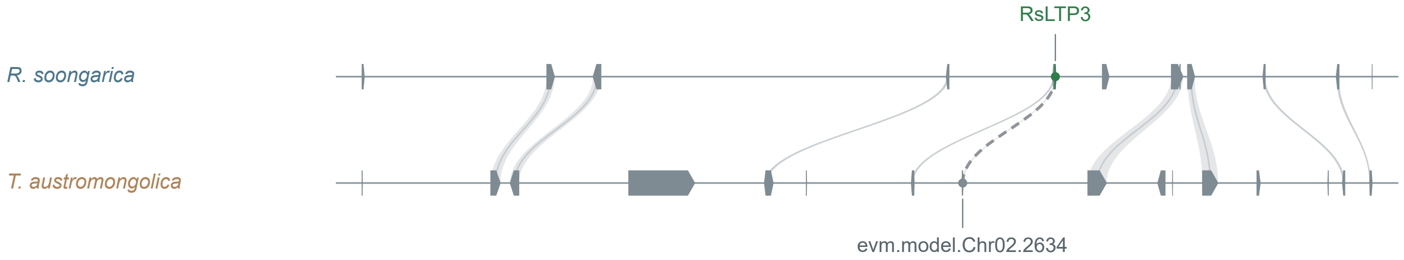

Chr02 135.591–134.710 Mb (reversed)

One endpoint is outside current AMP catalogue

RSO__STRG.2250.1.p1 / TAU__evm.model.Chr02.2634

Grey genes and links provide local context; highlighted genes are the focal pair. Reversed rows are labelled.

AMP-involving synteny: all candidate-pair contexts

Shared comparison cohort · corrected reader · independent genomic scales per row

05 nsLTP | Block 51

Block 51

Chr01 96.701–97.638 Mb

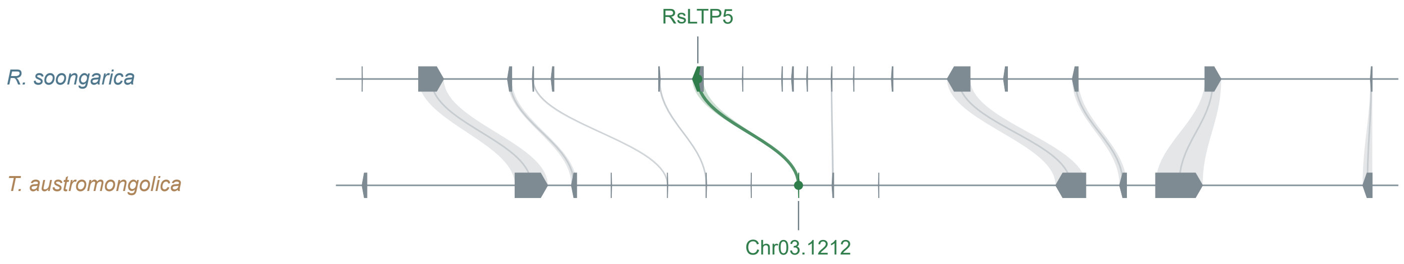

Chr03 79.840–79.060 Mb (reversed)

Both ends in current catalogue

RSO__gmmChr01G010000.1 / TAU__evm.model.Chr03.1212

06 nsLTP | Block 51

Block 51

Chr01 96.747–97.829 Mb

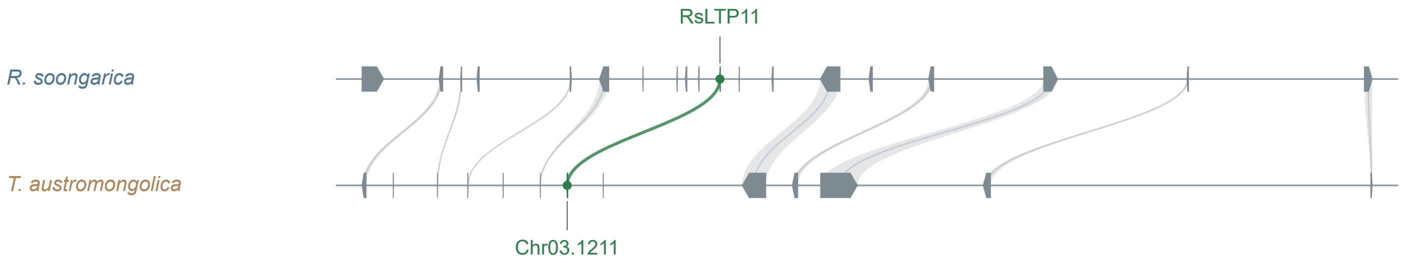

Chr03 79.691–78.698 Mb (reversed)

Both ends in current catalogue

RSO__gmmChr01G010060.1 / TAU__evm.model.Chr03.1211

07 nsLTP | Block 102

Block 102

Chr02 24.519–24.911 Mb

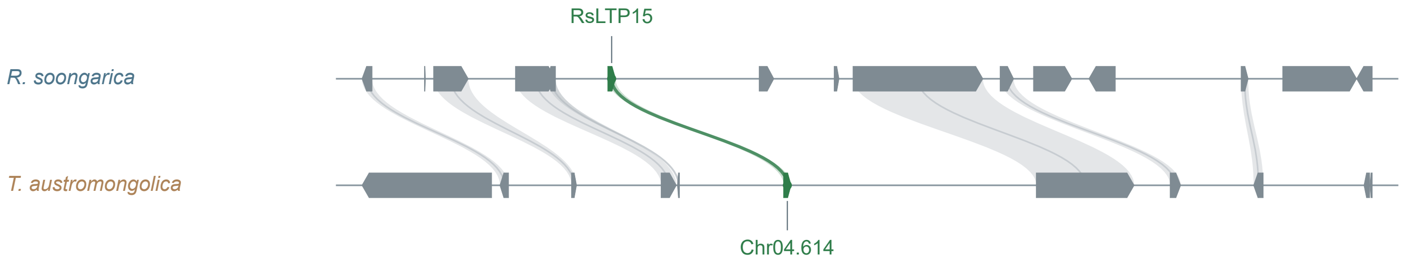

Chr04 21.221–20.752 Mb (reversed)

Both ends in current catalogue

RSO__gmmChr02G007880.1 / TAU__evm.model.Chr04.614

08 nsLTP | Block 106

Block 106

Chr02 46.765–47.467 Mb

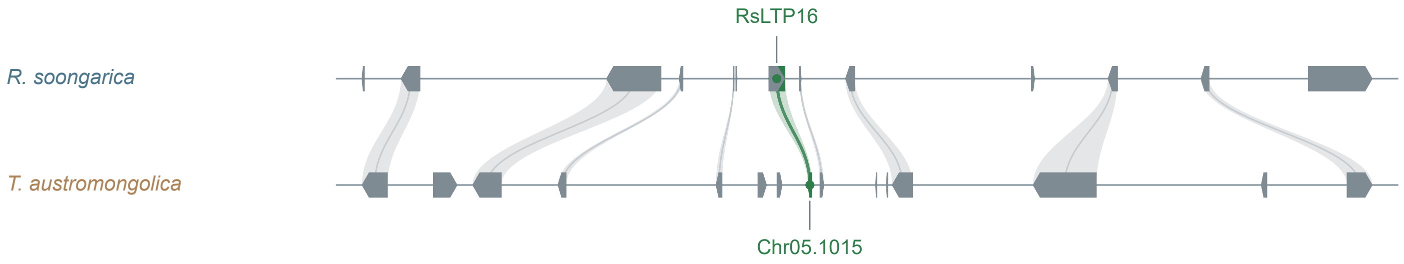

Chr05 53.535–53.757 Mb

Both ends in current catalogue

RSO__gmmChr02G012770.1 / TAU__evm.model.Chr05.1015

Grey genes and links provide local context; highlighted genes are the focal pair. Reversed rows are labelled.

AMP-involving synteny: all candidate-pair contexts

Shared comparison cohort · corrected reader · independent genomic scales per row

09 nsLTP | Block 106 Block 106

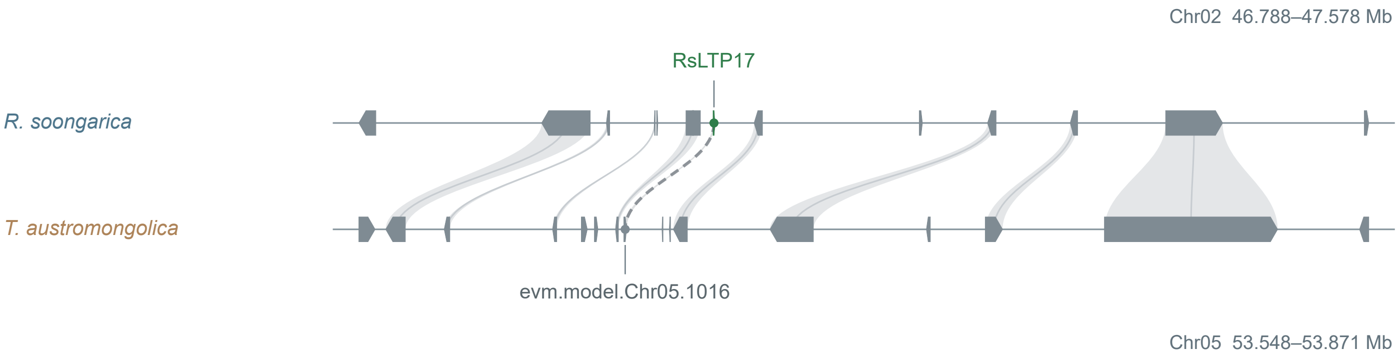

One endpoint is outside current AMP catalogue

RSO__STRG.4001.1.p1 / TAU__evm.model.Chr05.1016

10 Snakin/GASA | Block 107 Block 107

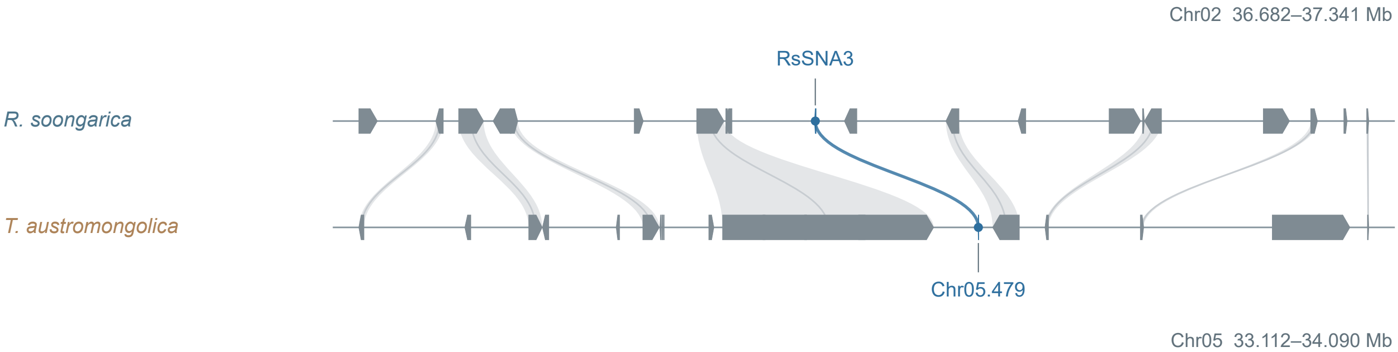

Both ends in current catalogue

RSO__STRG.12968.1.p1 / TAU__evm.model.Chr05.479

11 Snakin/GASA | Block 221 Block 221

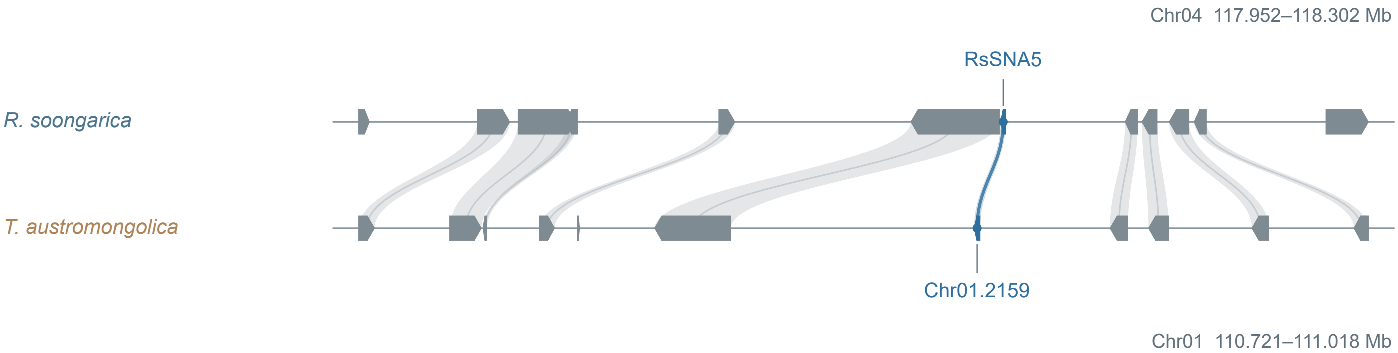

Both ends in current catalogue

RSO__MSTRG.7707.1.p1 / TAU__evm.model.Chr01.2159

12 nsLTP | Block 222 Block 222

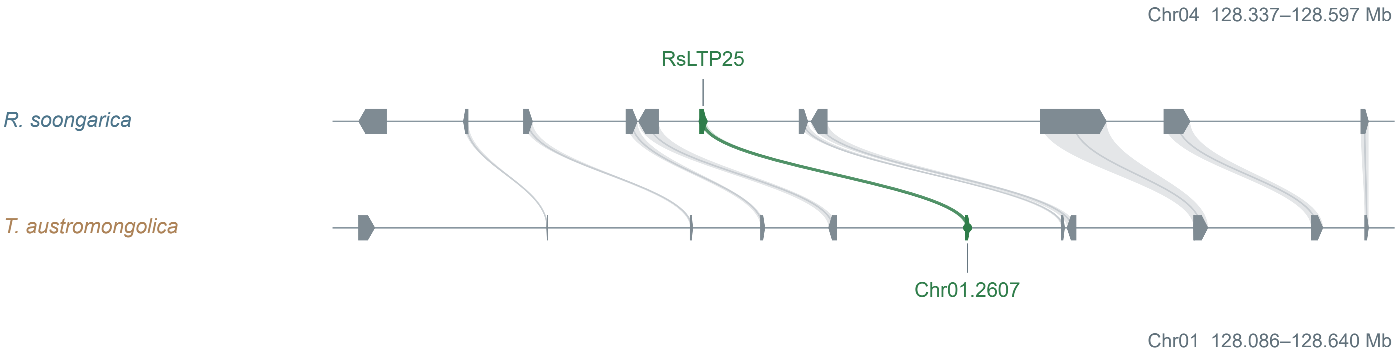

Both ends in current catalogue

RSO__STRG.28752.1.p1 / TAU__evm.model.Chr01.2607

Grey genes and links provide local context; highlighted genes are the focal pair. Reversed rows are labelled.

AMP-involving synteny: all candidate-pair contexts

Shared comparison cohort · corrected reader · independent genomic scales per row

13 Snakin/GASA | Block 223

Block 223

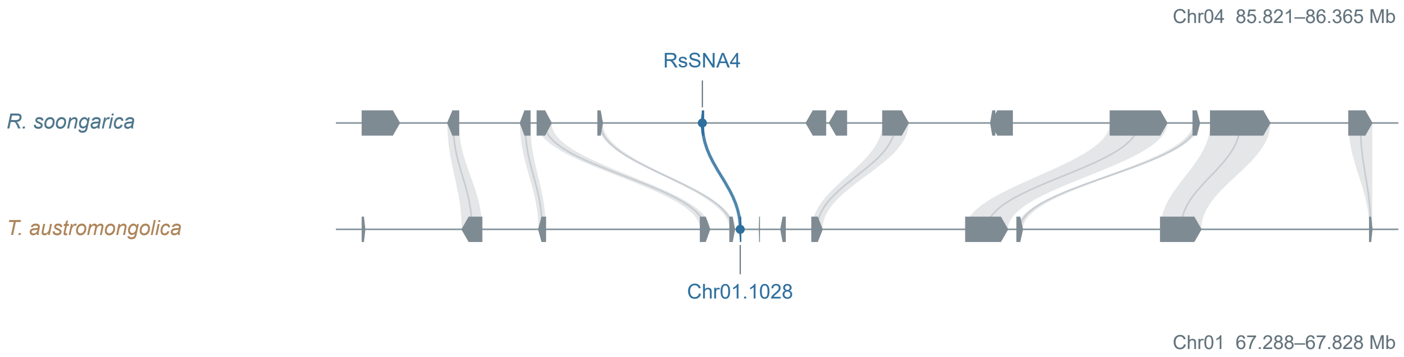

Both ends in current catalogue

RSO__STRG.26736.1.p1 / TAU__evm.model.Chr01.1028

14 nsLTP | Block 229

Block 229

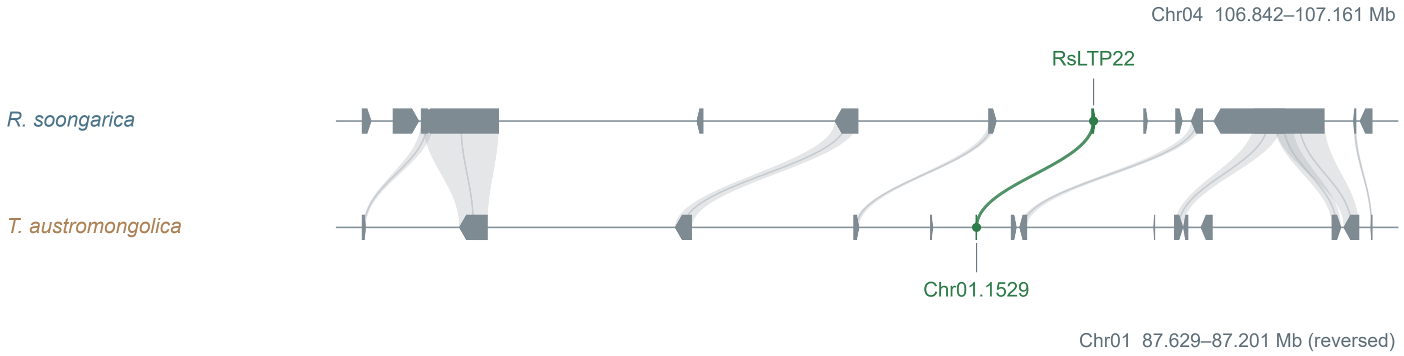

Both ends in current catalogue

RSO__gmmChr04G014500.2 / TAU__evm.model.Chr01.1529

15 Defensin | Block 230

Block 230

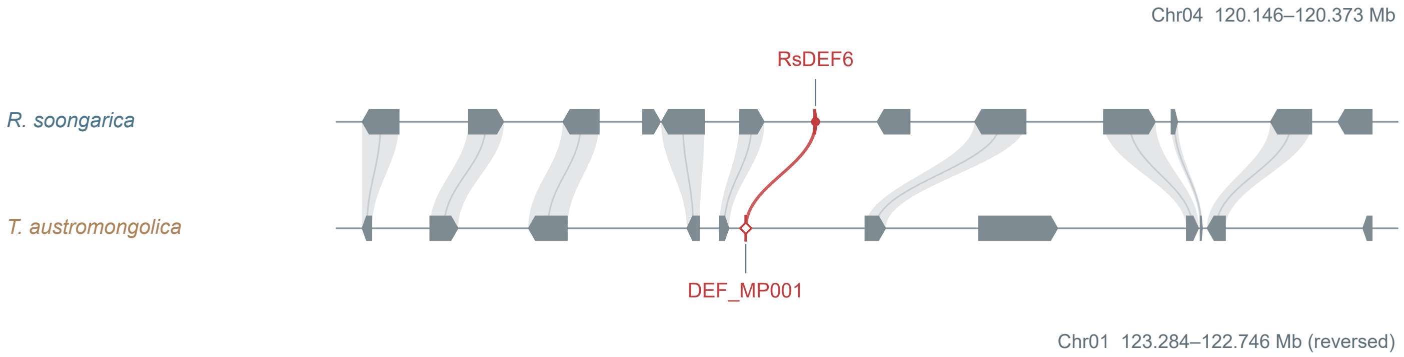

Both ends in current catalogue

RSO__gmmChr04G018870.1 / TAU__TAU_DEF_MP001

16 Defensin | Block 230

Block 230

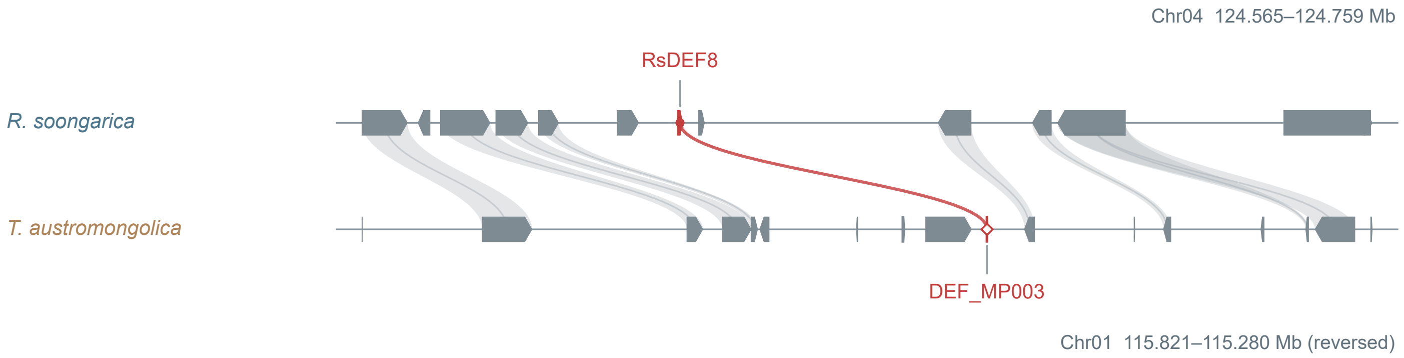

Both ends in current catalogue

RSO__gmmChr04G020400.1 / TAU__TAU_DEF_MP003

Grey genes and links provide local context; highlighted genes are the focal pair. Reversed rows are labelled.

AMP-involving synteny: all candidate-pair contexts

Shared comparison cohort · corrected reader · independent genomic scales per row

17 nsLTP | Block 239 Block 239

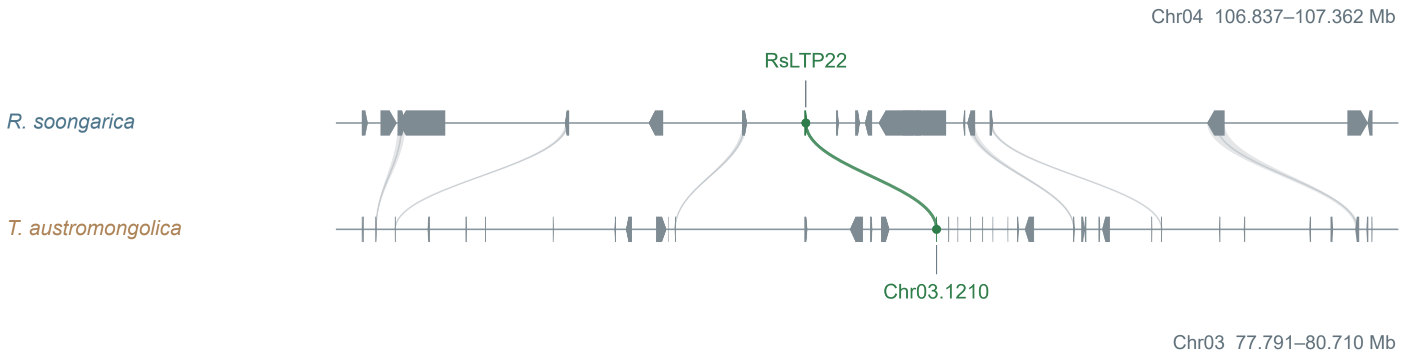

Both ends in current catalogue

RSO__gmmChr04G014500.2 / TAU__evm.model.Chr03.1210

18 nsLTP | Block 283 Block 283

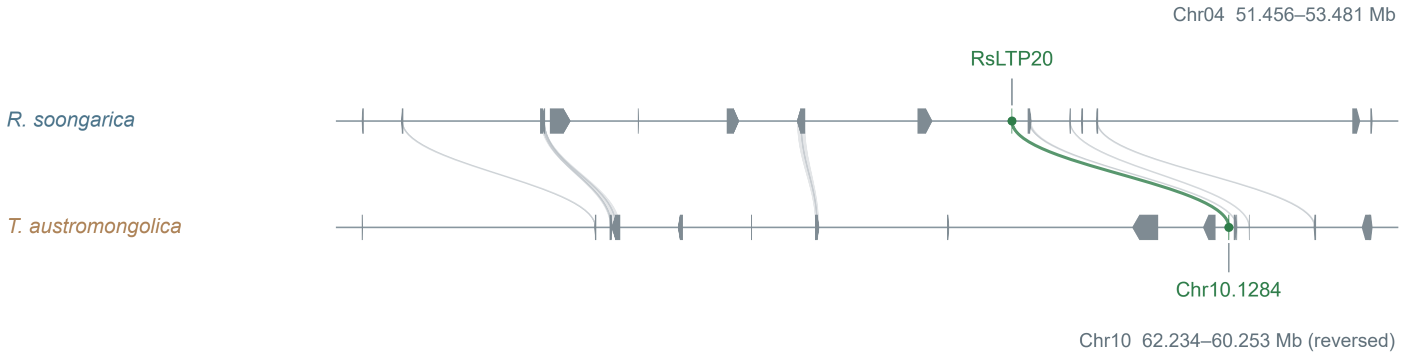

Both ends in current catalogue

RSO__gmmChr04G004380.1 / TAU__evm.model.Chr10.1284

19 nsLTP | Block 283 Block 283

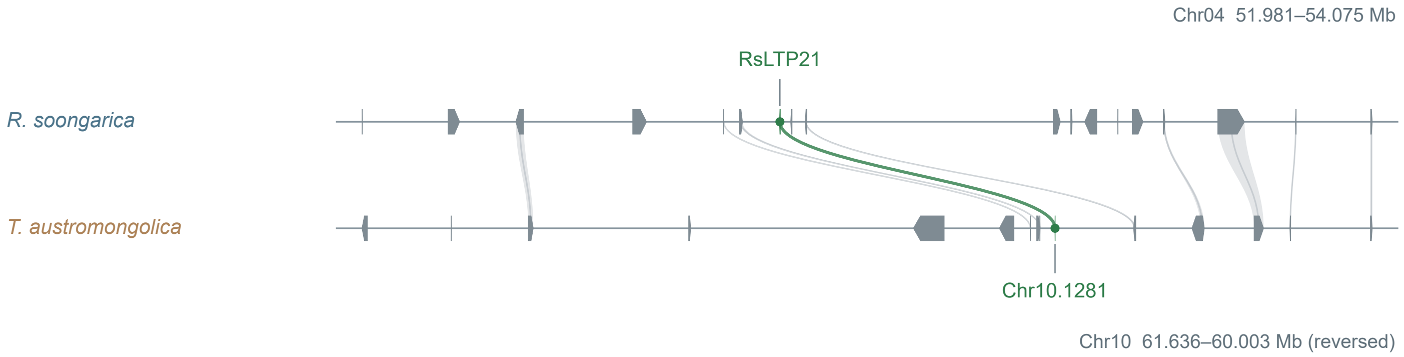

Both ends in current catalogue

RSO__gmmChr04G004390.1 / TAU__evm.model.Chr10.1281

20 Snakin/GASA | Block 406 Block 406

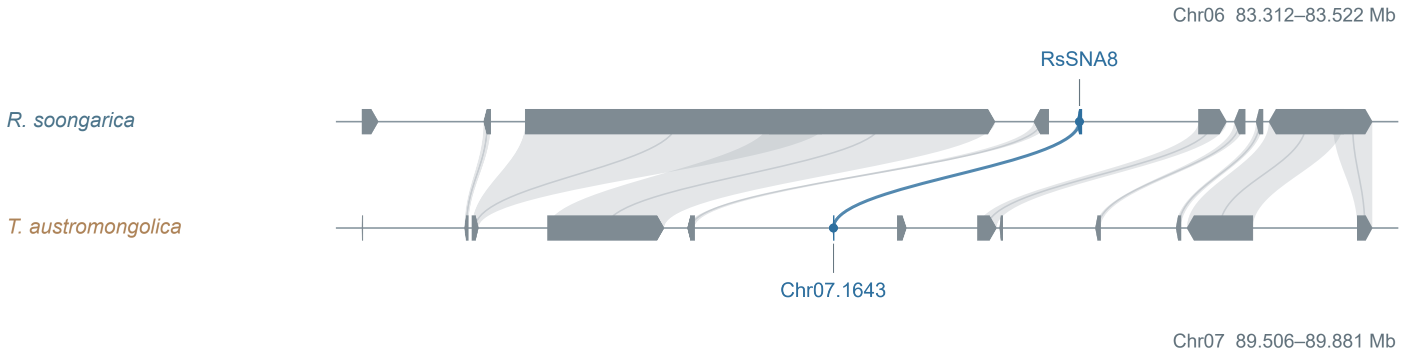

Both ends in current catalogue

RSO__STRG.16502.1.p1 / TAU__evm.model.Chr07.1643

Grey genes and links provide local context; highlighted genes are the focal pair. Reversed rows are labelled.

AMP-involving synteny: all candidate-pair contexts

Shared comparison cohort · corrected reader · independent genomic scales per row

21 nsLTP | Block 468

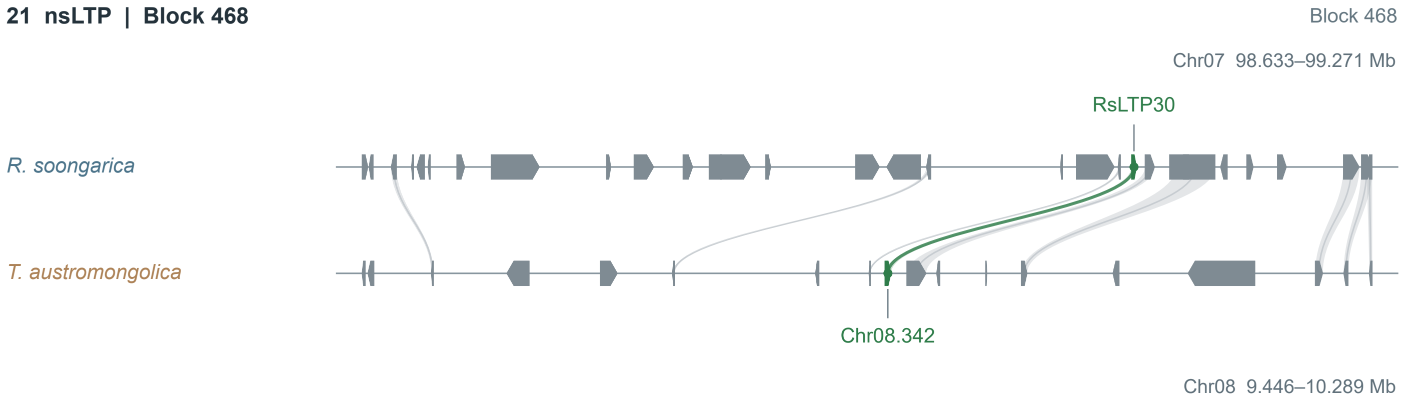

Both ends in current catalogue

RSO__STRG.19776.1.p1 / TAU__evm.model.Chr08.342

22 nsLTP | Block 489

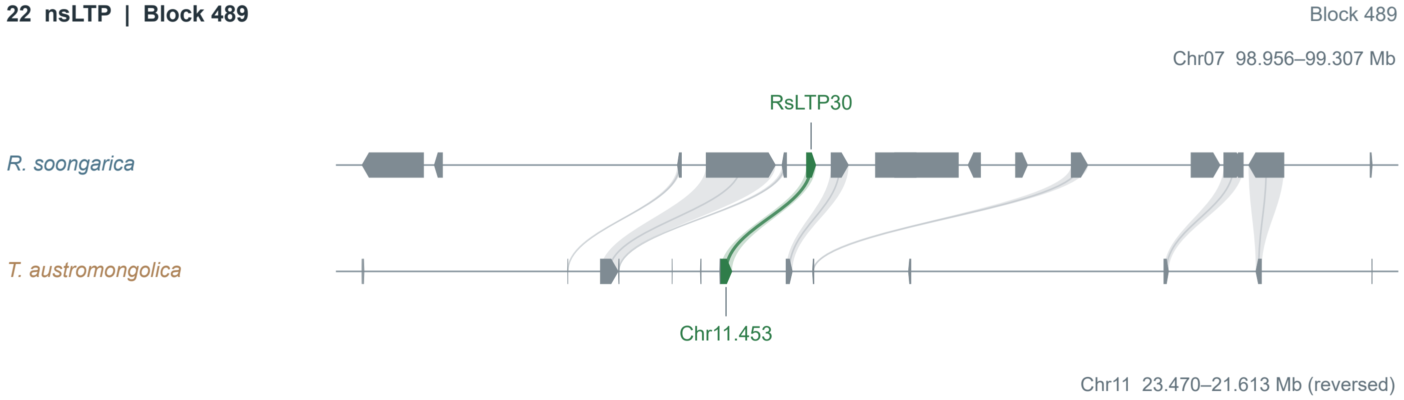

Both ends in current catalogue

RSO__STRG.19776.1.p1 / TAU__evm.model.Chr11.453

23 Snakin/GASA | Block 516

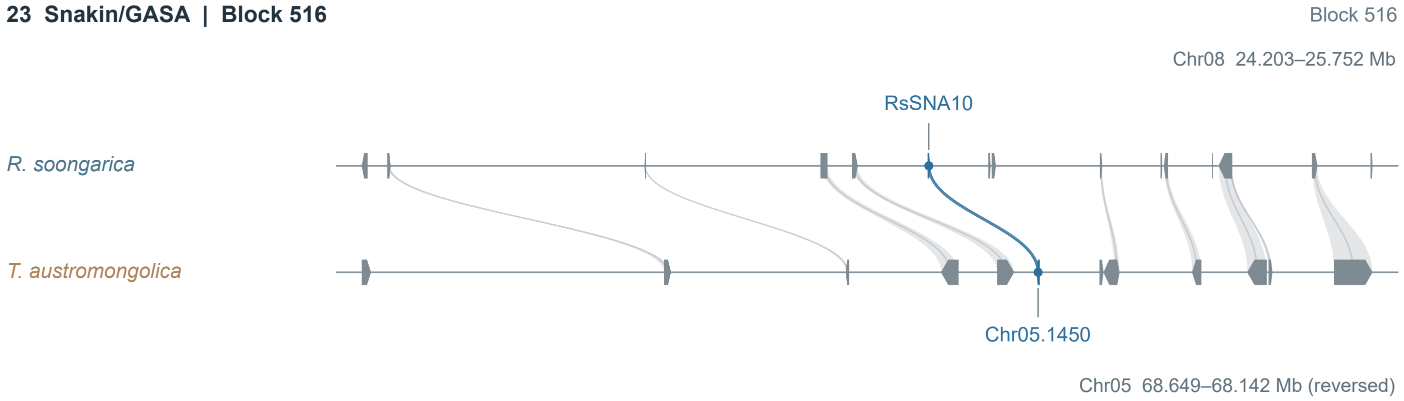

Both ends in current catalogue

RSO__gmmChr08G004090.1 / TAU__evm.model.Chr05.1450

24 Snakin/GASA | Block 529

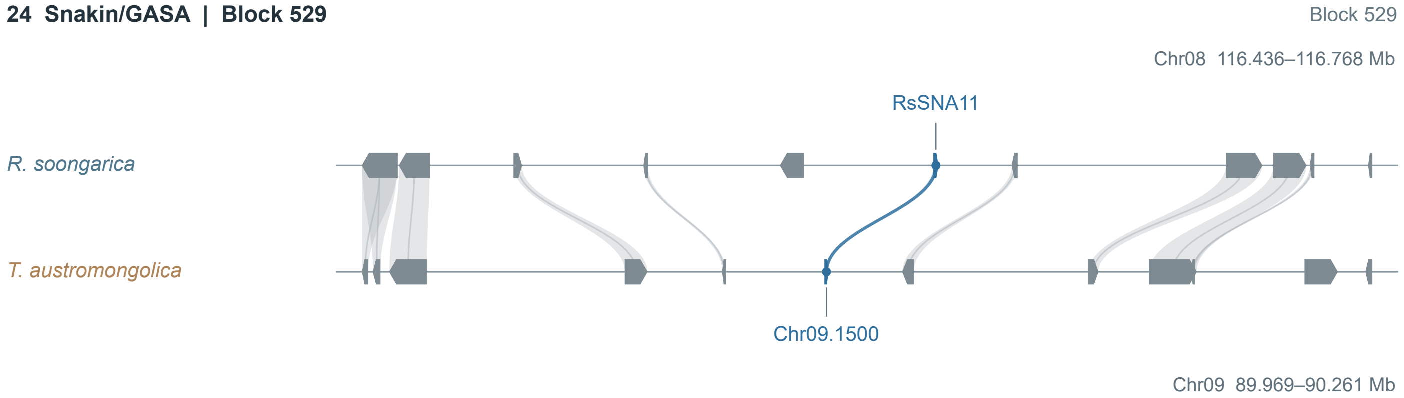

Both ends in current catalogue

RSO__gmmChr08G014630.1 / TAU__evm.model.Chr09.1500

Grey genes and links provide local context; highlighted genes are the focal pair. Reversed rows are labelled.

AMP-involving synteny: all candidate-pair contexts

Shared comparison cohort · corrected reader · independent genomic scales per row

25 nsLTP | Block 548

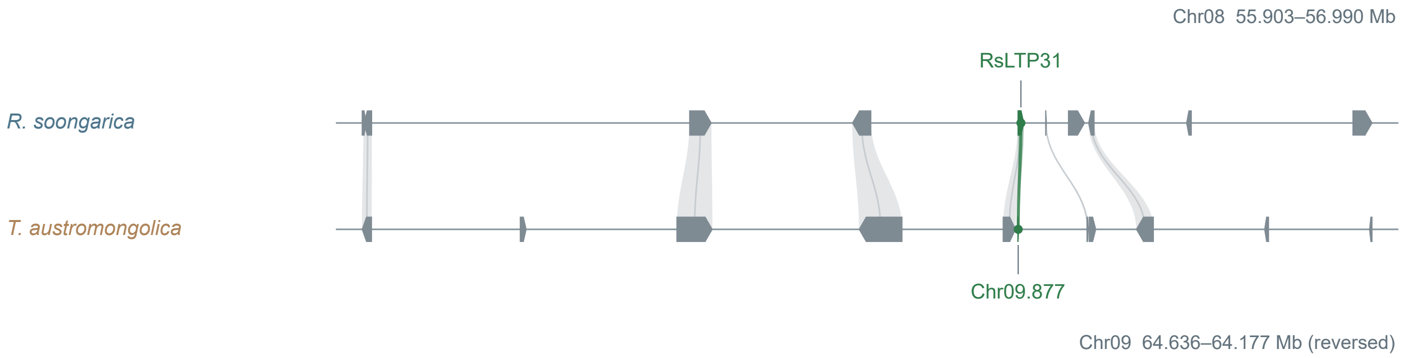

Both ends in current catalogue

RSO__gmmChr08G007020.1 / TAU__evm.model.Chr09.877

26 nsLTP | Block 585

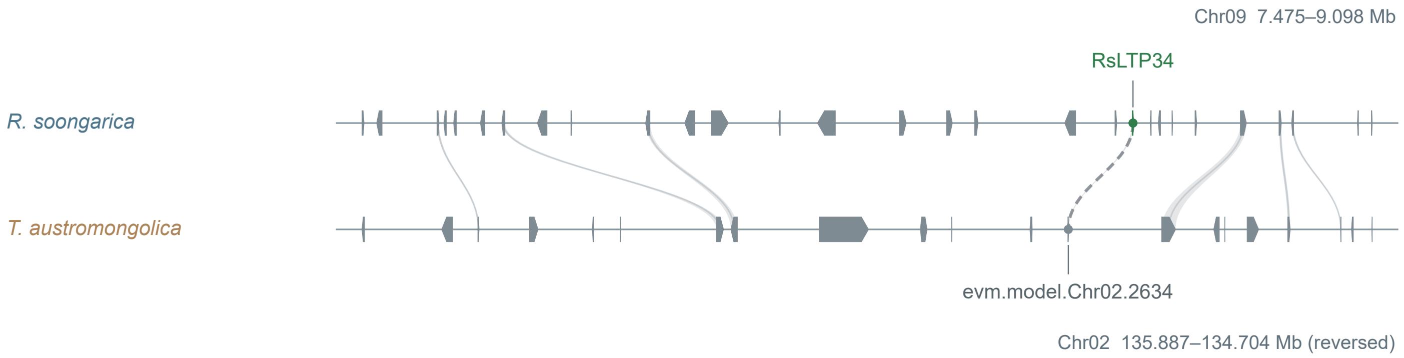

One endpoint is outside current AMP catalogue

RSO__STRG.16875.1.p1 / TAU__evm.model.Chr02.2634

27 nsLTP | Block 594

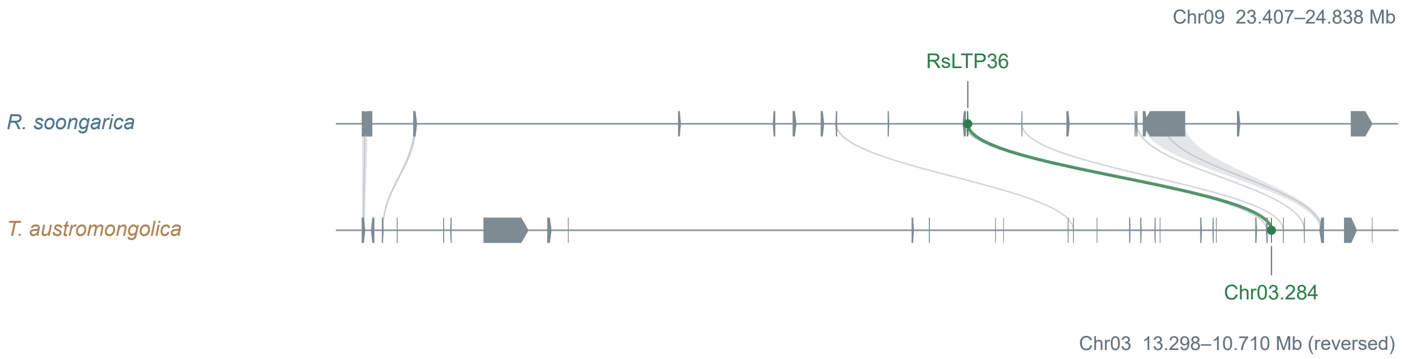

Both ends in current catalogue

RSO__gmmChr09G002740.1 / TAU__evm.model.Chr03.284

28 nsLTP | Block 594

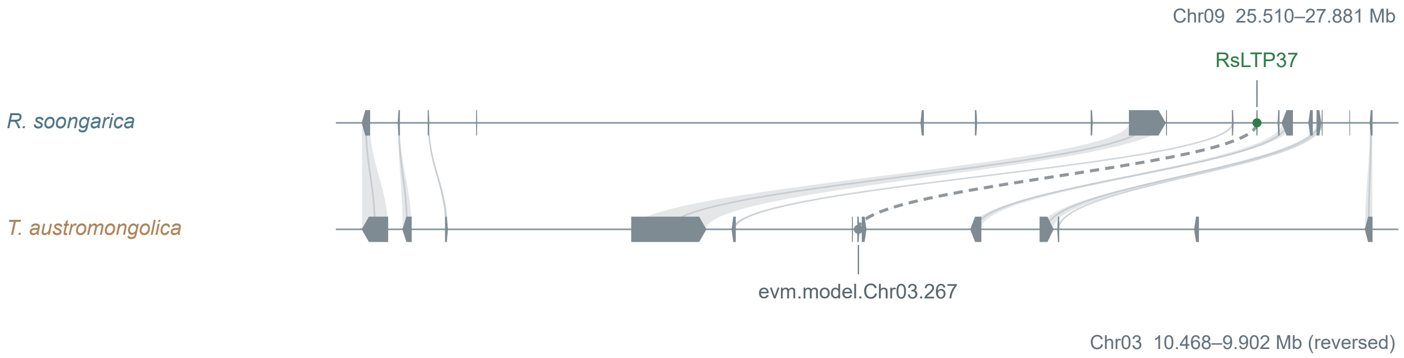

One endpoint is outside current AMP catalogue

RSO__STRG.16931.1.p1 / TAU__evm.model.Chr03.267

Grey genes and links provide local context; highlighted genes are the focal pair. Reversed rows are labelled.

AMP-involving synteny: all candidate-pair contexts

Shared comparison cohort · corrected reader · independent genomic scales per row

29 nsLTP | Block 595

Block 595

Chr09 8.189–9.081 Mb

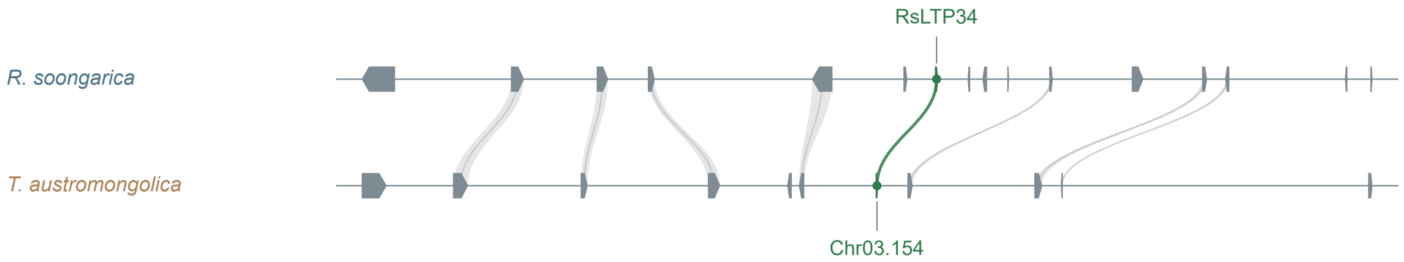

Chr03 5.496–5.042 Mb (reversed)

Both ends in current catalogue

RSO__STRG.16875.1.p1 / TAU__evm.model.Chr03.154

30 nsLTP | Block 674

Block 674

Chr10 11.181–11.916 Mb

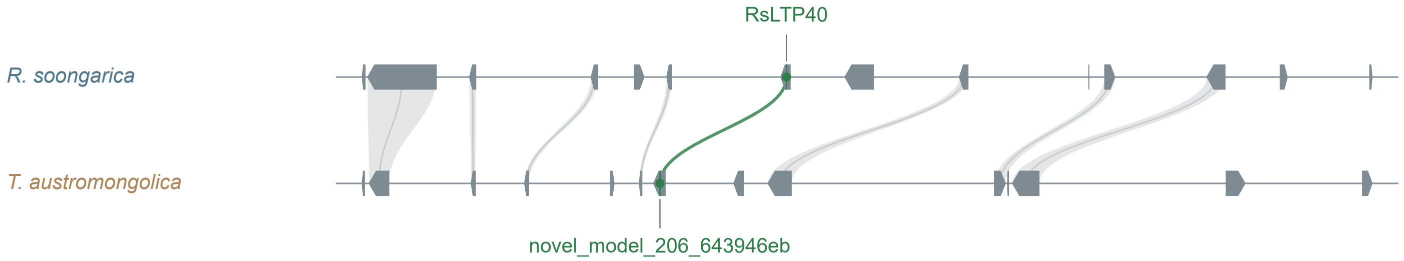

Chr12 119.045–118.391 Mb (reversed)

Both ends in current catalogue

RSO__STRG.29675.1.p1 / TAU__novel_model_206_643946eb

31 Snakin/GASA | Block 693

Block 693

Chr11 102.468–103.828 Mb

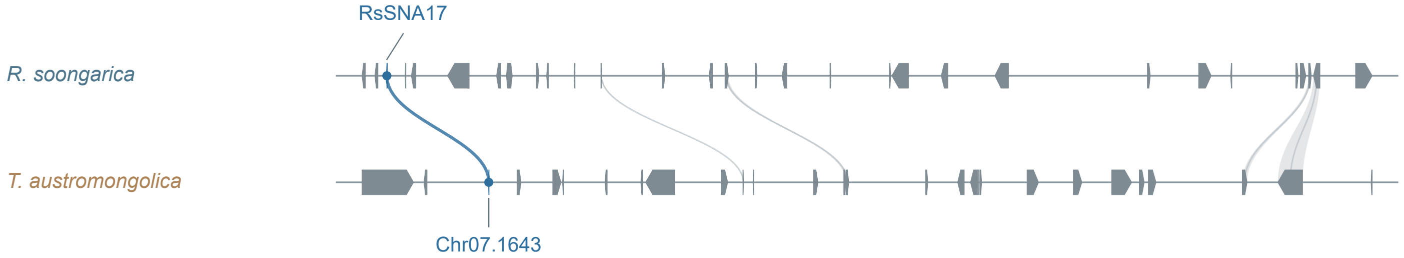

Chr07 89.560–90.407 Mb

Both ends in current catalogue

RSO__STRG.31259.1.p2 / TAU__evm.model.Chr07.1643

Grey genes and links provide local context; highlighted genes are the focal pair. Reversed rows are labelled.
