# Supplementary material for: Genome-Wide Identification, Evolutionary Characterization, and Salt-Stress Expression Profiling of Antimicrobial Peptide Gene Families in the Xerophytic Recretohalophyte Reaumuria soongarica
Source: Genes (Basel). 2026 Sep 16;17(9):1126. doi: 10.3390/genes17091126 (PMC13606368; doi:10.3390/genes17091126)
Supplement: Supplementary file 1 [file genes-17-01126-s001.zip › Files/File_S7_Tchinensis_expression_context_20260909.zip!/跨物种表达_实施_20260909/compute/verified_results/de_results/DESeq2_QC.pdf]

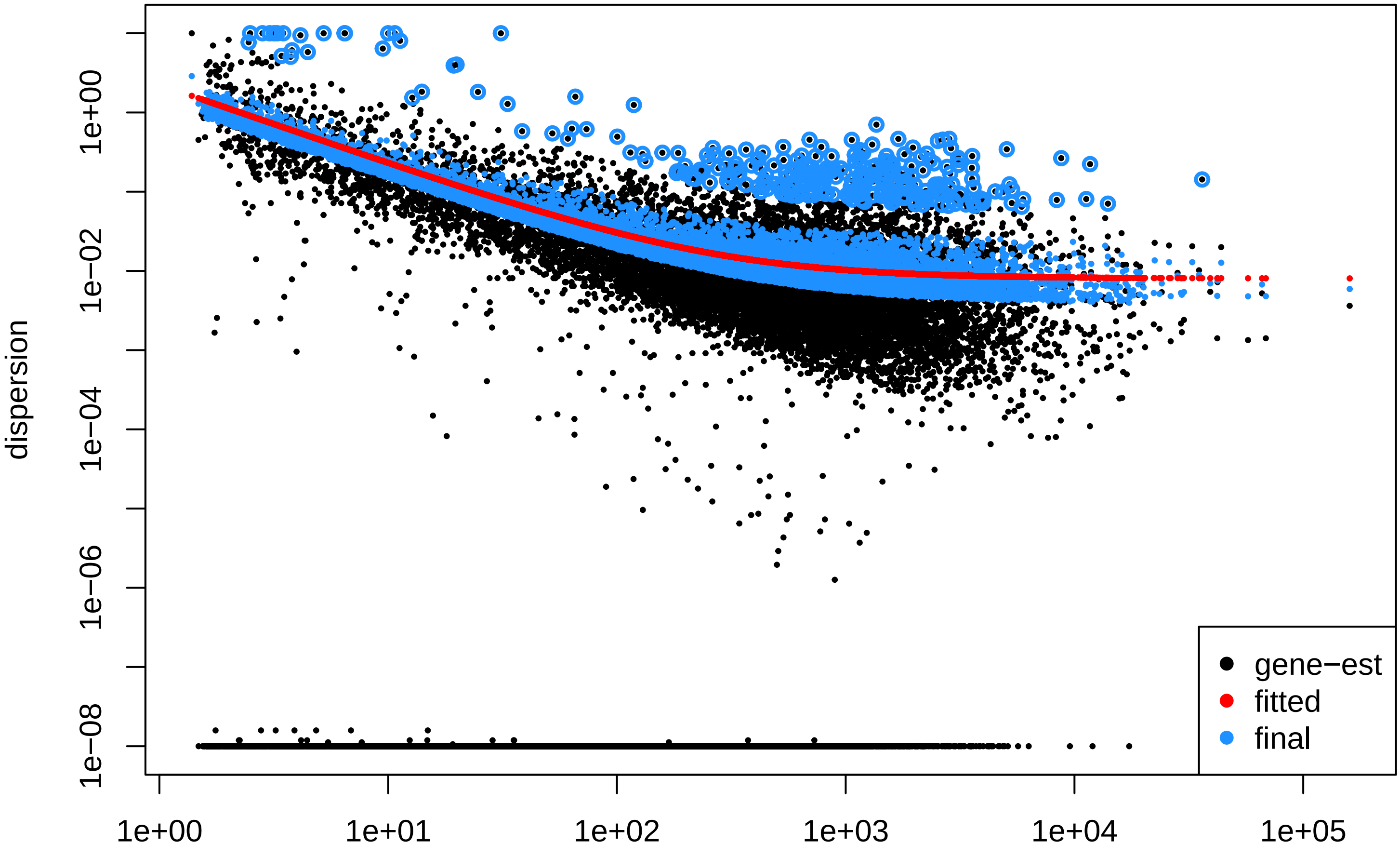
dispersion
1e+00
1e−02
1e−04
1e−06
1e−08
1e+00
1e+01
1e+02
1e+03
1e+04
1e+05
mean of normalized counts
gene−est
fitted
final

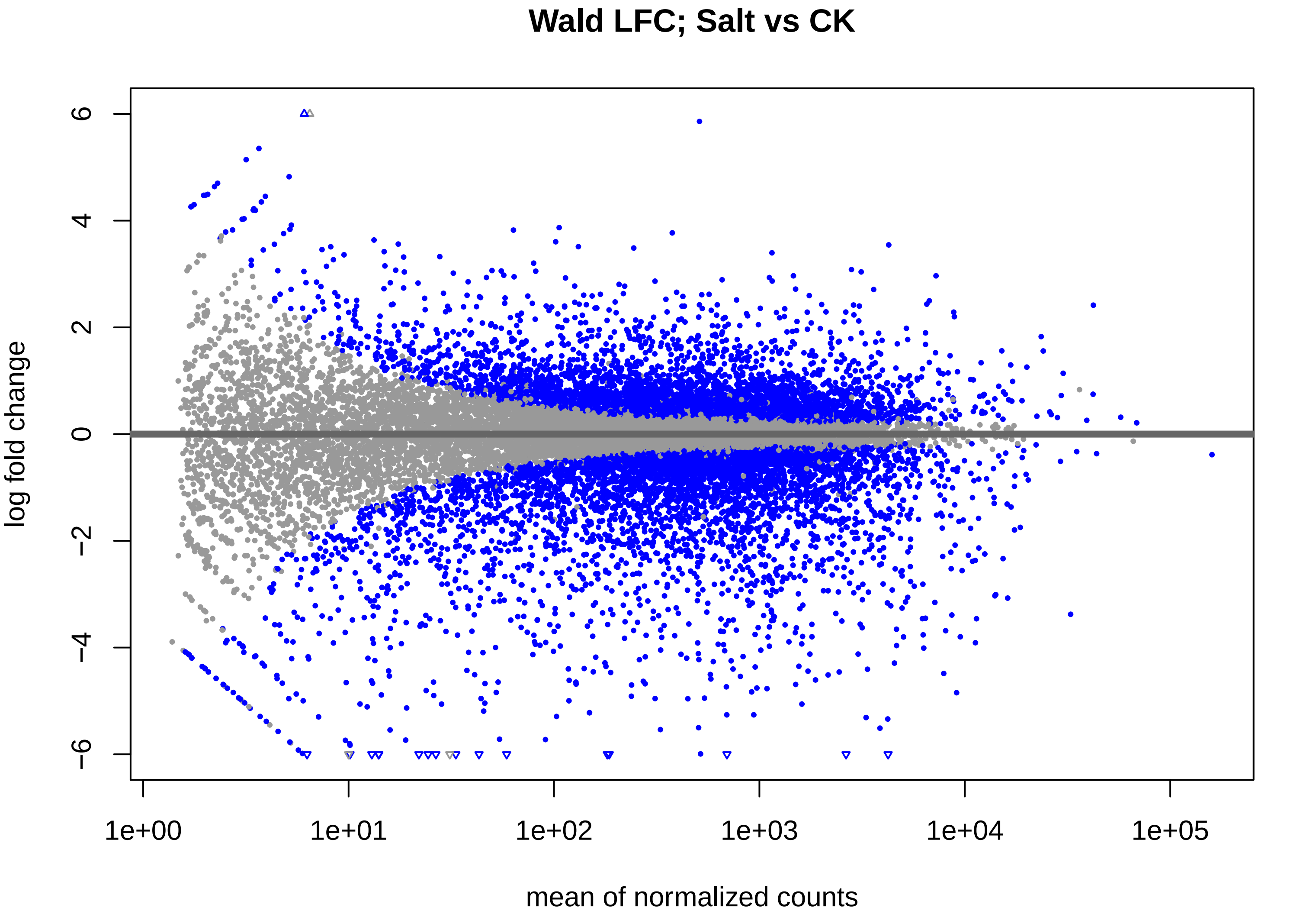

Wald LFC; Salt vs CK
log fold change
6
4
2
0
−2
−4
−6
mean of normalized counts
1e+00
1e+01
1e+02
1e+03
1e+04
1e+05

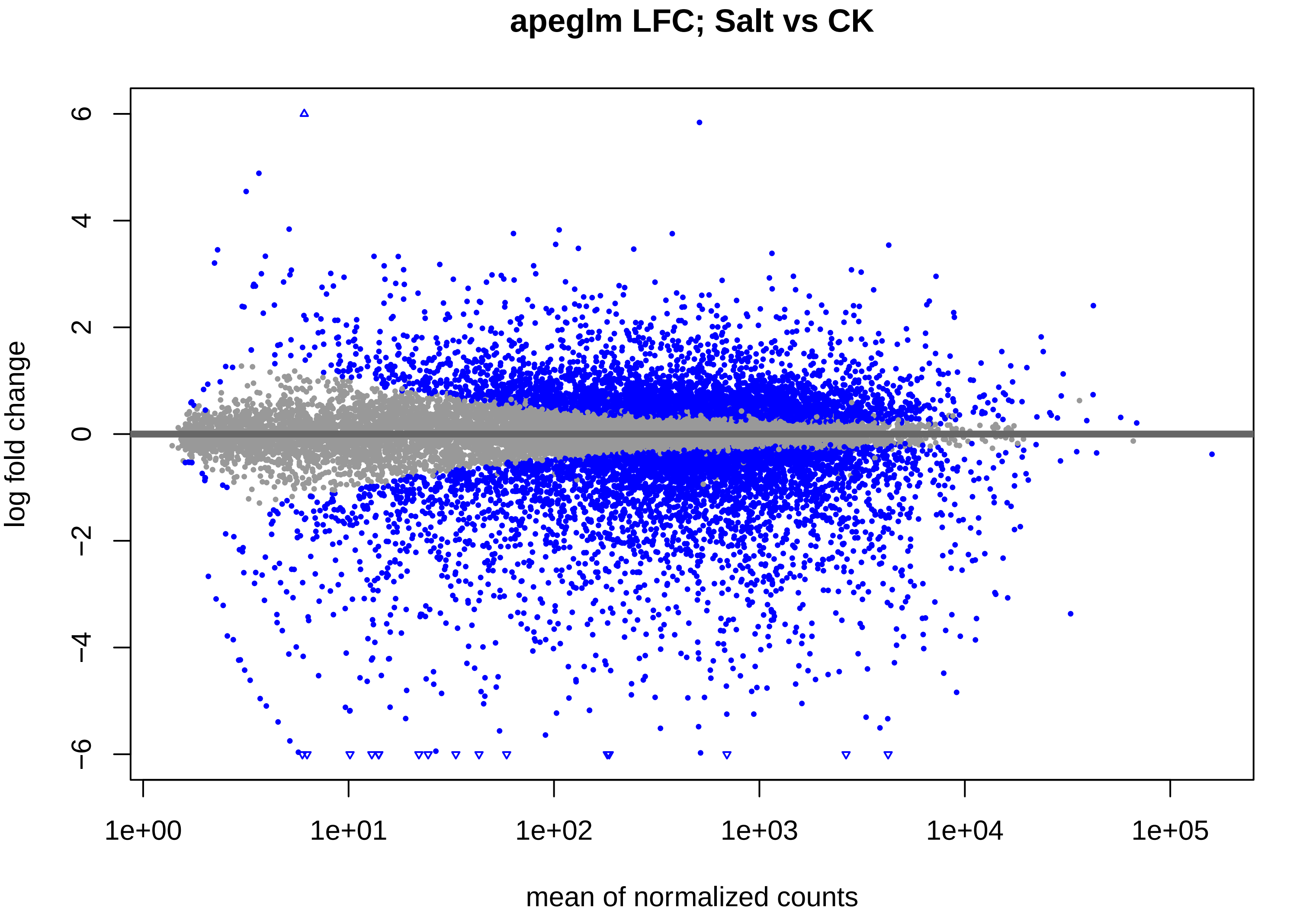
apeglm LFC; Salt vs CK
log fold change
6
4
2
0
−2
−4
−6
1e+00
1e+01
1e+02
1e+03
1e+04
1e+05
mean of normalized counts

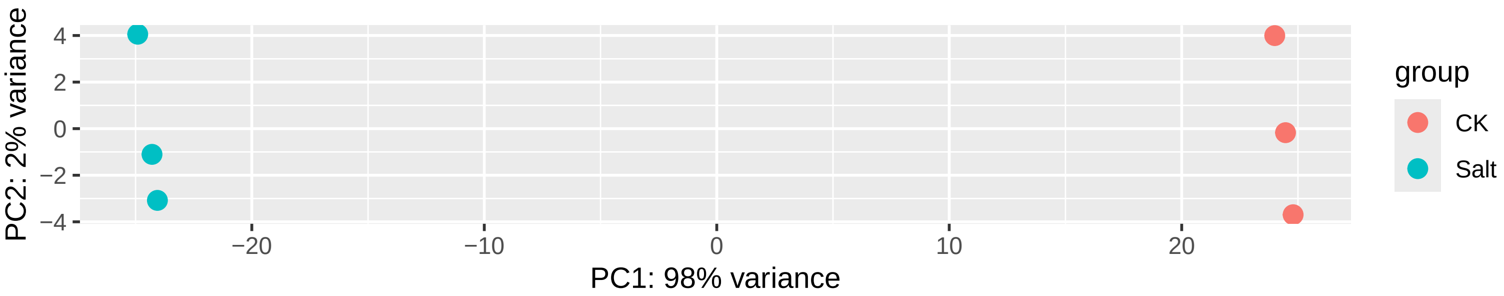
4
2
0
−2
−4
PC2: 2% variance
−20
−10
0
10
20
PC1: 98% variance
group
CK
Salt
