# Supplementary material for: Genome-Wide Identification, Evolutionary Characterization, and Salt-Stress Expression Profiling of Antimicrobial Peptide Gene Families in the Xerophytic Recretohalophyte Reaumuria soongarica
Source: Genes (Basel). 2026 Sep 16;17(9):1126. doi: 10.3390/genes17091126 (PMC13606368; doi:10.3390/genes17091126)
Supplement: Supplementary file 1 [file genes-17-01126-s001.zip › Files/File_S7_Tchinensis_expression_context_20260909.zip!/跨物种表达_实施_20260909/compute/verified_results/read_qc/SRR19973986.html]

fastp report at 2026-09-09 16:46:35 

# fastp report Summary General | | | | --- | --- | | fastp version: | 1.3.3 (https://github.com/OpenGene/fastp) | | sequencing: | paired end (150 cycles + 150 cycles) | | mean length before filtering: | 150bp, 150bp | | mean length after filtering: | 150bp, 150bp | | duplication rate: | 13.845439% | | Insert size peak: | 269 | Before filtering | | | | --- | --- | | total reads: | 48.231076 M | | total bases: | 7.234661 G | | Q20 bases: | 7.234549 G (99.998449%) | | Q30 bases: | 7.234549 G (99.998449%) | | Q40 bases: | 0 (0.000000%) | | GC content: | 45.325989% | After filtering | | | | --- | --- | | total reads: | 48.231076 M | | total bases: | 7.234661 G | | Q20 bases: | 7.234549 G (99.998449%) | | Q30 bases: | 7.234549 G (99.998449%) | | Q40 bases: | 0 (0.000000%) | | GC content: | 45.325989% | Filtering result | | | | --- | --- | | reads passed filters: | 48.231076 M (100.000000%) | | reads with low quality: | 0 (0.000000%) | | reads with too many N: | 0 (0.000000%) | Insert size estimation This estimation is based on paired-end overlap analysis, and there are 80.711859% reads found not overlapped. The nonoverlapped read pairs may have insert size <30 or >270, or contain too much sequencing errors to be detected as overlapped. Filtering statistics (although no output file specified, the filtered statistics are still presented here) | | | | --- | --- | | Before filtering: read1: quality Value of each position will be shown on mouse over. | After filtering: read1: quality Value of each position will be shown on mouse over. | | Before filtering: read2: quality Value of each position will be shown on mouse over. | After filtering: read2: quality Value of each position will be shown on mouse over. | | | | | | | | | | | Before filtering: read1: base contents Value of each position will be shown on mouse over. | After filtering: read1: base contents Value of each position will be shown on mouse over. | | Before filtering: read2: base contents Value of each position will be shown on mouse over. | After filtering: read2: base contents Value of each position will be shown on mouse over. | | | | | Before filtering: read1: KMER counting Darker background means larger counts. The count will be shown on mouse over. \| AA \| AT \| AC \| AG \| TA \| TT \| TC \| TG \| CA \| CT \| CC \| CG \| GA \| GT \| GC \| GG; AAA \| AAAAA \| AAAAT \| AAAAC \| AAAAG \| AAATA \| AAATT \| AAATC \| AAATG \| AAACA \| AAACT \| AAACC \| AAACG \| AAAGA \| AAAGT \| AAAGC \| AAAGG; AAT \| AATAA \| AATAT \| AATAC \| AATAG \| AATTA \| AATTT \| AATTC \| AATTG \| AATCA \| AATCT \| AATCC \| AATCG \| AATGA \| AATGT \| AATGC \| AATGG; AAC \| AACAA \| AACAT \| AACAC \| AACAG \| AACTA \| AACTT \| AACTC \| AACTG \| AACCA \| AACCT \| AACCC \| AACCG \| AACGA \| AACGT \| AACGC \| AACGG; AAG \| AAGAA \| AAGAT \| AAGAC \| AAGAG \| AAGTA \| AAGTT \| AAGTC \| AAGTG \| AAGCA \| AAGCT \| AAGCC \| AAGCG \| AAGGA \| AAGGT \| AAGGC \| AAGGG; ATA \| ATAAA \| ATAAT \| ATAAC \| ATAAG \| ATATA \| ATATT \| ATATC \| ATATG \| ATACA \| ATACT \| ATACC \| ATACG \| ATAGA \| ATAGT \| ATAGC \| ATAGG; ATT \| ATTAA \| ATTAT \| ATTAC \| ATTAG \| ATTTA \| ATTTT \| ATTTC \| ATTTG \| ATTCA \| ATTCT \| ATTCC \| ATTCG \| ATTGA \| ATTGT \| ATTGC \| ATTGG; ATC \| ATCAA \| ATCAT \| ATCAC \| ATCAG \| ATCTA \| ATCTT \| ATCTC \| ATCTG \| ATCCA \| ATCCT \| ATCCC \| ATCCG \| ATCGA \| ATCGT \| ATCGC \| ATCGG; ATG \| ATGAA \| ATGAT \| ATGAC \| ATGAG \| ATGTA \| ATGTT \| ATGTC \| ATGTG \| ATGCA \| ATGCT \| ATGCC \| ATGCG \| ATGGA \| ATGGT \| ATGGC \| ATGGG; ACA \| ACAAA \| ACAAT \| ACAAC \| ACAAG \| ACATA \| ACATT \| ACATC \| ACATG \| ACACA \| ACACT \| ACACC \| ACACG \| ACAGA \| ACAGT \| ACAGC \| ACAGG; ACT \| ACTAA \| ACTAT \| ACTAC \| ACTAG \| ACTTA \| ACTTT \| ACTTC \| ACTTG \| ACTCA \| ACTCT \| ACTCC \| ACTCG \| ACTGA \| ACTGT \| ACTGC \| ACTGG; ACC \| ACCAA \| ACCAT \| ACCAC \| ACCAG \| ACCTA \| ACCTT \| ACCTC \| ACCTG \| ACCCA \| ACCCT \| ACCCC \| ACCCG \| ACCGA \| ACCGT \| ACCGC \| ACCGG; ACG \| ACGAA \| ACGAT \| ACGAC \| ACGAG \| ACGTA \| ACGTT \| ACGTC \| ACGTG \| ACGCA \| ACGCT \| ACGCC \| ACGCG \| ACGGA \| ACGGT \| ACGGC \| ACGGG; AGA \| AGAAA \| AGAAT \| AGAAC \| AGAAG \| AGATA \| AGATT \| AGATC \| AGATG \| AGACA \| AGACT \| AGACC \| AGACG \| AGAGA \| AGAGT \| AGAGC \| AGAGG; AGT \| AGTAA \| AGTAT \| AGTAC \| AGTAG \| AGTTA \| AGTTT \| AGTTC \| AGTTG \| AGTCA \| AGTCT \| AGTCC \| AGTCG \| AGTGA \| AGTGT \| AGTGC \| AGTGG; AGC \| AGCAA \| AGCAT \| AGCAC \| AGCAG \| AGCTA \| AGCTT \| AGCTC \| AGCTG \| AGCCA \| AGCCT \| AGCCC \| AGCCG \| AGCGA \| AGCGT \| AGCGC \| AGCGG; AGG \| AGGAA \| AGGAT \| AGGAC \| AGGAG \| AGGTA \| AGGTT \| AGGTC \| AGGTG \| AGGCA \| AGGCT \| AGGCC \| AGGCG \| AGGGA \| AGGGT \| AGGGC \| AGGGG; TAA \| TAAAA \| TAAAT \| TAAAC \| TAAAG \| TAATA \| TAATT \| TAATC \| TAATG \| TAACA \| TAACT \| TAACC \| TAACG \| TAAGA \| TAAGT \| TAAGC \| TAAGG; TAT \| TATAA \| TATAT \| TATAC \| TATAG \| TATTA \| TATTT \| TATTC \| TATTG \| TATCA \| TATCT \| TATCC \| TATCG \| TATGA \| TATGT \| TATGC \| TATGG; TAC \| TACAA \| TACAT \| TACAC \| TACAG \| TACTA \| TACTT \| TACTC \| TACTG \| TACCA \| TACCT \| TACCC \| TACCG \| TACGA \| TACGT \| TACGC \| TACGG; TAG \| TAGAA \| TAGAT \| TAGAC \| TAGAG \| TAGTA \| TAGTT \| TAGTC \| TAGTG \| TAGCA \| TAGCT \| TAGCC \| TAGCG \| TAGGA \| TAGGT \| TAGGC \| TAGGG; TTA \| TTAAA \| TTAAT \| TTAAC \| TTAAG \| TTATA \| TTATT \| TTATC \| TTATG \| TTACA \| TTACT \| TTACC \| TTACG \| TTAGA \| TTAGT \| TTAGC \| TTAGG; TTT \| TTTAA \| TTTAT \| TTTAC \| TTTAG \| TTTTA \| TTTTT \| TTTTC \| TTTTG \| TTTCA \| TTTCT \| TTTCC \| TTTCG \| TTTGA \| TTTGT \| TTTGC \| TTTGG; TTC \| TTCAA \| TTCAT \| TTCAC \| TTCAG \| TTCTA \| TTCTT \| TTCTC \| TTCTG \| TTCCA \| TTCCT \| TTCCC \| TTCCG \| TTCGA \| TTCGT \| TTCGC \| TTCGG; TTG \| TTGAA \| TTGAT \| TTGAC \| TTGAG \| TTGTA \| TTGTT \| TTGTC \| TTGTG \| TTGCA \| TTGCT \| TTGCC \| TTGCG \| TTGGA \| TTGGT \| TTGGC \| TTGGG; TCA \| TCAAA \| TCAAT \| TCAAC \| TCAAG \| TCATA \| TCATT \| TCATC \| TCATG \| TCACA \| TCACT \| TCACC \| TCACG \| TCAGA \| TCAGT \| TCAGC \| TCAGG; TCT \| TCTAA \| TCTAT \| TCTAC \| TCTAG \| TCTTA \| TCTTT \| TCTTC \| TCTTG \| TCTCA \| TCTCT \| TCTCC \| TCTCG \| TCTGA \| TCTGT \| TCTGC \| TCTGG; TCC \| TCCAA \| TCCAT \| TCCAC \| TCCAG \| TCCTA \| TCCTT \| TCCTC \| TCCTG \| TCCCA \| TCCCT \| TCCCC \| TCCCG \| TCCGA \| TCCGT \| TCCGC \| TCCGG; TCG \| TCGAA \| TCGAT \| TCGAC \| TCGAG \| TCGTA \| TCGTT \| TCGTC \| TCGTG \| TCGCA \| TCGCT \| TCGCC \| TCGCG \| TCGGA \| TCGGT \| TCGGC \| TCGGG; TGA \| TGAAA \| TGAAT \| TGAAC \| TGAAG \| TGATA \| TGATT \| TGATC \| TGATG \| TGACA \| TGACT \| TGACC \| TGACG \| TGAGA \| TGAGT \| TGAGC \| TGAGG; TGT \| TGTAA \| TGTAT \| TGTAC \| TGTAG \| TGTTA \| TGTTT \| TGTTC \| TGTTG \| TGTCA \| TGTCT \| TGTCC \| TGTCG \| TGTGA \| TGTGT \| TGTGC \| TGTGG; TGC \| TGCAA \| TGCAT \| TGCAC \| TGCAG \| TGCTA \| TGCTT \| TGCTC \| TGCTG \| TGCCA \| TGCCT \| TGCCC \| TGCCG \| TGCGA \| TGCGT \| TGCGC \| TGCGG; TGG \| TGGAA \| TGGAT \| TGGAC \| TGGAG \| TGGTA \| TGGTT \| TGGTC \| TGGTG \| TGGCA \| TGGCT \| TGGCC \| TGGCG \| TGGGA \| TGGGT \| TGGGC \| TGGGG; CAA \| CAAAA \| CAAAT \| CAAAC \| CAAAG \| CAATA \| CAATT \| CAATC \| CAATG \| CAACA \| CAACT \| CAACC \| CAACG \| CAAGA \| CAAGT \| CAAGC \| CAAGG; CAT \| CATAA \| CATAT \| CATAC \| CATAG \| CATTA \| CATTT \| CATTC \| CATTG \| CATCA \| CATCT \| CATCC \| CATCG \| CATGA \| CATGT \| CATGC \| CATGG; CAC \| CACAA \| CACAT \| CACAC \| CACAG \| CACTA \| CACTT \| CACTC \| CACTG \| CACCA \| CACCT \| CACCC \| CACCG \| CACGA \| CACGT \| CACGC \| CACGG; CAG \| CAGAA \| CAGAT \| CAGAC \| CAGAG \| CAGTA \| CAGTT \| CAGTC \| CAGTG \| CAGCA \| CAGCT \| CAGCC \| CAGCG \| CAGGA \| CAGGT \| CAGGC \| CAGGG; CTA \| CTAAA \| CTAAT \| CTAAC \| CTAAG \| CTATA \| CTATT \| CTATC \| CTATG \| CTACA \| CTACT \| CTACC \| CTACG \| CTAGA \| CTAGT \| CTAGC \| CTAGG; CTT \| CTTAA \| CTTAT \| CTTAC \| CTTAG \| CTTTA \| CTTTT \| CTTTC \| CTTTG \| CTTCA \| CTTCT \| CTTCC \| CTTCG \| CTTGA \| CTTGT \| CTTGC \| CTTGG; CTC \| CTCAA \| CTCAT \| CTCAC \| CTCAG \| CTCTA \| CTCTT \| CTCTC \| CTCTG \| CTCCA \| CTCCT \| CTCCC \| CTCCG \| CTCGA \| CTCGT \| CTCGC \| CTCGG; CTG \| CTGAA \| CTGAT \| CTGAC \| CTGAG \| CTGTA \| CTGTT \| CTGTC \| CTGTG \| CTGCA \| CTGCT \| CTGCC \| CTGCG \| CTGGA \| CTGGT \| CTGGC \| CTGGG; CCA \| CCAAA \| CCAAT \| CCAAC \| CCAAG \| CCATA \| CCATT \| CCATC \| CCATG \| CCACA \| CCACT \| CCACC \| CCACG \| CCAGA \| CCAGT \| CCAGC \| CCAGG; CCT \| CCTAA \| CCTAT \| CCTAC \| CCTAG \| CCTTA \| CCTTT \| CCTTC \| CCTTG \| CCTCA \| CCTCT \| CCTCC \| CCTCG \| CCTGA \| CCTGT \| CCTGC \| CCTGG; CCC \| CCCAA \| CCCAT \| CCCAC \| CCCAG \| CCCTA \| CCCTT \| CCCTC \| CCCTG \| CCCCA \| CCCCT \| CCCCC \| CCCCG \| CCCGA \| CCCGT \| CCCGC \| CCCGG; CCG \| CCGAA \| CCGAT \| CCGAC \| CCGAG \| CCGTA \| CCGTT \| CCGTC \| CCGTG \| CCGCA \| CCGCT \| CCGCC \| CCGCG \| CCGGA \| CCGGT \| CCGGC \| CCGGG; CGA \| CGAAA \| CGAAT \| CGAAC \| CGAAG \| CGATA \| CGATT \| CGATC \| CGATG \| CGACA \| CGACT \| CGACC \| CGACG \| CGAGA \| CGAGT \| CGAGC \| CGAGG; CGT \| CGTAA \| CGTAT \| CGTAC \| CGTAG \| CGTTA \| CGTTT \| CGTTC \| CGTTG \| CGTCA \| CGTCT \| CGTCC \| CGTCG \| CGTGA \| CGTGT \| CGTGC \| CGTGG; CGC \| CGCAA \| CGCAT \| CGCAC \| CGCAG \| CGCTA \| CGCTT \| CGCTC \| CGCTG \| CGCCA \| CGCCT \| CGCCC \| CGCCG \| CGCGA \| CGCGT \| CGCGC \| CGCGG; CGG \| CGGAA \| CGGAT \| CGGAC \| CGGAG \| CGGTA \| CGGTT \| CGGTC \| CGGTG \| CGGCA \| CGGCT \| CGGCC \| CGGCG \| CGGGA \| CGGGT \| CGGGC \| CGGGG; GAA \| GAAAA \| GAAAT \| GAAAC \| GAAAG \| GAATA \| GAATT \| GAATC \| GAATG \| GAACA \| GAACT \| GAACC \| GAACG \| GAAGA \| GAAGT \| GAAGC \| GAAGG; GAT \| GATAA \| GATAT \| GATAC \| GATAG \| GATTA \| GATTT \| GATTC \| GATTG \| GATCA \| GATCT \| GATCC \| GATCG \| GATGA \| GATGT \| GATGC \| GATGG; GAC \| GACAA \| GACAT \| GACAC \| GACAG \| GACTA \| GACTT \| GACTC \| GACTG \| GACCA \| GACCT \| GACCC \| GACCG \| GACGA \| GACGT \| GACGC \| GACGG; GAG \| GAGAA \| GAGAT \| GAGAC \| GAGAG \| GAGTA \| GAGTT \| GAGTC \| GAGTG \| GAGCA \| GAGCT \| GAGCC \| GAGCG \| GAGGA \| GAGGT \| GAGGC \| GAGGG; GTA \| GTAAA \| GTAAT \| GTAAC \| GTAAG \| GTATA \| GTATT \| GTATC \| GTATG \| GTACA \| GTACT \| GTACC \| GTACG \| GTAGA \| GTAGT \| GTAGC \| GTAGG; GTT \| GTTAA \| GTTAT \| GTTAC \| GTTAG \| GTTTA \| GTTTT \| GTTTC \| GTTTG \| GTTCA \| GTTCT \| GTTCC \| GTTCG \| GTTGA \| GTTGT \| GTTGC \| GTTGG; GTC \| GTCAA \| GTCAT \| GTCAC \| GTCAG \| GTCTA \| GTCTT \| GTCTC \| GTCTG \| GTCCA \| GTCCT \| GTCCC \| GTCCG \| GTCGA \| GTCGT \| GTCGC \| GTCGG; GTG \| GTGAA \| GTGAT \| GTGAC \| GTGAG \| GTGTA \| GTGTT \| GTGTC \| GTGTG \| GTGCA \| GTGCT \| GTGCC \| GTGCG \| GTGGA \| GTGGT \| GTGGC \| GTGGG; GCA \| GCAAA \| GCAAT \| GCAAC \| GCAAG \| GCATA \| GCATT \| GCATC \| GCATG \| GCACA \| GCACT \| GCACC \| GCACG \| GCAGA \| GCAGT \| GCAGC \| GCAGG; GCT \| GCTAA \| GCTAT \| GCTAC \| GCTAG \| GCTTA \| GCTTT \| GCTTC \| GCTTG \| GCTCA \| GCTCT \| GCTCC \| GCTCG \| GCTGA \| GCTGT \| GCTGC \| GCTGG; GCC \| GCCAA \| GCCAT \| GCCAC \| GCCAG \| GCCTA \| GCCTT \| GCCTC \| GCCTG \| GCCCA \| GCCCT \| GCCCC \| GCCCG \| GCCGA \| GCCGT \| GCCGC \| GCCGG; GCG \| GCGAA \| GCGAT \| GCGAC \| GCGAG \| GCGTA \| GCGTT \| GCGTC \| GCGTG \| GCGCA \| GCGCT \| GCGCC \| GCGCG \| GCGGA \| GCGGT \| GCGGC \| GCGGG; GGA \| GGAAA \| GGAAT \| GGAAC \| GGAAG \| GGATA \| GGATT \| GGATC \| GGATG \| GGACA \| GGACT \| GGACC \| GGACG \| GGAGA \| GGAGT \| GGAGC \| GGAGG; GGT \| GGTAA \| GGTAT \| GGTAC \| GGTAG \| GGTTA \| GGTTT \| GGTTC \| GGTTG \| GGTCA \| GGTCT \| GGTCC \| GGTCG \| GGTGA \| GGTGT \| GGTGC \| GGTGG; GGC \| GGCAA \| GGCAT \| GGCAC \| GGCAG \| GGCTA \| GGCTT \| GGCTC \| GGCTG \| GGCCA \| GGCCT \| GGCCC \| GGCCG \| GGCGA \| GGCGT \| GGCGC \| GGCGG; GGG \| GGGAA \| GGGAT \| GGGAC \| GGGAG \| GGGTA \| GGGTT \| GGGTC \| GGGTG \| GGGCA \| GGGCT \| GGGCC \| GGGCG \| GGGGA \| GGGGT \| GGGGC \| GGGGG | After filtering: read1: KMER counting Darker background means larger counts. The count will be shown on mouse over. \| AA \| AT \| AC \| AG \| TA \| TT \| TC \| TG \| CA \| CT \| CC \| CG \| GA \| GT \| GC \| GG; AAA \| AAAAA \| AAAAT \| AAAAC \| AAAAG \| AAATA \| AAATT \| AAATC \| AAATG \| AAACA \| AAACT \| AAACC \| AAACG \| AAAGA \| AAAGT \| AAAGC \| AAAGG; AAT \| AATAA \| AATAT \| AATAC \| AATAG \| AATTA \| AATTT \| AATTC \| AATTG \| AATCA \| AATCT \| AATCC \| AATCG \| AATGA \| AATGT \| AATGC \| AATGG; AAC \| AACAA \| AACAT \| AACAC \| AACAG \| AACTA \| AACTT \| AACTC \| AACTG \| AACCA \| AACCT \| AACCC \| AACCG \| AACGA \| AACGT \| AACGC \| AACGG; AAG \| AAGAA \| AAGAT \| AAGAC \| AAGAG \| AAGTA \| AAGTT \| AAGTC \| AAGTG \| AAGCA \| AAGCT \| AAGCC \| AAGCG \| AAGGA \| AAGGT \| AAGGC \| AAGGG; ATA \| ATAAA \| ATAAT \| ATAAC \| ATAAG \| ATATA \| ATATT \| ATATC \| ATATG \| ATACA \| ATACT \| ATACC \| ATACG \| ATAGA \| ATAGT \| ATAGC \| ATAGG; ATT \| ATTAA \| ATTAT \| ATTAC \| ATTAG \| ATTTA \| ATTTT \| ATTTC \| ATTTG \| ATTCA \| ATTCT \| ATTCC \| ATTCG \| ATTGA \| ATTGT \| ATTGC \| ATTGG; ATC \| ATCAA \| ATCAT \| ATCAC \| ATCAG \| ATCTA \| ATCTT \| ATCTC \| ATCTG \| ATCCA \| ATCCT \| ATCCC \| ATCCG \| ATCGA \| ATCGT \| ATCGC \| ATCGG; ATG \| ATGAA \| ATGAT \| ATGAC \| ATGAG \| ATGTA \| ATGTT \| ATGTC \| ATGTG \| ATGCA \| ATGCT \| ATGCC \| ATGCG \| ATGGA \| ATGGT \| ATGGC \| ATGGG; ACA \| ACAAA \| ACAAT \| ACAAC \| ACAAG \| ACATA \| ACATT \| ACATC \| ACATG \| ACACA \| ACACT \| ACACC \| ACACG \| ACAGA \| ACAGT \| ACAGC \| ACAGG; ACT \| ACTAA \| ACTAT \| ACTAC \| ACTAG \| ACTTA \| ACTTT \| ACTTC \| ACTTG \| ACTCA \| ACTCT \| ACTCC \| ACTCG \| ACTGA \| ACTGT \| ACTGC \| ACTGG; ACC \| ACCAA \| ACCAT \| ACCAC \| ACCAG \| ACCTA \| ACCTT \| ACCTC \| ACCTG \| ACCCA \| ACCCT \| ACCCC \| ACCCG \| ACCGA \| ACCGT \| ACCGC \| ACCGG; ACG \| ACGAA \| ACGAT \| ACGAC \| ACGAG \| ACGTA \| ACGTT \| ACGTC \| ACGTG \| ACGCA \| ACGCT \| ACGCC \| ACGCG \| ACGGA \| ACGGT \| ACGGC \| ACGGG; AGA \| AGAAA \| AGAAT \| AGAAC \| AGAAG \| AGATA \| AGATT \| AGATC \| AGATG \| AGACA \| AGACT \| AGACC \| AGACG \| AGAGA \| AGAGT \| AGAGC \| AGAGG; AGT \| AGTAA \| AGTAT \| AGTAC \| AGTAG \| AGTTA \| AGTTT \| AGTTC \| AGTTG \| AGTCA \| AGTCT \| AGTCC \| AGTCG \| AGTGA \| AGTGT \| AGTGC \| AGTGG; AGC \| AGCAA \| AGCAT \| AGCAC \| AGCAG \| AGCTA \| AGCTT \| AGCTC \| AGCTG \| AGCCA \| AGCCT \| AGCCC \| AGCCG \| AGCGA \| AGCGT \| AGCGC \| AGCGG; AGG \| AGGAA \| AGGAT \| AGGAC \| AGGAG \| AGGTA \| AGGTT \| AGGTC \| AGGTG \| AGGCA \| AGGCT \| AGGCC \| AGGCG \| AGGGA \| AGGGT \| AGGGC \| AGGGG; TAA \| TAAAA \| TAAAT \| TAAAC \| TAAAG \| TAATA \| TAATT \| TAATC \| TAATG \| TAACA \| TAACT \| TAACC \| TAACG \| TAAGA \| TAAGT \| TAAGC \| TAAGG; TAT \| TATAA \| TATAT \| TATAC \| TATAG \| TATTA \| TATTT \| TATTC \| TATTG \| TATCA \| TATCT \| TATCC \| TATCG \| TATGA \| TATGT \| TATGC \| TATGG; TAC \| TACAA \| TACAT \| TACAC \| TACAG \| TACTA \| TACTT \| TACTC \| TACTG \| TACCA \| TACCT \| TACCC \| TACCG \| TACGA \| TACGT \| TACGC \| TACGG; TAG \| TAGAA \| TAGAT \| TAGAC \| TAGAG \| TAGTA \| TAGTT \| TAGTC \| TAGTG \| TAGCA \| TAGCT \| TAGCC \| TAGCG \| TAGGA \| TAGGT \| TAGGC \| TAGGG; TTA \| TTAAA \| TTAAT \| TTAAC \| TTAAG \| TTATA \| TTATT \| TTATC \| TTATG \| TTACA \| TTACT \| TTACC \| TTACG \| TTAGA \| TTAGT \| TTAGC \| TTAGG; TTT \| TTTAA \| TTTAT \| TTTAC \| TTTAG \| TTTTA \| TTTTT \| TTTTC \| TTTTG \| TTTCA \| TTTCT \| TTTCC \| TTTCG \| TTTGA \| TTTGT \| TTTGC \| TTTGG; TTC \| TTCAA \| TTCAT \| TTCAC \| TTCAG \| TTCTA \| TTCTT \| TTCTC \| TTCTG \| TTCCA \| TTCCT \| TTCCC \| TTCCG \| TTCGA \| TTCGT \| TTCGC \| TTCGG; TTG \| TTGAA \| TTGAT \| TTGAC \| TTGAG \| TTGTA \| TTGTT \| TTGTC \| TTGTG \| TTGCA \| TTGCT \| TTGCC \| TTGCG \| TTGGA \| TTGGT \| TTGGC \| TTGGG; TCA \| TCAAA \| TCAAT \| TCAAC \| TCAAG \| TCATA \| TCATT \| TCATC \| TCATG \| TCACA \| TCACT \| TCACC \| TCACG \| TCAGA \| TCAGT \| TCAGC \| TCAGG; TCT \| TCTAA \| TCTAT \| TCTAC \| TCTAG \| TCTTA \| TCTTT \| TCTTC \| TCTTG \| TCTCA \| TCTCT \| TCTCC \| TCTCG \| TCTGA \| TCTGT \| TCTGC \| TCTGG; TCC \| TCCAA \| TCCAT \| TCCAC \| TCCAG \| TCCTA \| TCCTT \| TCCTC \| TCCTG \| TCCCA \| TCCCT \| TCCCC \| TCCCG \| TCCGA \| TCCGT \| TCCGC \| TCCGG; TCG \| TCGAA \| TCGAT \| TCGAC \| TCGAG \| TCGTA \| TCGTT \| TCGTC \| TCGTG \| TCGCA \| TCGCT \| TCGCC \| TCGCG \| TCGGA \| TCGGT \| TCGGC \| TCGGG; TGA \| TGAAA \| TGAAT \| TGAAC \| TGAAG \| TGATA \| TGATT \| TGATC \| TGATG \| TGACA \| TGACT \| TGACC \| TGACG \| TGAGA \| TGAGT \| TGAGC \| TGAGG; TGT \| TGTAA \| TGTAT \| TGTAC \| TGTAG \| TGTTA \| TGTTT \| TGTTC \| TGTTG \| TGTCA \| TGTCT \| TGTCC \| TGTCG \| TGTGA \| TGTGT \| TGTGC \| TGTGG; TGC \| TGCAA \| TGCAT \| TGCAC \| TGCAG \| TGCTA \| TGCTT \| TGCTC \| TGCTG \| TGCCA \| TGCCT \| TGCCC \| TGCCG \| TGCGA \| TGCGT \| TGCGC \| TGCGG; TGG \| TGGAA \| TGGAT \| TGGAC \| TGGAG \| TGGTA \| TGGTT \| TGGTC \| TGGTG \| TGGCA \| TGGCT \| TGGCC \| TGGCG \| TGGGA \| TGGGT \| TGGGC \| TGGGG; CAA \| CAAAA \| CAAAT \| CAAAC \| CAAAG \| CAATA \| CAATT \| CAATC \| CAATG \| CAACA \| CAACT \| CAACC \| CAACG \| CAAGA \| CAAGT \| CAAGC \| CAAGG; CAT \| CATAA \| CATAT \| CATAC \| CATAG \| CATTA \| CATTT \| CATTC \| CATTG \| CATCA \| CATCT \| CATCC \| CATCG \| CATGA \| CATGT \| CATGC \| CATGG; CAC \| CACAA \| CACAT \| CACAC \| CACAG \| CACTA \| CACTT \| CACTC \| CACTG \| CACCA \| CACCT \| CACCC \| CACCG \| CACGA \| CACGT \| CACGC \| CACGG; CAG \| CAGAA \| CAGAT \| CAGAC \| CAGAG \| CAGTA \| CAGTT \| CAGTC \| CAGTG \| CAGCA \| CAGCT \| CAGCC \| CAGCG \| CAGGA \| CAGGT \| CAGGC \| CAGGG; CTA \| CTAAA \| CTAAT \| CTAAC \| CTAAG \| CTATA \| CTATT \| CTATC \| CTATG \| CTACA \| CTACT \| CTACC \| CTACG \| CTAGA \| CTAGT \| CTAGC \| CTAGG; CTT \| CTTAA \| CTTAT \| CTTAC \| CTTAG \| CTTTA \| CTTTT \| CTTTC \| CTTTG \| CTTCA \| CTTCT \| CTTCC \| CTTCG \| CTTGA \| CTTGT \| CTTGC \| CTTGG; CTC \| CTCAA \| CTCAT \| CTCAC \| CTCAG \| CTCTA \| CTCTT \| CTCTC \| CTCTG \| CTCCA \| CTCCT \| CTCCC \| CTCCG \| CTCGA \| CTCGT \| CTCGC \| CTCGG; CTG \| CTGAA \| CTGAT \| CTGAC \| CTGAG \| CTGTA \| CTGTT \| CTGTC \| CTGTG \| CTGCA \| CTGCT \| CTGCC \| CTGCG \| CTGGA \| CTGGT \| CTGGC \| CTGGG; CCA \| CCAAA \| CCAAT \| CCAAC \| CCAAG \| CCATA \| CCATT \| CCATC \| CCATG \| CCACA \| CCACT \| CCACC \| CCACG \| CCAGA \| CCAGT \| CCAGC \| CCAGG; CCT \| CCTAA \| CCTAT \| CCTAC \| CCTAG \| CCTTA \| CCTTT \| CCTTC \| CCTTG \| CCTCA \| CCTCT \| CCTCC \| CCTCG \| CCTGA \| CCTGT \| CCTGC \| CCTGG; CCC \| CCCAA \| CCCAT \| CCCAC \| CCCAG \| CCCTA \| CCCTT \| CCCTC \| CCCTG \| CCCCA \| CCCCT \| CCCCC \| CCCCG \| CCCGA \| CCCGT \| CCCGC \| CCCGG; CCG \| CCGAA \| CCGAT \| CCGAC \| CCGAG \| CCGTA \| CCGTT \| CCGTC \| CCGTG \| CCGCA \| CCGCT \| CCGCC \| CCGCG \| CCGGA \| CCGGT \| CCGGC \| CCGGG; CGA \| CGAAA \| CGAAT \| CGAAC \| CGAAG \| CGATA \| CGATT \| CGATC \| CGATG \| CGACA \| CGACT \| CGACC \| CGACG \| CGAGA \| CGAGT \| CGAGC \| CGAGG; CGT \| CGTAA \| CGTAT \| CGTAC \| CGTAG \| CGTTA \| CGTTT \| CGTTC \| CGTTG \| CGTCA \| CGTCT \| CGTCC \| CGTCG \| CGTGA \| CGTGT \| CGTGC \| CGTGG; CGC \| CGCAA \| CGCAT \| CGCAC \| CGCAG \| CGCTA \| CGCTT \| CGCTC \| CGCTG \| CGCCA \| CGCCT \| CGCCC \| CGCCG \| CGCGA \| CGCGT \| CGCGC \| CGCGG; CGG \| CGGAA \| CGGAT \| CGGAC \| CGGAG \| CGGTA \| CGGTT \| CGGTC \| CGGTG \| CGGCA \| CGGCT \| CGGCC \| CGGCG \| CGGGA \| CGGGT \| CGGGC \| CGGGG; GAA \| GAAAA \| GAAAT \| GAAAC \| GAAAG \| GAATA \| GAATT \| GAATC \| GAATG \| GAACA \| GAACT \| GAACC \| GAACG \| GAAGA \| GAAGT \| GAAGC \| GAAGG; GAT \| GATAA \| GATAT \| GATAC \| GATAG \| GATTA \| GATTT \| GATTC \| GATTG \| GATCA \| GATCT \| GATCC \| GATCG \| GATGA \| GATGT \| GATGC \| GATGG; GAC \| GACAA \| GACAT \| GACAC \| GACAG \| GACTA \| GACTT \| GACTC \| GACTG \| GACCA \| GACCT \| GACCC \| GACCG \| GACGA \| GACGT \| GACGC \| GACGG; GAG \| GAGAA \| GAGAT \| GAGAC \| GAGAG \| GAGTA \| GAGTT \| GAGTC \| GAGTG \| GAGCA \| GAGCT \| GAGCC \| GAGCG \| GAGGA \| GAGGT \| GAGGC \| GAGGG; GTA \| GTAAA \| GTAAT \| GTAAC \| GTAAG \| GTATA \| GTATT \| GTATC \| GTATG \| GTACA \| GTACT \| GTACC \| GTACG \| GTAGA \| GTAGT \| GTAGC \| GTAGG; GTT \| GTTAA \| GTTAT \| GTTAC \| GTTAG \| GTTTA \| GTTTT \| GTTTC \| GTTTG \| GTTCA \| GTTCT \| GTTCC \| GTTCG \| GTTGA \| GTTGT \| GTTGC \| GTTGG; GTC \| GTCAA \| GTCAT \| GTCAC \| GTCAG \| GTCTA \| GTCTT \| GTCTC \| GTCTG \| GTCCA \| GTCCT \| GTCCC \| GTCCG \| GTCGA \| GTCGT \| GTCGC \| GTCGG; GTG \| GTGAA \| GTGAT \| GTGAC \| GTGAG \| GTGTA \| GTGTT \| GTGTC \| GTGTG \| GTGCA \| GTGCT \| GTGCC \| GTGCG \| GTGGA \| GTGGT \| GTGGC \| GTGGG; GCA \| GCAAA \| GCAAT \| GCAAC \| GCAAG \| GCATA \| GCATT \| GCATC \| GCATG \| GCACA \| GCACT \| GCACC \| GCACG \| GCAGA \| GCAGT \| GCAGC \| GCAGG; GCT \| GCTAA \| GCTAT \| GCTAC \| GCTAG \| GCTTA \| GCTTT \| GCTTC \| GCTTG \| GCTCA \| GCTCT \| GCTCC \| GCTCG \| GCTGA \| GCTGT \| GCTGC \| GCTGG; GCC \| GCCAA \| GCCAT \| GCCAC \| GCCAG \| GCCTA \| GCCTT \| GCCTC \| GCCTG \| GCCCA \| GCCCT \| GCCCC \| GCCCG \| GCCGA \| GCCGT \| GCCGC \| GCCGG; GCG \| GCGAA \| GCGAT \| GCGAC \| GCGAG \| GCGTA \| GCGTT \| GCGTC \| GCGTG \| GCGCA \| GCGCT \| GCGCC \| GCGCG \| GCGGA \| GCGGT \| GCGGC \| GCGGG; GGA \| GGAAA \| GGAAT \| GGAAC \| GGAAG \| GGATA \| GGATT \| GGATC \| GGATG \| GGACA \| GGACT \| GGACC \| GGACG \| GGAGA \| GGAGT \| GGAGC \| GGAGG; GGT \| GGTAA \| GGTAT \| GGTAC \| GGTAG \| GGTTA \| GGTTT \| GGTTC \| GGTTG \| GGTCA \| GGTCT \| GGTCC \| GGTCG \| GGTGA \| GGTGT \| GGTGC \| GGTGG; GGC \| GGCAA \| GGCAT \| GGCAC \| GGCAG \| GGCTA \| GGCTT \| GGCTC \| GGCTG \| GGCCA \| GGCCT \| GGCCC \| GGCCG \| GGCGA \| GGCGT \| GGCGC \| GGCGG; GGG \| GGGAA \| GGGAT \| GGGAC \| GGGAG \| GGGTA \| GGGTT \| GGGTC \| GGGTG \| GGGCA \| GGGCT \| GGGCC \| GGGCG \| GGGGA \| GGGGT \| GGGGC \| GGGGG | | Before filtering: read2: KMER counting Darker background means larger counts. The count will be shown on mouse over. \| AA \| AT \| AC \| AG \| TA \| TT \| TC \| TG \| CA \| CT \| CC \| CG \| GA \| GT \| GC \| GG; AAA \| AAAAA \| AAAAT \| AAAAC \| AAAAG \| AAATA \| AAATT \| AAATC \| AAATG \| AAACA \| AAACT \| AAACC \| AAACG \| AAAGA \| AAAGT \| AAAGC \| AAAGG; AAT \| AATAA \| AATAT \| AATAC \| AATAG \| AATTA \| AATTT \| AATTC \| AATTG \| AATCA \| AATCT \| AATCC \| AATCG \| AATGA \| AATGT \| AATGC \| AATGG; AAC \| AACAA \| AACAT \| AACAC \| AACAG \| AACTA \| AACTT \| AACTC \| AACTG \| AACCA \| AACCT \| AACCC \| AACCG \| AACGA \| AACGT \| AACGC \| AACGG; AAG \| AAGAA \| AAGAT \| AAGAC \| AAGAG \| AAGTA \| AAGTT \| AAGTC \| AAGTG \| AAGCA \| AAGCT \| AAGCC \| AAGCG \| AAGGA \| AAGGT \| AAGGC \| AAGGG; ATA \| ATAAA \| ATAAT \| ATAAC \| ATAAG \| ATATA \| ATATT \| ATATC \| ATATG \| ATACA \| ATACT \| ATACC \| ATACG \| ATAGA \| ATAGT \| ATAGC \| ATAGG; ATT \| ATTAA \| ATTAT \| ATTAC \| ATTAG \| ATTTA \| ATTTT \| ATTTC \| ATTTG \| ATTCA \| ATTCT \| ATTCC \| ATTCG \| ATTGA \| ATTGT \| ATTGC \| ATTGG; ATC \| ATCAA \| ATCAT \| ATCAC \| ATCAG \| ATCTA \| ATCTT \| ATCTC \| ATCTG \| ATCCA \| ATCCT \| ATCCC \| ATCCG \| ATCGA \| ATCGT \| ATCGC \| ATCGG; ATG \| ATGAA \| ATGAT \| ATGAC \| ATGAG \| ATGTA \| ATGTT \| ATGTC \| ATGTG \| ATGCA \| ATGCT \| ATGCC \| ATGCG \| ATGGA \| ATGGT \| ATGGC \| ATGGG; ACA \| ACAAA \| ACAAT \| ACAAC \| ACAAG \| ACATA \| ACATT \| ACATC \| ACATG \| ACACA \| ACACT \| ACACC \| ACACG \| ACAGA \| ACAGT \| ACAGC \| ACAGG; ACT \| ACTAA \| ACTAT \| ACTAC \| ACTAG \| ACTTA \| ACTTT \| ACTTC \| ACTTG \| ACTCA \| ACTCT \| ACTCC \| ACTCG \| ACTGA \| ACTGT \| ACTGC \| ACTGG; ACC \| ACCAA \| ACCAT \| ACCAC \| ACCAG \| ACCTA \| ACCTT \| ACCTC \| ACCTG \| ACCCA \| ACCCT \| ACCCC \| ACCCG \| ACCGA \| ACCGT \| ACCGC \| ACCGG; ACG \| ACGAA \| ACGAT \| ACGAC \| ACGAG \| ACGTA \| ACGTT \| ACGTC \| ACGTG \| ACGCA \| ACGCT \| ACGCC \| ACGCG \| ACGGA \| ACGGT \| ACGGC \| ACGGG; AGA \| AGAAA \| AGAAT \| AGAAC \| AGAAG \| AGATA \| AGATT \| AGATC \| AGATG \| AGACA \| AGACT \| AGACC \| AGACG \| AGAGA \| AGAGT \| AGAGC \| AGAGG; AGT \| AGTAA \| AGTAT \| AGTAC \| AGTAG \| AGTTA \| AGTTT \| AGTTC \| AGTTG \| AGTCA \| AGTCT \| AGTCC \| AGTCG \| AGTGA \| AGTGT \| AGTGC \| AGTGG; AGC \| AGCAA \| AGCAT \| AGCAC \| AGCAG \| AGCTA \| AGCTT \| AGCTC \| AGCTG \| AGCCA \| AGCCT \| AGCCC \| AGCCG \| AGCGA \| AGCGT \| AGCGC \| AGCGG; AGG \| AGGAA \| AGGAT \| AGGAC \| AGGAG \| AGGTA \| AGGTT \| AGGTC \| AGGTG \| AGGCA \| AGGCT \| AGGCC \| AGGCG \| AGGGA \| AGGGT \| AGGGC \| AGGGG; TAA \| TAAAA \| TAAAT \| TAAAC \| TAAAG \| TAATA \| TAATT \| TAATC \| TAATG \| TAACA \| TAACT \| TAACC \| TAACG \| TAAGA \| TAAGT \| TAAGC \| TAAGG; TAT \| TATAA \| TATAT \| TATAC \| TATAG \| TATTA \| TATTT \| TATTC \| TATTG \| TATCA \| TATCT \| TATCC \| TATCG \| TATGA \| TATGT \| TATGC \| TATGG; TAC \| TACAA \| TACAT \| TACAC \| TACAG \| TACTA \| TACTT \| TACTC \| TACTG \| TACCA \| TACCT \| TACCC \| TACCG \| TACGA \| TACGT \| TACGC \| TACGG; TAG \| TAGAA \| TAGAT \| TAGAC \| TAGAG \| TAGTA \| TAGTT \| TAGTC \| TAGTG \| TAGCA \| TAGCT \| TAGCC \| TAGCG \| TAGGA \| TAGGT \| TAGGC \| TAGGG; TTA \| TTAAA \| TTAAT \| TTAAC \| TTAAG \| TTATA \| TTATT \| TTATC \| TTATG \| TTACA \| TTACT \| TTACC \| TTACG \| TTAGA \| TTAGT \| TTAGC \| TTAGG; TTT \| TTTAA \| TTTAT \| TTTAC \| TTTAG \| TTTTA \| TTTTT \| TTTTC \| TTTTG \| TTTCA \| TTTCT \| TTTCC \| TTTCG \| TTTGA \| TTTGT \| TTTGC \| TTTGG; TTC \| TTCAA \| TTCAT \| TTCAC \| TTCAG \| TTCTA \| TTCTT \| TTCTC \| TTCTG \| TTCCA \| TTCCT \| TTCCC \| TTCCG \| TTCGA \| TTCGT \| TTCGC \| TTCGG; TTG \| TTGAA \| TTGAT \| TTGAC \| TTGAG \| TTGTA \| TTGTT \| TTGTC \| TTGTG \| TTGCA \| TTGCT \| TTGCC \| TTGCG \| TTGGA \| TTGGT \| TTGGC \| TTGGG; TCA \| TCAAA \| TCAAT \| TCAAC \| TCAAG \| TCATA \| TCATT \| TCATC \| TCATG \| TCACA \| TCACT \| TCACC \| TCACG \| TCAGA \| TCAGT \| TCAGC \| TCAGG; TCT \| TCTAA \| TCTAT \| TCTAC \| TCTAG \| TCTTA \| TCTTT \| TCTTC \| TCTTG \| TCTCA \| TCTCT \| TCTCC \| TCTCG \| TCTGA \| TCTGT \| TCTGC \| TCTGG; TCC \| TCCAA \| TCCAT \| TCCAC \| TCCAG \| TCCTA \| TCCTT \| TCCTC \| TCCTG \| TCCCA \| TCCCT \| TCCCC \| TCCCG \| TCCGA \| TCCGT \| TCCGC \| TCCGG; TCG \| TCGAA \| TCGAT \| TCGAC \| TCGAG \| TCGTA \| TCGTT \| TCGTC \| TCGTG \| TCGCA \| TCGCT \| TCGCC \| TCGCG \| TCGGA \| TCGGT \| TCGGC \| TCGGG; TGA \| TGAAA \| TGAAT \| TGAAC \| TGAAG \| TGATA \| TGATT \| TGATC \| TGATG \| TGACA \| TGACT \| TGACC \| TGACG \| TGAGA \| TGAGT \| TGAGC \| TGAGG; TGT \| TGTAA \| TGTAT \| TGTAC \| TGTAG \| TGTTA \| TGTTT \| TGTTC \| TGTTG \| TGTCA \| TGTCT \| TGTCC \| TGTCG \| TGTGA \| TGTGT \| TGTGC \| TGTGG; TGC \| TGCAA \| TGCAT \| TGCAC \| TGCAG \| TGCTA \| TGCTT \| TGCTC \| TGCTG \| TGCCA \| TGCCT \| TGCCC \| TGCCG \| TGCGA \| TGCGT \| TGCGC \| TGCGG; TGG \| TGGAA \| TGGAT \| TGGAC \| TGGAG \| TGGTA \| TGGTT \| TGGTC \| TGGTG \| TGGCA \| TGGCT \| TGGCC \| TGGCG \| TGGGA \| TGGGT \| TGGGC \| TGGGG; CAA \| CAAAA \| CAAAT \| CAAAC \| CAAAG \| CAATA \| CAATT \| CAATC \| CAATG \| CAACA \| CAACT \| CAACC \| CAACG \| CAAGA \| CAAGT \| CAAGC \| CAAGG; CAT \| CATAA \| CATAT \| CATAC \| CATAG \| CATTA \| CATTT \| CATTC \| CATTG \| CATCA \| CATCT \| CATCC \| CATCG \| CATGA \| CATGT \| CATGC \| CATGG; CAC \| CACAA \| CACAT \| CACAC \| CACAG \| CACTA \| CACTT \| CACTC \| CACTG \| CACCA \| CACCT \| CACCC \| CACCG \| CACGA \| CACGT \| CACGC \| CACGG; CAG \| CAGAA \| CAGAT \| CAGAC \| CAGAG \| CAGTA \| CAGTT \| CAGTC \| CAGTG \| CAGCA \| CAGCT \| CAGCC \| CAGCG \| CAGGA \| CAGGT \| CAGGC \| CAGGG; CTA \| CTAAA \| CTAAT \| CTAAC \| CTAAG \| CTATA \| CTATT \| CTATC \| CTATG \| CTACA \| CTACT \| CTACC \| CTACG \| CTAGA \| CTAGT \| CTAGC \| CTAGG; CTT \| CTTAA \| CTTAT \| CTTAC \| CTTAG \| CTTTA \| CTTTT \| CTTTC \| CTTTG \| CTTCA \| CTTCT \| CTTCC \| CTTCG \| CTTGA \| CTTGT \| CTTGC \| CTTGG; CTC \| CTCAA \| CTCAT \| CTCAC \| CTCAG \| CTCTA \| CTCTT \| CTCTC \| CTCTG \| CTCCA \| CTCCT \| CTCCC \| CTCCG \| CTCGA \| CTCGT \| CTCGC \| CTCGG; CTG \| CTGAA \| CTGAT \| CTGAC \| CTGAG \| CTGTA \| CTGTT \| CTGTC \| CTGTG \| CTGCA \| CTGCT \| CTGCC \| CTGCG \| CTGGA \| CTGGT \| CTGGC \| CTGGG; CCA \| CCAAA \| CCAAT \| CCAAC \| CCAAG \| CCATA \| CCATT \| CCATC \| CCATG \| CCACA \| CCACT \| CCACC \| CCACG \| CCAGA \| CCAGT \| CCAGC \| CCAGG; CCT \| CCTAA \| CCTAT \| CCTAC \| CCTAG \| CCTTA \| CCTTT \| CCTTC \| CCTTG \| CCTCA \| CCTCT \| CCTCC \| CCTCG \| CCTGA \| CCTGT \| CCTGC \| CCTGG; CCC \| CCCAA \| CCCAT \| CCCAC \| CCCAG \| CCCTA \| CCCTT \| CCCTC \| CCCTG \| CCCCA \| CCCCT \| CCCCC \| CCCCG \| CCCGA \| CCCGT \| CCCGC \| CCCGG; CCG \| CCGAA \| CCGAT \| CCGAC \| CCGAG \| CCGTA \| CCGTT \| CCGTC \| CCGTG \| CCGCA \| CCGCT \| CCGCC \| CCGCG \| CCGGA \| CCGGT \| CCGGC \| CCGGG; CGA \| CGAAA \| CGAAT \| CGAAC \| CGAAG \| CGATA \| CGATT \| CGATC \| CGATG \| CGACA \| CGACT \| CGACC \| CGACG \| CGAGA \| CGAGT \| CGAGC \| CGAGG; CGT \| CGTAA \| CGTAT \| CGTAC \| CGTAG \| CGTTA \| CGTTT \| CGTTC \| CGTTG \| CGTCA \| CGTCT \| CGTCC \| CGTCG \| CGTGA \| CGTGT \| CGTGC \| CGTGG; CGC \| CGCAA \| CGCAT \| CGCAC \| CGCAG \| CGCTA \| CGCTT \| CGCTC \| CGCTG \| CGCCA \| CGCCT \| CGCCC \| CGCCG \| CGCGA \| CGCGT \| CGCGC \| CGCGG; CGG \| CGGAA \| CGGAT \| CGGAC \| CGGAG \| CGGTA \| CGGTT \| CGGTC \| CGGTG \| CGGCA \| CGGCT \| CGGCC \| CGGCG \| CGGGA \| CGGGT \| CGGGC \| CGGGG; GAA \| GAAAA \| GAAAT \| GAAAC \| GAAAG \| GAATA \| GAATT \| GAATC \| GAATG \| GAACA \| GAACT \| GAACC \| GAACG \| GAAGA \| GAAGT \| GAAGC \| GAAGG; GAT \| GATAA \| GATAT \| GATAC \| GATAG \| GATTA \| GATTT \| GATTC \| GATTG \| GATCA \| GATCT \| GATCC \| GATCG \| GATGA \| GATGT \| GATGC \| GATGG; GAC \| GACAA \| GACAT \| GACAC \| GACAG \| GACTA \| GACTT \| GACTC \| GACTG \| GACCA \| GACCT \| GACCC \| GACCG \| GACGA \| GACGT \| GACGC \| GACGG; GAG \| GAGAA \| GAGAT \| GAGAC \| GAGAG \| GAGTA \| GAGTT \| GAGTC \| GAGTG \| GAGCA \| GAGCT \| GAGCC \| GAGCG \| GAGGA \| GAGGT \| GAGGC \| GAGGG; GTA \| GTAAA \| GTAAT \| GTAAC \| GTAAG \| GTATA \| GTATT \| GTATC \| GTATG \| GTACA \| GTACT \| GTACC \| GTACG \| GTAGA \| GTAGT \| GTAGC \| GTAGG; GTT \| GTTAA \| GTTAT \| GTTAC \| GTTAG \| GTTTA \| GTTTT \| GTTTC \| GTTTG \| GTTCA \| GTTCT \| GTTCC \| GTTCG \| GTTGA \| GTTGT \| GTTGC \| GTTGG; GTC \| GTCAA \| GTCAT \| GTCAC \| GTCAG \| GTCTA \| GTCTT \| GTCTC \| GTCTG \| GTCCA \| GTCCT \| GTCCC \| GTCCG \| GTCGA \| GTCGT \| GTCGC \| GTCGG; GTG \| GTGAA \| GTGAT \| GTGAC \| GTGAG \| GTGTA \| GTGTT \| GTGTC \| GTGTG \| GTGCA \| GTGCT \| GTGCC \| GTGCG \| GTGGA \| GTGGT \| GTGGC \| GTGGG; GCA \| GCAAA \| GCAAT \| GCAAC \| GCAAG \| GCATA \| GCATT \| GCATC \| GCATG \| GCACA \| GCACT \| GCACC \| GCACG \| GCAGA \| GCAGT \| GCAGC \| GCAGG; GCT \| GCTAA \| GCTAT \| GCTAC \| GCTAG \| GCTTA \| GCTTT \| GCTTC \| GCTTG \| GCTCA \| GCTCT \| GCTCC \| GCTCG \| GCTGA \| GCTGT \| GCTGC \| GCTGG; GCC \| GCCAA \| GCCAT \| GCCAC \| GCCAG \| GCCTA \| GCCTT \| GCCTC \| GCCTG \| GCCCA \| GCCCT \| GCCCC \| GCCCG \| GCCGA \| GCCGT \| GCCGC \| GCCGG; GCG \| GCGAA \| GCGAT \| GCGAC \| GCGAG \| GCGTA \| GCGTT \| GCGTC \| GCGTG \| GCGCA \| GCGCT \| GCGCC \| GCGCG \| GCGGA \| GCGGT \| GCGGC \| GCGGG; GGA \| GGAAA \| GGAAT \| GGAAC \| GGAAG \| GGATA \| GGATT \| GGATC \| GGATG \| GGACA \| GGACT \| GGACC \| GGACG \| GGAGA \| GGAGT \| GGAGC \| GGAGG; GGT \| GGTAA \| GGTAT \| GGTAC \| GGTAG \| GGTTA \| GGTTT \| GGTTC \| GGTTG \| GGTCA \| GGTCT \| GGTCC \| GGTCG \| GGTGA \| GGTGT \| GGTGC \| GGTGG; GGC \| GGCAA \| GGCAT \| GGCAC \| GGCAG \| GGCTA \| GGCTT \| GGCTC \| GGCTG \| GGCCA \| GGCCT \| GGCCC \| GGCCG \| GGCGA \| GGCGT \| GGCGC \| GGCGG; GGG \| GGGAA \| GGGAT \| GGGAC \| GGGAG \| GGGTA \| GGGTT \| GGGTC \| GGGTG \| GGGCA \| GGGCT \| GGGCC \| GGGCG \| GGGGA \| GGGGT \| GGGGC \| GGGGG | After filtering: read2: KMER counting Darker background means larger counts. The count will be shown on mouse over. \| AA \| AT \| AC \| AG \| TA \| TT \| TC \| TG \| CA \| CT \| CC \| CG \| GA \| GT \| GC \| GG; AAA \| AAAAA \| AAAAT \| AAAAC \| AAAAG \| AAATA \| AAATT \| AAATC \| AAATG \| AAACA \| AAACT \| AAACC \| AAACG \| AAAGA \| AAAGT \| AAAGC \| AAAGG; AAT \| AATAA \| AATAT \| AATAC \| AATAG \| AATTA \| AATTT \| AATTC \| AATTG \| AATCA \| AATCT \| AATCC \| AATCG \| AATGA \| AATGT \| AATGC \| AATGG; AAC \| AACAA \| AACAT \| AACAC \| AACAG \| AACTA \| AACTT \| AACTC \| AACTG \| AACCA \| AACCT \| AACCC \| AACCG \| AACGA \| AACGT \| AACGC \| AACGG; AAG \| AAGAA \| AAGAT \| AAGAC \| AAGAG \| AAGTA \| AAGTT \| AAGTC \| AAGTG \| AAGCA \| AAGCT \| AAGCC \| AAGCG \| AAGGA \| AAGGT \| AAGGC \| AAGGG; ATA \| ATAAA \| ATAAT \| ATAAC \| ATAAG \| ATATA \| ATATT \| ATATC \| ATATG \| ATACA \| ATACT \| ATACC \| ATACG \| ATAGA \| ATAGT \| ATAGC \| ATAGG; ATT \| ATTAA \| ATTAT \| ATTAC \| ATTAG \| ATTTA \| ATTTT \| ATTTC \| ATTTG \| ATTCA \| ATTCT \| ATTCC \| ATTCG \| ATTGA \| ATTGT \| ATTGC \| ATTGG; ATC \| ATCAA \| ATCAT \| ATCAC \| ATCAG \| ATCTA \| ATCTT \| ATCTC \| ATCTG \| ATCCA \| ATCCT \| ATCCC \| ATCCG \| ATCGA \| ATCGT \| ATCGC \| ATCGG; ATG \| ATGAA \| ATGAT \| ATGAC \| ATGAG \| ATGTA \| ATGTT \| ATGTC \| ATGTG \| ATGCA \| ATGCT \| ATGCC \| ATGCG \| ATGGA \| ATGGT \| ATGGC \| ATGGG; ACA \| ACAAA \| ACAAT \| ACAAC \| ACAAG \| ACATA \| ACATT \| ACATC \| ACATG \| ACACA \| ACACT \| ACACC \| ACACG \| ACAGA \| ACAGT \| ACAGC \| ACAGG; ACT \| ACTAA \| ACTAT \| ACTAC \| ACTAG \| ACTTA \| ACTTT \| ACTTC \| ACTTG \| ACTCA \| ACTCT \| ACTCC \| ACTCG \| ACTGA \| ACTGT \| ACTGC \| ACTGG; ACC \| ACCAA \| ACCAT \| ACCAC \| ACCAG \| ACCTA \| ACCTT \| ACCTC \| ACCTG \| ACCCA \| ACCCT \| ACCCC \| ACCCG \| ACCGA \| ACCGT \| ACCGC \| ACCGG; ACG \| ACGAA \| ACGAT \| ACGAC \| ACGAG \| ACGTA \| ACGTT \| ACGTC \| ACGTG \| ACGCA \| ACGCT \| ACGCC \| ACGCG \| ACGGA \| ACGGT \| ACGGC \| ACGGG; AGA \| AGAAA \| AGAAT \| AGAAC \| AGAAG \| AGATA \| AGATT \| AGATC \| AGATG \| AGACA \| AGACT \| AGACC \| AGACG \| AGAGA \| AGAGT \| AGAGC \| AGAGG; AGT \| AGTAA \| AGTAT \| AGTAC \| AGTAG \| AGTTA \| AGTTT \| AGTTC \| AGTTG \| AGTCA \| AGTCT \| AGTCC \| AGTCG \| AGTGA \| AGTGT \| AGTGC \| AGTGG; AGC \| AGCAA \| AGCAT \| AGCAC \| AGCAG \| AGCTA \| AGCTT \| AGCTC \| AGCTG \| AGCCA \| AGCCT \| AGCCC \| AGCCG \| AGCGA \| AGCGT \| AGCGC \| AGCGG; AGG \| AGGAA \| AGGAT \| AGGAC \| AGGAG \| AGGTA \| AGGTT \| AGGTC \| AGGTG \| AGGCA \| AGGCT \| AGGCC \| AGGCG \| AGGGA \| AGGGT \| AGGGC \| AGGGG; TAA \| TAAAA \| TAAAT \| TAAAC \| TAAAG \| TAATA \| TAATT \| TAATC \| TAATG \| TAACA \| TAACT \| TAACC \| TAACG \| TAAGA \| TAAGT \| TAAGC \| TAAGG; TAT \| TATAA \| TATAT \| TATAC \| TATAG \| TATTA \| TATTT \| TATTC \| TATTG \| TATCA \| TATCT \| TATCC \| TATCG \| TATGA \| TATGT \| TATGC \| TATGG; TAC \| TACAA \| TACAT \| TACAC \| TACAG \| TACTA \| TACTT \| TACTC \| TACTG \| TACCA \| TACCT \| TACCC \| TACCG \| TACGA \| TACGT \| TACGC \| TACGG; TAG \| TAGAA \| TAGAT \| TAGAC \| TAGAG \| TAGTA \| TAGTT \| TAGTC \| TAGTG \| TAGCA \| TAGCT \| TAGCC \| TAGCG \| TAGGA \| TAGGT \| TAGGC \| TAGGG; TTA \| TTAAA \| TTAAT \| TTAAC \| TTAAG \| TTATA \| TTATT \| TTATC \| TTATG \| TTACA \| TTACT \| TTACC \| TTACG \| TTAGA \| TTAGT \| TTAGC \| TTAGG; TTT \| TTTAA \| TTTAT \| TTTAC \| TTTAG \| TTTTA \| TTTTT \| TTTTC \| TTTTG \| TTTCA \| TTTCT \| TTTCC \| TTTCG \| TTTGA \| TTTGT \| TTTGC \| TTTGG; TTC \| TTCAA \| TTCAT \| TTCAC \| TTCAG \| TTCTA \| TTCTT \| TTCTC \| TTCTG \| TTCCA \| TTCCT \| TTCCC \| TTCCG \| TTCGA \| TTCGT \| TTCGC \| TTCGG; TTG \| TTGAA \| TTGAT \| TTGAC \| TTGAG \| TTGTA \| TTGTT \| TTGTC \| TTGTG \| TTGCA \| TTGCT \| TTGCC \| TTGCG \| TTGGA \| TTGGT \| TTGGC \| TTGGG; TCA \| TCAAA \| TCAAT \| TCAAC \| TCAAG \| TCATA \| TCATT \| TCATC \| TCATG \| TCACA \| TCACT \| TCACC \| TCACG \| TCAGA \| TCAGT \| TCAGC \| TCAGG; TCT \| TCTAA \| TCTAT \| TCTAC \| TCTAG \| TCTTA \| TCTTT \| TCTTC \| TCTTG \| TCTCA \| TCTCT \| TCTCC \| TCTCG \| TCTGA \| TCTGT \| TCTGC \| TCTGG; TCC \| TCCAA \| TCCAT \| TCCAC \| TCCAG \| TCCTA \| TCCTT \| TCCTC \| TCCTG \| TCCCA \| TCCCT \| TCCCC \| TCCCG \| TCCGA \| TCCGT \| TCCGC \| TCCGG; TCG \| TCGAA \| TCGAT \| TCGAC \| TCGAG \| TCGTA \| TCGTT \| TCGTC \| TCGTG \| TCGCA \| TCGCT \| TCGCC \| TCGCG \| TCGGA \| TCGGT \| TCGGC \| TCGGG; TGA \| TGAAA \| TGAAT \| TGAAC \| TGAAG \| TGATA \| TGATT \| TGATC \| TGATG \| TGACA \| TGACT \| TGACC \| TGACG \| TGAGA \| TGAGT \| TGAGC \| TGAGG; TGT \| TGTAA \| TGTAT \| TGTAC \| TGTAG \| TGTTA \| TGTTT \| TGTTC \| TGTTG \| TGTCA \| TGTCT \| TGTCC \| TGTCG \| TGTGA \| TGTGT \| TGTGC \| TGTGG; TGC \| TGCAA \| TGCAT \| TGCAC \| TGCAG \| TGCTA \| TGCTT \| TGCTC \| TGCTG \| TGCCA \| TGCCT \| TGCCC \| TGCCG \| TGCGA \| TGCGT \| TGCGC \| TGCGG; TGG \| TGGAA \| TGGAT \| TGGAC \| TGGAG \| TGGTA \| TGGTT \| TGGTC \| TGGTG \| TGGCA \| TGGCT \| TGGCC \| TGGCG \| TGGGA \| TGGGT \| TGGGC \| TGGGG; CAA \| CAAAA \| CAAAT \| CAAAC \| CAAAG \| CAATA \| CAATT \| CAATC \| CAATG \| CAACA \| CAACT \| CAACC \| CAACG \| CAAGA \| CAAGT \| CAAGC \| CAAGG; CAT \| CATAA \| CATAT \| CATAC \| CATAG \| CATTA \| CATTT \| CATTC \| CATTG \| CATCA \| CATCT \| CATCC \| CATCG \| CATGA \| CATGT \| CATGC \| CATGG; CAC \| CACAA \| CACAT \| CACAC \| CACAG \| CACTA \| CACTT \| CACTC \| CACTG \| CACCA \| CACCT \| CACCC \| CACCG \| CACGA \| CACGT \| CACGC \| CACGG; CAG \| CAGAA \| CAGAT \| CAGAC \| CAGAG \| CAGTA \| CAGTT \| CAGTC \| CAGTG \| CAGCA \| CAGCT \| CAGCC \| CAGCG \| CAGGA \| CAGGT \| CAGGC \| CAGGG; CTA \| CTAAA \| CTAAT \| CTAAC \| CTAAG \| CTATA \| CTATT \| CTATC \| CTATG \| CTACA \| CTACT \| CTACC \| CTACG \| CTAGA \| CTAGT \| CTAGC \| CTAGG; CTT \| CTTAA \| CTTAT \| CTTAC \| CTTAG \| CTTTA \| CTTTT \| CTTTC \| CTTTG \| CTTCA \| CTTCT \| CTTCC \| CTTCG \| CTTGA \| CTTGT \| CTTGC \| CTTGG; CTC \| CTCAA \| CTCAT \| CTCAC \| CTCAG \| CTCTA \| CTCTT \| CTCTC \| CTCTG \| CTCCA \| CTCCT \| CTCCC \| CTCCG \| CTCGA \| CTCGT \| CTCGC \| CTCGG; CTG \| CTGAA \| CTGAT \| CTGAC \| CTGAG \| CTGTA \| CTGTT \| CTGTC \| CTGTG \| CTGCA \| CTGCT \| CTGCC \| CTGCG \| CTGGA \| CTGGT \| CTGGC \| CTGGG; CCA \| CCAAA \| CCAAT \| CCAAC \| CCAAG \| CCATA \| CCATT \| CCATC \| CCATG \| CCACA \| CCACT \| CCACC \| CCACG \| CCAGA \| CCAGT \| CCAGC \| CCAGG; CCT \| CCTAA \| CCTAT \| CCTAC \| CCTAG \| CCTTA \| CCTTT \| CCTTC \| CCTTG \| CCTCA \| CCTCT \| CCTCC \| CCTCG \| CCTGA \| CCTGT \| CCTGC \| CCTGG; CCC \| CCCAA \| CCCAT \| CCCAC \| CCCAG \| CCCTA \| CCCTT \| CCCTC \| CCCTG \| CCCCA \| CCCCT \| CCCCC \| CCCCG \| CCCGA \| CCCGT \| CCCGC \| CCCGG; CCG \| CCGAA \| CCGAT \| CCGAC \| CCGAG \| CCGTA \| CCGTT \| CCGTC \| CCGTG \| CCGCA \| CCGCT \| CCGCC \| CCGCG \| CCGGA \| CCGGT \| CCGGC \| CCGGG; CGA \| CGAAA \| CGAAT \| CGAAC \| CGAAG \| CGATA \| CGATT \| CGATC \| CGATG \| CGACA \| CGACT \| CGACC \| CGACG \| CGAGA \| CGAGT \| CGAGC \| CGAGG; CGT \| CGTAA \| CGTAT \| CGTAC \| CGTAG \| CGTTA \| CGTTT \| CGTTC \| CGTTG \| CGTCA \| CGTCT \| CGTCC \| CGTCG \| CGTGA \| CGTGT \| CGTGC \| CGTGG; CGC \| CGCAA \| CGCAT \| CGCAC \| CGCAG \| CGCTA \| CGCTT \| CGCTC \| CGCTG \| CGCCA \| CGCCT \| CGCCC \| CGCCG \| CGCGA \| CGCGT \| CGCGC \| CGCGG; CGG \| CGGAA \| CGGAT \| CGGAC \| CGGAG \| CGGTA \| CGGTT \| CGGTC \| CGGTG \| CGGCA \| CGGCT \| CGGCC \| CGGCG \| CGGGA \| CGGGT \| CGGGC \| CGGGG; GAA \| GAAAA \| GAAAT \| GAAAC \| GAAAG \| GAATA \| GAATT \| GAATC \| GAATG \| GAACA \| GAACT \| GAACC \| GAACG \| GAAGA \| GAAGT \| GAAGC \| GAAGG; GAT \| GATAA \| GATAT \| GATAC \| GATAG \| GATTA \| GATTT \| GATTC \| GATTG \| GATCA \| GATCT \| GATCC \| GATCG \| GATGA \| GATGT \| GATGC \| GATGG; GAC \| GACAA \| GACAT \| GACAC \| GACAG \| GACTA \| GACTT \| GACTC \| GACTG \| GACCA \| GACCT \| GACCC \| GACCG \| GACGA \| GACGT \| GACGC \| GACGG; GAG \| GAGAA \| GAGAT \| GAGAC \| GAGAG \| GAGTA \| GAGTT \| GAGTC \| GAGTG \| GAGCA \| GAGCT \| GAGCC \| GAGCG \| GAGGA \| GAGGT \| GAGGC \| GAGGG; GTA \| GTAAA \| GTAAT \| GTAAC \| GTAAG \| GTATA \| GTATT \| GTATC \| GTATG \| GTACA \| GTACT \| GTACC \| GTACG \| GTAGA \| GTAGT \| GTAGC \| GTAGG; GTT \| GTTAA \| GTTAT \| GTTAC \| GTTAG \| GTTTA \| GTTTT \| GTTTC \| GTTTG \| GTTCA \| GTTCT \| GTTCC \| GTTCG \| GTTGA \| GTTGT \| GTTGC \| GTTGG; GTC \| GTCAA \| GTCAT \| GTCAC \| GTCAG \| GTCTA \| GTCTT \| GTCTC \| GTCTG \| GTCCA \| GTCCT \| GTCCC \| GTCCG \| GTCGA \| GTCGT \| GTCGC \| GTCGG; GTG \| GTGAA \| GTGAT \| GTGAC \| GTGAG \| GTGTA \| GTGTT \| GTGTC \| GTGTG \| GTGCA \| GTGCT \| GTGCC \| GTGCG \| GTGGA \| GTGGT \| GTGGC \| GTGGG; GCA \| GCAAA \| GCAAT \| GCAAC \| GCAAG \| GCATA \| GCATT \| GCATC \| GCATG \| GCACA \| GCACT \| GCACC \| GCACG \| GCAGA \| GCAGT \| GCAGC \| GCAGG; GCT \| GCTAA \| GCTAT \| GCTAC \| GCTAG \| GCTTA \| GCTTT \| GCTTC \| GCTTG \| GCTCA \| GCTCT \| GCTCC \| GCTCG \| GCTGA \| GCTGT \| GCTGC \| GCTGG; GCC \| GCCAA \| GCCAT \| GCCAC \| GCCAG \| GCCTA \| GCCTT \| GCCTC \| GCCTG \| GCCCA \| GCCCT \| GCCCC \| GCCCG \| GCCGA \| GCCGT \| GCCGC \| GCCGG; GCG \| GCGAA \| GCGAT \| GCGAC \| GCGAG \| GCGTA \| GCGTT \| GCGTC \| GCGTG \| GCGCA \| GCGCT \| GCGCC \| GCGCG \| GCGGA \| GCGGT \| GCGGC \| GCGGG; GGA \| GGAAA \| GGAAT \| GGAAC \| GGAAG \| GGATA \| GGATT \| GGATC \| GGATG \| GGACA \| GGACT \| GGACC \| GGACG \| GGAGA \| GGAGT \| GGAGC \| GGAGG; GGT \| GGTAA \| GGTAT \| GGTAC \| GGTAG \| GGTTA \| GGTTT \| GGTTC \| GGTTG \| GGTCA \| GGTCT \| GGTCC \| GGTCG \| GGTGA \| GGTGT \| GGTGC \| GGTGG; GGC \| GGCAA \| GGCAT \| GGCAC \| GGCAG \| GGCTA \| GGCTT \| GGCTC \| GGCTG \| GGCCA \| GGCCT \| GGCCC \| GGCCG \| GGCGA \| GGCGT \| GGCGC \| GGCGG; GGG \| GGGAA \| GGGAT \| GGGAC \| GGGAG \| GGGTA \| GGGTT \| GGGTC \| GGGTG \| GGGCA \| GGGCT \| GGGCC \| GGGCG \| GGGGA \| GGGGT \| GGGGC \| GGGGG |

/home/dxy/tools/miniconda3/envs/salmon\_env/bin/fastp -i /data/dxy/project/45\_红砂/revision\_20260909\_cross\_species\_expression/raw\_fastq/SRR19973986\_1.fastq.gz -I /data/dxy/project/45\_红砂/revision\_20260909\_cross\_species\_expression/raw\_fastq/SRR19973986\_2.fastq.gz --disable\_adapter\_trimming --disable\_quality\_filtering --disable\_length\_filtering --disable\_trim\_poly\_g -w 4 -j /data/dxy/project/45\_红砂/revision\_20260909\_cross\_species\_expression/read\_qc/SRR19973986.json -h /data/dxy/project/45\_红砂/revision\_20260909\_cross\_species\_expression/read\_qc/SRR19973986.html

fastp 1.3.3, at 2026-09-09 16:46:35
