# Supplementary material for: Genome-Wide Identification, Evolutionary Characterization, and Salt-Stress Expression Profiling of Antimicrobial Peptide Gene Families in the Xerophytic Recretohalophyte Reaumuria soongarica
Source: Genes (Basel). 2026 Sep 16;17(9):1126. doi: 10.3390/genes17091126 (PMC13606368; doi:10.3390/genes17091126)
Supplement: Supplementary file 1 [file genes-17-01126-s001.zip › Files/File_S8_catalogue_synced_figures_20260909.zip!/analysis/图5图6_紧凑排版/Figure5_primary73_compact.pdf]

Sequence motifs in upstream proxies

73 primary genes · 2,000 bp · 15 double-stranded patterns

Colour: ln(1 + nonredundant site count)

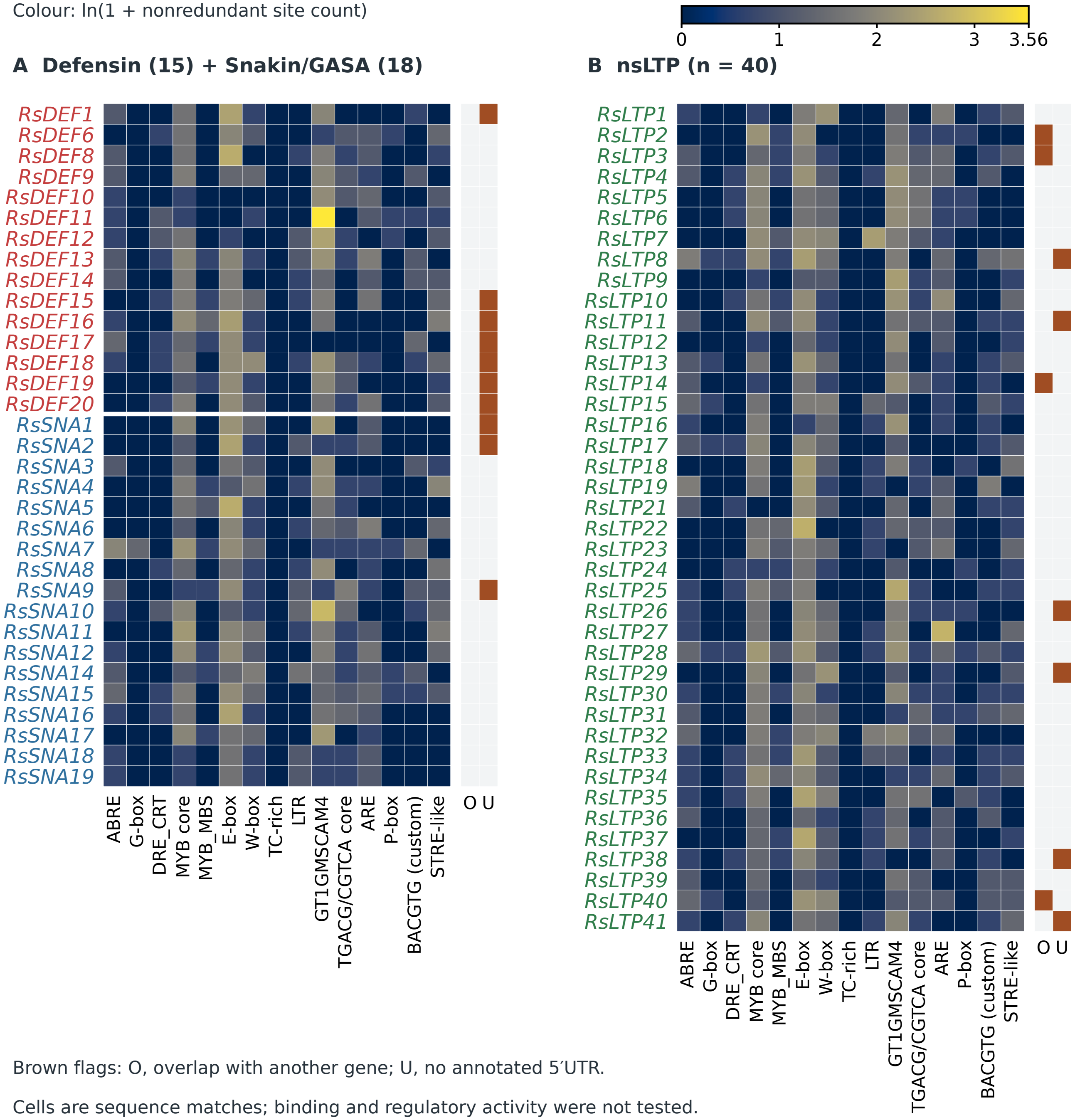

Brown flags: O, overlap with another gene; U, no annotated 5′UTR.

Cells are sequence matches; binding and regulatory activity were not tested.

Proxies use 66 annotated and 7 new-model boundaries; no independently mapped TSSs.
