# Supplementary material for: Genome-Wide Identification, Evolutionary Characterization, and Salt-Stress Expression Profiling of Antimicrobial Peptide Gene Families in the Xerophytic Recretohalophyte Reaumuria soongarica
Source: Genes (Basel). 2026 Sep 16;17(9):1126. doi: 10.3390/genes17091126 (PMC13606368; doi:10.3390/genes17091126)
Supplement: Supplementary file 1 [file genes-17-01126-s001.zip › Files/File_S8_catalogue_synced_figures_20260909.zip!/analysis/图5图6_紧凑排版/Figure6_expression52_compact.pdf]

Expression profiles of reference-covered AMP genes

52 count-filtered members · nine RNA-seq libraries

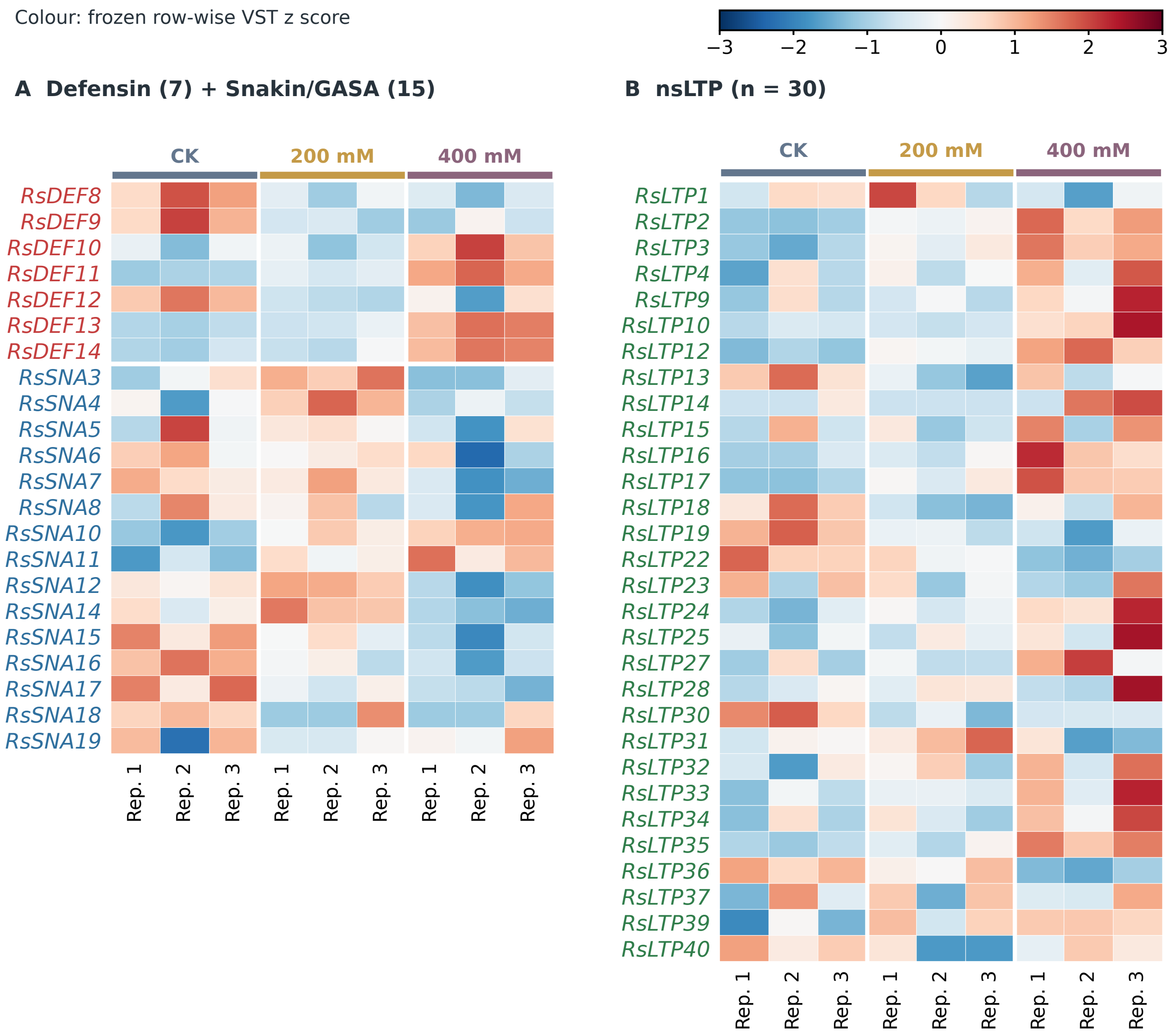

73 primary members: 66 in the original reference; 52 passed the count filter.

Seven new models were not quantified. No missing values were imputed.

Frozen row-wise VST z scores and sample order are retained; no clustering was applied.
