# Supplementary material for: Genome-Wide Identification, Evolutionary Characterization, and Salt-Stress Expression Profiling of Antimicrobial Peptide Gene Families in the Xerophytic Recretohalophyte Reaumuria soongarica
Source: Genes (Basel). 2026 Sep 16;17(9):1126. doi: 10.3390/genes17091126 (PMC13606368; doi:10.3390/genes17091126)
Supplement: Supplementary file 1 [file genes-17-01126-s001.zip › Files/File_S8_catalogue_synced_figures_20260909.zip!/analysis/比较_F8/Figure8_primary73_synced_20260909.pdf]

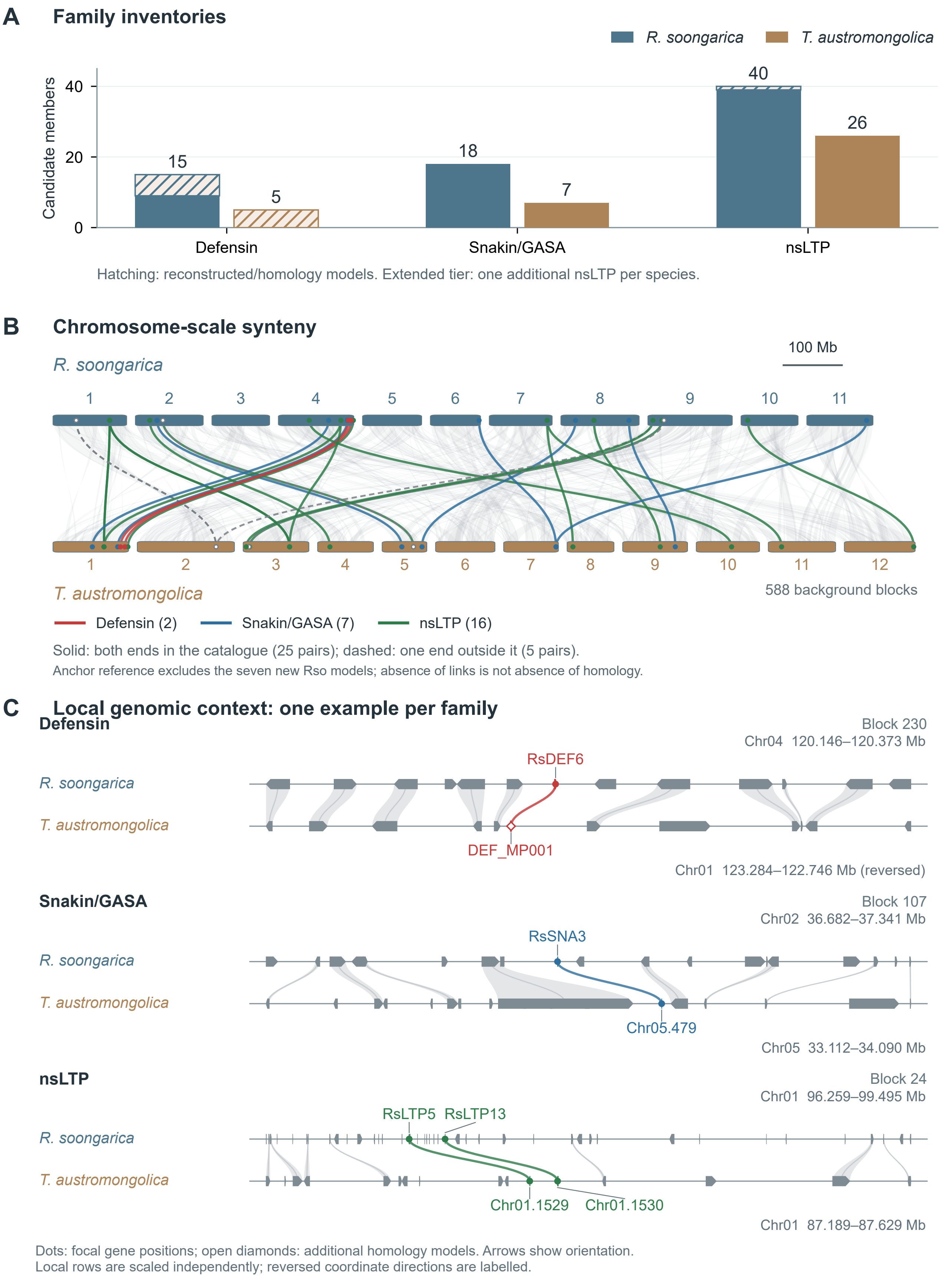
A
Family inventories
R. soongarica
T. austromongolica
Candidate members
0
20
40
15
5
18
7
40
26
Defensin
Snakin/GASA
nsLTP
Hatching: reconstructed/homology models. Extended tier: one additional nsLTP per species.
B
Chromosome-scale synteny
100 Mb
R. soongarica
1 2 3 4 5 6 7 8 9 10 11
1 2 3 4 5 6 7 8 9 10 11 12
T. austromongolica
588 background blocks
Defensin (2)
Snakin/GASA (7)
nsLTP (16)
Solid: both ends in the catalogue (25 pairs); dashed: one end outside it (5 pairs).
Anchor reference excludes the seven new Rso models; absence of links is not absence of homology.
C
Local genomic context: one example per family
Defensin
Block 230
Chr04 120.146–120.373 Mb
RsDEF6
R. soongarica
T. austromongolica
DEF_MP001
Chr01 123.284–122.746 Mb (reversed)
Snakin/GASA
Block 107
Chr02 36.682–37.341 Mb
RsSNA3
R. soongarica
T. austromongolica
Chr05.479
Chr05 33.112–34.090 Mb
nsLTP
Block 24
Chr01 96.259–99.495 Mb
RsLTP5 RsLTP13
R. soongarica
T. austromongolica
Chr01.1529 Chr01.1530
Chr01 87.189–87.629 Mb
Dots: focal gene positions; open diamonds: additional homology models. Arrows show orientation.
Local rows are scaled independently; reversed coordinate directions are labelled.
