# Supplementary material for: Genome-Wide Identification, Evolutionary Characterization, and Salt-Stress Expression Profiling of Antimicrobial Peptide Gene Families in the Xerophytic Recretohalophyte Reaumuria soongarica
Source: Genes (Basel). 2026 Sep 16;17(9):1126. doi: 10.3390/genes17091126 (PMC13606368; doi:10.3390/genes17091126)
Supplement: Supplementary file 1 [file genes-17-01126-s001.zip › Files/File_S8_catalogue_synced_figures_20260909.zip!/analysis/比较_F8/全部30对_局部共线性核对.pdf]

AMP-involving synteny: all candidate-pair contexts

Primary catalogue · existing-reference anchors · independent genomic scales per row

01 nsLTP | Block 24

Block 24

Chr01 96.260–99.456 Mb

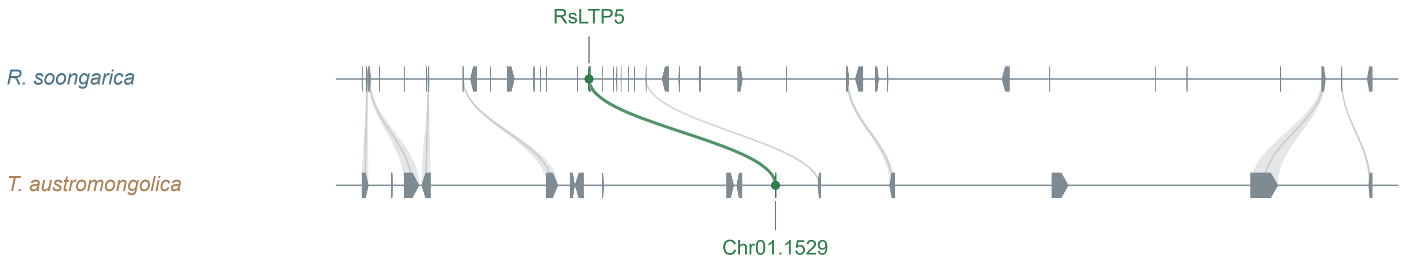

Chr01 87.189–87.629 Mb

Both ends in current catalogue

RSO__gmmChr01G010000.1 / TAU__evm.model.Chr01.1529

02 nsLTP | Block 24

Block 24

Chr01 96.389–99.492 Mb

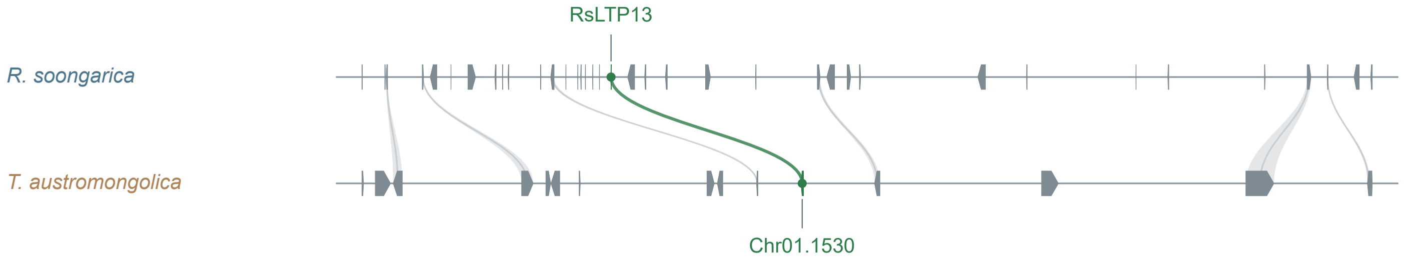

Chr01 87.201–87.629 Mb

Both ends in current catalogue

RSO__gmmChr01G010080.1 / TAU__evm.model.Chr01.1530

03 nsLTP | Block 42

Block 42

Chr01 39.165–40.093 Mb

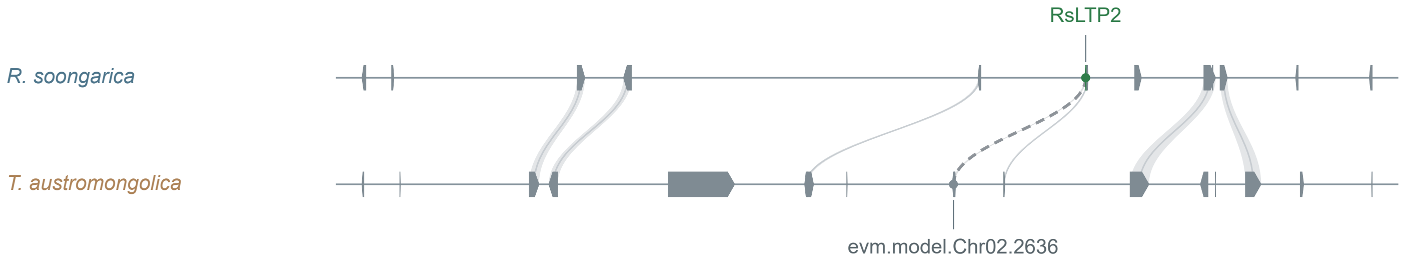

Chr02 135.623–134.746 Mb (reversed)

One endpoint is outside current AMP catalogue

RSO__gmmChr01G005030.1 / TAU__evm.model.Chr02.2636

04 nsLTP | Block 42

Block 42

Chr01 39.191–40.122 Mb

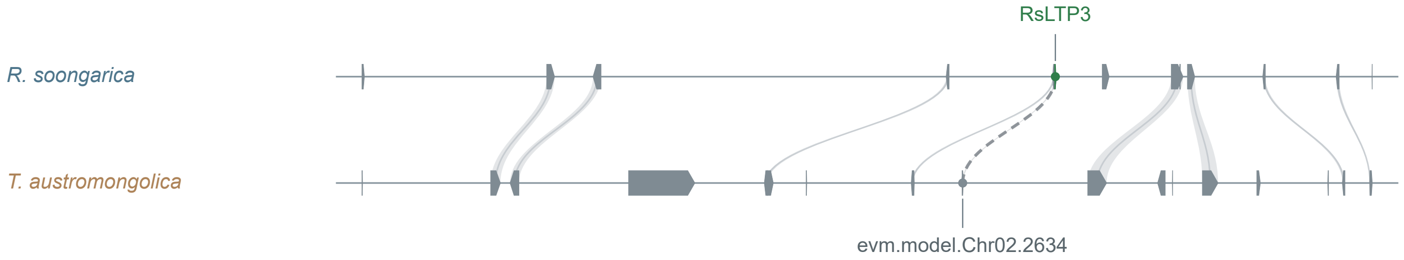

Chr02 135.591–134.710 Mb (reversed)

One endpoint is outside current AMP catalogue

RSO__STRG.2250.1.p1 / TAU__evm.model.Chr02.2634

Grey genes and links provide local context; highlighted genes are the focal pair. Reversed rows are labelled.

AMP-involving synteny: all candidate-pair contexts

Primary catalogue · existing-reference anchors · independent genomic scales per row

05 nsLTP | Block 51

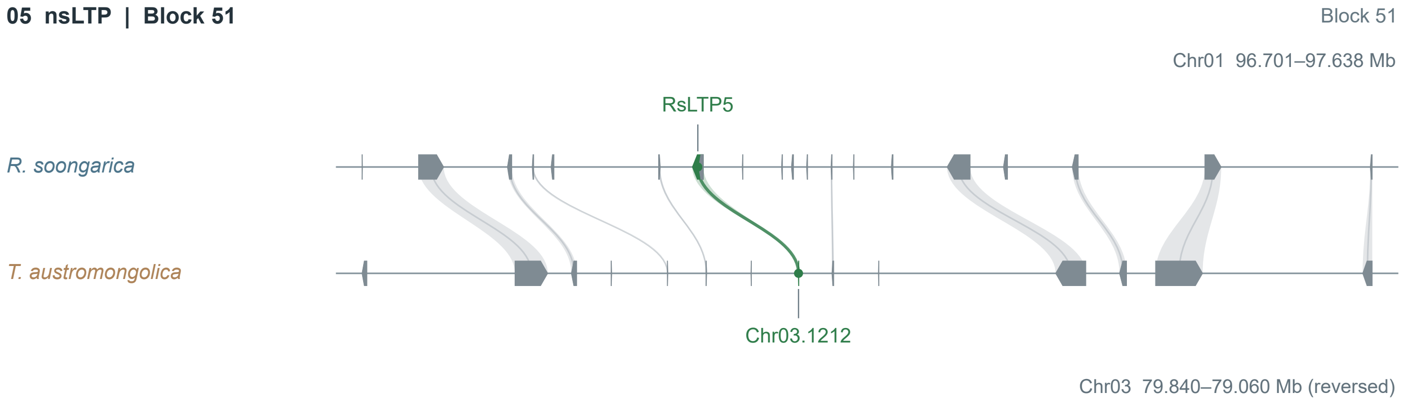

Both ends in current catalogue

RSO__gmmChr01G010000.1 / TAU__evm.model.Chr03.1212

06 nsLTP | Block 51

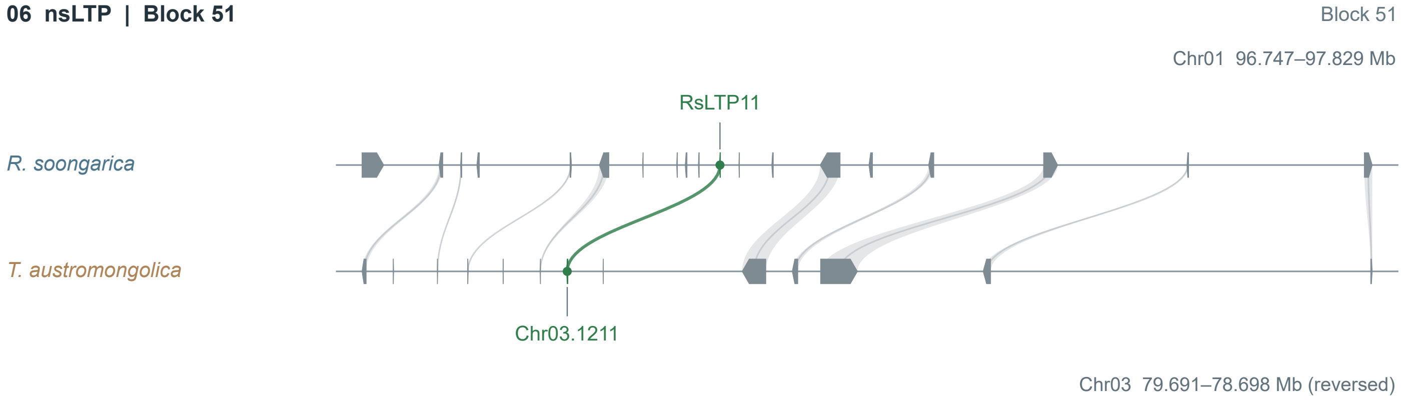

Both ends in current catalogue

RSO__gmmChr01G010060.1 / TAU__evm.model.Chr03.1211

07 nsLTP | Block 102

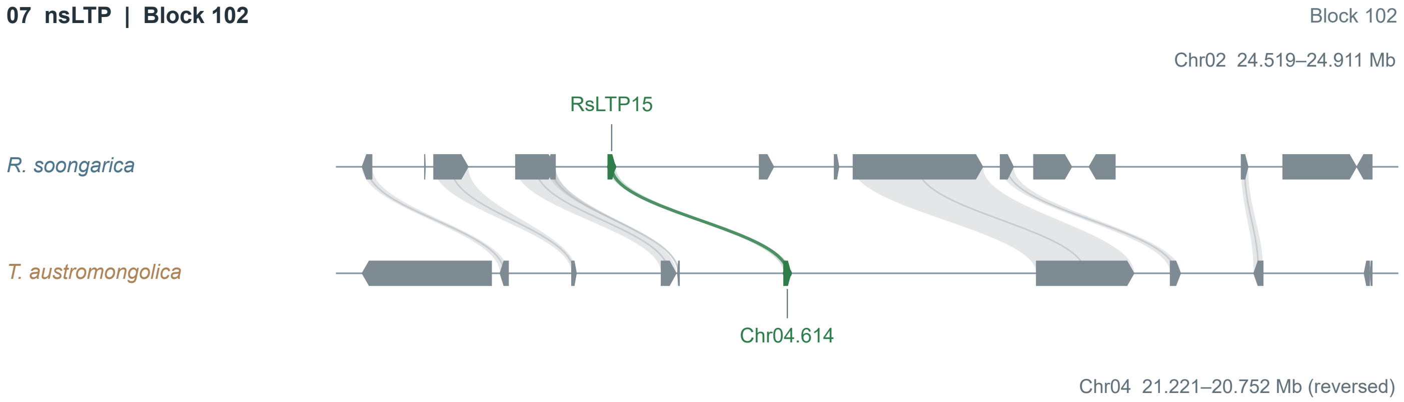

Both ends in current catalogue

RSO__gmmChr02G007880.1 / TAU__evm.model.Chr04.614

08 nsLTP | Block 106

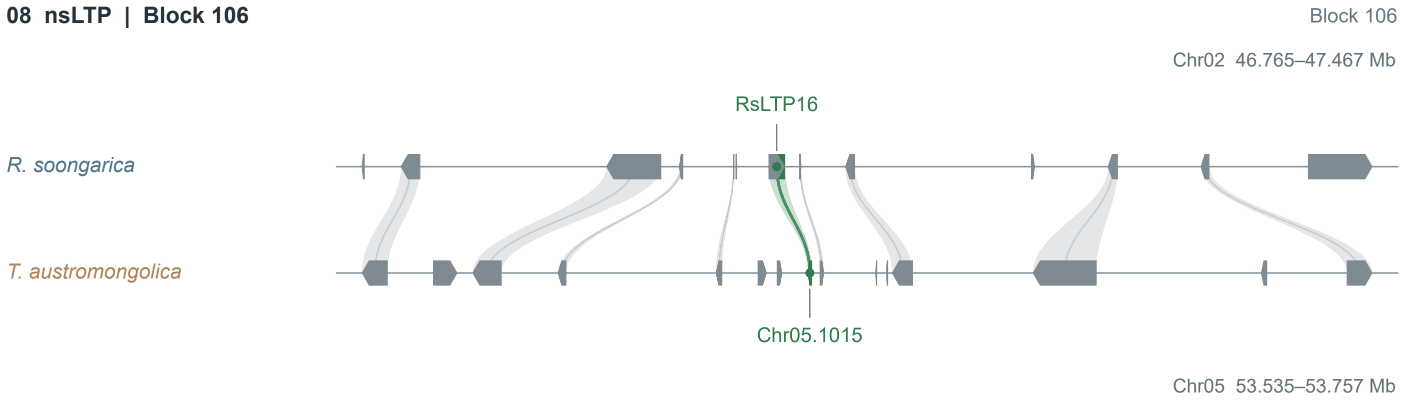

Both ends in current catalogue

RSO__gmmChr02G012770.1 / TAU__evm.model.Chr05.1015

Grey genes and links provide local context; highlighted genes are the focal pair. Reversed rows are labelled.

AMP-involving synteny: all candidate-pair contexts

Primary catalogue · existing-reference anchors · independent genomic scales per row

09 nsLTP | Block 106

Block 106

Chr02 46.788–47.578 Mb

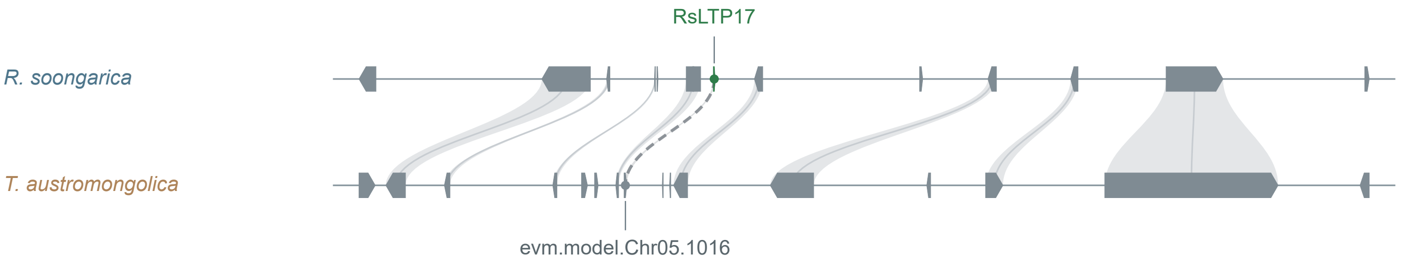

Chr05 53.548–53.871 Mb

One endpoint is outside current AMP catalogue

RSO__STRG.4001.1.p1 / TAU__evm.model.Chr05.1016

10 Snakin/GASA | Block 107

Block 107

Chr02 36.682–37.341 Mb

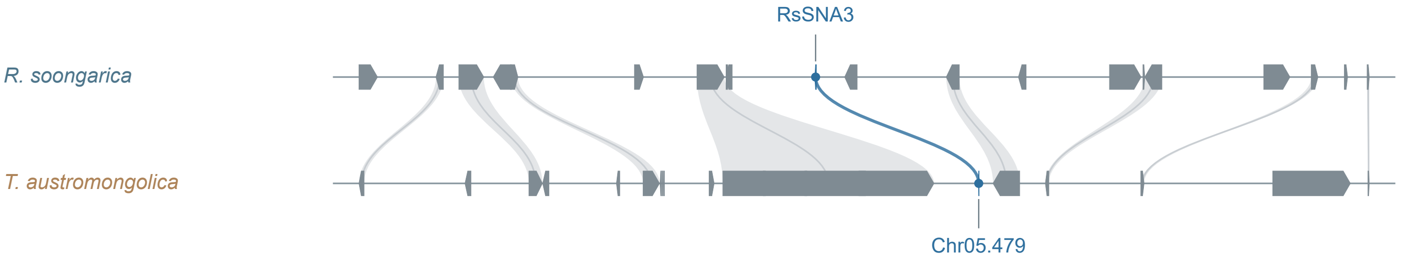

Chr05 33.112–34.090 Mb

Both ends in current catalogue

RSO__STRG.12968.1.p1 / TAU__evm.model.Chr05.479

11 Snakin/GASA | Block 221

Block 221

Chr04 117.952–118.302 Mb

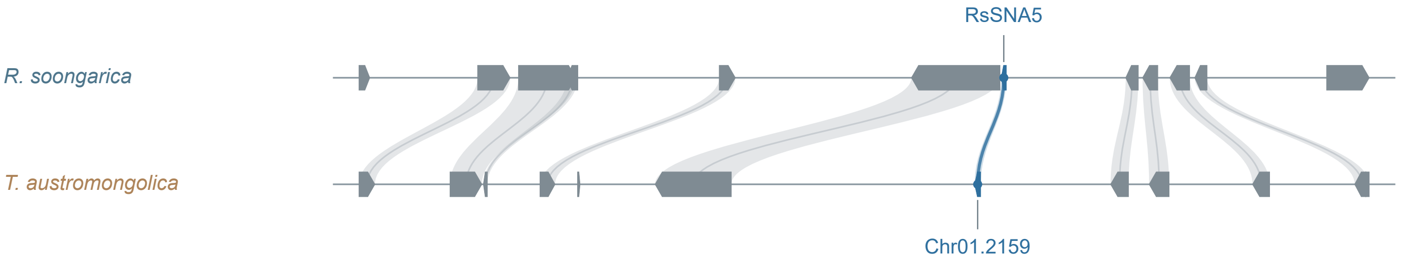

Chr01 110.721–111.018 Mb

Both ends in current catalogue

RSO__MSTRG.7707.1.p1 / TAU__evm.model.Chr01.2159

12 nsLTP | Block 222

Block 222

Chr04 128.337–128.597 Mb

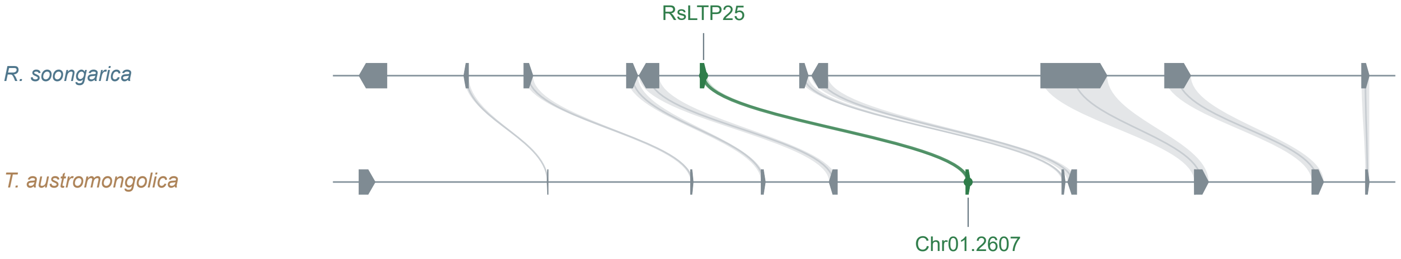

Chr01 128.086–128.640 Mb

Both ends in current catalogue

RSO__STRG.28752.1.p1 / TAU__evm.model.Chr01.2607

Grey genes and links provide local context; highlighted genes are the focal pair. Reversed rows are labelled.

AMP-involving synteny: all candidate-pair contexts

Primary catalogue · existing-reference anchors · independent genomic scales per row

13 Snakin/GASA | Block 223

Block 223

Chr04 85.821–86.365 Mb

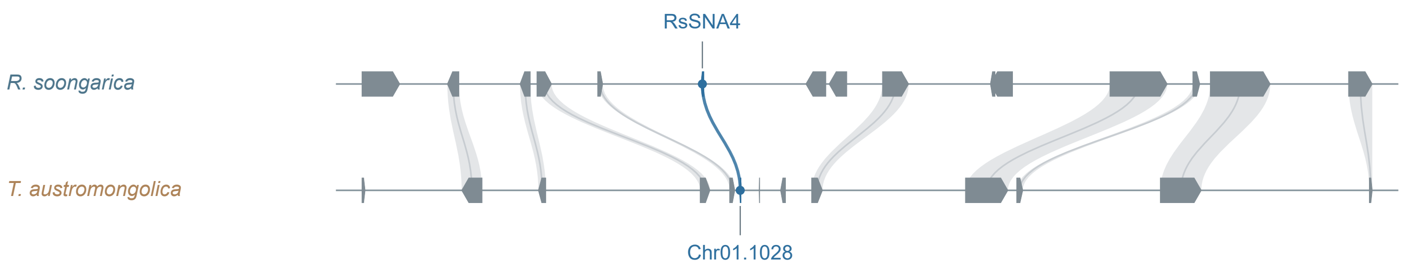

Chr01 67.288–67.828 Mb

Both ends in current catalogue

RSO__STRG.26736.1.p1 / TAU__evm.model.Chr01.1028

14 nsLTP | Block 229

Block 229

Chr04 106.842–107.161 Mb

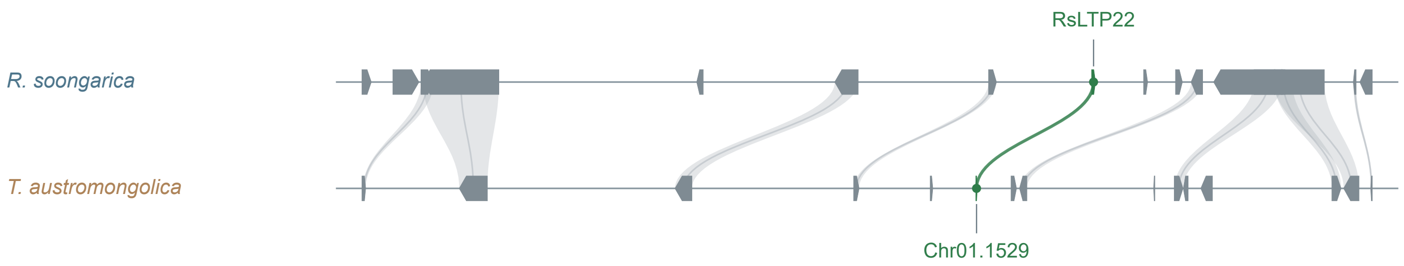

Chr01 87.629–87.201 Mb (reversed)

Both ends in current catalogue

RSO__gmmChr04G014500.2 / TAU__evm.model.Chr01.1529

15 Defensin | Block 230

Block 230

Chr04 120.146–120.373 Mb

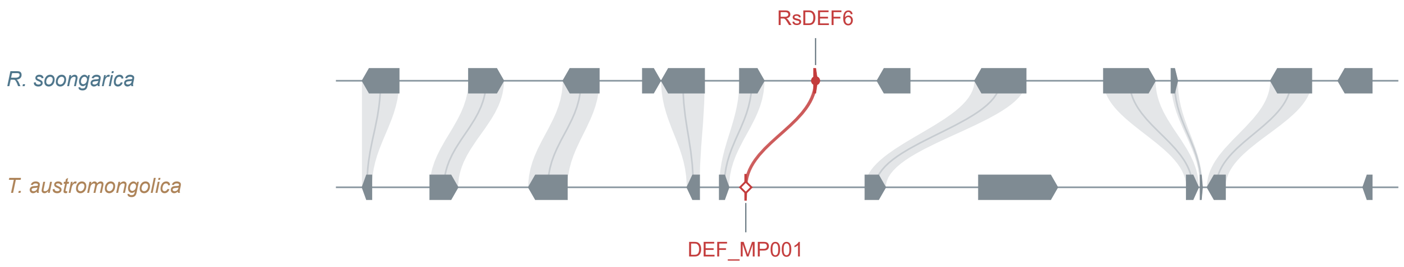

Chr01 123.284–122.746 Mb (reversed)

Both ends in current catalogue

RSO__gmmChr04G018870.1 / TAU__TAU_DEF_MP001

16 Defensin | Block 230

Block 230

Chr04 124.565–124.759 Mb

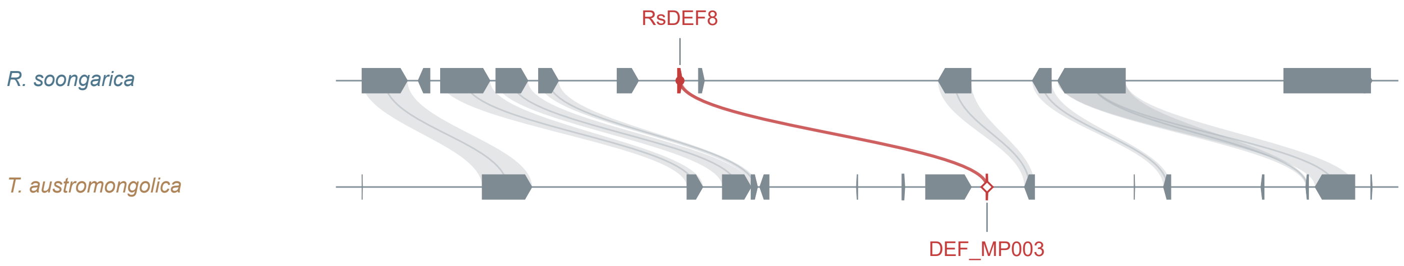

Chr01 115.821–115.280 Mb (reversed)

Both ends in current catalogue

RSO__gmmChr04G020400.1 / TAU__TAU_DEF_MP003

Grey genes and links provide local context; highlighted genes are the focal pair. Reversed rows are labelled.

AMP-involving synteny: all candidate-pair contexts

Primary catalogue · existing-reference anchors · independent genomic scales per row

17 nsLTP | Block 239

Block 239

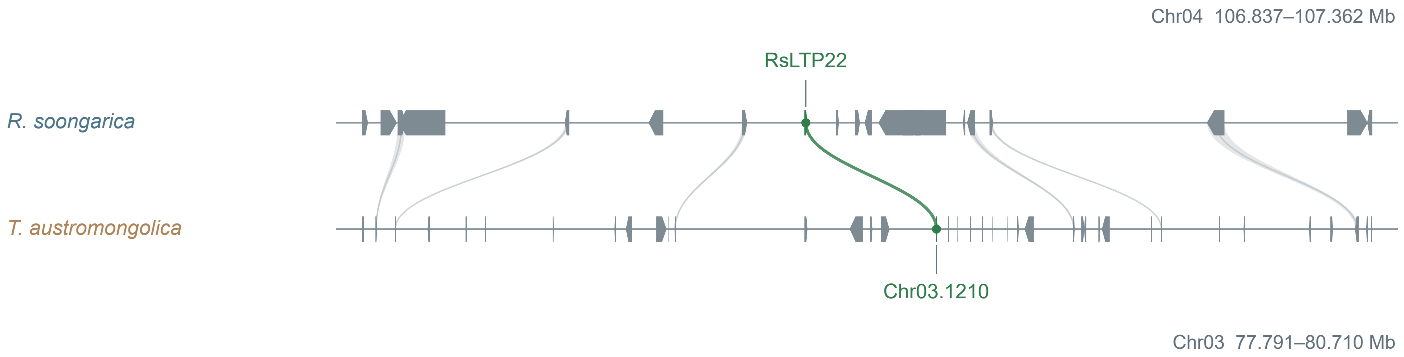

Both ends in current catalogue

RSO__gmmChr04G014500.2 / TAU__evm.model.Chr03.1210

18 nsLTP | Block 283

Block 283

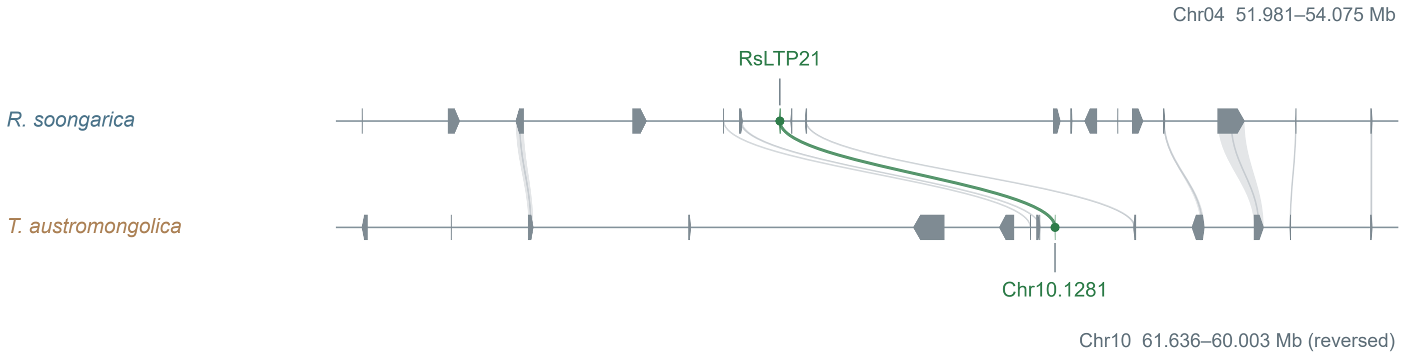

Both ends in current catalogue

RSO__gmmChr04G004390.1 / TAU__evm.model.Chr10.1281

19 Snakin/GASA | Block 406

Block 406

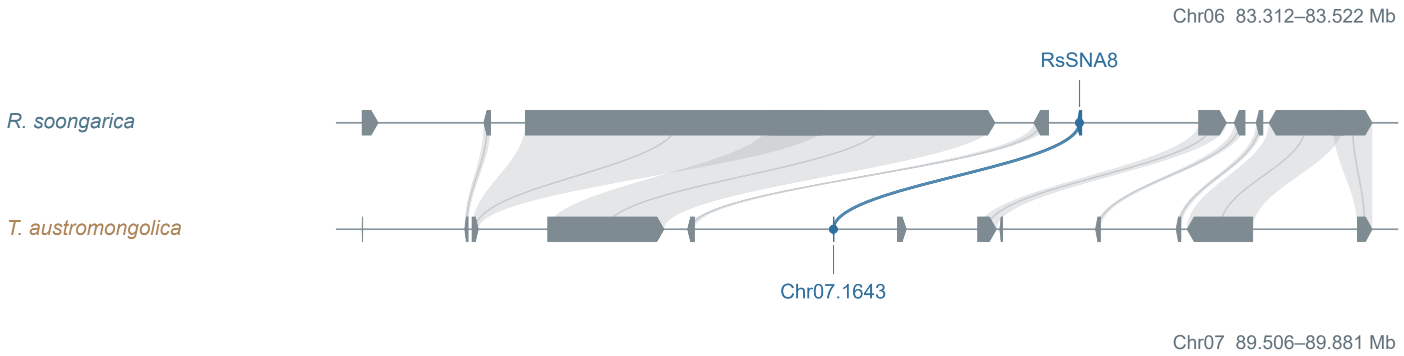

Both ends in current catalogue

RSO__STRG.16502.1.p1 / TAU__evm.model.Chr07.1643

20 nsLTP | Block 468

Block 468

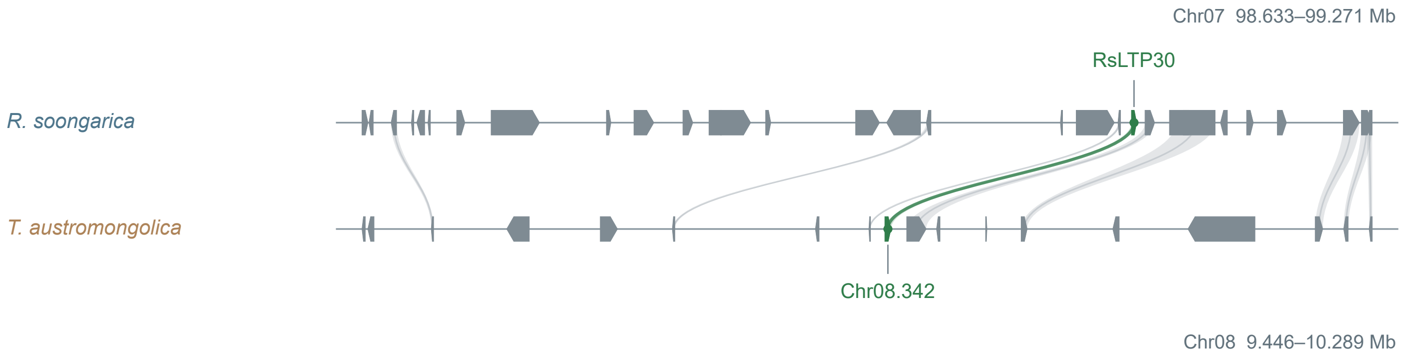

Both ends in current catalogue

RSO__STRG.19776.1.p1 / TAU__evm.model.Chr08.342

Grey genes and links provide local context; highlighted genes are the focal pair. Reversed rows are labelled.

AMP-involving synteny: all candidate-pair contexts

Primary catalogue · existing-reference anchors · independent genomic scales per row

21 nsLTP | Block 489

Block 489

Chr07 98.956–99.307 Mb

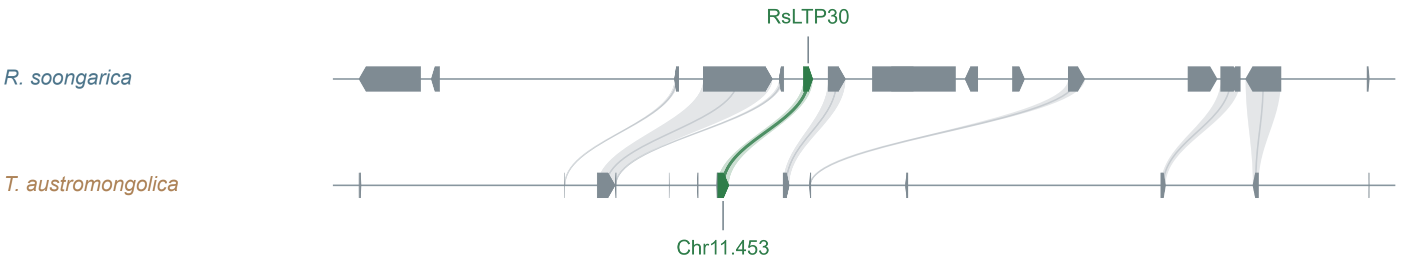

Chr11 23.470–21.613 Mb (reversed)

Both ends in current catalogue

RSO__STRG.19776.1.p1 / TAU__evm.model.Chr11.453

22 Snakin/GASA | Block 516

Block 516

Chr08 24.203–25.752 Mb

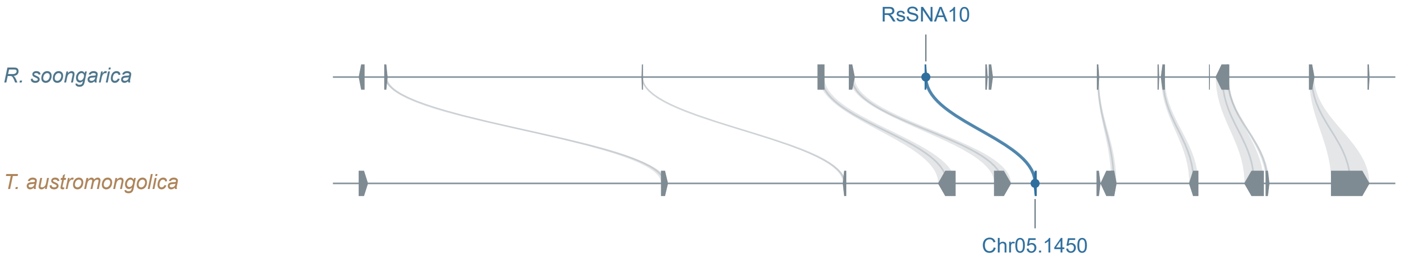

Chr05 68.649–68.142 Mb (reversed)

Both ends in current catalogue

RSO__gmmChr08G004090.1 / TAU__evm.model.Chr05.1450

23 Snakin/GASA | Block 529

Block 529

Chr08 116.436–116.768 Mb

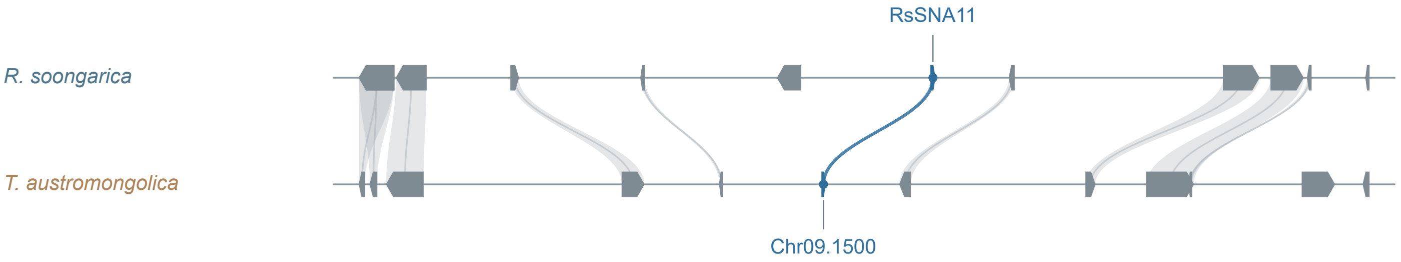

Chr09 89.969–90.261 Mb

Both ends in current catalogue

RSO__gmmChr08G014630.1 / TAU__evm.model.Chr09.1500

24 nsLTP | Block 548

Block 548

Chr08 55.903–56.990 Mb

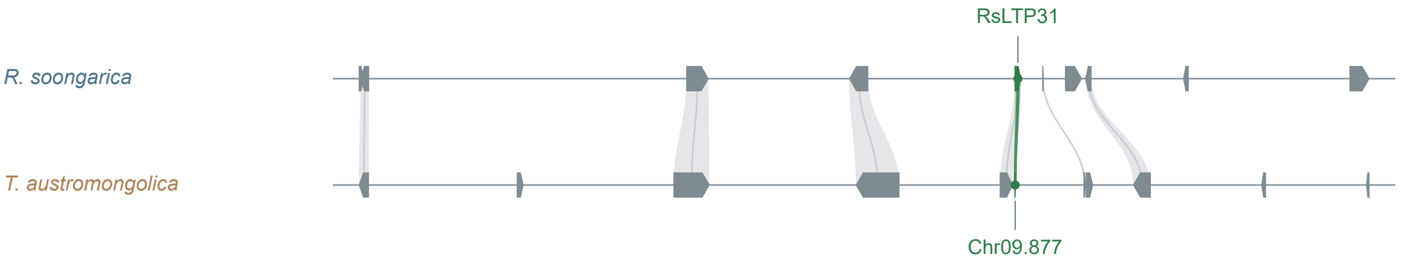

Chr09 64.636–64.177 Mb (reversed)

Both ends in current catalogue

RSO__gmmChr08G007020.1 / TAU__evm.model.Chr09.877

Grey genes and links provide local context; highlighted genes are the focal pair. Reversed rows are labelled.

AMP-involving synteny: all candidate-pair contexts

Primary catalogue · existing-reference anchors · independent genomic scales per row

25 nsLTP | Block 585

Block 585

Chr09 7.475–9.098 Mb

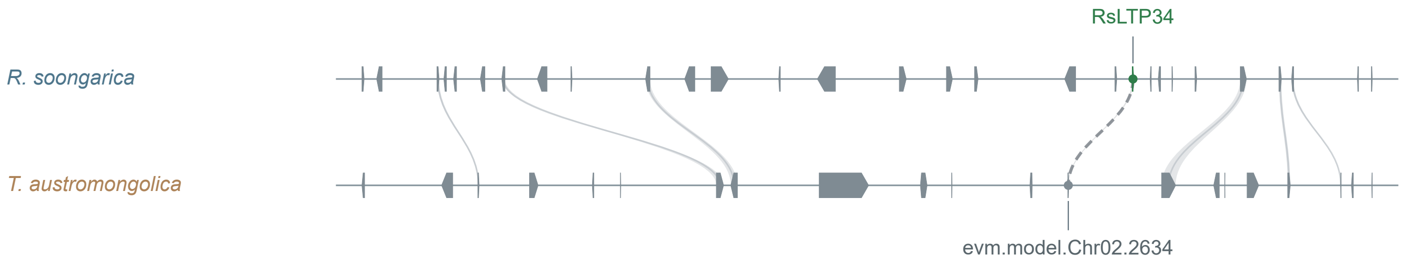

Chr02 135.887–134.704 Mb (reversed)

One endpoint is outside current AMP catalogue

RSO__STRG.16875.1.p1 / TAU__evm.model.Chr02.2634

26 nsLTP | Block 594

Block 594

Chr09 23.407–24.838 Mb

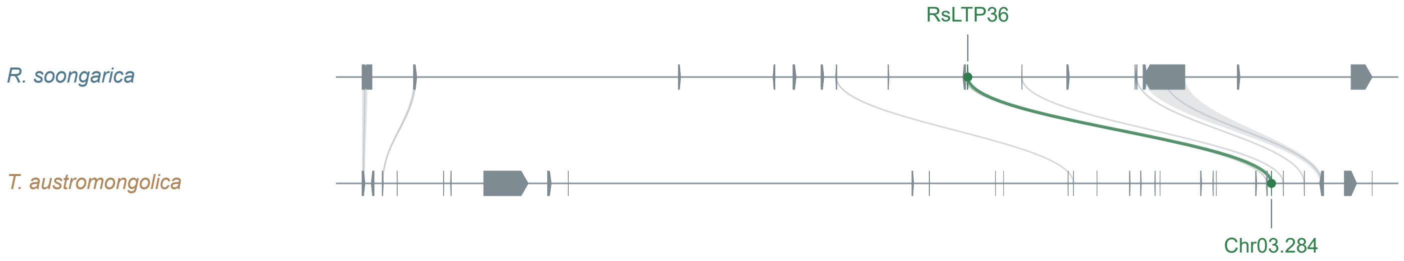

Chr03 13.298–10.710 Mb (reversed)

Both ends in current catalogue

RSO__gmmChr09G002740.1 / TAU__evm.model.Chr03.284

27 nsLTP | Block 594

Block 594

Chr09 25.510–27.881 Mb

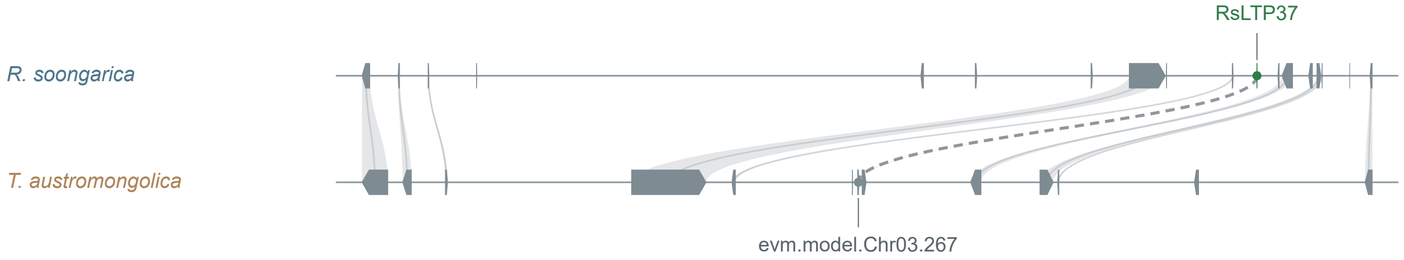

Chr03 10.468–9.902 Mb (reversed)

One endpoint is outside current AMP catalogue

RSO__STRG.16931.1.p1 / TAU__evm.model.Chr03.267

28 nsLTP | Block 595

Block 595

Chr09 8.189–9.081 Mb

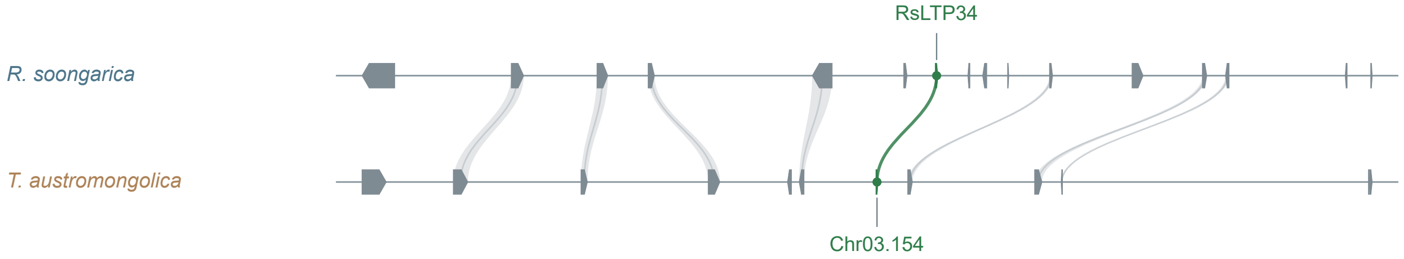

Chr03 5.496–5.042 Mb (reversed)

Both ends in current catalogue

RSO__STRG.16875.1.p1 / TAU__evm.model.Chr03.154

Grey genes and links provide local context; highlighted genes are the focal pair. Reversed rows are labelled.

AMP-involving synteny: all candidate-pair contexts

Primary catalogue · existing-reference anchors · independent genomic scales per row

29 nsLTP | Block 674

Block 674

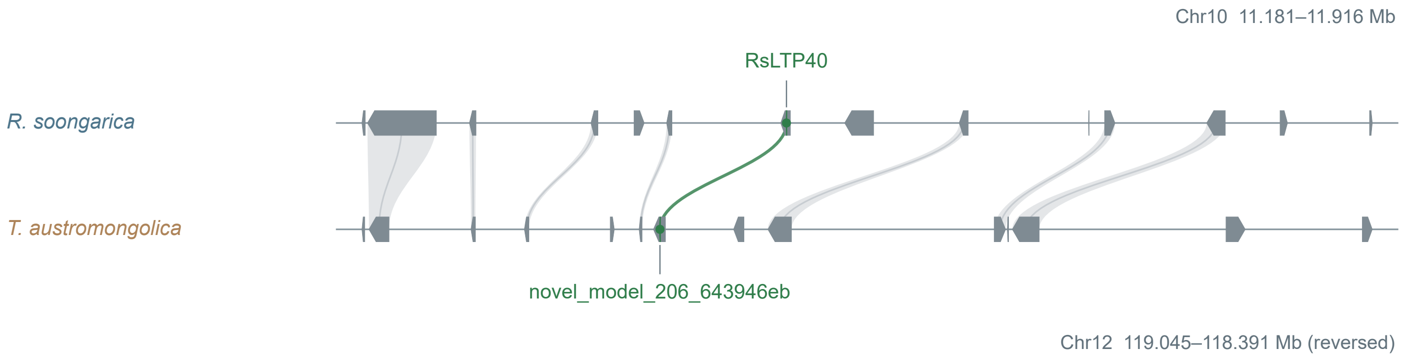

Both ends in current catalogue

RSO__STRG.29675.1.p1 / TAU__novel_model_206_643946eb

30 Snakin/GASA | Block 693

Block 693

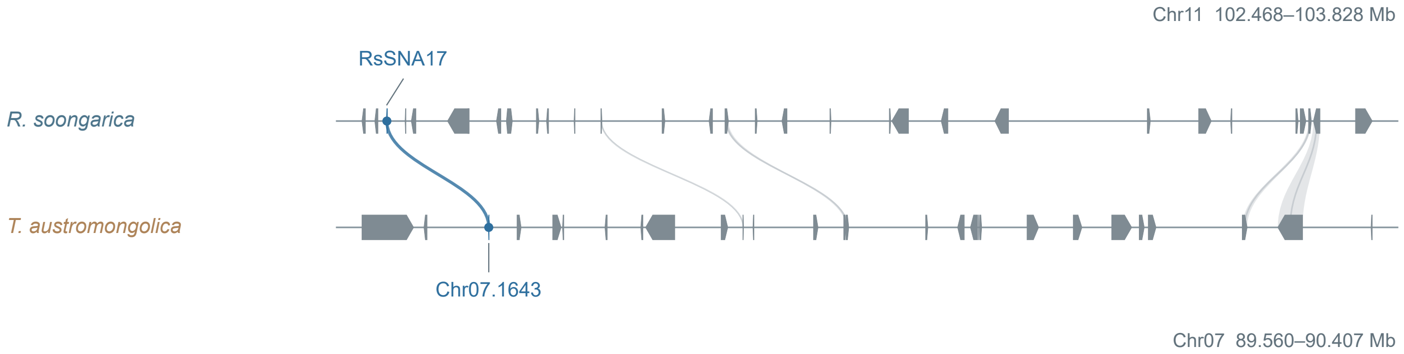

Both ends in current catalogue

RSO__STRG.31259.1.p2 / TAU__evm.model.Chr07.1643

Grey genes and links provide local context; highlighted genes are the focal pair. Reversed rows are labelled.
