# Supplementary material for: Genome-Wide Identification, Evolutionary Characterization, and Salt-Stress Expression Profiling of Antimicrobial Peptide Gene Families in the Xerophytic Recretohalophyte Reaumuria soongarica
Source: Genes (Basel). 2026 Sep 16;17(9):1126. doi: 10.3390/genes17091126 (PMC13606368; doi:10.3390/genes17091126)
Supplement: Supplementary file 1 [file genes-17-01126-s001.zip › Files/File_S8_catalogue_synced_figures_20260909.zip!/analysis/注释_F1_F3_F4/Figure3_primary73_gene_structure.pdf]

Gene structures of 73 primary-catalogue members

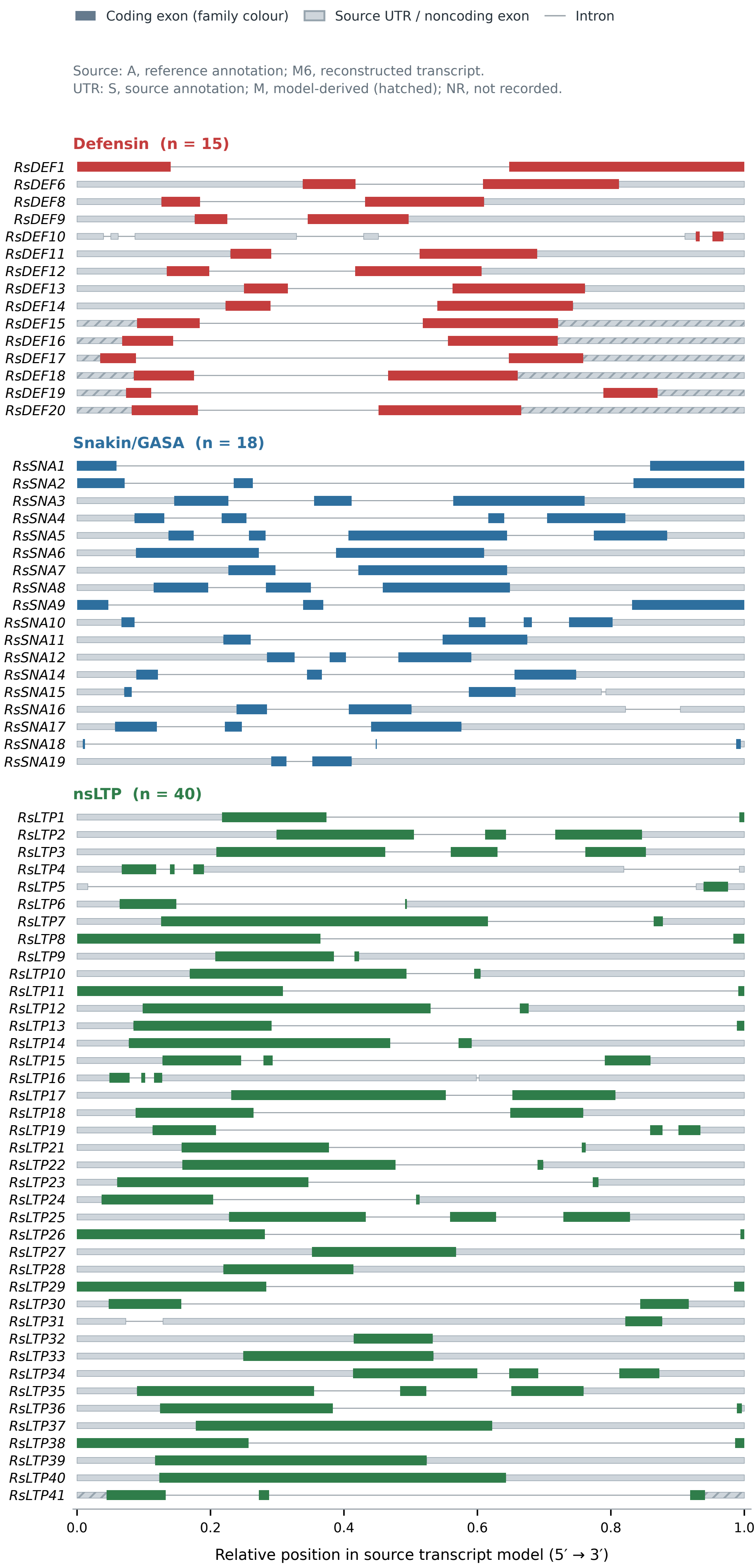

Defensin (n = 15)

	Span (bp)	Source	UTR
RsDEF1	457	A	NR
RsDEF6	851	A	S
RsDEF8	956	A	S
RsDEF9	1,128	A	S
RsDEF10	10,590	A	S
RsDEF11	1,001	A	S
RsDEF12	868	A	S
RsDEF13	842	A	S
RsDEF14	822	A	S
RsDEF15	810	M6	M
RsDEF16	1,000	M6	M
RsDEF17	1,474	M6	M
RsDEF18	845	M6	M
RsDEF19	2,022	M6	M
RsDEF20	768	M6	M

Snakin/GASA (n = 18)

	Span (bp)	Source	UTR
RsSNA1	1,290	A	NR
RsSNA2	1,098	A	NR
RsSNA3	926	A	S
RsSNA4	1,556	A	S
RsSNA5	1,685	A	S
RsSNA6	1,028	A	S
RsSNA7	1,026	A	S
RsSNA8	956	A	S
RsSNA9	1,030	A	NR
RsSNA10	2,810	A	S
RsSNA11	1,436	A	S
RsSNA12	1,671	A	S
RsSNA14	1,968	A	S
RsSNA15	4,131	A	S
RsSNA16	2,005	A	S
RsSNA17	1,349	A	S
RsSNA18	27,687	A	S
RsSNA19	3,093	A	S

nsLTP (n = 40)

	Span (bp)	Source	UTR
RsLTP1	2,201	A	S
RsLTP2	1,726	A	S
RsLTP3	1,286	A	S
RsLTP4	6,081	A	S
RsLTP5	9,777	A	S
RsLTP6	4,175	A	S
RsLTP7	752	A	S
RsLTP8	985	A	NR
RsLTP9	1,986	A	S
RsLTP10	1,060	A	S
RsLTP11	1,116	A	NR
RsLTP12	791	A	S
RsLTP13	1,692	A	S
RsLTP14	876	A	S
RsLTP15	2,897	A	S
RsLTP16	10,953	A	S
RsLTP17	1,339	A	S
RsLTP18	1,942	A	S
RsLTP19	4,246	A	S
RsLTP21	1,670	A	S
RsLTP22	1,152	A	S
RsLTP23	1,212	A	S
RsLTP24	2,060	A	S
RsLTP25	1,704	A	S
RsLTP26	1,255	A	NR
RsLTP27	1,365	A	S
RsLTP28	1,464	A	S
RsLTP29	1,104	A	NR
RsLTP30	3,129	A	S
RsLTP31	6,200	A	S
RsLTP32	2,409	A	S
RsLTP33	1,011	A	S
RsLTP34	2,297	A	S
RsLTP35	1,158	A	S
RsLTP36	1,331	A	S
RsLTP37	690	A	S
RsLTP38	1,374	A	NR
RsLTP39	811	A	S
RsLTP40	624	A	S
RsLTP41	3,510	M6	M

Models are normalized separately; unrecorded UTRs are not inferred absent. Terminal stop codons are included in coloured blocks.
