# Supplementary material for: Genome-Wide Identification, Evolutionary Characterization, and Salt-Stress Expression Profiling of Antimicrobial Peptide Gene Families in the Xerophytic Recretohalophyte Reaumuria soongarica
Source: Genes (Basel). 2026 Sep 16;17(9):1126. doi: 10.3390/genes17091126 (PMC13606368; doi:10.3390/genes17091126)
Supplement: Supplementary file 1 [file genes-17-01126-s001.zip › Files/File_S8_catalogue_synced_figures_20260909.zip!/analysis/注释_F1_F3_F4_紧凑排版/Figure3_primary73_compact.pdf]

Gene structures | 73 primary members

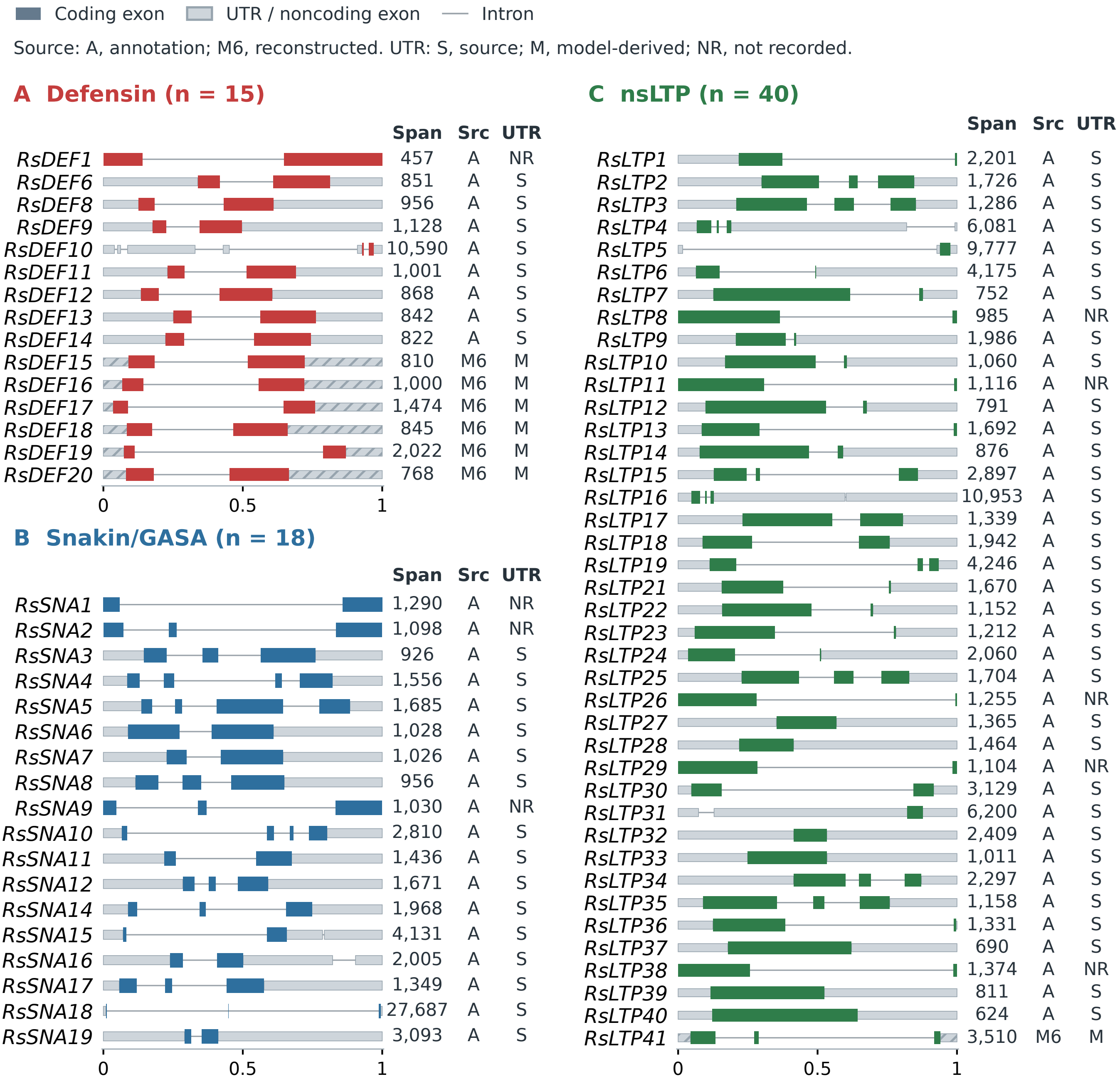

Position is normalized separately for each full source transcript (5′ → 3′); span is in bp.

M: inferred noncoding exon portions (hatched), without an independently annotated UTR or TSS.

NR does not imply UTR absence. Coding blocks include the terminal stop codon.
