# Supplementary material for: Genome-Wide Identification, Evolutionary Characterization, and Salt-Stress Expression Profiling of Antimicrobial Peptide Gene Families in the Xerophytic Recretohalophyte Reaumuria soongarica
Source: Genes (Basel). 2026 Sep 16;17(9):1126. doi: 10.3390/genes17091126 (PMC13606368; doi:10.3390/genes17091126)
Supplement: Supplementary file 1 [file genes-17-01126-s001.zip › Files/File_S8_catalogue_synced_figures_20260909.zip!/analysis/注释_F1_F3_F4_紧凑排版/Figure4_primary73_compact.pdf]

MEME motifs | 73 primary proteins

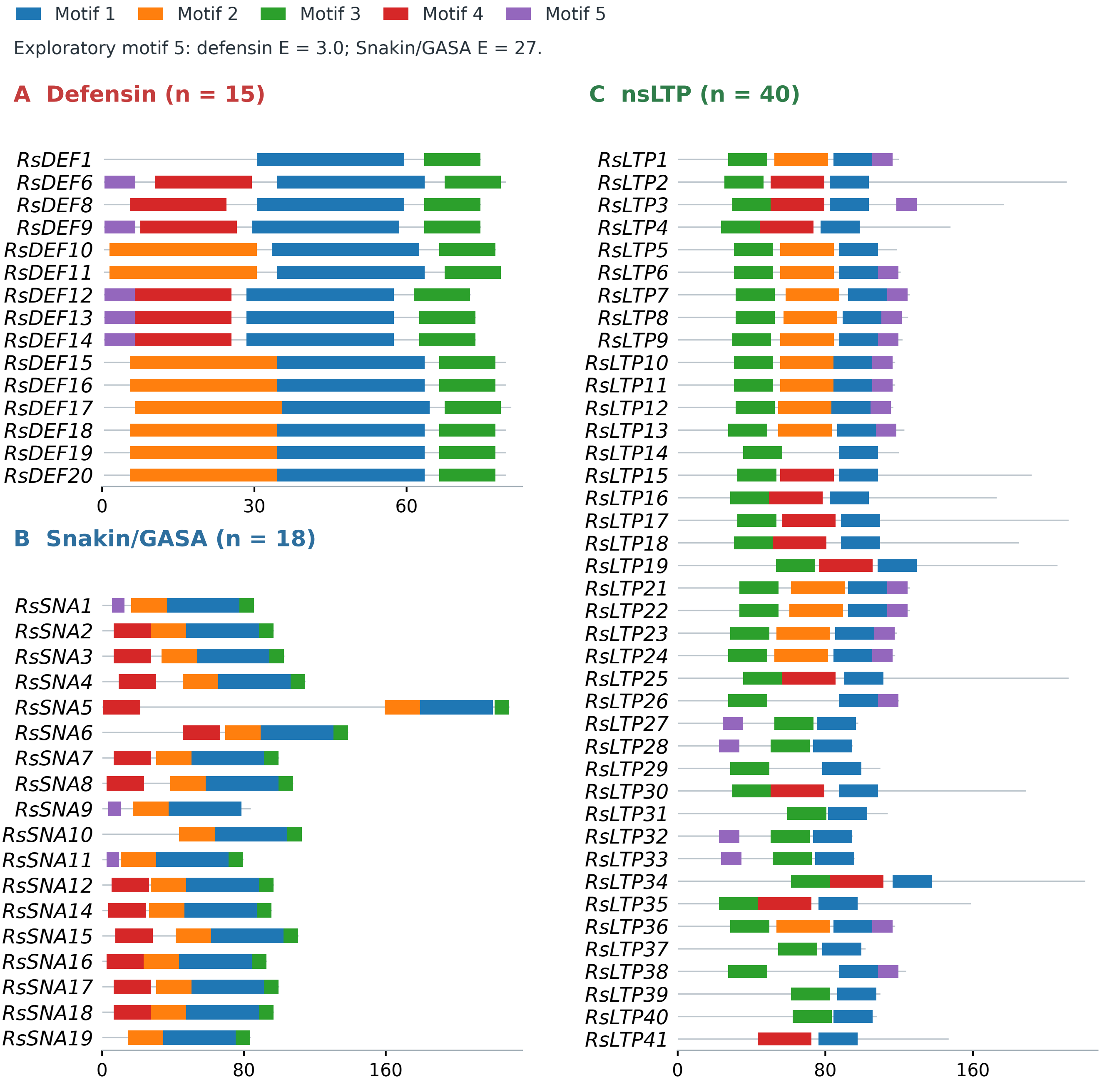

Axes show full-precursor positions in amino acids; complete proteins are retained.

All five requested motifs per family are shown (site P ≤ 10^{-4}); weak motif E-values are flagged above.

Ranks and colours are family-specific and do not imply motif homology between families.
