# Supplementary material for: Genome-Wide Identification, Evolutionary Characterization, and Salt-Stress Expression Profiling of Antimicrobial Peptide Gene Families in the Xerophytic Recretohalophyte Reaumuria soongarica
Source: Genes (Basel). 2026 Sep 16;17(9):1126. doi: 10.3390/genes17091126 (PMC13606368; doi:10.3390/genes17091126)
Supplement: Supplementary file 1 [file genes-17-01126-s001.zip › Files/File_S8_catalogue_synced_figures_20260909.zip!/analysis/系统树_F2/图件/Support_audit/Vector_PDF/Support_audit_C_nsLTP_all_nodes.pdf]

(C) nsLTP | all available SH-aLRT / UFBoot (%)

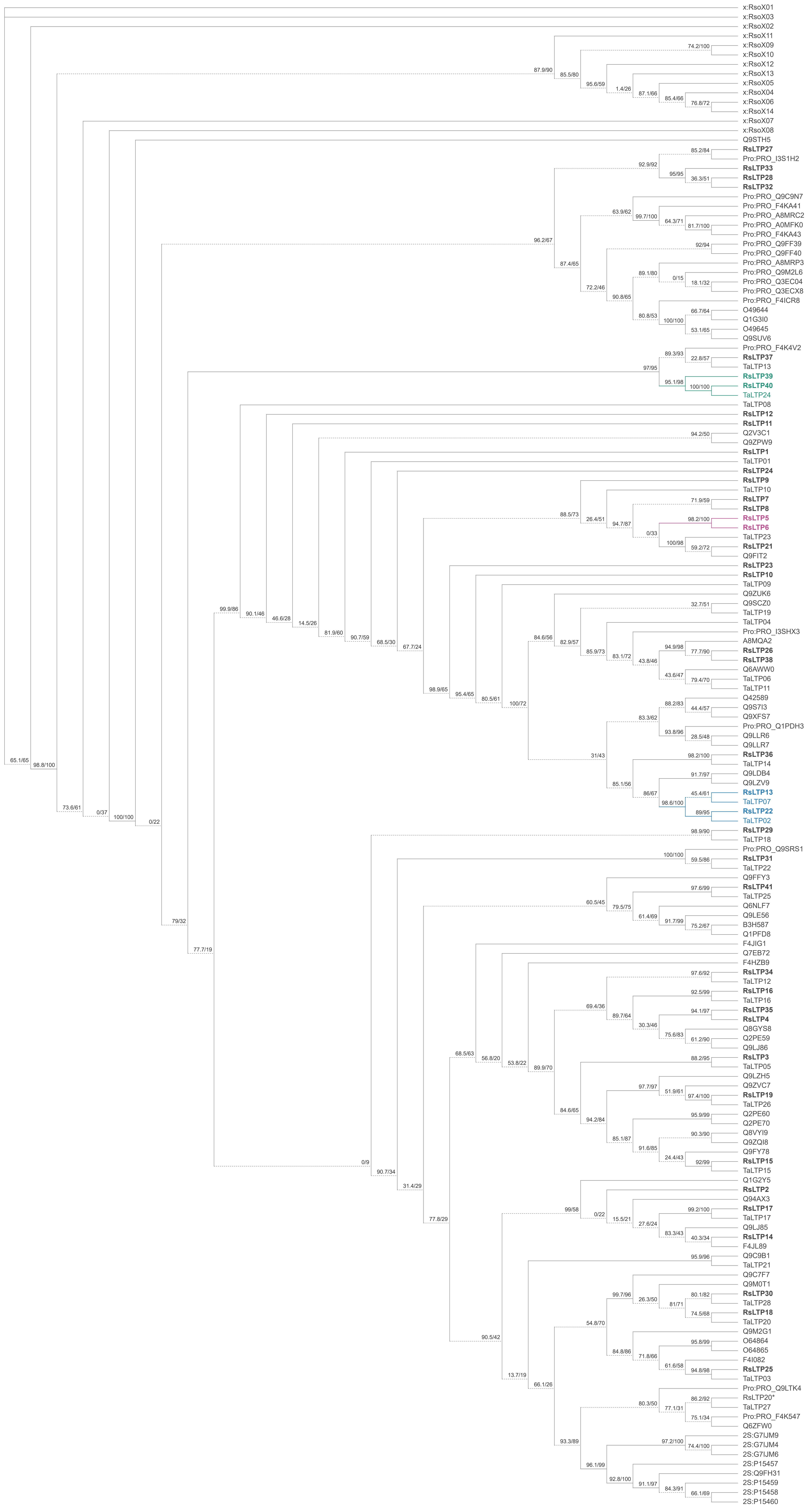

Support audit only. Cladogram lengths are not evolutionary distances; the display origin is not a biological root.
