# Supplementary material for: Genome-Wide Identification, Evolutionary Characterization, and Salt-Stress Expression Profiling of Antimicrobial Peptide Gene Families in the Xerophytic Recretohalophyte Reaumuria soongarica
Source: Genes (Basel). 2026 Sep 16;17(9):1126. doi: 10.3390/genes17091126 (PMC13606368; doi:10.3390/genes17091126)
Supplement: Supplementary file 1 [file genes-17-01126-s001.zip › Files/File_S8_catalogue_synced_figures_20260909.zip!/analysis/系统树_F2/图件/Vector_PDF/Figure2A_Defensin_primary73_key_clades.pdf]

(A) Defensin | 23 sequences

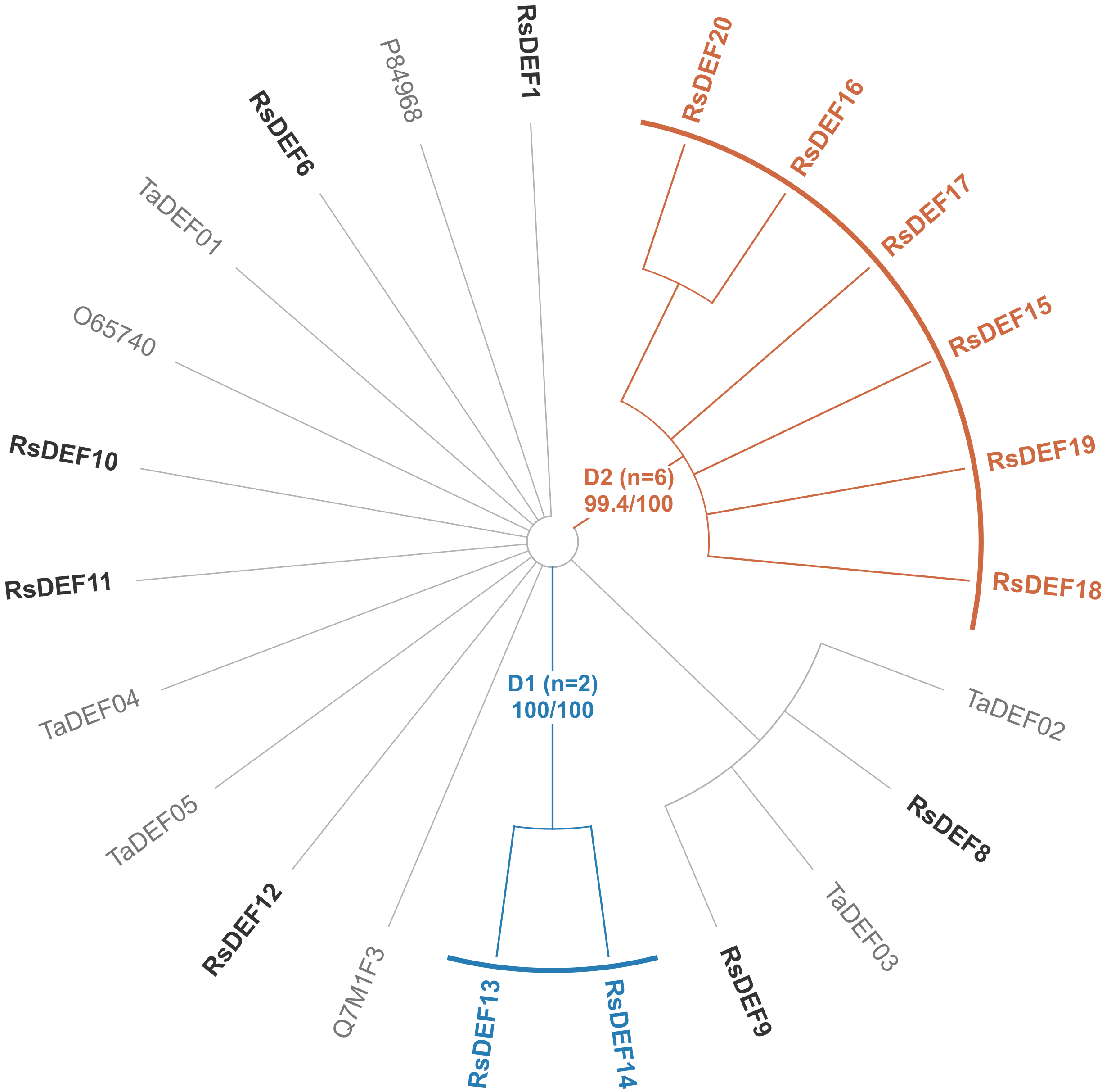

Bold: primary R. soongarica catalogue *: extended RsLTP20 x: historical exclusion

n: all sequences in group | SH-aLRT / UFBoot (%) | Groups are not formal subfamilies

Circular cladogram; radial lengths not to scale; centre is not a biological root
