# Supplementary material for: Genome-Wide Identification, Evolutionary Characterization, and Salt-Stress Expression Profiling of Antimicrobial Peptide Gene Families in the Xerophytic Recretohalophyte Reaumuria soongarica
Source: Genes (Basel). 2026 Sep 16;17(9):1126. doi: 10.3390/genes17091126 (PMC13606368; doi:10.3390/genes17091126)
Supplement: Supplementary file 1 [file genes-17-01126-s001.zip › Files/File_S8_catalogue_synced_figures_20260909.zip!/analysis/表达_F6_F7/figures/Figure6_expression_heatmap_52.pdf]

## Expression profiles of reference-covered AMP genes

52 count-filtered members across nine RNA-seq libraries

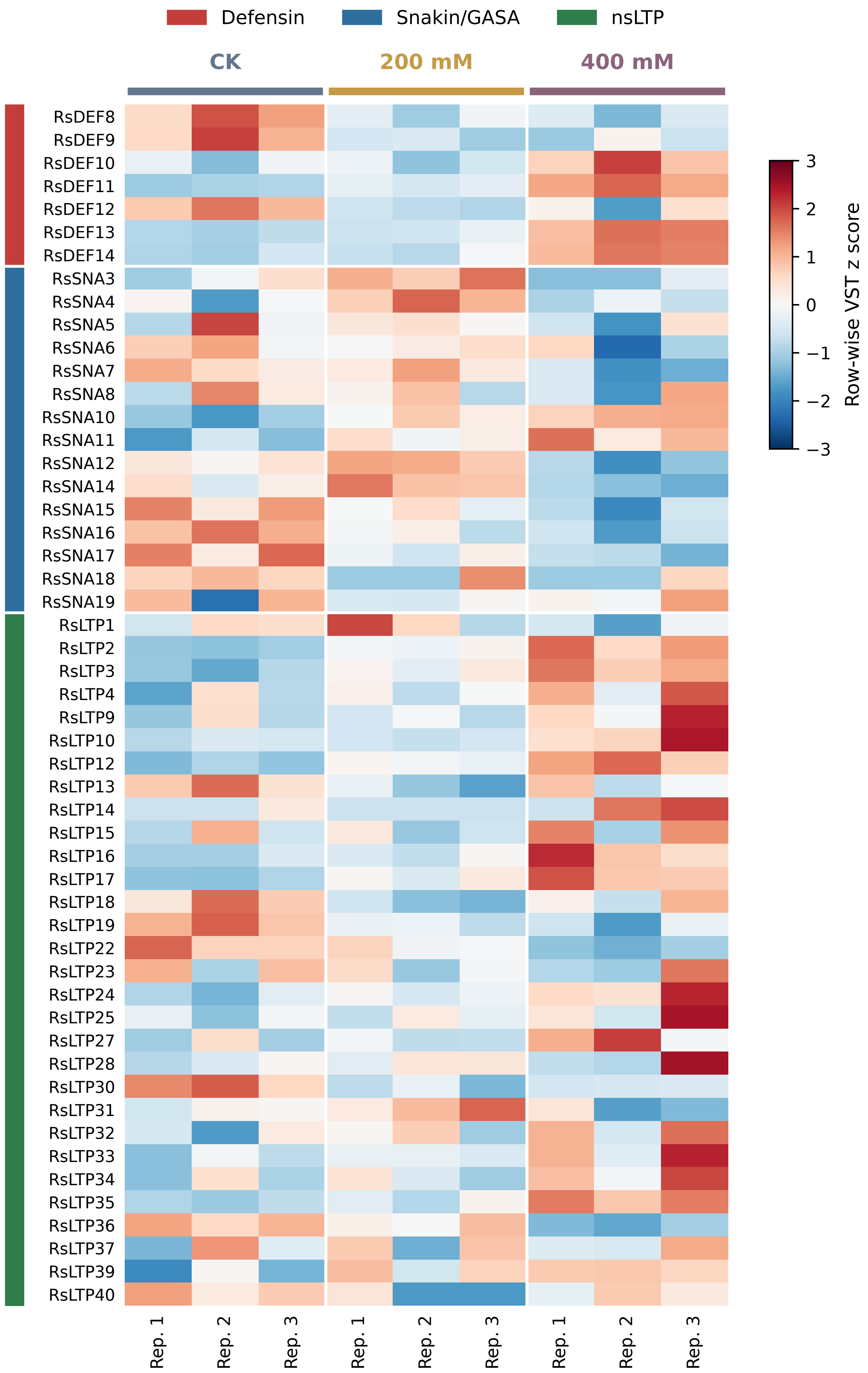


73 primary members: 66 in the original reference, including 52 passing the count filter.
Seven new models were not quantified; no missing values were imputed.
