# Supplementary material for: Genome-Wide Identification, Evolutionary Characterization, and Salt-Stress Expression Profiling of Antimicrobial Peptide Gene Families in the Xerophytic Recretohalophyte Reaumuria soongarica
Source: Genes (Basel). 2026 Sep 16;17(9):1126. doi: 10.3390/genes17091126 (PMC13606368; doi:10.3390/genes17091126)
Supplement: Supplementary file 1 [file genes-17-01126-s001.zip › Files/File_S8_catalogue_synced_figures_20260909.zip!/analysis/表达_F6_F7/figures/Figure7_apeglm_display_sensitivity.pdf]

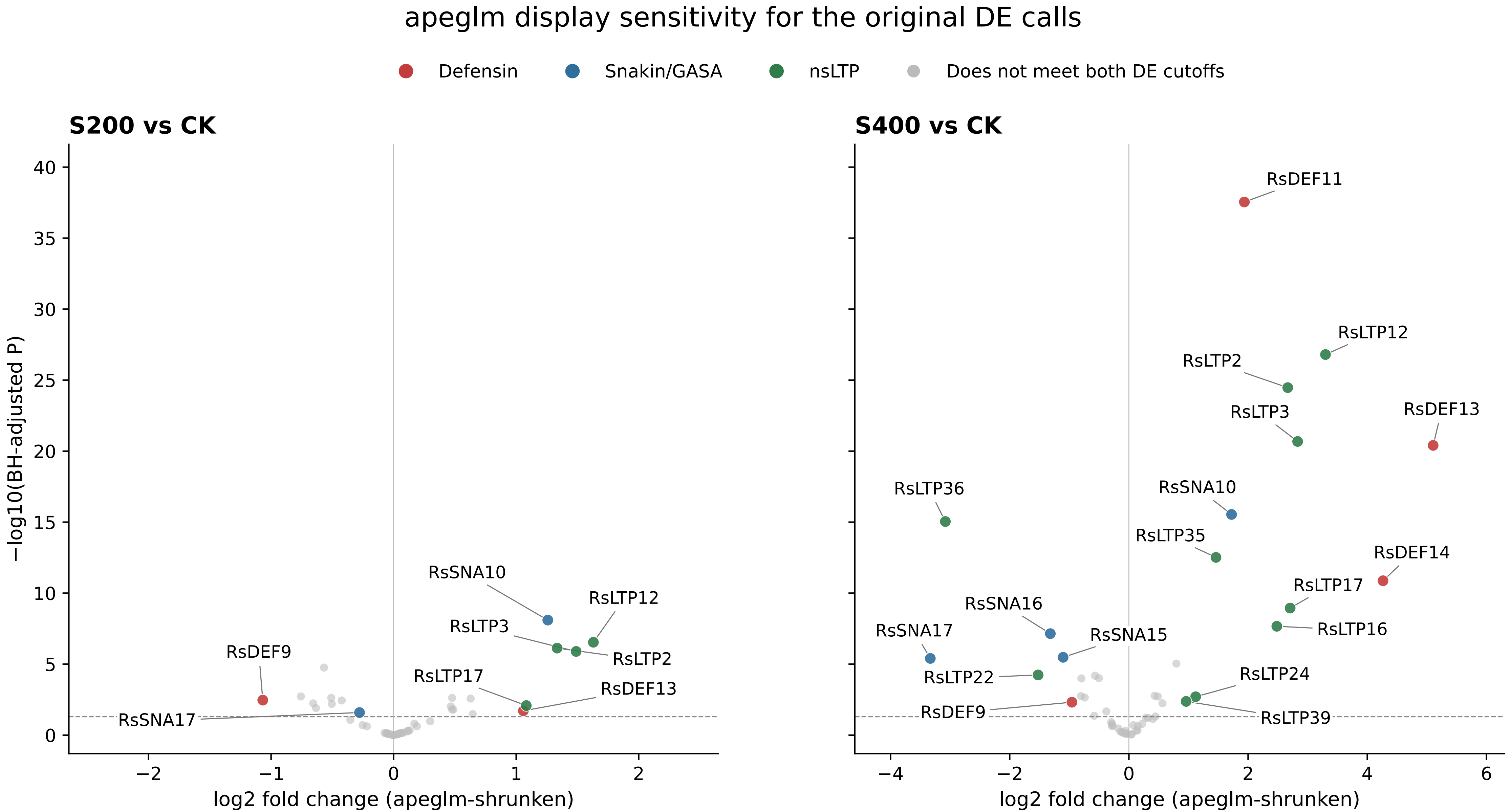

Colors and labels use the unchanged original BH-adjusted P and MLE fold-change criteria.
No |shrunken log2FC| cutoff is applied; this is a display sensitivity figure, not a new DE analysis.
