# Supplementary material for: Genome-Wide Identification, Evolutionary Characterization, and Salt-Stress Expression Profiling of Antimicrobial Peptide Gene Families in the Xerophytic Recretohalophyte Reaumuria soongarica
Source: Genes (Basel). 2026 Sep 16;17(9):1126. doi: 10.3390/genes17091126 (PMC13606368; doi:10.3390/genes17091126)
Supplement: Supplementary file 1 [file genes-17-01126-s001.zip › Files/File_S8_catalogue_synced_figures_20260909.zip!/analysis/表达_F6_F7/figures/Figure7_expression_volcano_all_DE_labeled_66.pdf]

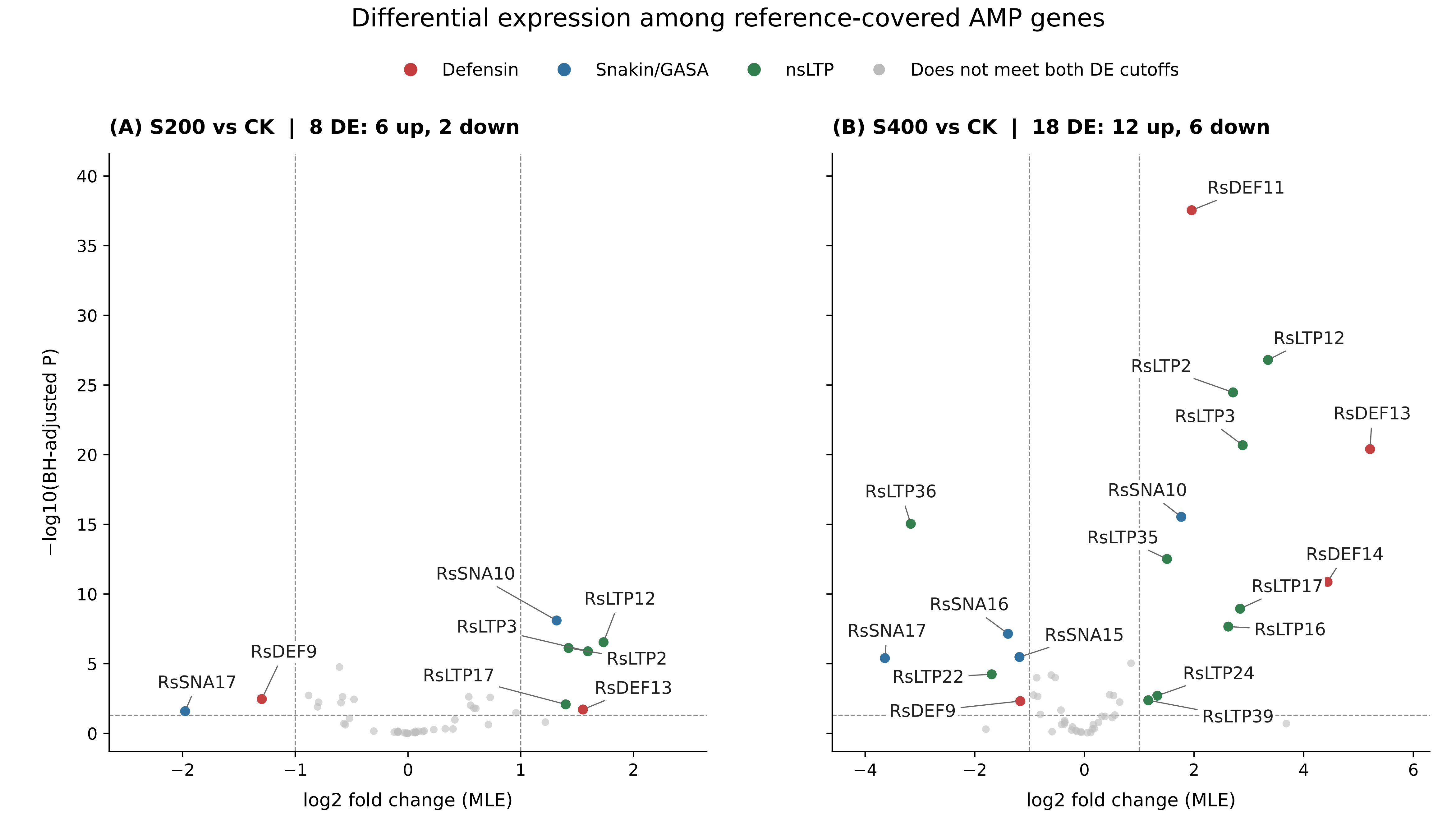

BH-adjusted P < 0.05 and |log2 fold change (MLE)| > 1; all DE genes are labeled.
66 reference-covered members out of 73 primary members; 52 passed the count filter.
Members lacking finite statistics are omitted from the scatter plots, with reasons retained in Table S14.
