# Supplementary material for: Genome-Wide Identification, Evolutionary Characterization, and Salt-Stress Expression Profiling of Antimicrobial Peptide Gene Families in the Xerophytic Recretohalophyte Reaumuria soongarica
Source: Genes (Basel). 2026 Sep 16;17(9):1126. doi: 10.3390/genes17091126 (PMC13606368; doi:10.3390/genes17091126)
Supplement: Supplementary file 1 [file genes-17-01126-s001.zip › Files/File_S8_catalogue_synced_figures_20260909.zip!/archive_historical/before_Figure3_standard_PDF_20260909/current_figures/Vector_PDF/Figure3_catalogue_synced_20260909.pdf]

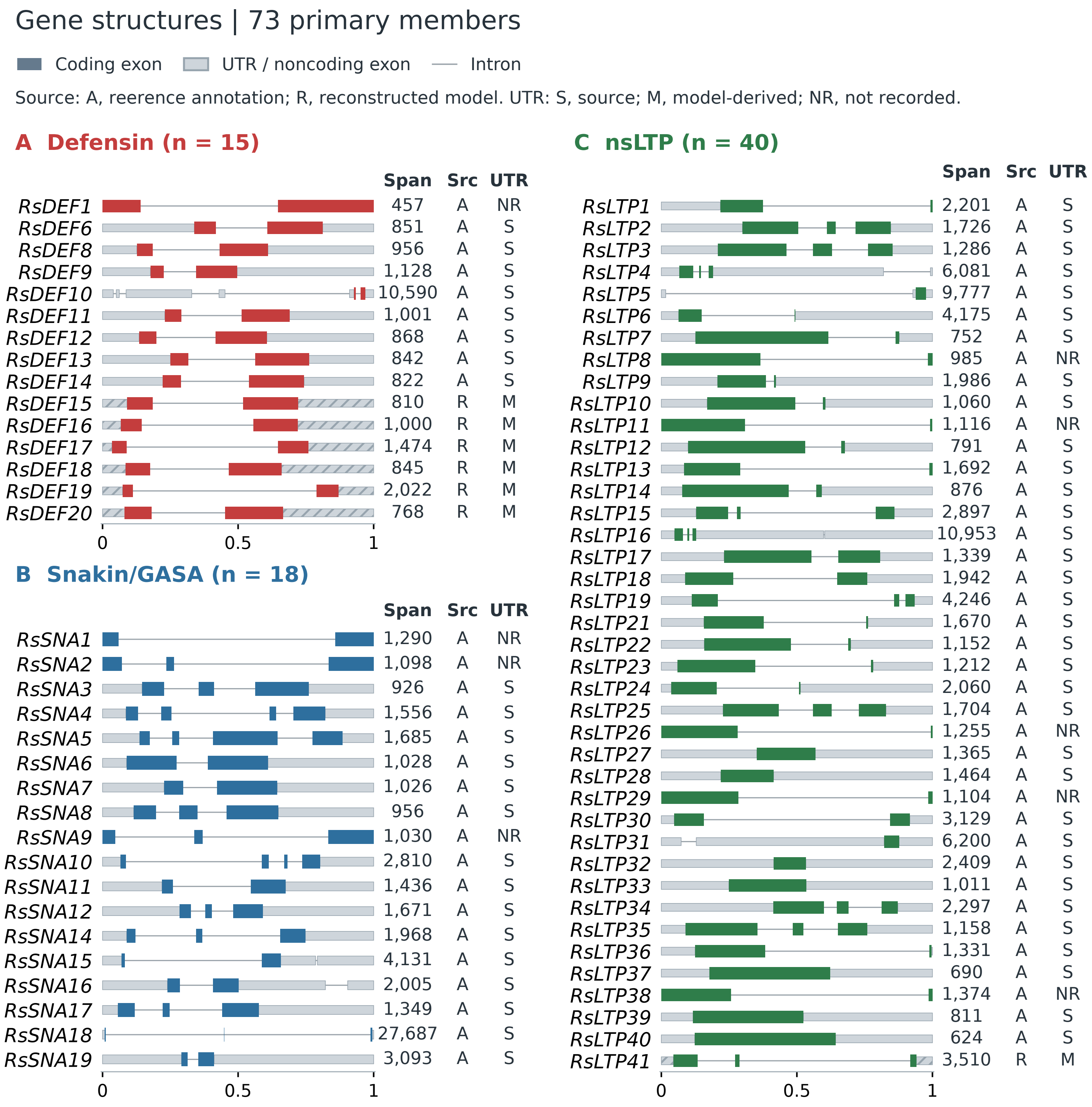
Gene structures | 73 primary members
Coding exon
UTR / noncoding exon
Intron
Source: A, reerence annotation; R, reconstructed model. UTR: S, source; M, model-derived; NR, not recorded.
A Defensin (n = 15)
Span Src UTR
RsDEF1 457 A NR
RsDEF6 851 A S
RsDEF8 956 A S
RsDEF9 1,128 A S
RsDEF10 10,590 A S
RsDEF11 1,001 A S
RsDEF12 868 A S
RsDEF13 842 A S
RsDEF14 822 A S
RsDEF15 810 R M
RsDEF16 1,000 R M
RsDEF17 1,474 R M
RsDEF18 845 R M
RsDEF19 2,022 R M
RsDEF20 768 R M
0 0.5 1
B Snakin/GASA (n = 18)
Span Src UTR
RsSNA1 1,290 A NR
RsSNA2 1,098 A NR
RsSNA3 926 A S
RsSNA4 1,556 A S
RsSNA5 1,685 A S
RsSNA6 1,028 A S
RsSNA7 1,026 A S
RsSNA8 956 A S
RsSNA9 1,030 A NR
RsSNA10 2,810 A S
RsSNA11 1,436 A S
RsSNA12 1,671 A S
RsSNA14 1,968 A S
RsSNA15 4,131 A S
RsSNA16 2,005 A S
RsSNA17 1,349 A S
RsSNA18 27,687 A S
RsSNA19 3,093 A S
0 0.5 1
C nsLTP (n = 40)
Span Src UTR
RsLTP1 2,201 A S
RsLTP2 1,726 A S
RsLTP3 1,286 A S
RsLTP4 6,081 A S
RsLTP5 9,777 A S
RsLTP6 4,175 A S
RsLTP7 752 A S
RsLTP8 985 A NR
RsLTP9 1,986 A S
RsLTP10 1,060 A S
RsLTP11 1,116 A NR
RsLTP12 791 A S
RsLTP13 1,692 A S
RsLTP14 876 A S
RsLTP15 2,897 A S
RsLTP16 10,953 A S
RsLTP17 1,339 A S
RsLTP18 1,942 A S
RsLTP19 4,246 A S
RsLTP21 1,670 A S
RsLTP22 1,152 A S
RsLTP23 1,212 A S
RsLTP24 2,060 A S
RsLTP25 1,704 A S
RsLTP26 1,255 A NR
RsLTP27 1,365 A S
RsLTP28 1,464 A S
RsLTP29 1,104 A NR
RsLTP30 3,129 A S
RsLTP31 6,200 A S
RsLTP32 2,409 A S
RsLTP33 1,011 A S
RsLTP34 2,297 A S
RsLTP35 1,158 A S
RsLTP36 1,331 A S
RsLTP37 690 A S
RsLTP38 1,374 A NR
RsLTP39 811 A S
RsLTP40 624 A S
RsLTP41 3,510 R M
0 0.5 1
