# Supplementary material for: Genome-Wide Identification, Evolutionary Characterization, and Salt-Stress Expression Profiling of Antimicrobial Peptide Gene Families in the Xerophytic Recretohalophyte Reaumuria soongarica
Source: Genes (Basel). 2026 Sep 16;17(9):1126. doi: 10.3390/genes17091126 (PMC13606368; doi:10.3390/genes17091126)
Supplement: Supplementary file 1 [file genes-17-01126-s001.zip › Files/File_S8_catalogue_synced_figures_20260909.zip!/archive_historical/promoter15_before_20260909/figures/Figure5_promoter_motif_occurrence.pdf]

Sequence motifs in upstream proxies

73 primary AMP genes · 2,000 bp · 15 double-stranded patterns

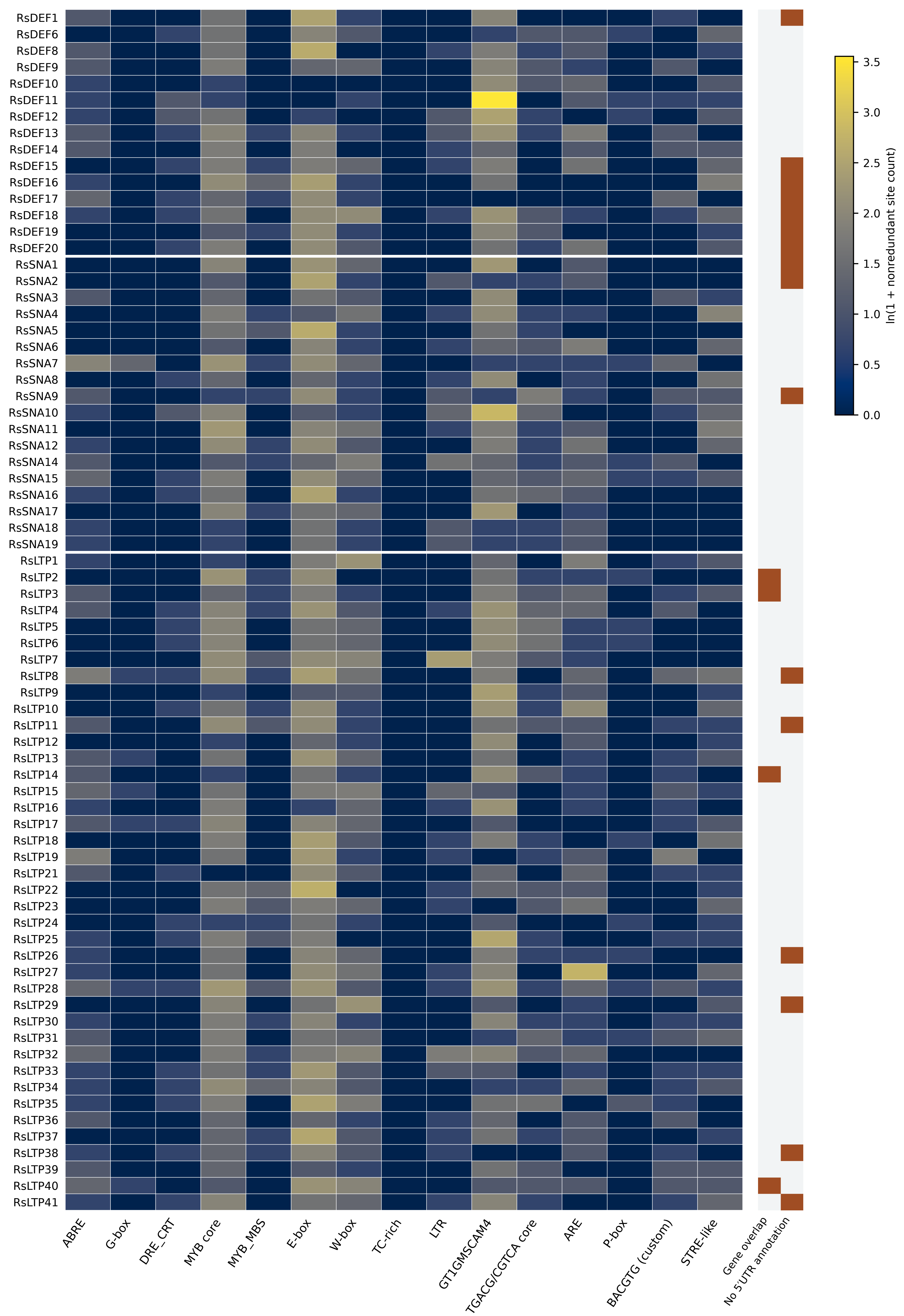

Cells show sequence matches. No binding or regulatory activity is inferred.
Brown flags indicate overlap or absent 5′UTR annotation (Table S28).
Proxies use 66 annotated gene boundaries and 7 supported new model boundaries; TSSs were not independently mapped.
