# Supplementary material for: Genome-Wide Identification, Evolutionary Characterization, and Salt-Stress Expression Profiling of Antimicrobial Peptide Gene Families in the Xerophytic Recretohalophyte Reaumuria soongarica
Source: Genes (Basel). 2026 Sep 16;17(9):1126. doi: 10.3390/genes17091126 (PMC13606368; doi:10.3390/genes17091126)
Supplement: Supplementary file 1 [file genes-17-01126-s001.zip › Files/File_S8_catalogue_synced_figures_20260909.zip!/current_figures/Vector_PDF/Figure1_catalogue_synced_20260909.pdf]

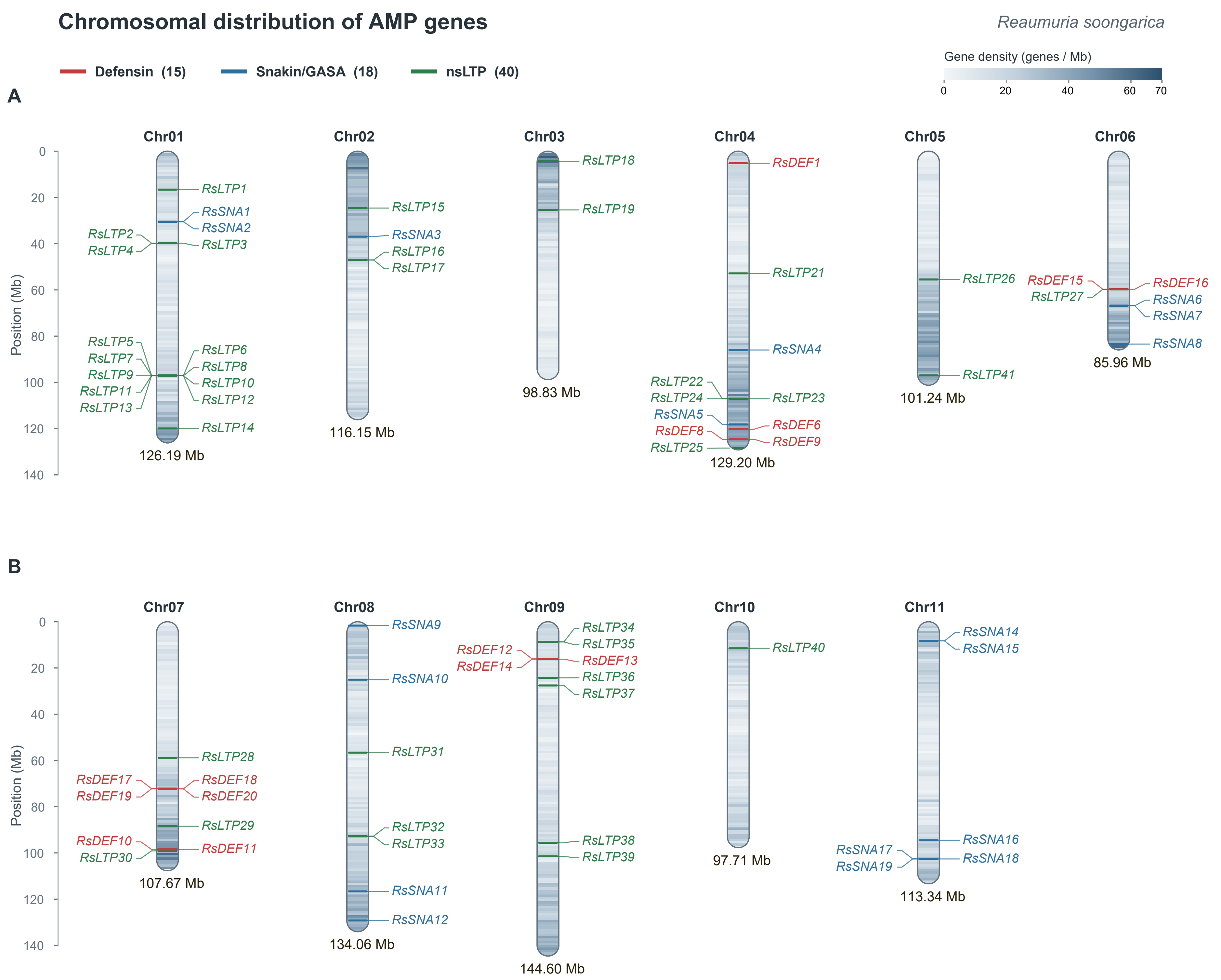
Chromosomal distribution of AMP genes
Reaumuria soongarica
Defensin (15)
Snakin/GASA (18)
nsLTP (40)
Gene density (genes / Mb)
0
20
40
60
70
A
Chr01
Chr02
Chr03
Chr04
Chr05
Chr06
Position (Mb)
0
20
40
60
80
100
120
140
RsLTP1
RsSNA1
RsSNA2
RsLTP2
RsLTP4
RsLTP3
RsLTP5
RsLTP7
RsLTP9
RsLTP11
RsLTP13
RsLTP6
RsLTP8
RsLTP10
RsLTP12
RsLTP14
126.19 Mb
RsLTP15
RsSNA3
RsLTP16
RsLTP17
116.15 Mb
RsLTP18
RsLTP19
98.83 Mb
RsDEF1
RsLTP21
RsSNA4
RsLTP22
RsLTP24
RsLTP23
RsSNA5
RsDEF8
RsLTP25
RsDEF6
RsDEF9
129.20 Mb
RsLTP26
RsLTP41
101.24 Mb
RsDEF15
RsLTP27
RsDEF16
RsSNA6
RsSNA7
RsSNA8
85.96 Mb
B
Chr07
Chr08
Chr09
Chr10
Chr11
Position (Mb)
0
20
40
60
80
100
120
140
RsLTP28
RsDEF17
RsDEF19
RsDEF18
RsDEF20
RsLTP29
RsDEF10
RsLTP30
RsDEF11
107.67 Mb
RsSNA9
RsSNA10
RsLTP31
RsLTP32
RsLTP33
RsSNA11
RsSNA12
134.06 Mb
RsLTP34
RsLTP35
RsDEF12
RsDEF14
RsDEF13
RsLTP36
RsLTP37
RsLTP38
RsLTP39
144.60 Mb
RsLTP40
97.71 Mb
RsSNA14
RsSNA15
RsSNA16
RsSNA17
RsSNA19
RsSNA18
113.34 Mb
