# Supplementary material for: Genome-Wide Identification, Evolutionary Characterization, and Salt-Stress Expression Profiling of Antimicrobial Peptide Gene Families in the Xerophytic Recretohalophyte Reaumuria soongarica
Source: Genes (Basel). 2026 Sep 16;17(9):1126. doi: 10.3390/genes17091126 (PMC13606368; doi:10.3390/genes17091126)
Supplement: Supplementary file 1 [file genes-17-01126-s001.zip › Files/File_S8_catalogue_synced_figures_20260909.zip!/current_figures/Vector_PDF/Figure2_catalogue_synced_20260909.pdf]

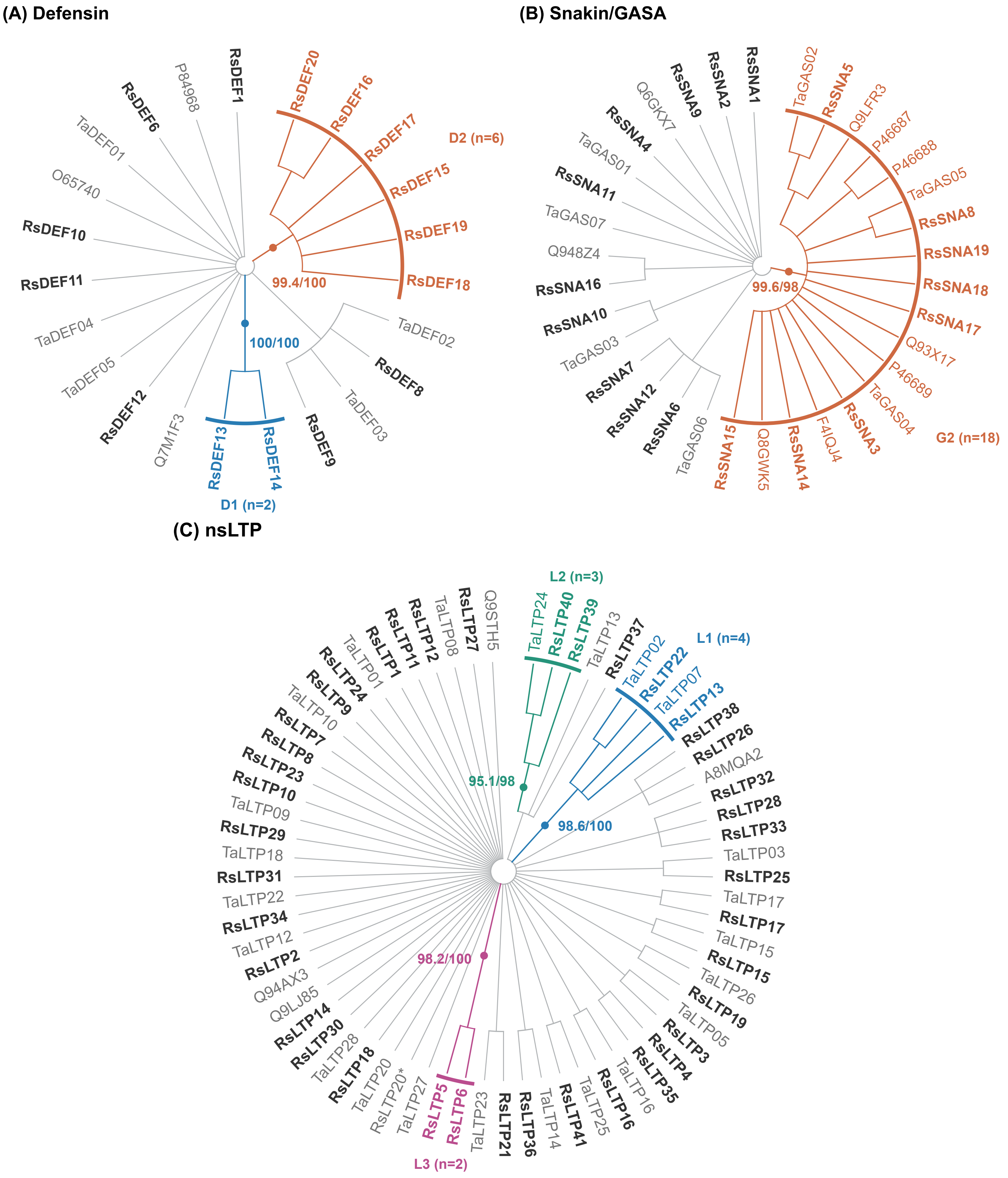

(A) Defensin
(B) Snakin/GASA
(C) nsLTP
RsDEF20
RsDEF16
RsDEF17
RsDEF15
RsDEF19
RsDEF18
D2 (n=6)
99.4/100
RsDEF1
P84968
RsDEF6
TaDEF01
O65740
RsDEF10
RsDEF11
TaDEF04
TaDEF05
RsDEF12
Q7M1F3
RsDEF13
RsDEF14
D1 (n=2)
100/100
RsDEF9
TaDEF03
RsDEF8
TaDEF02
RsSNA1
RsSNA2
RsSNA9
Q6GKX7
RsSNA4
TaGAS01
RsSNA11
TaGAS07
Q948Z4
RsSNA16
RsSNA10
TaGAS03
RsSNA7
RsSNA12
RsSNA6
TaGAS06
TaGAS02
RsSNA5
Q9LFR3
P46687
P46688
TaGAS05
RsSNA8
RsSNA19
RsSNA18
RsSNA17
Q93X17
P46689
TaGAS04
RsSNA3
F4IQJ4
RsSNA14
Q8GWK5
RsSNA15
G2 (n=18)
99.6/98
L2 (n=3)
TaLTP24
RsLTP40
RsLTP39
TaLTP13
RsLTP37
L1 (n=4)
TaLTP02
RsLTP22
TaLTP07
RsLTP13
RsLTP38
RsLTP26
A8MQA2
RsLTP32
RsLTP28
RsLTP33
TaLTP03
RsLTP25
TaLTP17
RsLTP17
TaLTP15
RsLTP15
TaLTP26
RsLTP19
TaLTP05
RsLTP3
RsLTP4
RsLTP35
TaLTP16
RsLTP16
TaLTP25
RsLTP41
TaLTP14
RsLTP36
RsLTP21
TaLTP23
RsLTP6
RsLTP5
L3 (n=2)
TaLTP27
RsLTP20*
TaLTP20
RsLTP18
TaLTP28
RsLTP30
RsLTP14
Q9LJ85
Q94AX3
RsLTP2
TaLTP12
RsLTP34
TaLTP22
RsLTP31
TaLTP18
RsLTP29
TaLTP09
RsLTP10
RsLTP23
RsLTP8
RsLTP7
TaLTP10
RsLTP9
RsLTP24
TaLTP01
RsLTP1
RsLTP11
RsLTP12
TaLTP08
RsLTP27
Q9STH5
95.1/98
98.6/100
98.2/100
