# Supplementary material for: Genome-Wide Identification, Evolutionary Characterization, and Salt-Stress Expression Profiling of Antimicrobial Peptide Gene Families in the Xerophytic Recretohalophyte Reaumuria soongarica
Source: Genes (Basel). 2026 Sep 16;17(9):1126. doi: 10.3390/genes17091126 (PMC13606368; doi:10.3390/genes17091126)
Supplement: Supplementary file 1 [file genes-17-01126-s001.zip › Files/File_S8_catalogue_synced_figures_20260909.zip!/current_figures/Vector_PDF/Figure4_catalogue_synced_20260909.pdf]

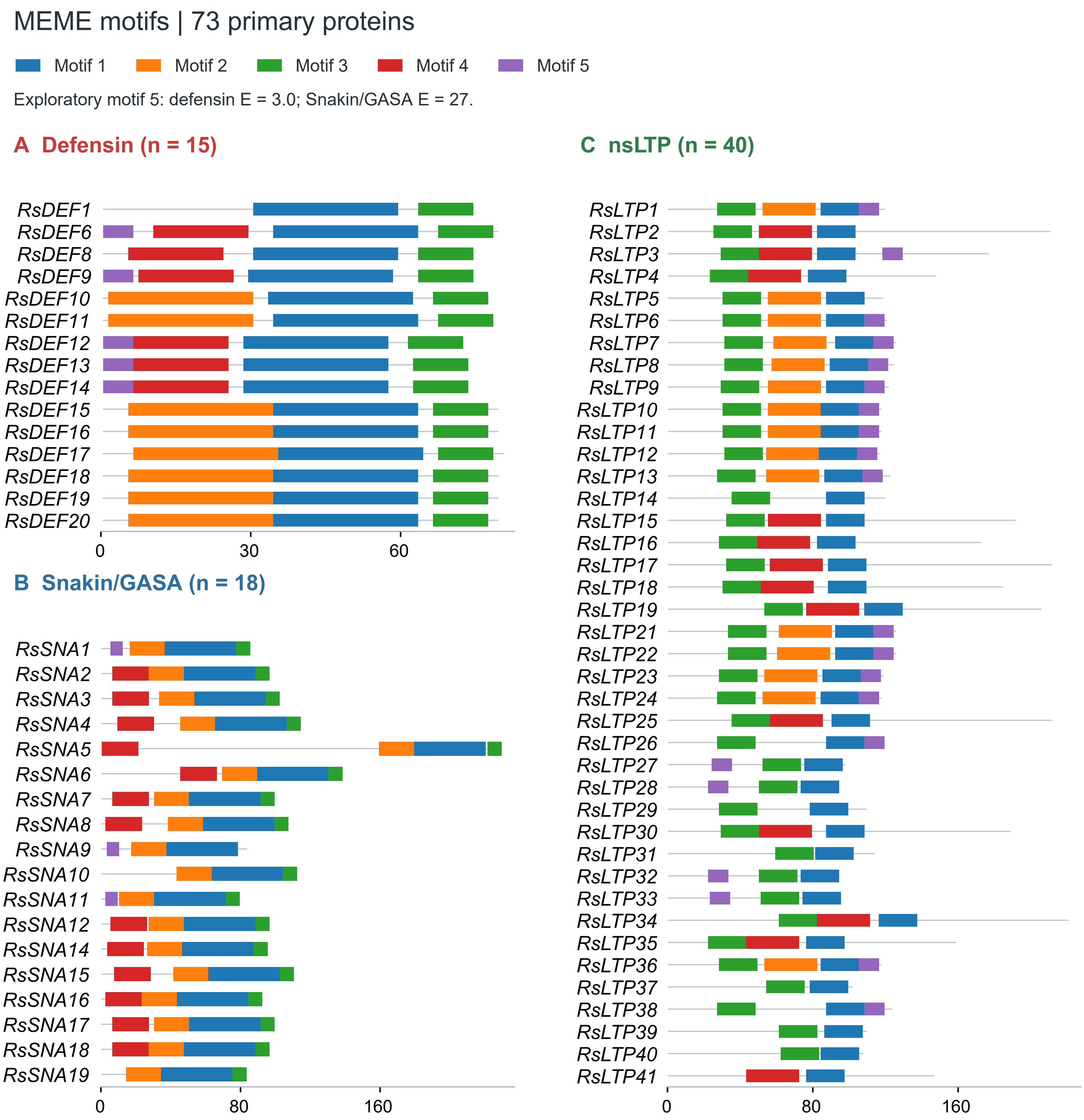
MEME motifs | 73 primary proteins
Motif 1
Motif 2
Motif 3
Motif 4
Motif 5
Exploratory motif 5: defensin E = 3.0; Snakin/GASA E = 27.
A Defensin (n = 15)
RsDEF1
RsDEF6
RsDEF8
RsDEF9
RsDEF10
RsDEF11
RsDEF12
RsDEF13
RsDEF14
RsDEF15
RsDEF16
RsDEF17
RsDEF18
RsDEF19
RsDEF20
0
30
60
B Snakin/GASA (n = 18)
RsSNA1
RsSNA2
RsSNA3
RsSNA4
RsSNA5
RsSNA6
RsSNA7
RsSNA8
RsSNA9
RsSNA10
RsSNA11
RsSNA12
RsSNA14
RsSNA15
RsSNA16
RsSNA17
RsSNA18
RsSNA19
0
80
160
C nsLTP (n = 40)
RsLTP1
RsLTP2
RsLTP3
RsLTP4
RsLTP5
RsLTP6
RsLTP7
RsLTP8
RsLTP9
RsLTP10
RsLTP11
RsLTP12
RsLTP13
RsLTP14
RsLTP15
RsLTP16
RsLTP17
RsLTP18
RsLTP19
RsLTP21
RsLTP22
RsLTP23
RsLTP24
RsLTP25
RsLTP26
RsLTP27
RsLTP28
RsLTP29
RsLTP30
RsLTP31
RsLTP32
RsLTP33
RsLTP34
RsLTP35
RsLTP36
RsLTP37
RsLTP38
RsLTP39
RsLTP40
RsLTP41
0
80
160
