# Supplementary material for: Genome-Wide Identification, Evolutionary Characterization, and Salt-Stress Expression Profiling of Antimicrobial Peptide Gene Families in the Xerophytic Recretohalophyte Reaumuria soongarica
Source: Genes (Basel). 2026 Sep 16;17(9):1126. doi: 10.3390/genes17091126 (PMC13606368; doi:10.3390/genes17091126)
Supplement: Supplementary file 1 [file genes-17-01126-s001.zip › Files/File_S8_catalogue_synced_figures_20260909.zip!/current_figures/Vector_PDF/Figure7_catalogue_synced_20260909.pdf]

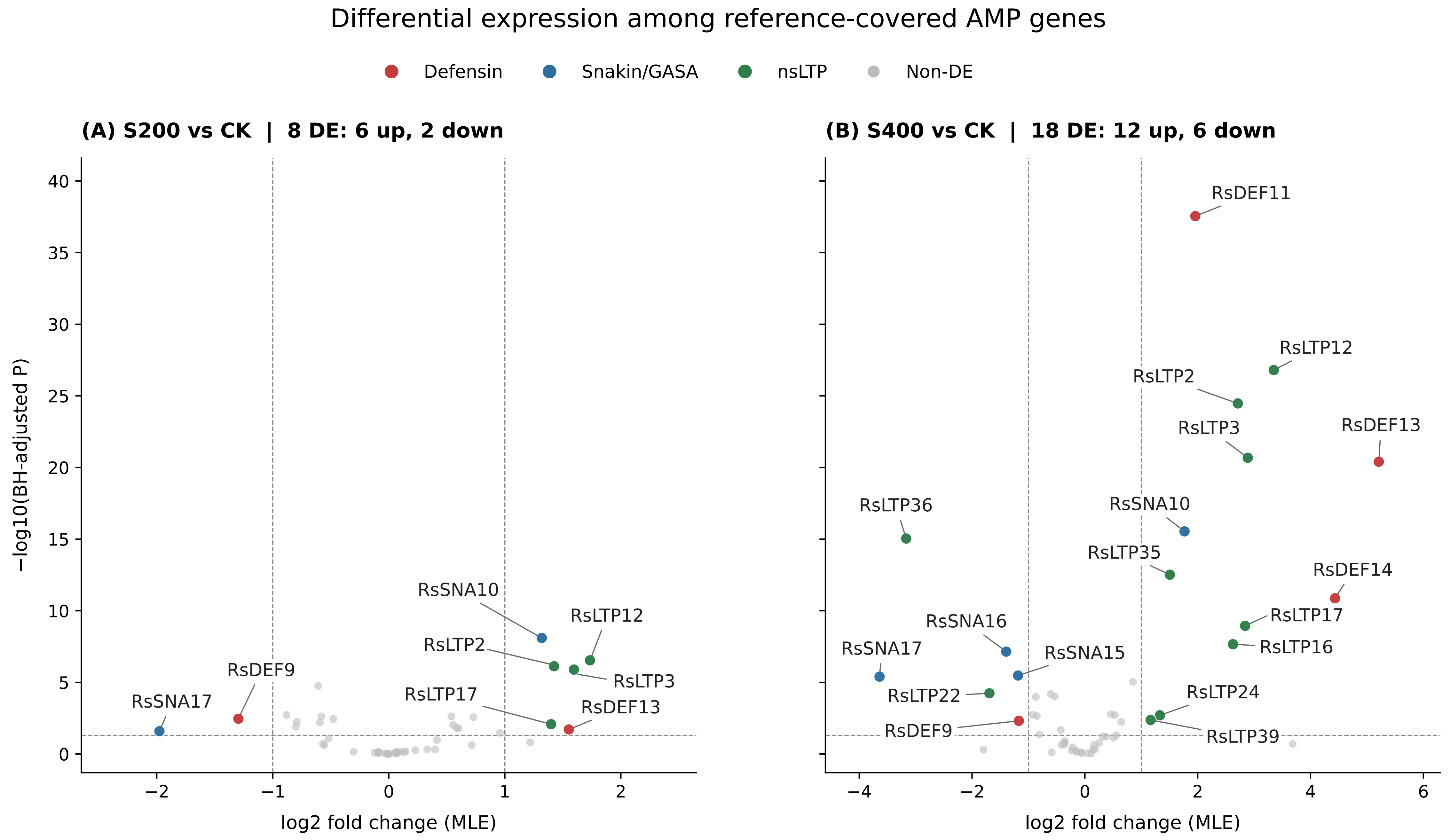

Differential expression among reference-covered AMP genes
Defensin
Snakin/GASA
nsLTP
Non-DE
(A) S200 vs CK | 8 DE: 6 up, 2 down
(B) S400 vs CK | 18 DE: 12 up, 6 down
−log10(BH-adjusted P)
log2 fold change (MLE)
RsSNA10
RsLTP12
RsLTP2
RsLTP3
RsDEF9
RsSNA17
RsLTP17
RsDEF13
RsDEF11
RsLTP36
RsSNA16
RsSNA15
RsLTP22
RsLTP35
RsDEF14
RsLTP16
RsLTP24
RsLTP39
